# Discovery and Optimization of Indoline-Based Compounds as Dual 5-LOX/sEH Inhibitors: *In Vitro* and *In Vivo* Anti-Inflammatory Characterization

**DOI:** 10.1021/acs.jmedchem.2c00817

**Published:** 2022-11-01

**Authors:** Ida Cerqua, Simona Musella, Lukas Klaus Peltner, Danilo D’Avino, Veronica Di Sarno, Elisabetta Granato, Vincenzo Vestuto, Rita Di Matteo, Simona Pace, Tania Ciaglia, Rossella Bilancia, Gerardina Smaldone, Francesca Di Matteo, Simone Di Micco, Giuseppe Bifulco, Giacomo Pepe, Manuela Giovanna Basilicata, Manuela Rodriquez, Isabel M. Gomez-Monterrey, Pietro Campiglia, Carmine Ostacolo, Fiorentina Roviezzo, Oliver Werz, Antonietta Rossi, Alessia Bertamino

**Affiliations:** † Department of Pharmacy, University Federico II of Naples, Via D. Montesano 49, 80131 Naples, Italy; ‡ Department of Pharmacy, University of Salerno, Via G. Paolo II 132, 84084 Fisciano, Salerno, Italy; § Department of Pharmaceutical/Medicinal Chemistry, Institute of Pharmacy, Friedrich-Schiller-University, Philosophenweg 14, D-07743 Jena, Germany; ∥ European Biomedical Research Institute (EBRIS), Via S. De Renzi 50, 84125 Salerno, Italy

## Abstract

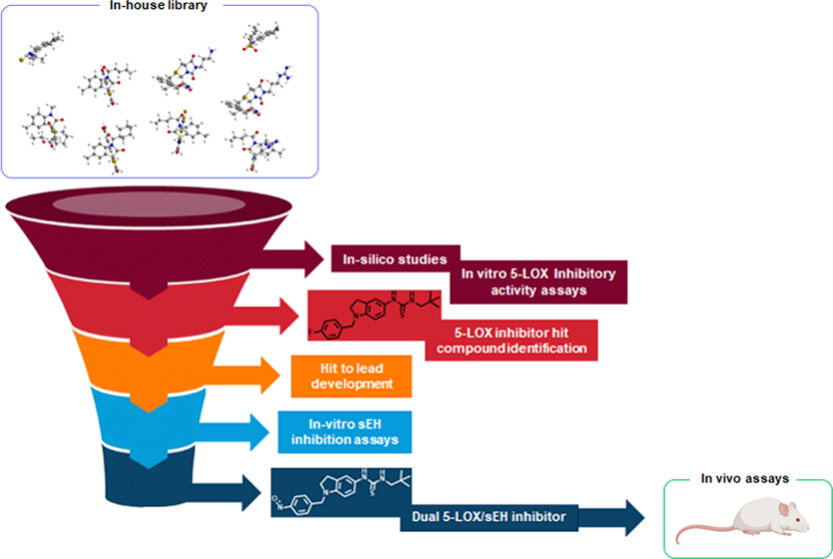

The design of multitarget drugs represents a promising strategy in medicinal chemistry and seems particularly suitable for the discovery of anti-inflammatory drugs. Here, we describe the identification of an indoline-based compound inhibiting both 5-lipoxygenase (5-LOX) and soluble epoxide hydrolase (sEH). *In silico* analysis of an in-house library identified nine compounds as potential 5-LOX inhibitors. Enzymatic and cellular assays revealed the indoline derivative **43** as a notable 5-LOX inhibitor, guiding the design of new analogues. These compounds underwent extensive *in vitro* investigation revealing dual 5-LOX/sEH inhibitors, with **73** showing the most promising activity (IC_50_s of 0.41 ± 0.01 and 0.43 ± 0.10 μM for 5-LOX and sEH, respectively). When challenged in vivo in zymosan-induced peritonitis and experimental asthma in mice, compound **73** showed remarkable anti-inflammatory efficacy. These results pave the way for the rational design of 5-LOX/sEH dual inhibitors and for further investigation of their potential use as anti-inflammatory agents.

## Introduction

Inflammation encompasses multiple physiological and pathological processes, triggered by converging pathways that generate a network of proinflammatory and proresolving mediators, involving different cell types and cellular responses.^1^ The arachidonic acid (AA) cascade is a key biochemical pathway for pharmacologically targeting inflammatory diseases. Upon release from membrane phospholipids, AA is further metabolized via three divergent pathways: (a) transformation into proinflammatory prostaglandins (PGs) and thromboxane initially mediated by cyclooxygenase (COX) enzyme; (b) conversion into leukotrienes (LTs) mediated by 5-lipoxygenase (5-LOX) and by 5-LOX-activating protein (FLAP); and (c) metabolization by cytochrome P450 monooxygenases (CYP450) into the proinflammatory 20-hydroxyeicosatetraenoic acid (20-HETE)^2^ and the anti-inflammatory epoxyeicosatrienoic acids (EETs). The latter are hydrolyzed by soluble epoxide hydrolase (sEH)^2,3^ to dihydroxyeicosatrienoic acids (DiHETrEs) with primarily proinflammatory effects.^4^ The balance among these mediators determines the evolution of the inflammatory process toward progression and/or chronicity or toward resolution.

Traditionally, COX inhibitors (nonsteroidal anti-inflammatory drugs, NSAIDs) represent the reference drugs in this field and are also among the most used therapeutics worldwide. However, severe gastrointestinal or cardiovascular side effects upon prolonged use of these drugs may limit their therapeutic benefits.^5,6^ Therefore, there are continuous efforts in the search for alternative pharmacological approaches characterized by a lower incidence of adverse reactions and improved efficacy.^7,8^

LTs are proinflammatory mediators involved in many allergic^9−11^ and nonallergic diseases.^12−15^ Two different approaches have been adopted to reduce the LT-mediated response: the antagonism of the Cys-LT_1_ receptor and the inhibition of LT biosynthesis.^16^ Zafirlukast and montelukast, for instance, are Cys-LT_1_ receptor antagonists approved for the treatment of asthma, rhinitis, and other allergic diseases. However, these therapeutics are penalized by several side effects and by unsatisfactory biological responses in certain groups of patients.^17,18^ On the other hand, the inhibition of 5-LOX or FLAP is considered a suitable strategy for decreasing the LT-mediated inflammatory response. The 5-LOX inhibitor zileuton is the only LT biosynthesis inhibitor, approved for the treatment of bronchial asthma, but due to its poor pharmacokinetics, its use is strongly limited by liver toxicity.^19^

The third pathway of AA leads to anti-inflammatory EETs, which contribute to the homeostatic equilibrium of biological processes. In particular, they exert anti-inflammatory,^20^ analgesic,^21^ fibrinolytic,^22^ antimigratory,^23^ and angiogenic^24^ effects. The tissue and plasma levels of EETs are increased by sEH inhibitors, which also block the DiHETrE synthesis, thereby counteracting inflammation.^25^ Indeed, the use of sEH inhibitors is proposed as a proper therapeutic strategy in several pathological animal models.^25,26^ Nevertheless, the clinical use of molecules interfering with one of the AA metabolic pathways is penalized by the wide pool of activities exerted by the downstream lipid mediators, which act as both proinflammatory and proresolving agents.^27^ Furthermore, targeting a single enzyme in the eicosanoid cascade can cause substrate shunting and amplification of alternative pathways, resulting in decreased efficacy and increased side effects.^28^ This is why polypharmacological approaches appear particularly useful in the design of anti-inflammatory drugs.^29,30^ Multitarget drugs, indeed, accomplish a balanced modulation of eicosanoid levels and largely suppress shunting and/or redirection phenomena.^31−33^ In this context, 5-LOX/sEH dual inhibitors offer the advantage of blocking the proinflammatory LT production and simultaneously increasing the anti-inflammatory eicosanoid (i.e., EETs) levels.^34^ In the present manuscript, we describe the design, the synthesis, and the pharmacological characterization of indoline-based dual 5-LOX/sEH inhibitors. The development of these molecules was carried out by an integrated approach of *in silico* and *in vitro* assays starting from an in-house molecular library. First, 53 bicyclic compounds from our library were cherry-picked and evaluated in silico, since the benzo[*b*]thiophene moiety is considered the pharmacophore of zileuton.^35^ This approximation is due to the absence of zileuton/5-LOX experimentally resolved structure in the protein data bank (http://www.rcsb.org/),^36^ so only the binding mode, derived from recent molecular docking studies, could be taken into account.^37,38^ From computer-aided analysis, nine derivatives were filtered and tested *in vitro* for their 5-LOX inhibitory properties leading to the identification of the indoline compound **43** as the hit compound. Therefore, we decided to design a second library of compound **43** analogues. Extensive *in vitro* testing of this library highlighted a remarkable dual inhibitory profile over 5-LOX and sEH for the synthesized molecules, with **73** emerging as the most potent compound. *In silico* studies also highlighted structure–activity relationship clues concerning the 5-LOX/sEH dual inhibition. When challenged *in vivo*, compound **73** showed remarkable efficacy in both peritonitis and asthma murine models, confirming the potential of these dual inhibitors in treating inflammatory diseases.

## Results and Discussion

### Chemistry

**Scheme 1:**
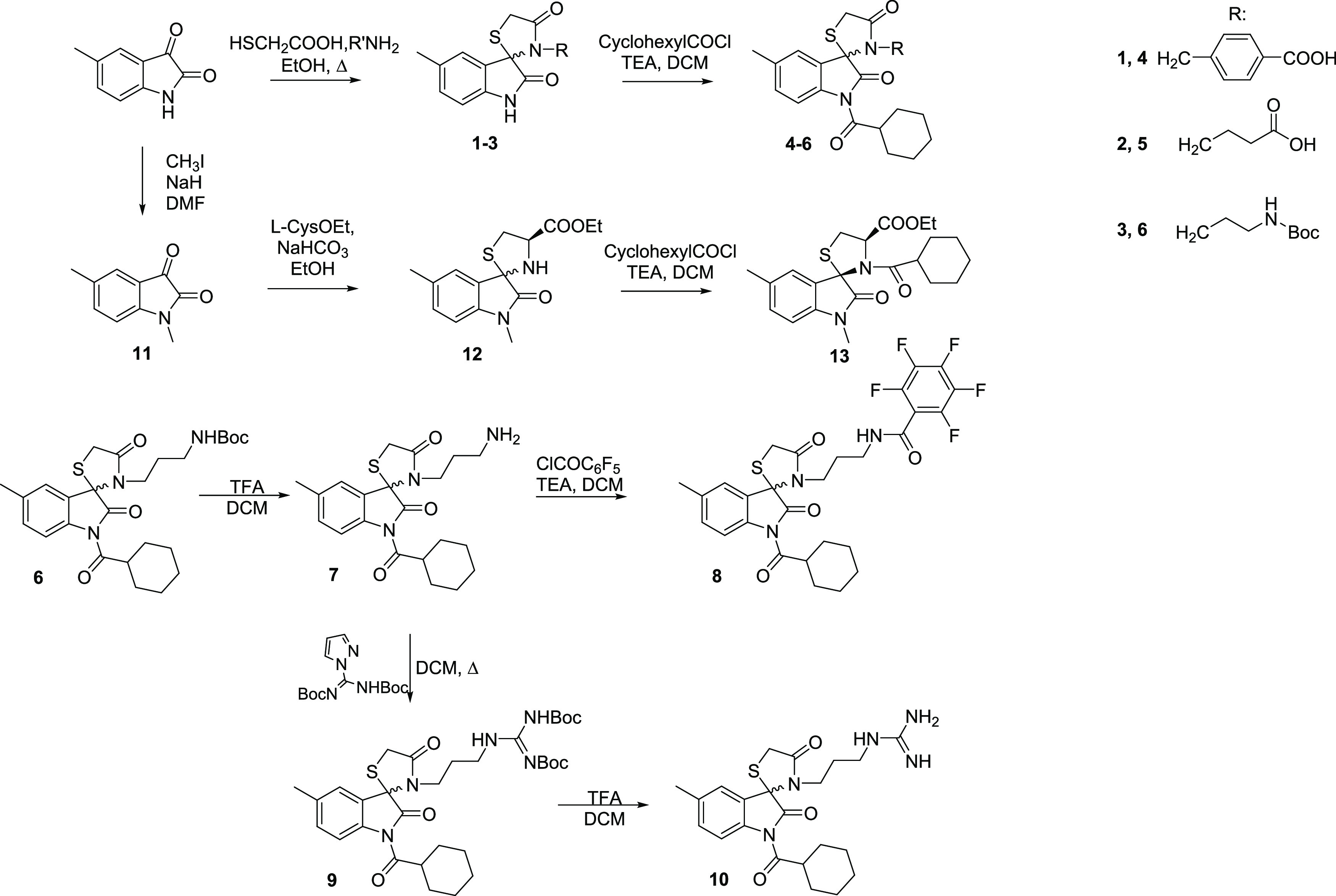
Synthesis of Spiro[indoline-3,2′-thiazolidine] Derivatives **4**, **5**, **8**, **10**, and **13**

Spiro[indoline-3,2′-thiazolidine] derivatives were synthesized according to Scheme 1.

5-Methylindoline-2,3-dione was reacted with mercaptoacetic acid in the presence of a stoichiometric amount of 4-(aminomethyl)benzoic acid, or 4-aminobutanoic acid, or *tert*-butyl(3-aminopropyl)carbamate, to obtain in one step intermediates **1**–**3** in 55, 59, and 85% yields, respectively. These intermediates were acylated at the N-1 position with cyclohexanecarbonyl chloride, giving final compounds **4** and **5** in 55 and 57% yields, respectively, and the Boc-protected intermediate **6** in a 72% yield. Intermediate **6** was then subjected to Boc deprotection in dichloromethane (DCM)/trifluoroacetic acid (TFA) affording the amino derivative **7**, which was coupled with 2,3,4,5,6-pentafluorobenzoyl chloride leading to **8** (65% yield). Besides, the amino group of intermediate **7** was subjected to guanidination using *tert*-butyl(((*tert*-butoxycarbonyl)amino)(1*H*-pyrazol-1-yl)methylene)carbamate to obtain Boc-protected derivative **9** in a 45% yield. Boc removal of the resulting intermediate gave **10** in a 92% yield.

5-Methylindoline-2,3-dione also underwent N-1 alkylation with methyl iodide affording derivative **11**, which was treated with l-Cys-OEt in ethanol and NaHCO_3_ to give the corresponding spiro thiazolidine intermediate **12** as a diastereoisomeric mixture (3/1 2′*R*,4′*R*/2′*S*,4′*R* as ratio) in an 85% yield. Acylation of this diastereoisomeric mixture using cyclohexanecarbonyl chloride yielded the final compound **13** (45% yield) as a pure isomer (2′*S*,4′*R*), in accordance with the literature.^39^

**Scheme 2:**
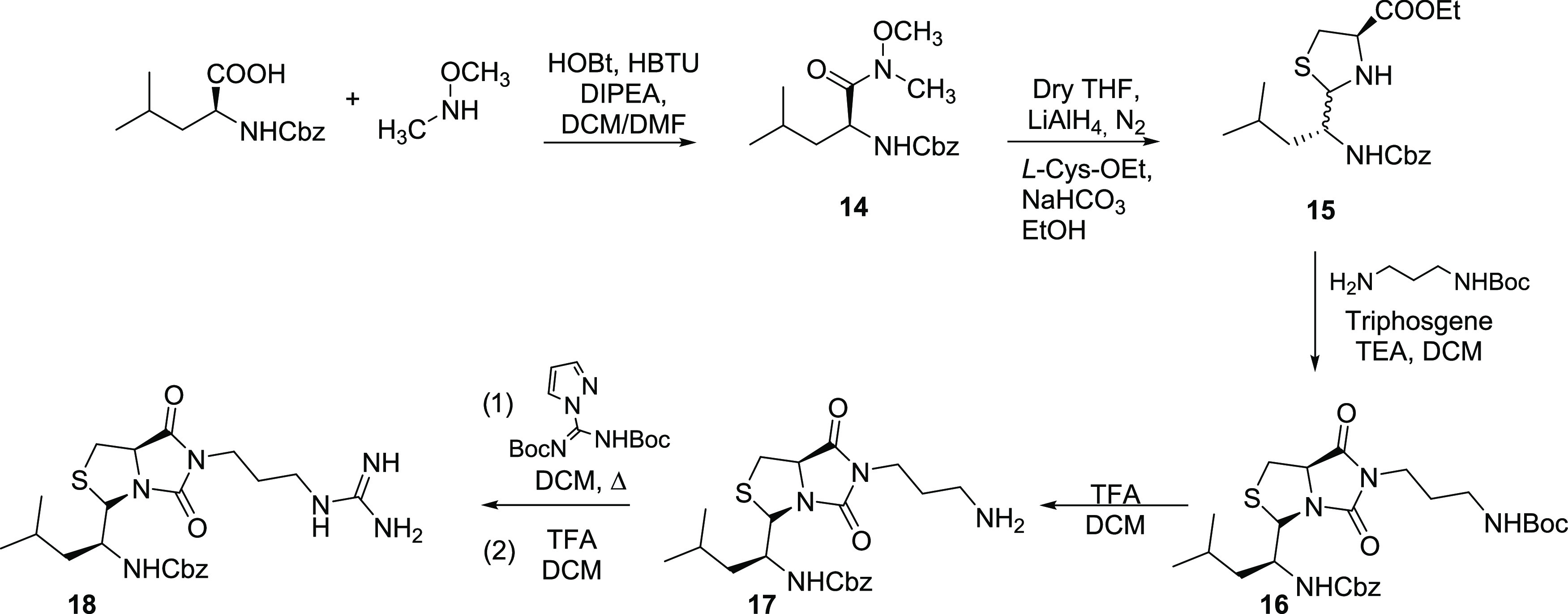
Synthesis of Hexahydroimidazo[1,5-*c*]thiazolidine Derivatives **17** and **18**

Derivatives **17** and **18** were obtained, as depicted in Scheme 2. Starting from *N*-Cbz-l-Leu-OH, reduction of the carboxylic acid function to aldehyde was performed.^40^ The aldehyde intermediate was reacted with l-Cys-OEt, using the same conditions described above giving thiazolidine **15** as a diastereoisomeric mixture in a 62% yield (3/2 2*R*,4*R*,2′*S*/2*S*,4*R*,2′*S* as ratio). Reaction of **15** with triphosgene and *tert*-butyl *N*-(3-aminopropyl)carbamate followed by a spontaneous intramolecular cyclization gave hydantoin **16**, which, upon the removal of the Boc protecting group, afforded the final derivative **17** as a pure diastereoisomer (3*S*,7a*R*,1′*S*) in a 55% yield. Stereochemistry was assigned by ROESY NMR spectra (Figure S16), assuming the retention of configuration for the amino acid moiety (C-7a and C-1′). A correlation between H-1′ (3.94 ppm) and H-3 (5.22 ppm) was observed, highlighting a cis configuration. On the other hand, the lack of peak correlation between H-7a (3.36 ppm) and H-3 indicates a trans configuration for the two protons. Finally, guanidination of **17**, followed by Boc removal, gave the final compound **18** in a 55% yield.

**Scheme 3:**
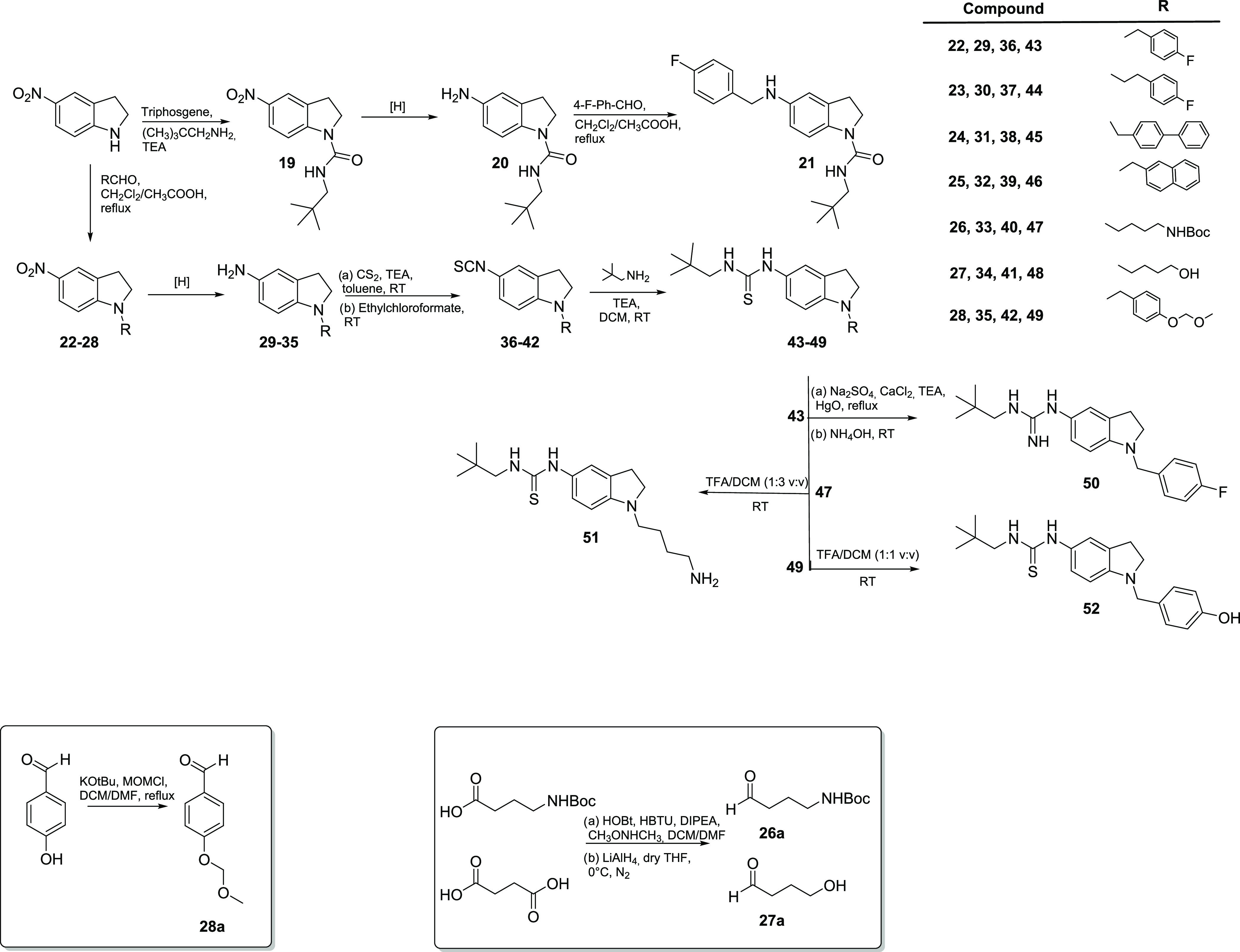
Synthesis of Indoline Derivatives **21**, **43**–**46**, and **48–52**

Derivatives **21**, **43**–**46**, and **48–52** were obtained, as shown in Scheme 3. Starting from 5-nitroindoline, different synthetic approaches were used to decorate both the N-1 and the C-5 positions. Using triphosgene and 2,2-dimethylpropan-1-amine, the urea derivative **19** was obtained in a 66% yield. Then, a continuous flow hydrogenation reaction provided the corresponding amine intermediate **20** (61% yield), which was converted to the final compound **21** (58% yield) through a reductive amination reaction with 4-fluorobenzaldehyde. Under the same conditions, N-1 alkylation of 5-nitroindoline was attained, using commercially available aldehydes, leading to intermediates **22**–**25** in 62–92% yields. Intermediates **26–28** were synthesized using the same protocol and the modified aldehydes **26a**, **27a**, and **28a** obtained, as described in Scheme 3. Continuous flow hydrogenation of these compounds afforded the corresponding amines **29**–**35** in 58–95% yields. Intermediates **29**–**35** were converted to isothiocyanates **36**–**42** by reaction with CS_2_ in toluene followed by treatment with ethyl chloroformate (48–77% yields). Reaction of these intermediates with 2,2-dimethylpropan-1-amine yielded thiourea analogues **43–49** in 52–67% yields. Compound **43** was further modified to guanidine **50** by reaction with HgO, in the presence of Na_2_SO_4_ and CaCl_2_, followed by the addition of NH_4_OH, as previously described.^41^ Compound **47** underwent Boc removal as described before, giving the final compound **51**. The final compound **52** was obtained from **49**, after removal of the MOM protecting group in acid conditions.

**Scheme 4:**
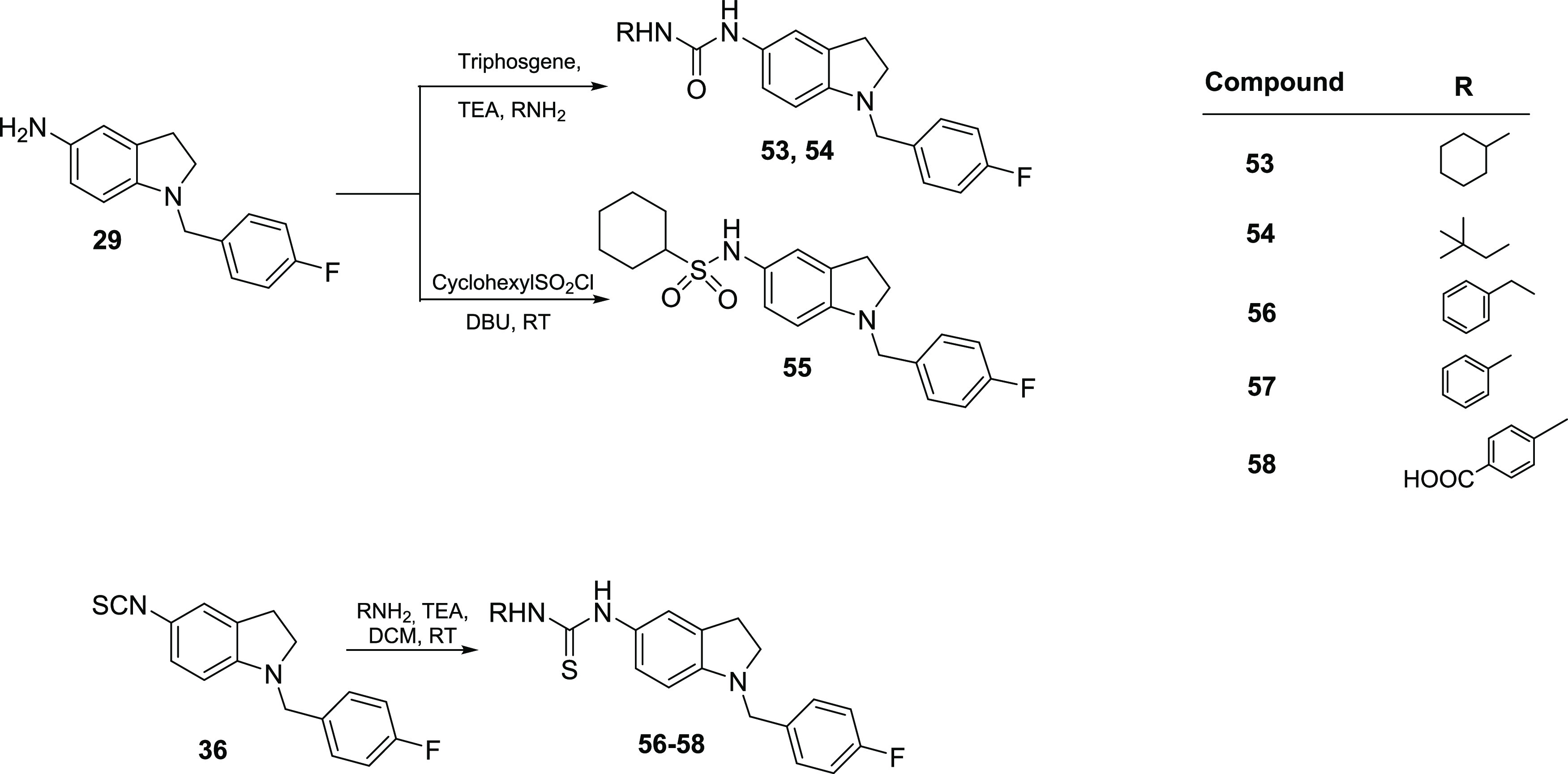
Synthesis of Indoline Derivatives **53–58**

Indoline derivatives **53**–**58** were synthesized according to Scheme 4. Intermediate **29** was converted to its carbamic chloride by reaction with triphosgene, which, upon reaction with cyclohexylamine and 2,2-dimethyl-1-propanamine, gave the final urea compounds **53** and **54** in 72 and 78% yields, respectively. Coupling of **29** with cyclohexanesulfonyl chloride afforded, instead, the final compound **55**, as previously described.^41^

The previously obtained intermediate **36** was converted to aromatic thiourea analogues **56**–**58** by the same method described above in 58–67% yields.

**Scheme 5:**
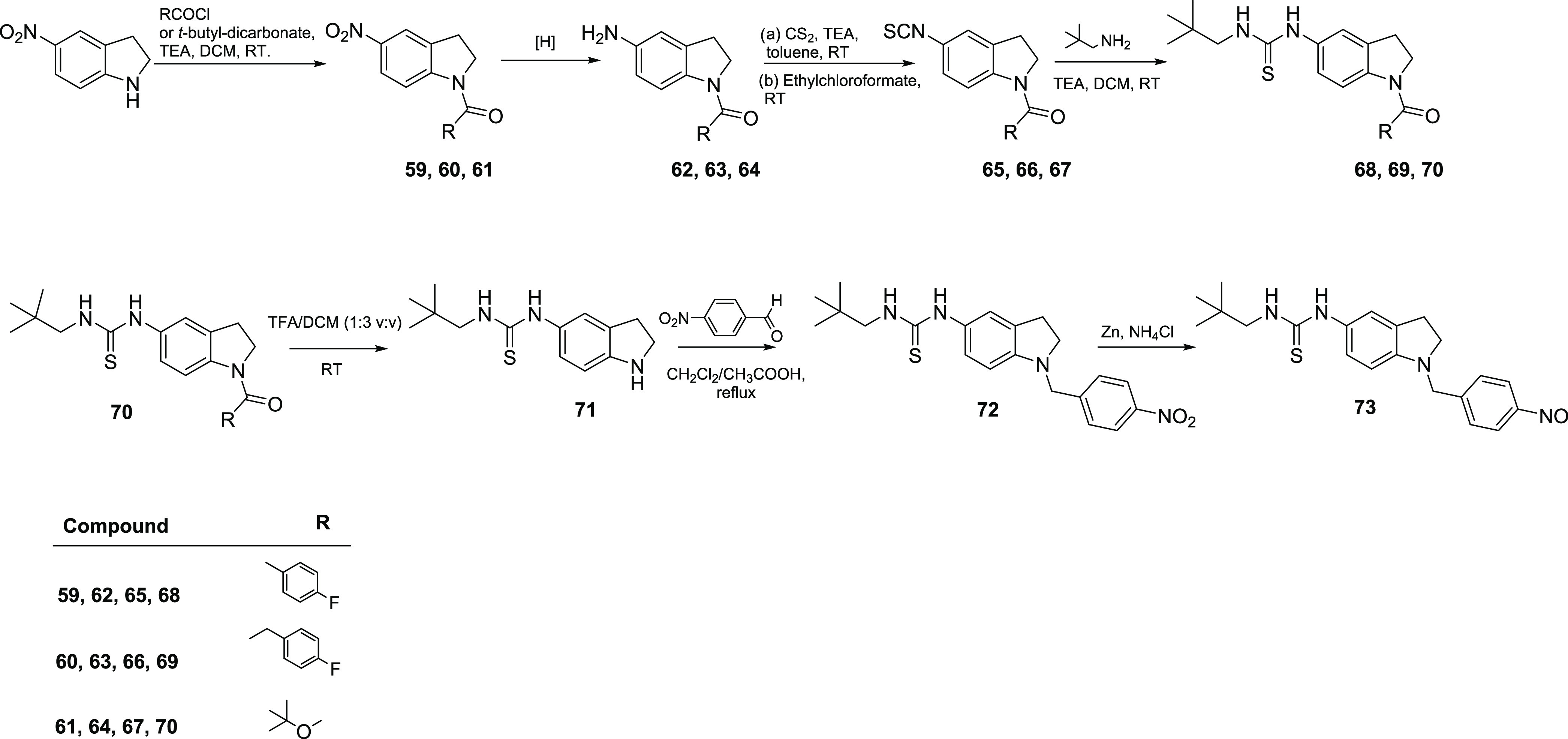
Synthesis of Indoline Derivatives **68**, **69**, and **73**

5-Nitroindoline was also decorated at the N-1 by acylation with 4-fluorobenzoyl chloride and 4-fluorophenylacetyl chloride to give intermediates **59** and **60** in 91 and 88% yields, respectively, and using di-tert-butyl dicarbonate to give the *N*-Boc intermediate **61** in an 88% yield (Scheme 5). The use of a continuous flow hydrogenation protocol followed by conversion to isothiocyanates **65–67** (58–62% yields) and reaction with 2,2-dimethylpropan-1-amine gave the final compounds **68** and **69**, which were isolated in 65 and 58% yields, respectively. On the other hand, intermediate **70** was further reacted using TFA for the removal of the Boc protecting group. The resulting intermediate **71** was sequentially subjected to reductive amination to form intermediate **72** in a 68% yield and to reduction using Zn in ammonium chloride to give the final nitroso compound **73** (64% yield; Scheme 5).

**Scheme 6:**
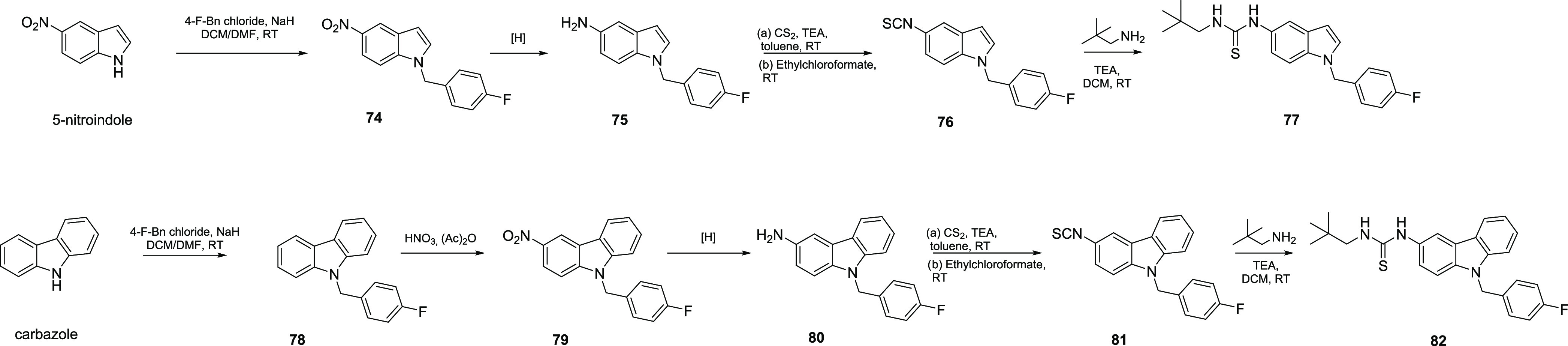
Synthesis of Indole Derivative **77** and Carbazole Derivative **82**

Finally, the procedures used for the synthesis of final compounds **77** and **82** are described in Scheme 6. These molecules were synthesized to expand the structure–activity relationship (SAR) clues about dual 5-LOX/sEH inhibitors by increasing the planarity and aromaticity of the scaffold with the indole ring and allowing further exploration of the binding site by sterically hindered carbazole moiety.

5-Nitroindole was modified at N-1 using 4-fluorobenzyl bromide by the synthetic strategy previously described.^42^ The corresponding intermediate **74** was subjected to the same sequential reaction steps described above to give the final thiourea compound **77** (34% overall yield).

The same reaction procedures were used, starting from 9-(4-fluorobenzyl)-9*H*-carbazole (**78**), which upon nitration in nitric acid and acetic anhydride was transformed into the corresponding thiourea compound **82** (21% overall yield).

### Molecular Docking and Evaluation of 5-LOX and sEH Inhibition

Our in-house library (53 molecules) was in silico-screened and based on visual inspection, energy score, and distance from catalytic iron ion (<4 Å); nine compounds were narrowed as potential inhibitors of 5-LOX. The bicyclic-based molecules, selected for the *in silico* preliminary screening toward 5-LOX, were structurally featured with (*R*)-dihydro-3*H*,5*H*-imidazo[1,5-*c*]thiazole-5,7(6*H*)-dione, 5-methylindolin-2-one, and indoline rings. For the spiro compounds, both enantiomers were considered in the calculations. The docking outcomes suggested that the indoline-based (**43** and **55** and (*R*)-dihydro-3*H*,5*H*-imidazo[1,5-*c*]thiazole-5,7(6*H*)-dione **17** and **18**) scaffolds are suitable molecular seeds for 5-LOX inhibitor design thanks to their ability to properly accommodate into the 5-LOX binding pocket (Table S1). The spiro compounds (**4**, **5**, and **13)** were the less promising scaffolds *in silico* (Table S1). For the sake of consistency, all derivatives were tested for their 5-LOX-inhibitory activity. At first, we evaluated their effectiveness in activated human polymorphonuclear leukocytes (PMNLs), using cell-based assays, which allows the analysis of the interference of the test compounds with 5-LOX in a biological environment. Results obtained were corroborated by testing compounds against the isolated human recombinant 5-LOX, which allows the identification of direct interference of the test compound with the target enzyme. The results are summarized in Table 1. Only derivative **43** reduced 5-LOX product levels in activated human PMNL (IC_50_ 1.38 ± 0.23 μM). In addition, **43** showed remarkable inhibitory activity against isolated 5-LOX (IC_50_ 0.45 ± 0.11).

**Table 1:**
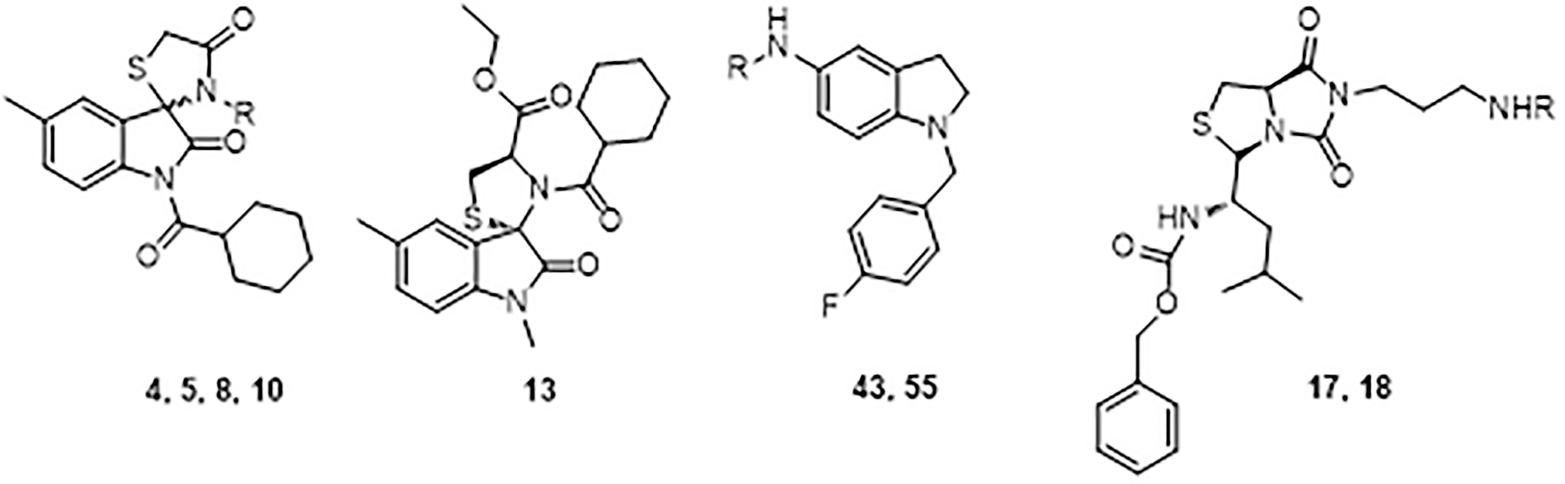
Inhibition of 5-LOX Product Formation in Activated PMNL of Human Isolated 5-LOX^a^^,^^b^

compound	R	IC_50_ (μM) in PMNL	IC_50_ for isolated 5-LOX
**4**	-CH_2_-4-COOH–Ph-	>10	>10
**5**	-(CH_2_)_3_COOH	>10	
**8**	-(CH_2_)_3_NHCOC_6_F_5_	>10	
**10**	-(CH_2_)_3_guanidine	>10	
**13**			
**17**	-(CH_2_)_3_NH_2_	>10	
**18**	-(C-H_2_)_3_guanidine	>10	
**43**	**-CSNHCH_2_C(CH_3_)_3_**	**1.38 ± 0.45**	**0.45 ± 0.11**
**55**	-SO_2_-cyclohexyl	>10	

a The values are given as the mean ± standard error of the mean (SEM) of single determinations obtained in 3–4 independent experiments.

b Zileuton used as a positive control at 3 μM gives residual activities of 3.90 ± 4.14 and 14.24 ± 5.88% over PMNL and isolated 5-LOX, respectively.

**Figure 1:**
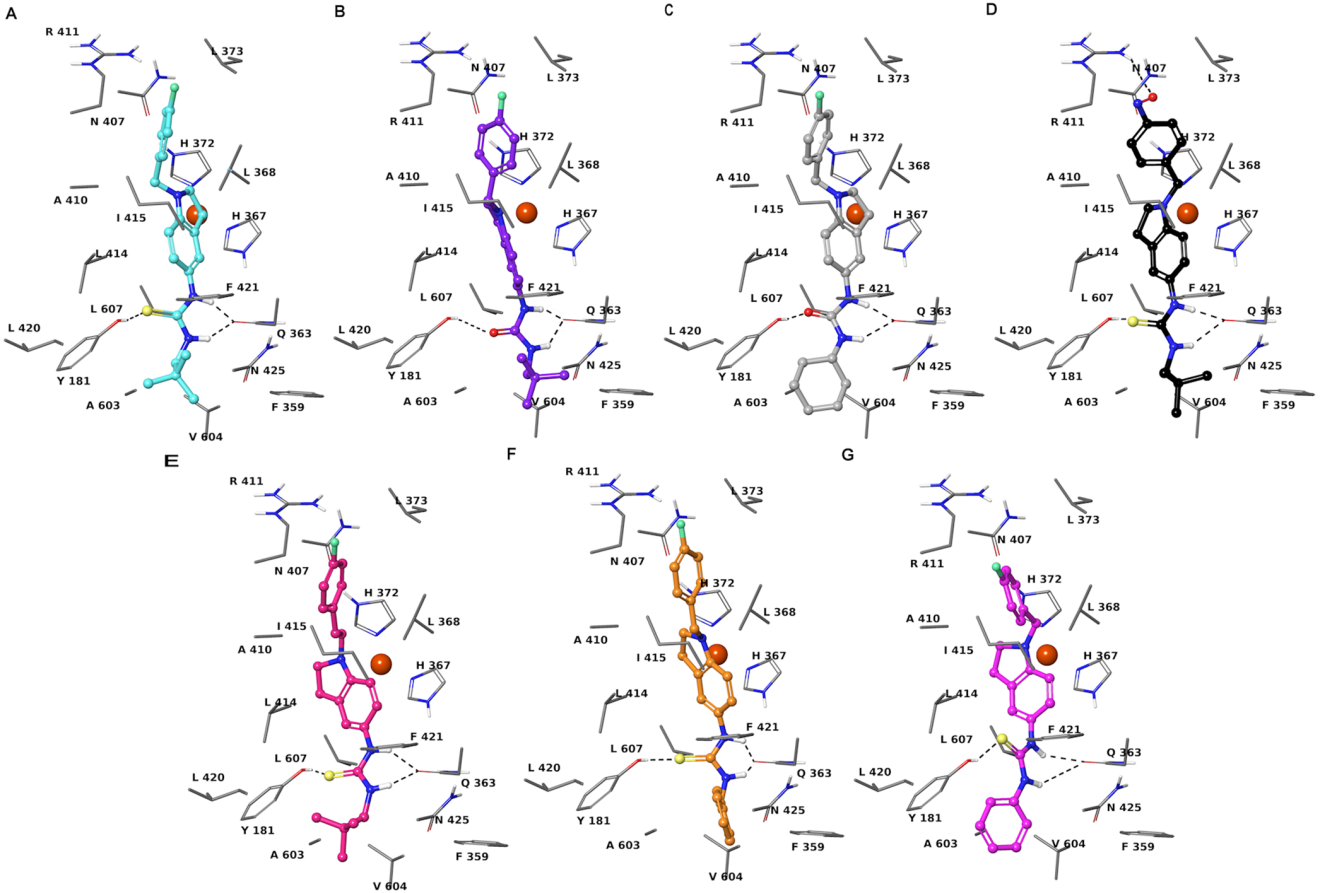
Three-dimensional model of the interactions given by **43** (A), **53** (B), **54** (C), **73** (D), **44** (E), **56** (F), and **57** (G) with 5-LOX. The protein is depicted by tube (colored: C, gray; polar H, white; N, dark blue; O, red). The small molecules are represented by sticks (cyan for **43**, purple for **53**, light gray for **54**, black for **73**, salmon for **44**, orange for **56**, pink for **57**) and balls (colored: C, as for the sticks; polar H, white; N, dark blue; O, red; S, yellow). The dashed black lines indicate the hydrogen bonds between the ligand and protein.

The biological activity of **43** was in agreement with structural observations from molecular docking. In particular, it was observed that the indoline moiety is placed near the iron, hampering the access to the open position of the ion coordination sphere and also contributing to the complex lineup by van der Waals interactions with H372, H367, L368, L414, I415, F421, and L607. The thiourea group of **43** donates two H-bonds to the side chain of Q363 and accepts a hydrogen bond from Y181 (Figure 1A). The neopentyl group of **43** establishes van der Waals contacts with Y181, F421, A424, N425, P569, H600, and A603. The 4-fluorobenzyl moiety is engaged in an aromatic H-bond with the N407 side chain and van der Waals contacts with W147, F151, H372, L368, L373, A410, R411, and I415. Furthermore, the fluorine interacts with the side chain of R411. The remarkable 5-LOX inhibitory activity of **43** together with the observed intermolecular interactions led to the design of 19 structurally correlated compounds.

**Figure 2:**
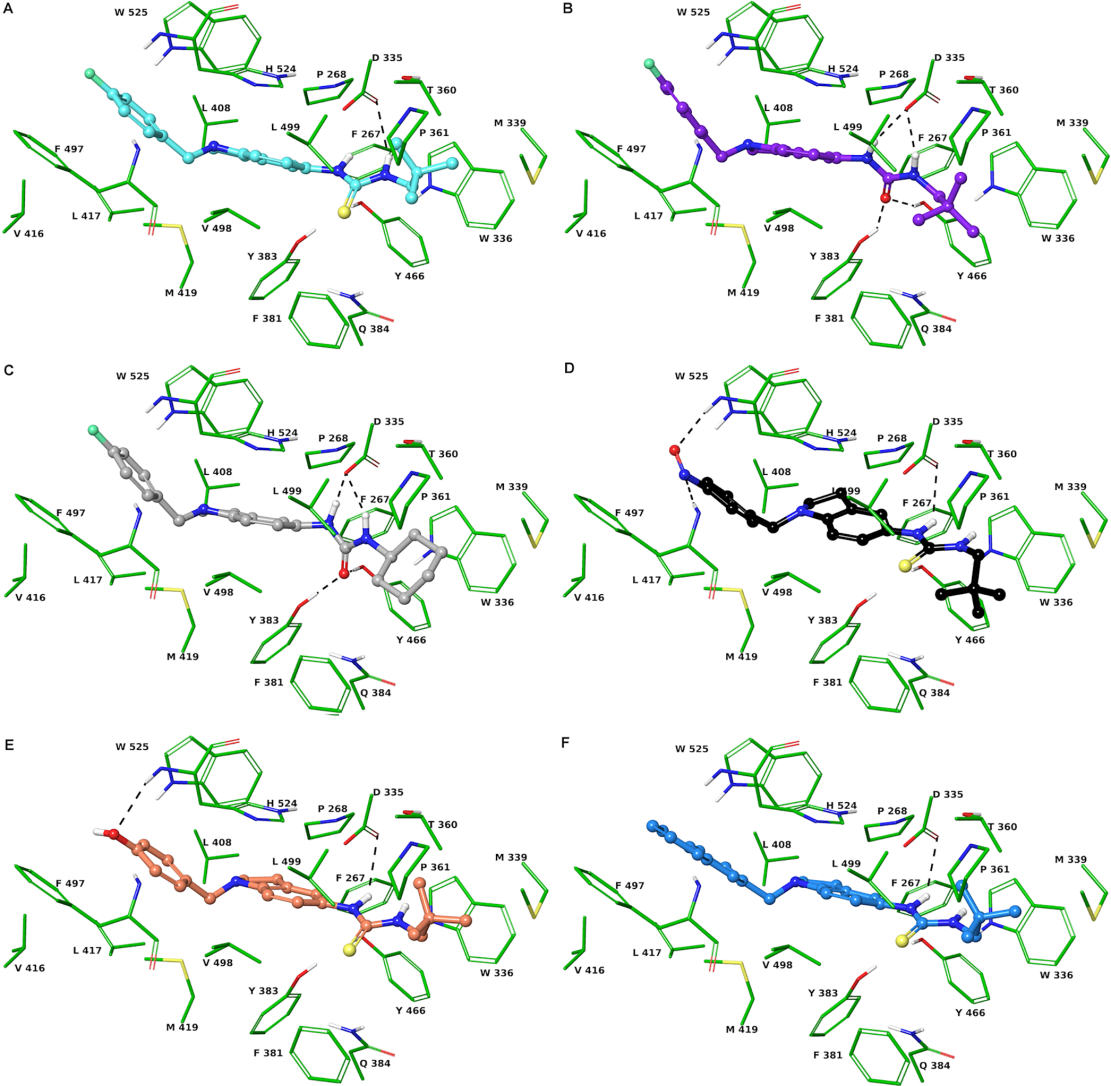
Three-dimensional model of the interactions given by **43** (A), **53** (B), **54** (C), **73** (D), **58** (E), and **46** (F) with sEH. The protein is depicted by tube (colored: C, green; polar H, white; N, dark blue; O, red). The small molecules are represented by sticks (cyan for **43**, purple for **53**, light gray for **54**, black for **73**, faded red-orange for **58**, azure for **46**) and balls (colored: C, as for the sticks; polar H, white; N, dark blue; O, red; S, yellow). The dashed black lines indicate the hydrogen bonds between the ligand and protein.

Specifically, we structurally modified the tert-butyl moiety (**56**–**58**), the thiourea group (**21**, **50**, **53**, **54**), the indoline at C-2 and C-3 (**77**, **82**), and the 4-fluorobenzyl group (**44**–**49**, **51**, **52**, **68**, **69**, **73**) to get clues about structure–activity relationships. All compounds were tested by different *in vitro* assays. Initially, their capability of reducing 5-LOX activity both in activated PMNL and of isolated 5-LOX was challenged. Then, considering the importance of the sEH enzyme in the AA cascade, we questioned whether the parental molecule (compound **43**) and its analogues could affect the activity of this enzyme. We started evaluating **43**/sEH *in silico* interaction, observing a good fit into the binding cavity of sEH by compound **43** (Figure 2A). The docked pose of **43** shown gives π–π interactions with H524 and W525 by indoline and 4-fluorobenzyl moieties, respectively. Its urea group donates a hydrogen bond to D335, whereas the neopentyl moiety gives van der Waals contacts with W336, M339, Q384, Y466, and L499.

The docking hypothesis was confirmed by *in vitro* results that revealed an IC_50_ of 1.39 ± 0.45 μM against the human isolated sEH (Table 2).

**Table 2:**
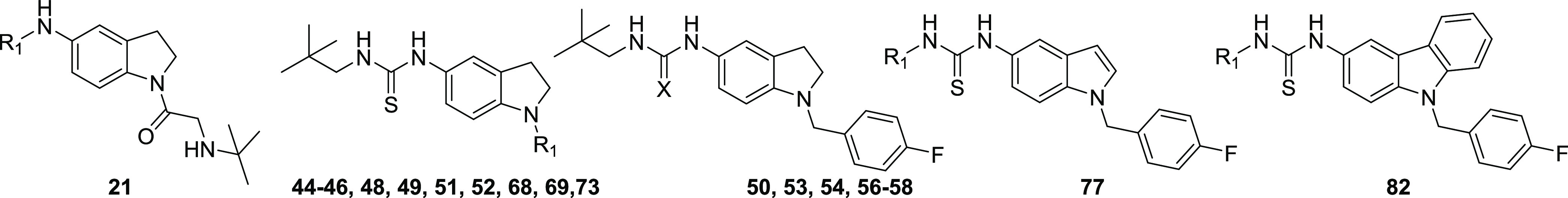
Inhibition of 5-LOX Product Formation in Activated PMNL of Human Isolated 5-LOX and Human Isolated sEH^a^^,^^b^

compound	R_1_	X	IC_50_ in PMNL (μM)	IC_50_ for isolated 5-LOX (μM)	IC_50_ for isolated sEH (μM)
**43**	4-F-PhCH_2_-		1.38 ± 0.23	0.45 ± 0.11	1.39 ± 0.45
**21**	4-F-PhCH_2_-		>10	>10	>10
**44**	4-F-PhCH_2_CH_2_-		4.87 ± 0.41	0.38 ± 0.05	1.14 ± 0.29
**45**	4-Ph-PhCH_2_-		>10	1.48 ± 0.48	>10
**46**	β-NaphthylCH_2_-		>10	1.02 ± 0.44	0.91 ± 0.24
**48**	HO(CH_2_)_3_CH_2_-		nd	9.02 ± 4.20	13.86 ± 1.60
**49**	4-CH_3_OCH_2_O-PhCH_2_-		2.93 ± 0.70	>10	2.40 ± 0.80
**50**	(CH_3_)_3_CCH_2_-	NH	>10	1.42 ± 0.23	>10
**51**	H_2_N(CH_2_)_3_CH_2_-		>10	>10	>10
**52**	4-HO-PhCH_2_-		2.90 ± 0.75	5.10 ± 2.92	0.79 ± 0.52
**53**	Cyclohexyl-	O	>10	0.28 ± 0.02	0.061 ± 0.003
**54**	(CH_3_)_3_CCH_2_-	O	nd	0.18 ± 0.05	0.10 ± 0.01
**56**	PhCH_2_-	S	0.95 ± 0.15	0.57 ± 0.12	3.86 ± 0.79
**57**	Ph-	S	1.98 ± 0.32	0.16 ± 0.05	10.39 ± 0.37
**58**	4-COOH-Ph-	S	>10	2.29 ± 0.46	>10
**68**	4-F-PhCO-		>10	>10	>10
**69**	4-F-PhCH_2_CO-		>10	>10	>10
**73**	4-ONPhCH_2_-		0.59 ± 0.09	0.41 ± 0.01	0.43 ± 0.10
**77**	(CH_3_)_3_CCH_2_-		>10	>10	2.12 ± 1.06
**82**	(CH_3_)_3_CCH_2_-		>10	>10	>10

a All values are given as the mean ± SEM of single determinations obtained in 3–4 independent experiments.

b Zileuton used as a positive control at 3 μM gives residual activities of 3.90 ± 4.14 and 14.24 ± 5.88% over PMNL and isolated 5-LOX, respectively. AUDA used as a positive control at 1 μM gives a residual activity of 3.90 ± 4.14% over isolated sEH.

c nd, not determined.

Considering this data, all of the indoline compounds were tested for their inhibitory activity on isolated sEH. Results obtained are summarized in Table 2.

Compounds **53**, **54**, and **73** exhibit the best experimental outcomes, filling equivalent spaces at the 5-LOX binding site when compared to **43**, and display the same pattern of intermolecular contacts by the common structural moieties (Figure 1). In contrast to the parent compound **43**, in **53** and **54** the thiourea is substituted with urea that preserves the same network of H-bonds in the 5-LOX binding pocket. Like the neopentyl group of **43**, the cyclohexyl substituent of **53** gives van der Waals contacts with Y181, F421, A424, N425, P569, H600, and A603. Compounds **53** and **54** inhibited the isolated 5-LOX enzyme potently, with an IC_50_ of 0.28 ± 0.02 and 0.18 ± 0.05 μM, respectively (Table 2). With respect to **43**, both compounds showed no 5-LOX inhibitory activity in the cell-based assay. This might be explained by poor membrane permeation, but different reasons cannot be excluded. Moreover, **53** and **54** also showed a potent inhibitory effect against sEH, with an IC_50_ of 61 ± 3 and 100 ± 10 nM, respectively (Table 2). This data is in accordance with the literature suggesting that the presence of an urea group is a pivotal requisite for potent sEH inhibition.^43^ Indeed, the two compounds fitted very well in the binding cavity of the enzyme, presenting superimposable conformations with the cocrystallized ligand 34N (Figure 2B,C).^44^ For both binders, the indoline and 4-fluorobenzyl moieties give π–π interaction with H524 and W525, respectively. Their urea group is involved in a network of four hydrogen bonds with the side chains of D335, Y383, and Y466, unlike **43** that donates a hydrogen bond with its thiourea group (Figure 2A). The neopentyl (**54**) and cyclohexyl (**53**) groups are involved in van der Waals contacts with Y336, M339, Q384, Y466, and Leu499. It is noteworthy that the contacts found for the indoline, urea, and neopentyl/cyclohexyl are also observed with 34N.^44^

In **50**, the thiourea was replaced by a guanidine, losing the hydrogen bond with the Y181 side chain. The loss of interaction with Y181 seems responsible for a partial loss of activity for compound **50** showing three times higher IC_50_ on isolated 5-LOX compared to **43** (1.42 ± 0.23 *vs* 0.45 ± 0.11 μM).

The most interesting compound of the series is represented by compound **73**. It is noteworthy that **73** structurally differs from all other compounds for the NO group instead of fluorine (Figure 1D). The NO group is engaged in hydrogen bonds with side chains of R411, tightening the affinity toward 5-LOX. The phenyl ring in the two aromatic H-bonds with the backbone CO of L368 and N407 shows the highest potency against both isolated 5-LOX and PMNL (0.41 ± 0.01 and 0.59 ± 0.09 μM, respectively; Figure 2D and Table 2). Moreover, **73** also exhibited an important efficacy against sEH with an IC_50_ of 0.43 μM ± 0.10 (Table 2). We observed that the NO group also favors the binding against sEH by establishing two H-bonds with the backbone NH group of F497 and H524.

With respect to **73**, the phenol moiety of **52** is only H-bonded to the backbone CO of N407, justifying a reduced activity on the isolated 5-LOX and PMNL (IC_50_ = 5.10 ± 2.92 and 2.90 ± 0.75 μM, respectively); otherwise, sEH inhibition is maintained with an IC_50_ of 0.79 ± 0.52 μM (Table 2). In fact, even though the NO and OH groups of **73** and **52** are also hydrogen-bonded to the backbone NH and CO of H524 and V416 in the sEH binding cavity, their presence displaces the thiourea moiety, compared to urea of **53** and **54**, giving rise to only one H-bond (Figure 2), justifying the mild reduction of sEH activity by **53** and **54**.

If compared with **43**, compounds **44**–**49** and **51** present a substantial modification of the substituent at indoline nitrogen that could affect the correct binding into the 5-LOX catalytic site. Specifically, the switch from methylene (**43**) to ethyl linker between 4-fluorophenyl and indoline moieties (**44**) is well tolerated, while a further increase of the substituent size (**45**, **46**, and **49**) impairs the interaction from the remaining structural portions of the small molecules, especially for **49**. Compound **48** showed a superimposable accommodation of common molecular portions with the parent compound in the 5-LOX binding site, but the hydroxybutyl chain is quite folded although H-bonded to the backbone CO of Q363. All of these compounds exhibit a micromolar activity against the isolated 5-LOX, except **44**, which showed an IC_50_ comparable to **43** (0.38 ± 0.05 *vs* 0.45 ± 0.11 μM). However, for derivatives **45** and **48**, a decrease of effectiveness against sEH was observed, while their analogues **44**, **46**, and **49** act on the same target with an IC_50_ in the low micromolar range. Unfortunately, none of them are effective against 5-LOX in PMNL. Compound **49** binding to sEH could be rationalized using the same considerations made for **53** and **54**. The compound accepts a H-bond from the backbone NH of H524, leading to an unfavorable entropic loss (Figure 2). Like **52** and **73**, increasing the size of the substituent at indoline nitrogen (**44** and **46**) also allows hydrogen bonding with only the side chain of D335 (Figure 2). Derivative **45**, endowed with a larger substituent at indoline nitrogen than **44** and **46**, is unable to give hydrogen bonds by the thiourea moiety (Figure 2).

The conversion of the indoline core to indole (**77**) induces a binding conformation in 5-LOX characterized by less contacts with W147, F151, H372, L368, L373, A410, R411, and I415, reflecting an almost complete loss of 5-LOX and sEH inhibition. In **82**, the indoline was converted into the more hindered carbazole, resulting in docked poses with distorted structural moieties, especially for thiourea and tricyclic aromatic portion, inducing a total inactivity against both targets. For **56–58**, a π–π interaction is observed among the phenyl ring and side chain of F421, unlike the parent compound. Moreover, **56** gives a π–π interaction with F359, while **57** and **58** give a π–π interaction with Y181. The compounds maintain good activity in the cell-free assay, but only **56** and **57** are able to affect the 5-LOX activity in PMNL, likely due to their higher lipophilicity than the acid analogue **58**. In consideration of the data described, taking particularly into account the activity of the synthesized derivatives on 5-LOX in PMNL, more properly resembling the biological environment, we have decided to select compound **73** for further pharmacological characterization of this class of compounds.

### Evaluation of COX-1 and COX-2 Inhibition in Intact Cells

We next investigated the impact of **73** on COX-1 and COX-2, enzymes within the AA cascade that are involved in the biosynthesis of prostanoids in addition to 5-LOX and sEH. A well-established *in vitro* cell culture assay (J774 murine macrophages) was performed to evaluate the effects of **73** against both COX isoforms.^45−47^ Stimulation of J774 macrophages with AA (15 μM) for 30 min induced a significant increase of PGE_2_ levels in comparison to unstimulated control cells. **73** weakly inhibited the production of PGE_2_ primarily generated via COX-1 at micromolar concentrations (Figure S76A). The same trend was observed for PGE_2_ production in LPS-stimulated cells in the absence (Figure S76B) or presence (Figure S76C) of AA, which is mainly mediated by inducible COX-2. Indomethacin and celecoxib as reference agents were active as expected. In addition, **73** did not affect COX-2 expression induced by the stimulation of cells with LPS (10 μg/mL) (Figure S76D). These results indicate a higher target selectivity of compound **73** for 5-LOX and sEH enzymes.

Finally, cytotoxic effects were excluded since **73** did not impair cell viability at all tested concentrations (Figure S76E). Overall, molecular docking and *in vitro* biological investigations suggest **73** as a promising drug candidate for further *in vivo* pharmacological studies.

### Evaluation of *In Vivo* Anti-Inflammatory Effects

#### Compound **73** Reduces Inflammation in Zymosan-Induced Peritonitis

**Figure 3:**
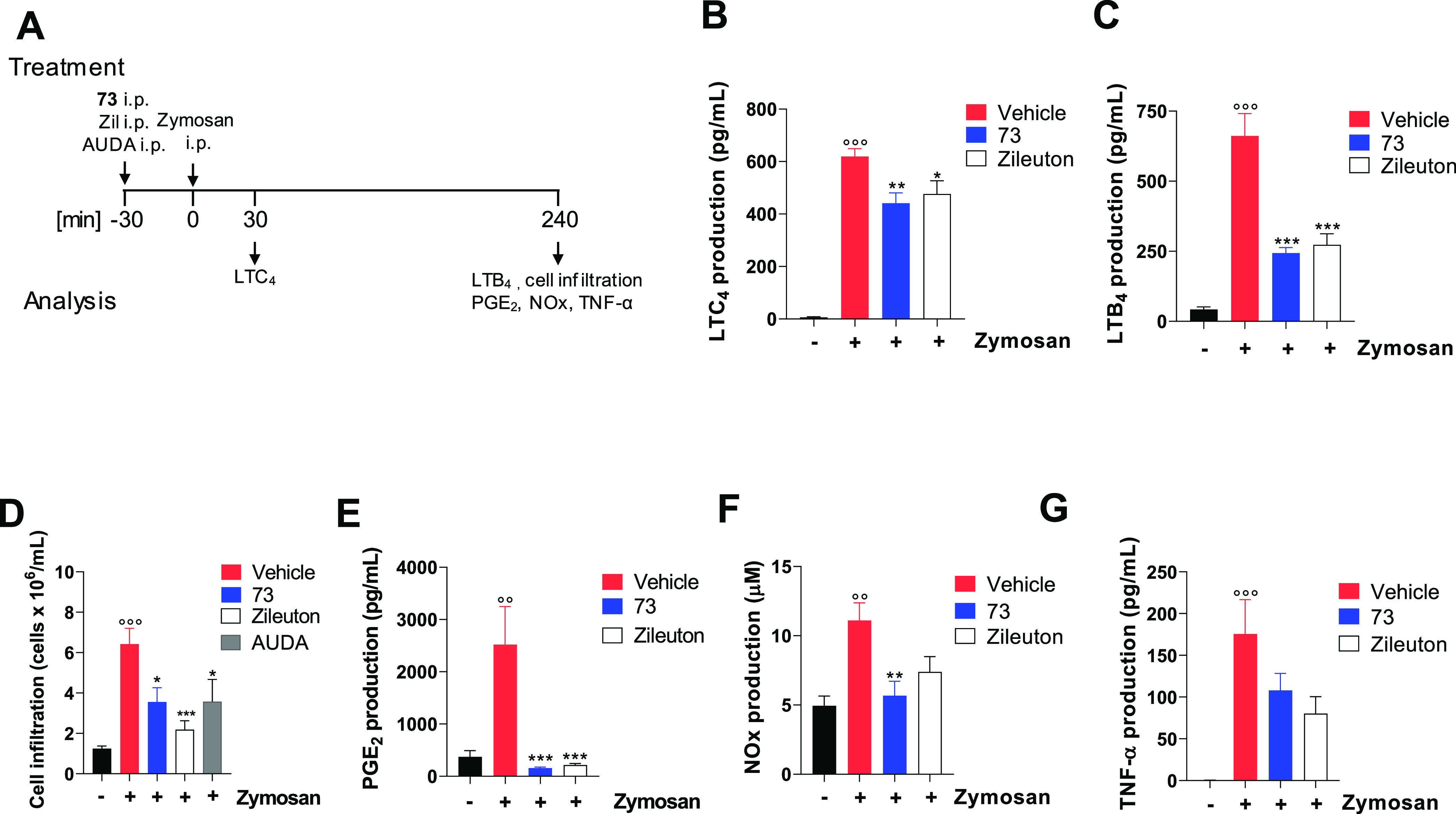
Compound **73** inhibits 5-LOX product formation and limits inflammation in murine peritonitis. (A) Time scale for zymosan-induced murine peritonitis. Mice received **73** (10 mg/kg, i.p.), zileuton, or AUDA (10 mg/kg, i.p.) 30 min before zymosan and were sacrificed 30 min (B) or 4 h (C–G) post peritonitis induction injection. (B) LTC_4_, (C) LTB_4_, (E) PGE_2_, and TNF-α (G) levels in the exudate analyzed by enzyme-linked immunosorbent assay (ELISA). (D) Immune cell infiltration into the peritoneal cavity. (F) NOx levels in the exudates by Griess assay. Values represent means ± SEM; *n* = 6 mice for each group. Data were analyzed by one-way analysis of variance (ANOVA) and Bonferroni. Statistical significance is reported as follows: °° *P* < 0.01 and °°° *P* < 0.001 vs control; * *P* < 0.05; ** *P* < 0.01; and *** *P* < 0.001 vs zym + vehicle.

The anti-inflammatory efficacy of **73** was evaluated *in vivo* in zymosan-induced mouse peritonitis, an experimental model of acute inflammation related to LTs and other lipid mediators.^48,49^ Zileuton and AUDA were used as controls (i.p. 10 mg/kg, 30 min before zymosan; Figure 3A). During the onset of inflammation, zymosan activates resident murine peritoneal macrophages that produce LTC_4_. The progressive phase of inflammation is instead dominated by infiltrated neutrophils, which generate the potent chemoattractant LTB_4_ and other proinflammatory mediators such as PGE_2_, nitric oxide, and TNF-α. Accordingly, 30 min and 4 h after zymosan injection, a significant increase of LTC_4_ and LTB_4_ was observed as compared to the unstimulated control group (Figure 3B,C). The i.p. pretreatment of mice with **73** (10 mg/kg, 30 min before zymosan; Figure 3A) significantly reduced LTC_4_ and LTB_4_ levels in the peritoneal exudate, comparable to zileuton (Figure 3B,C). Since LTB_4_ is a major chemoattractant for leukocytes, **73** caused a concomitant reduction of leukocyte recruitment in the peritoneal cavity (Figure 3D). Surprisingly, in compound **73**, in pretreated animals, a strong reduction of PGE_2_ levels was observed in comparison to vehicle-treated mice (Figure 3E), apparently in contrast with *in vitro* data (Figure 76S). Nevertheless, a closer look at the *in vitro* data reveals that compound **73** is able to exert a small but significant reduction of PGE_2_ at the highest concentration used (10 μM; Figure 76S). This is why the high local concentration reached in the peritoneum immediately after intraperitoneal administration could be accounted for the inhibitory effect over PGE_2_ levels. In addition, compound **73** also showed *invivo* anti-inflammatory effects by the inhibition of zymosan-induced NOx (Figure 3F) and TNF-α (Figure 3G) in the peritoneal exudates of zymosan-treated mice.

**Figure 4:**
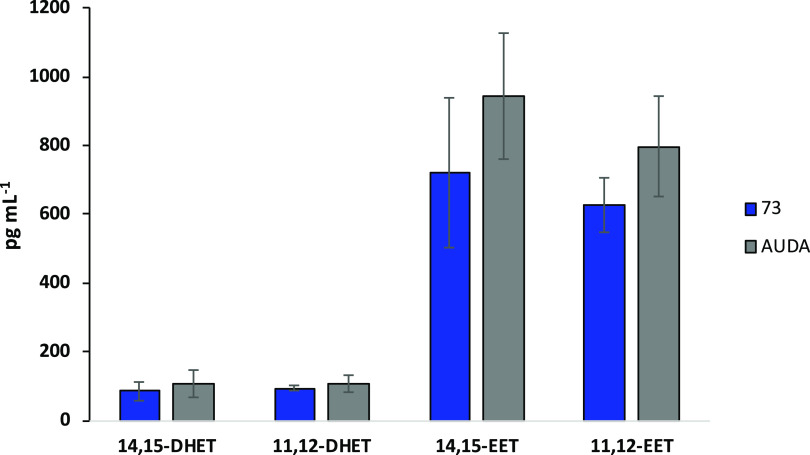
Effect of compound **73** (10 mg/kg, i.p.) and AUDA (10 mg/kg, i.p.) administration on eicosatrienoic acid levels in mouse peritoneal exudate during acute inflammation initiated by zymosan. Values represent means ± S.D.; *n* = 6 mice for each group.

Finally, to confirm in vivo sEH inhibition, we measured the levels of several epoxy- and dihydroxy-unsaturated fatty acids (Figures 4 and S77 and Tables S4–S6) in peritoneal exudate upon compound **73** administration, using AUDA as a positive control. In detail, we were unable to measure the levels of (±)5,6-epoxy-8*Z*,11*Z*,14*Z*-eicosatrienoic acid (5,6-EET), (±)8,9-epoxy-5*Z*,11*Z*,14*Z*-eicosatrienoic acid (8,9-EET), and the corresponding dihydroxy derivatives (±)5,6-dihydroxy-8*Z*,11*Z*,14*Z*-eicosatrienoic acid (5,6-DHET) and (±)8,9-dihydroxy-5*Z*,11*Z*,14*Z*-eicosatrienoic acid (8,9-DHET) since their amount was below the LOQ. Thus, quantification was performed only for (±)11,(12)-epoxy-5*Z*,8*Z*,14*Z*-eicosatrienoic acid (11,12-EET), (±)14(15)-epoxy-5*Z*,8*Z*,11*Z*-eicosatrienoic acid (14,15-EET), (±)11,12-dihydroxy-5*Z*,8*Z*,14*Z*-eicosatrienoic acid (11,12-DHET), and (±)14,15-dihydroxy-5*Z*,8*Z*,11*Z*-eicosatrienoic acid (14,15-DHET). We found that the epoxy-unsaturated fatty acid levels were about 8 times higher than the corresponding dihydroxy-unsaturated fatty acids in both **73**- and AUDA-treated mice, in accordance with the sEH inhibition mechanism (Figure 4).

Since compound **73** contains a nitrosobenzene and a dihydroindole moiety, the overall stability of the molecule can be questioned. This is why we decided to challenge the compound stability in mouse plasma. To this aim, blood samples were collected from animals at predetermined intervals (0.5, 1, 2, and 4 h) and, upon extraction, were analyzed by high-performance liquid chromatography/mass spectrometry (HPLC/MS) to quantify compound **73**. Results obtained (Table S3) show substantial stability of compound **73**, with a plasma half-life in mice of 1.6 h.

#### Compound **73** Relieves Hallmarks of Asthma and LT Pulmonary Levels

**Figure 5:**
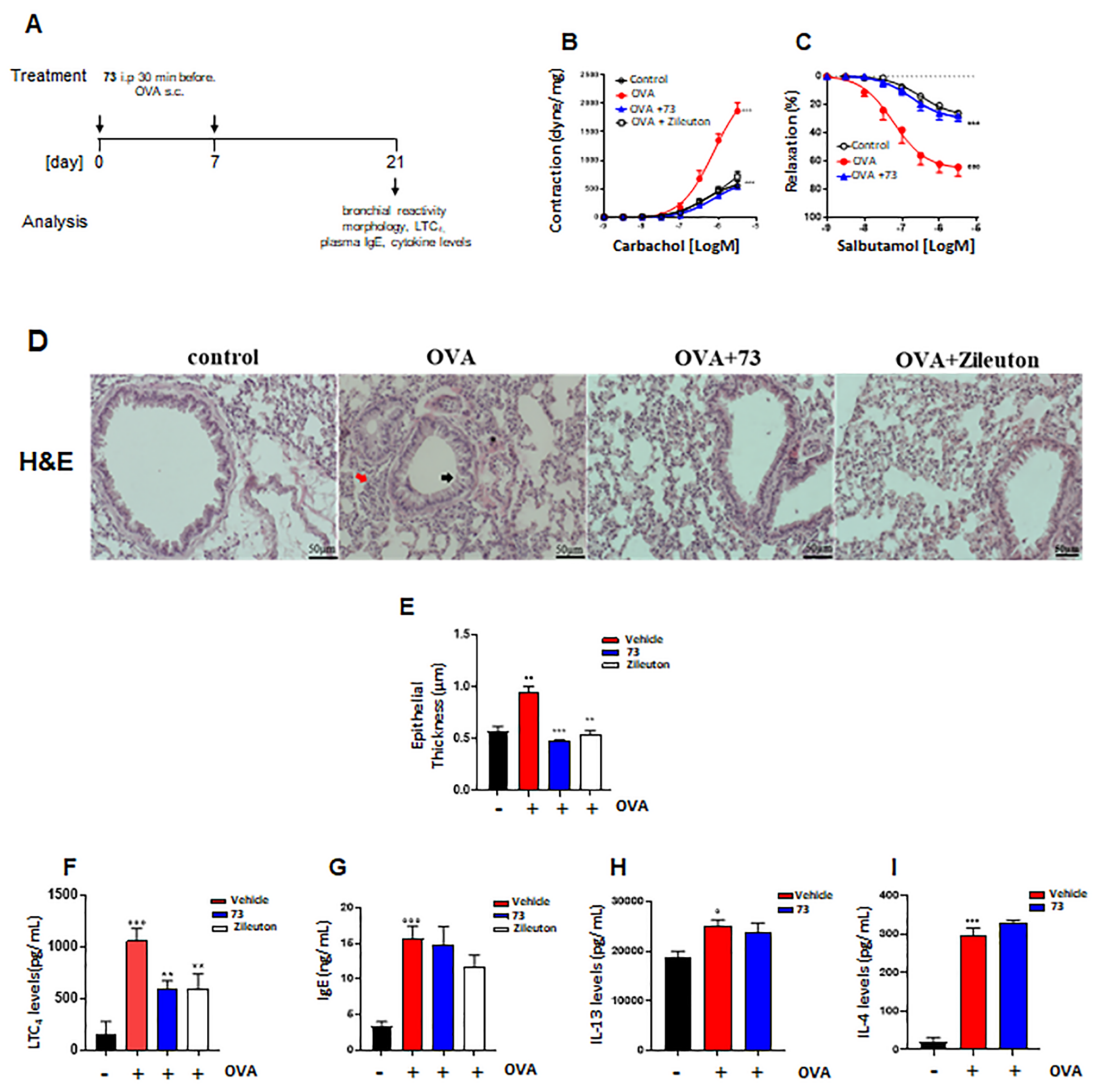
Compound **73** suppresses hallmarks of asthma and pulmonary LT formation in mice sensitized to ovalbumin. (A) Time scale for the experimental asthma model. Compound **73** (10 mg/kg) and zileuton (35 mg/kg) were i.p. administered to mice 30 min prior to injection of ovalbumin (OVA) at days 0 and 7. (B) Bronchial reactivity to carbachol or (C) salbutamol. (D) Lung slices were stained for H&E. (E) Epithelial thickness was evaluated using ImageJ Fiji. Pulmonary levels of (F) LTC_4_, (H) IL-13, (I) IL-4, and (G) plasma IgE levels were analyzed by ELISA. Values represent means ± SEM; *n* = 6 mice for each group. Data were analyzed by two-way ANOVA plus Bonferroni (B and C) and one-way ANOVA plus Bonferroni (E–I). Statistical significance is reported as follows: ° *P* < 0.05; °° *P* < 0.01; and °°° *P* < 0.001 vs control; ** *P* < 0.01 and *** *P* < 0.001 vs OVA + vehicle. Black arrow, bronchial epithelium thickness; red arrow, pulmonary cell infiltration in peribronchial areas; and asterisk; pulmonary cell infiltration in perivascular areas (D).

Since LTs play a pivotal role in the pathogenesis of asthma by inducing immune cell infiltration, pulmonary inflammation, and bronchoconstriction,^15^ we investigated the effects of **73** in an experimental model of asthma. Mice were pretreated with **73** i.p. 30 min before ovalbumin (OVA) injection on days 0 and 7. Animals were sacrificed after 21 days to evaluate bronchial hyper-reactivity, pulmonary inflammation, pulmonary LTC_4_ levels, plasma IgE, and Th2 cytokine production (Figure 5A). OVA sensitization induced airway hyper-reactivity to carbachol (Figure 5B), and increased bronchial relaxation in response to salbutamol was observed (Figure 5C). Intraperitoneal treatment of mice with **73** reversed OVA-induced bronchial hyper-reactivity to carbachol (Figure 5B) and fully restored the adrenergic bronchial relaxation induced by salbutamol (Figure 5C). OVA sensitization caused airway inflammation by inducing a morphological alteration (Figure 5D) and increasing the bronchial epithelium thickness (black arrow) (Figure 5D,E). Further, OVA sensitization promoted pulmonary cell infiltration in peribronchial (red arrow) and perivascular areas (asterisk) compared to the control group (Figure 5D). Pretreatment with **73** significantly reduced the epithelial thickness (Figure 5E) in OVA-sensitized mice. The beneficial effect of **73** on lung function was associated with the reduction of pulmonary LTC_4_ levels in sensitized mice treated with **73** (Figure 5F). However, **73** did not affect sensitization mechanisms. Indeed, **73** did not modulate plasma IgE levels (Figure 5G) and pulmonary T-helper type 2 cytokines such as interleukin-13 and interleukin-4 (Figure 5H,I) in OVA-sensitized mice. In the same setting of experiments, the effects of **73** were compared to those of zileuton (Figure 5). The data obtained provide similar efficacy of the two molecules; however, the required dose of zileuton is 35 mg/kg,^50^ while 10 mg/Kg of compound **73** generate a pharmacological response. The improved efficacy in terms of pharmacological activity reflects the synergic effect obtained by interfering with the two enzymes than a single target.

## Conclusions

Inflammatory diseases represent one of the most common illnesses worldwide; in particular, among these, asthma affects more than a hundred million people. Many therapies are commonly used to treat this type of airway inflammation, principally steroidal anti-inflammatory and adrenergic or anticholinergic drugs. These medications act with different mechanisms: blocking AA hydrolysis from membrane phospholipids, reducing the immune system response, relaxing the bronchial smooth muscle, etc. However, very few approved drugs can counteract the biosynthesis or the signaling mediated by LTs, the principal eicosanoids involved in asthma. In this frame, multitarget inhibitors, intervening in more than one step of the AA metabolism, represent an interesting therapeutic option. In the present paper, we have described several molecules characterized by 5-LOX/sEH dual inhibitory properties. The *in vitro* results were rationalized by *in silico* investigation highlighting several pivotal drug/target interactions that could drive the future design of 5-LOX and sEH single or, particularly, dual inhibitors. In fact, the effectiveness of 5-LOX/sEH dual inhibition approach has also been confirmed *in vivo* when compound **73** was challenged in two different murine models: zymosan-induced peritonitis and ovalbumin-induced asthma. Indeed, compound **73** displays *in vivo* anti-inflammatory effects in decreasing the LT levels as well as cell infiltration and the levels of proinflammatory mediators in the peritonitis model. Moreover, **73** reversed the OVA-induced airway inflammatory response by decreasing LTC_4_ levels. Globally, this evidence suggests that compound **73** can be taken into consideration for further development as a therapeutic tool in inflammatory diseases.

## Experimental Section

### General

(±)5,6-Epoxy-8*Z*,11*Z*,14*Z*-eicosatrienoic acid (5,6-EET), (±)8,9-epoxy-5*Z*,11*Z*,14*Z*-eicosatrienoic acid (8,9-EET), (±)11,(12)-epoxy-5*Z*,8*Z*,14*Z*-eicosatrienoic acid (11,12-EET), (±)14(15)-epoxy-5*Z*,8*Z*,11*Z*-eicosatrienoic acid (14,15-EET), (±)14(15)-epoxy-5*Z*,8*Z*,11*Z*-eicosatrienoic-16,16,17,17,18,18,19,19,20,20,20-d11 acid (14,15-EET-d11), (±)5,6-dihydroxy-8*Z*,11*Z*,14*Z*-eicosatrienoic acid (5,6-DHET), (±)8,9-dihydroxy-5*Z*,11*Z*,14*Z*-eicosatrienoic acid (8,9-DHET), (±)11,12-dihydroxy-5*Z*,8*Z*,14*Z*-eicosatrienoic acid (11,12-DHET), (±)14,15-dihydroxy-5*Z*,8*Z*,11*Z*-eicosatrienoic acid (14,15-DHET), and (±)11,12-dihydroxy-5*Z*,11*Z*,14*Z*-eicosatrienoic-16,16,17,17,18,18,19,19,20,20,20-d11 acid (11,12-DHET-d11) were purchased from Cayman Chemical (Michigan). All of the other reagents and solvents were purchased from Merck (Milan, Italy). Reactions were performed under magnetic stirring in round-bottomed flasks unless otherwise noted. Moisture-sensitive reactions were conducted in oven-dried glassware under a nitrogen stream, using freshly distilled solvents. Thin-layer chromatography (TLC) analysis of reaction mixtures was performed on precoated glass silica gel plates (F254, 0.25 mm, VWR International), while crude products were purified by the Isolera Spektra One automated flash chromatography system (Biotage, Uppsala, Sweden), using commercial silica gel cartridges (SNAP KP-Sil, Biotage). NMR spectra were recorded on a Bruker Avance 400 MHz apparatus at room temperature. Chemical shifts were reported in δ values (ppm) relative to internal Me_4_Si for ^1^H and ^13^C NMR. *J* values were reported in hertz (Hz). ^1^H NMR peaks were described using the following abbreviations: s (singlet), d (doublet), t (triplet), and m (multiplet). High-resolution mass spectrometry (HR-MS) spectra were recorded by an LTQ-Orbitrap-XL-ETD mass spectrometer (Thermo Scientific, Bremen, Germany), equipped with an ESI source. All of the final compounds showed a purity ≥95% as assessed by RP-UHPLC-PDA analysis, performed using a Nexera UHPLC system (Shimadzu, Kyoto, Japan) consisting of a CBM-40 lite controller, two LC-40B X3 pumps, an SPD-M 40 photodiode array detector, a CTO-30A column oven, and an SIL-40C X3 autosampler. The chromatographic analysis was accomplished on a Kinetex Evo C18 column, 150 mm × 2.1 mm × 2.6 μm (Phenomenex, Bologna, Italy), maintained at 40 °C. The optimal mobile phase consisted of 0.1% HCOOH/H_2_O v/v (A) and 0.1% HCOOH/ACN v/v (B) delivered at a constant flow rate of 0.3 mL/min^–1^. Analysis was performed in gradient elution as follows: 0–20.00 min, 5–95% B; 20.00–25.00 min, isocratic to 95% B; then 5 min for column re-equilibration. Data acquisition was set in the range of 190–800 nm, and chromatograms were monitored at 254 nm.

#### General Procedure A: Thiazolidinone Synthesis (**1–3**)

5-Methylisatin (1.0 mmol) was dissolved in ethanol (50 mL), and the solution was warmed at 100 °C. Mercaptoacetic acid (1.5 mmol) and 4-(aminomethyl)benzoic acid, or 4-aminobutanoic acid, or *N*-Boc-1,3-propanediamine (0.5 mmol) were added, and the mixture was stirred for 120 min. Then, 5.0 mL of a solution of NaHCO_3_ (10% v-v) was added and the organic phase was evaporated *in vacuo*. The crude was dissolved in dichloromethane, and a basic aqueous solution (Na_2_CO_3_ 2 N) was employed to wash the organic phase (3 × 100 mL). The dichloromethane layer was then dried on Na_2_SO_4_, filtered, and evaporated under *vacuo*. Flash chromatography on silica gel using different eluent systems yielded intermediates **1–3**.

#### General Procedure B: *N*-Acylation (**4–6, 8, 13, 59–61**)

**1–3** or **7** or **12** (0.5 mmol), or 5-nitroindoline, was dissolved in dichloromethane, and the proper commercially available acyl chloride or di-*tert*-butyl dicarbonate (0.6 mmol) and triethylamine (0.6 mmol) were added. The mixture was stirred at room temperature for 30 min. Subsequently, water was added and the organic phase was washed three times, dried on Na_2_SO_4_, filtered, and evaporated. Flash chromatography on silica gel was performed to purify N-acyl compounds, using a proper eluent system.

#### General Procedure C: *N*-Alkylation (**11, 74, 78**)

5-Methylisatin or 5-nitroindole (0.15 mmol) was dissolved in dimethylformamide (DMF) under magnetic stirring, and the temperature was set to 0 °C. To this solution, 0.23 mmol of NaH were added portionwise and the mixture was allowed to react for 30 min. Then, 0.23 mmol of methyl iodide or 4-fluorobenzyl chloride in DMF were added dropwise and the reaction was warmed to room temperature and maintained under stirring for a further 12 h. Then, the reaction was quenched with 10% aqueous solution of citric acid and washed with brine. The organic layer was separated, dried over anhydrous Na_2_SO_4_, filtered, and evaporated *in vacuo*. The crude product was purified by flash chromatography using *n*-hexane/ethyl acetate (4:1 v-v) as the mobile phase to obtain intermediates **11**, **74**, and **78**.

#### General Procedure D: Boc Removal (**7, 10, 17, 18, 51, 71**)

The *N*-Boc-protected intermediates (0.2 mmol) were dissolved in a mixture of TFA/DCM (1/3, v/v), and triisopropylsilane (TIS, 0.05 mmol) was added. The reaction was stirred at room temperature for 2 h. Then, a solution of NaOH (2 N) was added until pH 7. The mixture was diluted with water and dichloromethane, and the organic phase was extracted, dried over Na_2_SO_4_, filtered, and concentrated under vacuum. The intermediates obtained were not further purified.

#### General Procedure E: Guanidine Group Introduction (**9, 18**)

Compounds **7** or **17** (0.5 mmol) were dissolved in DCM, and *N*-Boc-1*H*-pyrazole-1-carboxamidine (0.6 mmol), triethylamine (0.5 mmol) and *N*,*N*-dimethylaminopyridine (0.35 mmol) were added and the reaction was warmed at 65 °C for 4 h. Then, the mixture was cooled and washed with a NaHCO_3_ aqueous solution (10% v-v) three times. Chromatographic purification on silica gel using different eluents gave guanidine compounds **9** and **18**.

#### General Procedure F: Urea Formation (**19, 53, 54**)

Aminic compounds (5-NO_2_-indoline or **29**, 0.1 mmol) were dissolved in dichloromethane, and triphosgene (0.025 mmol) and triethylamine (0.12 mmol) were added. The mixture was reacted for 30 min, and the second amine (2,2-dimethylpropan-1-amine or cyclohexylamine) was introduced and the reaction was stirred for 1 h at room temperature. Then, the organic solvent was treated with water (3 × 100 mL) and the organic phase was dried over Na_2_SO_4_, filtered, and evaporated. Ureidic compounds were isolated after flash chromatography using different ratios of *n*-hexane/ethyl acetate as the mobile phase.

#### General Procedure G: Reductive Amination (**21, 22–28, 72**)

5-NO_2_-indoline derivative (0.1 mmol) was dissolved in a solution of DCM/CH_3_COOH (5:1 v/v) at room temperature. To this solution, an amount of 0.2 mmol of proper aldehyde were added and the mixture was warmed to reflux for 1.5 h. Then, an amount of 0.18 mmol of sodium triacetoxyborohydride were added portionwise and the mixture was allowed to reflux for a further 3–5 h. After cooling to room temperature, NaOH(1N) was added. The organic phase was separated and extracted one more time with the alkaline solution. Then, it was dried over Na_2_SO_4_, filtered, and concentrated *in vacuo*. The crude products were purified by column chromatography using different mixtures of *n*-hexane/ethyl acetate as eluent.

#### General Procedure H: Continuous Flow Hydrogenation (**20, 29–35, 62–64, 75, 80**)

Reduction of 5-nitroindoline, 5-nitroindole, and 5-nitrocarbazole derivatives was performed by continuous flow hydrogenation employing the H-Cube hydrogenator and commercially available Pd/C 10% cartridges as a catalyst. Initial nitro compounds were dissolved in a mixture of tetrahydrofuran (THF)/CH_3_OH (1:1, v/v) at a final concentration of 0.1 M and were pumped at a flow rate of 1.0 mL/min. The temperature was set at 30 °C, while the hydrogen inlet pressure was set at 10 bar. Finally, the reaction solution was evaporated *in vacuo* and the obtained products were used in the following step without further purification.

#### General Procedure I: Synthesis of Isothiocyanide (**36–42, 65–67, 76, 81**)

The proper amine (0.1 mmol) was dissolved in toluene, and 0.1 mmol of triethylamine and 0.2 mmol of carbon disulfide were added and reacted overnight. Subsequently, the organic phase was concentrated *in vacuo* and the crude was dissolved in dichloromethane, and 0.1 mmol of triethylamine (TEA) and 0.1 mmol of ethyl chloroformate were added and the mixture was stirred for 12 h at room temperature. Then, an aqueous solution (10% w/w) of NaHCO_3_ was added (3 × 100 mL) and the extracted organic solvent was dried on Na_2_SO_4_, filtered, and evaporated. Isothiocyanide derivatives were obtained after flash chromatography using *n*-hexane/ethyl acetate as eluent.

#### General Procedure J: Synthesis of Thioureas (**43–49**, **56–58**, **68–70**, **77**, **82**)

Isothiocyanide derivatives (0.1 mmol) were solubilized in dichloromethane at room temperature, and 0.15 mmol of triethylamine and 0.15 mmol of proper amine were added and the mixture was reacted for 30 min. Then, the organic phase was treated with an aqueous solution (10% w/w) of NaHCO_3_ (3 × 100 mL) and subsequently with 2 M of HCl (3 × 100 mL). The dichloromethane phase was dried on Na_2_SO_4_, filtered, and concentrated. Flash chromatography using *n*-hexane/ethyl acetate as eluent afforded thioureidic compounds.

##### (2′*S*) and (2′*R*)-4-((5-Methyl-2,4′-dioxospiro[indoline-3,2′-thiazolidin]-3′-yl)methyl)benzoic Acid (**1**)

Intermediate **1** was synthesized according to the general procedure A, starting from 5-methylisatin and 4-(aminomethyl)benzoic acid. FC in hexane/ethyl acetate 3/7, *R_f_*: 0.48. Yellowish oil (55% yield). ^1^H NMR (400 MHz, CD_3_OD): δ: 2.13 (s, 3H, C*H*_3_); 3.88 (d, 1H, C*H*_2*a*_, *J* = 15.4 Hz); 4.17 (d, 1H, C*H*_2*a*_, *J* = 14.7 Hz); 4.22 (d, 1H, C*H*_*2b*_, *J* = 15.3 Hz); 4.53 (d, 1H, C*H*_*2b*_, *J* = 14.7 Hz); 6.76 (d, 2H, aryl, *J* = 8.0 Hz); 6.81 (s, 1H, aryl); 6.98 (d, 2H, aryl, *J* = 8.1 Hz); 7.11 (d, 1H, aryl, *J* = 8.0 Hz); 7.79 (d, 2H, aryl, *J* = 7.9 Hz). HR-MS *m*/*z* calcd for C_19_H_16_N_2_O_4_S [(M + H)]^+^: 369.0904; found 369.0907.

##### (2′*S*) and (2′*R*)-4-(5-Methyl-2,4′-dioxospiro[indoline-3,2′-thiazolidin]-3′-yl)butanoic Acid (**2**)

Intermediate **2** was synthesized according to the general procedure A, starting from 5-methylisatin and 4-aminobutanoic acid. FC in hexane/ethyl acetate 3/7, *R_f_*: 0.38. Yellow powder (59% yield). ^1^H NMR (400 MHz, CD_3_OD): δ: 1.51–1.64 (m, 2H, C*H*_*2*_); 2.18–2.33 (m, 5H, C*H*_*2*_ and C*H*_3_); 2.93–3.00 (m, 1H, C*H*_*2a*_); 3.32–3.40 (m, 1H, C*H*_*2b*_); 3.64 (d, 1H, C*H*_*2a*_, *J* = 15.2 Hz); 4.08 (d, 1H, C*H*_*2b*_, *J* = 15.2 Hz); 6.78 (d, 1H, aryl, *J* = 7.9 Hz); 7.06–7.09 (m, 2H, aryl). HR-MS *m*/*z* calcd for C_15_H_16_N_2_O_4_S [(M + H)]^+^: 321.0904; found 321.0911.

##### (2′*S*) and (2′*R*)-*tert*-Butyl (3-(5-Methyl-2,4′-dioxospiro[indoline-3,2′-thiazolidin]-3′-yl)propyl)carbamate (**3**)

Intermediate **3** was synthesized according to the general procedure A, starting from 5-methylisatin and *N*-Boc-1,3-propanediamine. FC in hexane/ethyl acetate 7/3, *R_f_*: 0.48. Yellow powder (85% yield). ^1^H NMR (400 MHz, CDCl_3_): δ: 1.34 (s, 9H, C*H*_3_); 1.45–1.51 (m, 2H, C*H*_2_); 2.28 (s, 3H, C*H*_3_); 2.85–3.01 (m, 3H, C*H*_2*a*_, and C*H*_2_); 3.25–3.32 (m, 1H, C*H*_2*b*_); 3.63 (d, 1H, C*H*_*2a*_, *J* = 15.9 Hz); 4.09 (d, 1H, C*H*_*2b*_, *J* = 15.8 Hz); 5.22 (bs, 1H, N*H*); 6.74 (d, 1H, aryl, *J* = 7.0 Hz); 7.08 (d, 1H, aryl, *J* = 8.4 Hz); 7.19 (s, 1H, aryl); 8.09 (s, 1H, N*H*). HR-MS *m*/*z* calcd for C_19_H_25_N_3_O_4_S [(M + H)]^+^: 392.1639; found 392.1634.

##### (2′*S*) and (2′*R*)-4-((1-(Cyclohexanecarbonyl)-5-methyl-2,4′-dioxospiro[indoline-3,2′-thiazolidin]-3′-yl)methyl)benzoic Acid (**4**)

Derivative **4** was synthesized starting from **1** and cyclohexanecarbonyl chloride following procedure B. FC in hexane/ethyl acetate 3/7, *R_f_*: 0.48. Yellow oil (55% yield). ^1^H NMR (400 MHz, CDCl_3_): δ: 1.07–1.37 (m, 7H, C*H*_*2*_); 1.56–1.68 (m, 2H, C*H*_*2*_); 1.73 (d, 1H, C*H*_*2b*_, *J* = 12.4 Hz); 2.25 (s, 3H, C*H*_3_); 2.83–2.90 (m, 1H, C*H*_2_); 3.74 (d, 1H, C*H*_2*a*_, *J* = 15.3 Hz); 3.98 (d, 1H, C*H*_*2a*_, *J* = 14.7 Hz); 4.17 (d, 1H, C*H*_*2b*_, *J* = 15.3 Hz); 4.74 (d, 1H, C*H*_*2b*_, *J* = 14.7 Hz); 6.90 (d, 2H, aryl, *J* = 8.3 Hz); 7.02 (s, 1H, aryl); 7.18–7.21 (m, 1H, aryl); 7.81 (d, 2H, aryl, *J* = 8.3 Hz); 8.00 (d, 1H, aryl, *J* = 8.4 Hz). ^13^C NMR (100 MHz, CDCl_3_): δ: 21.0, 25.3, 25.7, 28.6, 29.1, 33.0, 44.5, 46.3, 69.1, 117.4, 122.3, 126.4, 129.1, 129.5, 130.2, 132.7, 135.9, 138.3, 140.5, 170.8, 172.3, 174.9, 176.5. HR-MS *m*/*z* calcd for C_26_H_26_N_2_O_5_S [(M + H)]^+^: 479.1635; found 479.1641.

##### (2′*S*) and (2′*R*)-4-(1-(Cyclohexanecarbonyl)-5-methyl-2,4′-dioxospiro[indoline-3,2′-thiazolidin]-3′-yl)butanoic Acid (**5**)

Derivative **5** was synthesized starting from **2** and cyclohexanecarbonyl chloride following procedure B. FC in hexane/ethyl acetate 1/1, *R_f_*: 0.47. Yellow oil (57% yield). ^1^H NMR (400 MHz, CD_3_OD): δ: 1.12–1.58 (m, 7H, C*H*_*2*_); 1.66 (d, 1H, C*H*_*2b*_, *J* = 12.5 Hz); 1.75 (d, 1H, C*H*_*2b*_, *J* = 12.8 Hz); 1.88 (d, 1H, C*H*_*2b*_, *J* = 9.5 Hz); 2.18 (dd, 2H, C*H*_*2*_, *J*′ = 7.1, *J*″ = 13.0 Hz); 2.32 (s, 3H, C*H*_3_); 2.98–3.05 (m, 1H, C*H*_2_*a*); 3.12–3.20 (m, 1H, C*H*_*2b*_); 3.38–3.52 (m, 3H, C*H*_2_ and C*H*); 3.64 (d, 1H, C*H*_*2a*_, *J* = 15.1 Hz); 4.06 (d, 1H, C*H*_*2b*_, *J* = 15.1 Hz); 7.16–7.20 (m, 2H, aryl); 8.08 (d, 1H, aryl, *J* = 8.4 Hz). ^13^C NMR (100 MHz, CD_3_OD): δ: 21.1, 23.1, 25.5, 25.8, 29.0, 31.0, 32.8, 42.7, 44.7, 65.9, 117.4, 123.2, 126.0, 132.5, 136.1, 138.2, 172.6, 172.8, 175.8, 176.8. HR-MS *m*/*z* calcd for C_22_H_26_N_2_O_5_S [(M + H)]^+^: 431.1635; found 431.1630.

##### (2′*S*) and (2′*R*)-*tert*-Butyl (3-(1-(Cyclohexanecarbonyl)-5-methyl-2,4′-dioxospiro[indoline-3,2′-thiazolidin]-3′-yl)propyl)carbamate (**6**)

Intermediate **6** was synthesized starting from **3** and cyclohexanecarbonyl chloride following procedure B. FC in hexane/ethyl acetate 8/2, *R_f_*: 0.45. Yellow oil (72% yield). ^1^H NMR (400 MHz, CDCl_3_): δ: 1.25 (t, 2H, C*H*_2_, *J* = 7.1 Hz); 1.40 (s, 9H, C*H*_3_); 1.43–1.53 (m, 5H, C*H*_2_); 1.72 (d, 1H, C*H*_2_, *J* = 12.4 Hz); 1.82 (d, 2H, C*H*_2_, *J* = 12.3 Hz); 1.92 (d, 2H, C*H*_2_, *J* = 12.3 Hz); 2.38 (s, 3H, C*H*_3_); 3.00–3.07 (m, 2H, C*H*_2_); 3.10–3.17 (m, 1H, C*H*_2*a*_); 3.21–3.28 (m, 1H, C*H*_2*b*_); 3.50 (t, 1H, C*H*, *J* = 11.1 Hz); 3.72 (d, 1H, C*H*_*2a*_, *J* = 15.2 Hz); 4.13 (d, 1H, C*H*_*2b*_, *J* = 15.1 Hz); 5.12 (bs, 1H, N*H*); 7.23 (s, 1H, aryl); 7.26 (d, 1H, aryl, *J* = 8.5 Hz); 8.13 (d, 1H, aryl, *J* = 8.4 Hz). HR-MS *m*/*z* calcd for C_26_H_35_N_3_O_5_S [(M + H)]^+^: 502.2370; found 502.2374.

##### (2′*S*) and (2′*R*)-3′-(3-Aminopropyl)-1-(cyclohexanecarbonyl)-5-methylspiro[indoline-3,2′-thiazolidine]-2,4′-dione (**7**)

Obtained from intermediate **6** following the general procedure D. FC in dichloromethane/methanol 9.5/0.5, *R_f_*: 0.45. Yellowish powder (93% yield). ^1^H NMR (400 MHz, CDCl_3_): δ: 1.22–1.32 (m, 1H, C*H*_2_); 1.36–1.46 (m, 2H, C*H*_*2*_); 1.49–1.57 (m, 3H, C*H*_*2*_); 1.76 (d, 1H, C*H*_2_, *J* = 12.8 Hz); 1.85 (d, 2H, C*H*_2_, *J* = 12.4 Hz); 1.91–1.98 (m, 3H, C*H*_2_); 2.41 (s, 3H, C*H*_3_); 3.04 (bs, 1H, N*H*); 3.26–3.40 (m, 3H, C*H*_2_ and C*H*); 3.49 (t, 2H, C*H*_2_, *J* = 9.4 Hz); 3.82 (d, 1H, C*H*_2*a*_, *J* = 15.6 Hz); 4.22 (d, 1H, C*H*_2*b*_, *J* = 15.1 Hz); 7.23 (s, 1H, aryl); 7.31 (d, 1H, aryl, *J* = 8.3 Hz); 8.16 (d, 1H, aryl, *J* = 8.2 Hz). HR-MS *m*/*z* calcd for C_21_H_27_N_3_O_3_S [(M + H)]^+^: 402.1846; found 402.1844.

##### (2′*S*) and (2′*R*)-*N*-(3-(1-(Cyclohexanecarbonyl)-5-methyl-2,4′-dioxospiro[indoline-3,2′-thiazolidin]-3′-yl)propyl)-2,3,4,5,6-pentafluorobenzamide (**8**)

Derivative **8** was synthesized starting from **7** and 2,3,4,5,6-pentafluorobenzoyl chloride following procedure B. FC in dichloromethane/ethyl acetate 9.8/0.2, *R_f_*: 0.45. Yellow oil (65% yield). ^1^H NMR (400 MHz, CD_3_OD): δ: 1.19–1.44 (m, 7H, C*H*_*2*_); 1.49–1.57 (m, 2H, C*H*_*2*_); 1.63 (d, 1H, C*H*_*2b*_, *J* = 12.3 Hz); 1.84–1.85 (m, 2H, C*H*_*2*_); 2.28 (s, 3H, C*H*_3_); 3.10 (dd, 1H, C*H*_2*a*_, *J*′ = 6.4, *J*″ = 9.0 Hz); 3.16–3.18 (m, 1H, C*H*_*2b*_); 3.19–3.22 (m, 2H, C*H*_*2*_); 3.41–3.48 (m, 1H, C*H*); 3.74, (d, 1H, C*H*_*2a*_, *J* = 15.9 Hz); 3.99 (d, 1H, C*H*_*2b*_, *J* = 15.8 Hz); 7.20 (d, 1H, aryl, *J* = 7.1 Hz); 7.29 (s, 1H, aryl); 8.00 (d, 1H, aryl, *J* = 8.4 Hz). ^13^C NMR (100 MHz, CD_3_OD): δ: 19.6, 25.2, 25.4, 25.6, 27.4, 28.6, 28.9, 32.1, 37.1, 41.1, 44.6, 69.8, 116.8, 123.1, 126.0, 132.1, 136.3, 138.2, 173.6, 176.0, 176.4. HR-MS *m*/*z* calcd for C_28_H_26_F_5_N_3_O_4_S [(M + H)]^+^: 596.1637; found 596.1642.

##### (2′*S*) and (2′*R*)-1-(3-(1-(Cyclohexanecarbonyl)-5-methyl-2,4′-dioxospiro[indoline-3,2′-thiazolidin]-3′-yl) 1,3-Diboc-2-(propyl))guanidine (**9**)

Intermediate **9** was synthesized starting from **7** and *N*-Boc-1*H*-pyrazole-1-carboxamidine following procedure E. FC in dichloromethane/methanol 9/1, *R_f_*: 0.49. Yellowish oil (45% yield). ^1^H NMR (400 MHz, CDCl_3_): δ: 1.43 (s, 18H, C*H*_3_); 1.46–1.56 (m, 7H, C*H*_2_); 1.64 (d, 1H, C*H*_*2a*_, *J* = 12.4 Hz); 1.72–1.78 (m, 2H C*H*_*2*_,); 1.86 (d, 2H, C*H*_*2*_, *J* = 12.6 Hz); 2.31 (s, 3H, C*H*_3_); 3.10 (t, 2H, C*H*_2_, *J* = 6.2 Hz); 3.31 (t, 2H, C*H*_*2*_, *J* = 7.4 Hz); 3.39–3.46 (m, 1H, C*H*); 3.68 (d, 1H, C*H*_*2a*_, *J* = 15.0 Hz); 4.06 (d, 1H, C*H*_*2b*_, *J* = 15.2 Hz); 7.14 (s, 1H, aryl); 7.17 (d, 1H, aryl, *J* = 8.4 Hz); 8.06 (d, 1H, aryl, *J* = 8.4 Hz). HR-MS *m*/*z* calcd for C_32_H_45_N_5_O_7_S [(M + H)]^+^: 644.3112; found 644.3113.

##### (2′*S*) and (2′*R*)-1-(3-(1-(Cyclohexanecarbonyl)-5-methyl-2,4′-dioxospiro[indoline-3,2′-thiazolidin]-3′-yl)propyl)guanidine (**10**)

Derivative **10** was obtained following general procedure D, starting from **9**. FC in dichloromethane/methanol 9/1, *R_f_*: 0.49. Yellow oil (92% yield). ^1^H NMR (400 MHz, CD_3_OD): δ: 1.29–1.60 (m, 7H, C*H*_*2*_); 1.73 (d, 1H, C*H*_*2a*_, *J* = 12.2 Hz); 1.80–1.85 (m, 2H C*H*_*2*_,); 1.94 (d, 2H, C*H*_*2*_, *J* = 12.2 Hz); 2.40 (s, 3H, C*H*_3_); 3.13 (t, 2H, C*H*_*2*_, *J* = 6.8 Hz); 3.21 (t, 2H, C*H*_*2*_, *J* = 7.2 Hz); 3.52–3.57 (m, 1H, C*H*); 3.86 (d, 1H, C*H*_*2a*_, *J* = 15.5 Hz); 4.10 (d, 1H, C*H*_*2b*_, *J* = 15.5 Hz); 7.33 (d, 1H, aryl, *J* = 8.4 Hz); 7.38 (s, 1H, aryl); 8.10 (d, 1H, aryl, *J* = 8.4 Hz). ^13^C NMR (100 MHz, CD_3_OD): δ: 19.7, 25.5, 25.6, 28.6, 28.9, 32.2, 38.2, 40.4, 44.6, 69.8, 116.9, 123.0, 125.9, 132.3, 136.3, 138.2, 157.2, 173.9, 176.0, 176.4. HR-MS *m*/*z* calcd for C_22_H_29_N_5_O_2_S [(M + H)]^+^: 444.2064; found 444.2070.

##### 1,5-Dimethylindoline-2,3-dione (**11**)

Intermediate **11** was obtained following general procedure C, starting from 5-methylisatin, which was reacted with methyl iodide. FC in hexane/ethyl acetate 8/2, *R_f_*: 0.45. Yellow powder (74% yield). ^1^H and DEPT NMR spectra are in accordance with the literature.^39^

##### Synthesis of (2′*S*,4′*R*) and (2′*R*,4′*R*)-Ethyl 1,5-Dimethyl-2-oxospiro[indoline-3,2′-thiazolidine]-4′-carboxylate (**12**)

To an ethanolic solution of 1,5-dimethylindoline-2,3-dione (**11**, 0.1 mmol), 0.15 mmol of l-Cys-OEt and 0.2 mmol of NaHCO_3_ were added and the solution was stirred at room temperature for 3 h. Then, ethanol was evaporated *in vacuo* and the crude was dissolved in dichloromethane and washed with water (3 × 150 mL). The organic phase was dried over Na_2_SO_4_, filtered, and evaporated. The diastereoisomeric mixture of thiazolidines **12** was almost quantitatively isolated without other treatments. FC in hexane/ethyl acetate 7/3, *R_f_*: 0.48. Yellow oil (85% yield). ^1^H and ^13^C NMR spectra are in accordance with the literature.^39^

##### (2′*S*,4′*R*)-Ethyl 3′-(Cyclohexanecarbonyl)-1,5-dimethyl-2-oxospiro[indoline-3,2′-thiazolidine]-4′-carboxylate (**13**)

Derivative **13** was synthesized according to the general procedure B, starting from **12** and cyclohexanecarbonyl chloride. FC in hexane/ethyl acetate 7/3, *R_f_*: 0.45. Yellow oil (45% yield). ^1^H and DEPT NMR spectra are in accordance with the literature.^39^

##### Synthesis of (*S*)-Benzyl (1-(Methoxy(methyl)amino)-4-methyl-1-oxopentan-2-yl)carbamate (**14**)

To a solution of *Z*-l-Leu-OH (0.1 mmol), or 4-((*tert*-butoxycarbonyl)amino)butanoic acid, or succinic acid in DCM/DMF (1/1), 0.12 mmol of HOBt, 0.12 mmol of 2-(1*H*-benzotriazole-1-yl)-1,1,3,3-tetramethyluronium hexafluorophosphate (HBTU), and 0.24 mmol of diisopropylethylamine, at room temperature, were added. After 30 min, 0.12 mmol of *N*,*O*-dimethylhydroxylamine were added and the reaction was mixed at room temperature overnight. Then, the crude was washed with water (3 × 50 mL), a 10% aqueous solution of citric acid (3 × 50 mL), and a saturated aqueous solution of sodium bicarbonate (3 × 50 mL). The combined organic layer was dried over anhydrous sodium sulfate, filtered, concentrated, and purified by flash chromatography in 70:30 *n*-hexane/ethyl acetate to give the Weinreb amide **14**. FC in hexane/ethyl acetate 7/3, *R_f_*: 0.52. Yellowish oil (90% yield). ^1^H NMR (400 MHz, CDCl_3_): δ: 0.95 (d, 3H, C*H*_3_, *J* = 6.2 Hz); 0.98 (d, 3H, C*H*_3_, *J* = 6.4 Hz); 1.49 (t, 2H, C*H*_2_, *J* = 6.8 Hz); 1.70–1.78 (m, 1H, *CH*); 3.22 (s, 3H, *CH*_3_); 3.82 (s, 3H, O*CH*_3_); 4.80–4.84 (m, 1H, *CH*); 5.11 (q, 2H, *CH*_2_); 5.36 (d, 1H, N*H*, *J* = 8.7 Hz); 7.33–7.37 (m, 5H, aryl). ESI-MS **m*/*z** calcd for C_16_H_24_N_2_O_4_ [(M + H)]^+^: 307.1652; found 307.1649.

##### Synthesis of (2*R*,4*R*,2′*S*) and (2*S*,4*R*,2′*S*)-Ethyl 2-(1-(((Benzyloxy)carbonyl)amino)-3-methylbutyl)thiazolidine-4-carboxylate (**15**)

The obtained *N*-methoxy-*N*-methylcarbamoyl derivative (0.09 mmol) was dissolved in dry THF and mixed at 0 °C under a nitrogen atmosphere. Then, 0.25 mmol of LiAlH_4_ (1 M in THF) were added and the reaction was mixed at 0 °C for 6 min. The crude was washed with a 10% aqueous solution of citric acid (3 × 50 mL), and the organic layer was dried over anhydrous sodium sulfate, filtered, and concentrated. No further purification was performed for the aldehyde intermediate.

*Z*-l-Leu-H (0.1 mmol) intermediate was dissolved in ethanol; then, 0.12 mmol of l-Cys-OEt and 0.12 mmol of NaHCO_3_ were added and the reaction was mixed at room temperature overnight. The solvent was removed *in vacuo*, and the crude was diluted with DCM and washed with water (3 × 50 mL). The collected organic layer was dried over anhydrous sodium sulfate, filtered, concentrated, and purified by flash chromatography in 40:10 *n*-hexane/ethyl acetate to obtain intermediate **15**. FC in hexane/ethyl acetate 7/3, *R_f_*: 0.57. Yellowish oil (62% yield). ^1^H NMR (400 MHz, CDCl_3_): δ: 0.93–0.99 (m, 3H, C*H*_3_); 1.26–1.33 (m, 6H, 2C*H*_3_); 1.36–1.43 (m, 2H, C*H*_2_); 1.68–1.76 (m, 1H, C*H*); 2.69 (t, 1H, *CH*_2*a*_, *J* = 10.0 Hz); 3.22–3.28 (m, 1H, *CH*_2*b*_); 3.77–3.84 (m, 1H, *CH*); 4.15 (q, 2H, *CH*_2_); 4.20–4.28 (m, 2H, C*H*_2_); 7.33–7.40 (m, 5H, aryl). ESI-MS **m*/*z** calcd for C_19_H_28_N_2_O_4_S[(M + H)]^+^: 381.1843; found 381.1840.

##### Synthesis of (3*S*,7a*R*,1′*S*) Benzyl (1-(6-(3-((*tert*-Butoxycarbonyl)amino)propyl)-5,7-dioxotetrahydro-1*H*,3*H*-imidazo[1,5-*c*]thiazol-3-yl)-3-methylbutyl)carbamate (**16**)

To 0.1 mmol of intermediate **15** dissolved in dichloromethane, 0.025 mmol of triphosgene, 0.12 mmol of TEA, and 0.12 mmol of *N*-Boc-1,3-propanediamine were added. The reaction was mixed at room temperature for 20 min and then mildly heated to allow intramolecular cyclization, generating hydantoin derivative **16**. Then, the solution was washed with water (3 × 20 mL). The combined organic layer was dried over anhydrous sodium sulfate, filtered, concentrated, and purified by flash chromatography in 50/50 *n*-hexane/ethyl acetate. FC in hexane/ethyl acetate 1/1, *R_f_*: 0.55. Yellow oil (55% yield). ^1^H NMR (400 MHz, CDCl_3_): δ: 0.95 (d, 3H, C*H*_3_, *J* = 6.2 Hz); 1.51 (s, 9H, C*H*_3_); 1.67–1.73 (m, 1H, C*H*); 1.84–1.90 (m, 2H, C*H*_2_); 2.96 (t, 1H, *CH*_2*a*_, *J* = 9.9 Hz); 3.31 (t, 1H, *CH*_2*b*_, *J* = 8.6 Hz); 3.41–3.48 (m, 2H, *CH*_2_); 3.48–3.56 (m, 2H, *CH*_2_); 4.14–4.18 (m, 1H, *CH*); 4.31 (t, 1H, *CH*, *J* = 7.1 Hz); 5.12 (s, 2H, C*H*_2_); 5.30 (s, 2H, C*H*_2_); 7.28–7.36 (m, 5H, aryl). ESI-MS **m*/*z** calcd for C_26_H_38_N_4_O_6_S [(M + H)]^+^: 535.2585; found 535.2585.

##### (3*S*,7a*R*,1′*S*) Benzyl (1-(6-(3-Aminopropyl)-5,7-dioxohexahydroimidazo[1,5-*c*]thiazol-3-yl)-3-methylbutyl)carbamate (**17**)

Derivative **17** was synthesized starting from intermediate **16** following procedure D. FC in dichloromethane/methanol 8/2, *R_f_*: 0.51. Yellow oil (87% yield). ^1^H NMR (400 MHz, CD_3_OD): δ: 0.93 (d, 3H, C*H*_3_, *J* = 6.2 Hz); 0.96 (d, 3H, C*H*_3_, *J* = 6.4 Hz); 1.42–1.46 (m, 2H, C*H*_2_); 1.66–1.79 (m, 3H, C*H*_2_ and *CH*); 2.64 (t, 2H, *CH*_*2*_, *J* = 6.9 Hz); 3.02 (d, 1H, *CH*_*2a*_, *J* = 11.0 Hz); 3.33–3.37 (m, 2H, C*H*_2b_ and *CH*); 3.55 (t, 2H, C*H*_2_, *J* = 6.7 Hz); 3.91–3.96 (m, 1H, C*H*); 5.07 (d, 1H, *CH*_*2*_*a*, *J* = 12.5 Hz); 5.16 (d, 1H, *CH*_*2b*_, *J* = 12.5 Hz); 5.22 (d, 1H, *CH*, *J* = 6.1 Hz); 7.30–7.39 (m, 5H, aryl). ^13^C NMR (100 MHz, CD_3_OD): δ: 20.5, 22.5, 24.7, 30.1, 31.8, 36.0, 37.9, 40.3, 53.7, 65.0, 66.1, 67.5, 127.3, 127.6, 128.1, 137.1, 157.4, 158.8, 171.9. ESI-MS **m*/*z** calcd for C_21_H_30_N_4_O_4_S [(M + H)]^+^: 435.2061; found 435.2051.

##### (3*S*,7a*R*,1′*S*)-Benzyl (1-(6-(3-Guanidinopropyl)-5,7-dioxohexahydroimidazo[1,5-*c*]thiazol-3-yl)-3-methylbutyl)carbamate (**18**)

Derivative **18** was synthesized starting from **17** and *N*-Boc-1*H*-1-carboxamidine following procedure E. FC in ethyl acetate/methanol 8/2, *R_f_*: 0.52. Yellow oil (55% yield). ^1^H NMR (400 MHz, CD_3_OD): δ: 0.93 (d, 3H, C*H*_3_, *J* = 6.2 Hz); 0.96 (d, 3H, C*H*_3_, *J* = 6.4 Hz); 1.43 (t, 2H, C*H*_2_, *J* = 7.0 Hz); 1.66–1.73 (m, 1H, *CH*); 1.83–1.90 (m, 2H, *CH*_*2*_); 3.04 (t, 1H, *CH*_*2a*_, *J* = 10.8 Hz); 3.18 (t, 2H, *CH*_*2*_, *J* = 6.8 Hz); 3.33–3.39 (m, 1H, C*H*_2b_); 3.56 (t, 2H, C*H*_2_, *J* = 6.7 Hz); 3.95–4.00 (m, 1H, C*H*); 4.44 (t, 1H, C*H*, *J* = 8.0 Hz); 5.07 (d, 1H, *CH*_*2a*_, *J* = 12.5 Hz); 5.16 (d, 1H, *CH*_*2b*_, *J* = 12.5 Hz); 5.23 (d, 1H, *CH*, *J* = 5.6 Hz); 7.29–7.38 (m, 5H, aryl). ^13^C NMR (100 MHz, CD_3_OD): δ: 20.5, 22.5, 24.6, 27.0, 31.6, 35.8, 38.3, 40.4, 48.5, 53.7, 65.1, 66.1, 67.3, 127.3, 127.6, 128.1, 137.1, 157.4, 158.4, 171.8. ESI-MS **m*/*z** calcd for C_22_H_32_N_6_O_4_S [(M + H)]^+^: 477.2279; found 477.2271.

##### *N*-Neopentyl-5-nitroindoline-1-carboxamide (**19**)

Obtained from intermediate 5-nitroindoline and 2,2-dimethylpropan-1-amine following the general procedure F. FC in hexane/ethyl acetate 8/2, *R_f_*: 0.47. Yellow oil (66% yield). ^1^H NMR (400 MHz, CDCl_3_): δ: 0.99 (s, 9H, C*H*_3_); 3.20 (d, 2H, C*H*_2_, *J* = 4.9 Hz); 3.32 (t, 2H, C*H*_2_, *J* = 7.0 Hz); 4.09 (t, 2H, C*H*_2_, *J* = 6.9 Hz); 4.78 (bs, 1H, N*H*); 8.02 (s, 1H, aryl); 8.07 (d, 1H, aryl, *J* = 7.2 Hz); 8.13 (d, 1H, aryl, *J* = 7.0 Hz). ESI-MS **m*/*z** calcd for C_14_H_19_N_3_O_3_ [(M + H)]^+^: 278.1499; found 278.1502.

##### 5-Amino-*N*-neopentylindoline-1-carboxamide (**20**)

Intermediate **20** was synthesized according to the general procedure H, starting from **l9**. FC in hexane/ethyl acetate 7/3, *R_f_*: 0.47. White solid (61% yield). ^1^H NMR (400 MHz, CD_3_OD): δ: 0.96 (s, 9H, C*H*_3_); 3.09–3.12 (m, 4H, C*H*_2_); 3.93 (t, 2H, C*H*_2_, *J* = 7.0 Hz); 6.57 (d, 1H, aryl, *J* = 6.8 Hz); 6.65 (s, 1H, aryl); 7.58 (d, 1H, aryl, *J* = 6.8 Hz). ESI-MS **m*/*z** calcd for C_14_H_21_N_3_O [(M + H)]^+^: 248.1757; found, 248.1755.

##### 5-((4-Fluorobenzyl)amino)-*N*-neopentylindoline-1-carboxamide (**21**)

Derivative **21** was synthesized according to the general procedure G, starting from **20** and 4-fluorobenzaldehyde. FC in hexane/ethyl acetate 7/3, *R_f_*: 0.47. Yellow oil (58% yield). ^1^H NMR (400 MHz, CD_3_OD): δ: 0.94 (s, 9H, C*H*_3_); 3.06–3.10 (m, 4H, C*H*_2_); 3.91 (t, 2H, C*H*_2_, *J* = 8.6 Hz); 4.26 (s, 2H, C*H*_2_); 6.46 (d, 1H, aryl, *J* = 8.6 Hz); 6.55 (s, 1H, aryl); 7.03 (t, 2H, aryl, *J* = 8.8 Hz); 7.36–7.40 (m, 2H, aryl); 7.54 (d, 1H, aryl, *J* = 8.6 Hz). ^13^C NMR (100 MHz, CD_3_OD): δ: 26.3, 27.7, 31.9, 50.7, 110.0, 111.7, 114.4, 114.9, 128.8, 131.7, 134.9, 136.1, 144.1, 156.4, 160.7, 163.1. ESI-MS **m*/*z** calcd for C_21_H_26_FN_3_O [(M + H)]^+^: 356.2133; found 356.2139.

##### 1-(4-Fluorobenzyl)-5-nitroindoline (**22**)

Intermediate **22** was synthesized starting from 5-nitroindoline and 4-fluorobenzaldehyde following procedure G. FC in hexane/ethyl acetate 8/2, *R_f_*: 0.48. Yellow oil (92% yield). ^1^H NMR (400 MHz, CDCl_3_): δ: 3.11 (t, 2H, C*H*_2_, *J* = 8.6 Hz); 3.63 (t, 2H, C*H*_2_, *J* = 8.9 Hz); 4.42 (s, 2H, C*H*_2_); 6.38 (d, 1H, aryl, *J* = 8.8 Hz); 7.07 (t, 2H, aryl, *J* = 8.6 Hz); 7.24–7.28 (m, 2H, aryl); 7.93 (s, 1H, aryl); 8.07 (d, 1H, aryl, *J* = 8.8 Hz). ESI-MS **m*/*z** calcd for C_15_H_13_FN_2_O_2_ [(M + H)]^+^: 273.1034; found 273.1039.

##### 1-(4-Fluorophenethyl)-5-nitroindoline (**23**)

Intermediate **23** was synthesized starting from 5-nitroindoline and (4-fluorophenyl)acetaldehyde following procedure G. FC in hexane/ethyl acetate 8/2, *R_f_*: 0.47. Yellow oil (85% yield). ^1^H NMR (400 MHz, CDCl_3_): δ: 2.89 (t, 2H, C*H*_2_, *J* = 7.2 Hz); 3.04 (t, 2H, C*H*_2_, *J* = 8.6 Hz); 3.48 (t, 2H, C*H*_2_, *J* = 7.5 Hz); 3.61 (t, 2H, C*H*_2_, *J* = 8.8 Hz); 6.18 (d, 1H, aryl, *J* = 8.8 Hz); 7.00 (t, 2H, aryl, *J* = 8.7 Hz); 7.16–7.20 (m, 2H, aryl); 7.84 (s, 1H, aryl); 8.00 (d, 1H, aryl, *J* = 6.6 Hz). ESI-MS **m*/*z** calcd for C_16_H_15_FN_2_O_2_ [(M + H)]^+^: 287.1190; found 287.1184.

##### 1-([1,1′-Biphenyl]-4-ylmethyl)-5-nitroindoline (**24**)

Obtained from 5-nitroindoline and biphenyl-4-carboxaldehyde following the general procedure G. FC in hexane/ethyl acetate 9/1, *R_f_*: 0.48. Yellow oil (68% yield). ^1^H NMR (400 MHz, CDCl_3_): δ: 3.05 (t, 2H, C*H*_2_, *J* = 8.4 Hz); 3.61 (t, 2H, C*H*_2_, *J* = 8.8 Hz); 4.41 (s, 2H, C*H*_2_); 6.33 (d, 1H, aryl, *J* = 8.8 Hz); 7.25–7.30 (m, 2H, aryl); 7.37 (t, 1H, aryl, *J* = 7.8 Hz); 7.49–7.62 (m, 6H, aryl); 7.86 (s, 1H, aryl); 8.00 (d, 1H, aryl, *J* = 10.8 Hz). ESI-MS **m*/*z** calcd for C_21_H_18_N_2_O_2_ [(M + H)]^+^: 331.1441; found 331.1445.

##### 1-(Naphthalen-2-ylmethyl)-5-nitroindoline (**25**)

Obtained from 5-nitroindoline and 2-naphthaldehyde following the general procedure G. FC in hexane/ethyl acetate 9/1, *R_f_*: 0.52. Yellow oil (62% yield). ^1^H NMR (400 MHz, CDCl_3_): δ: 3.14 (t, 2H, C*H*_2_, *J* = 8.6 Hz); 3.69 (t, 2H, C*H*_2_, *J* = 8.9 Hz); 4.61 (s, 2H, C*H*_2_); 6.45 (d, 1H, aryl, *J* = 8.8 Hz); 7.40 (d, 1H, aryl, *J* = 10.1 Hz); 7.50–7.54 (m, 2H, aryl); 7.73 (s, 1H, aryl); 7.82–7.88 (m, 3H, aryl); 7.96 (s, 1H, aryl); 8.09 (d, 1H, aryl, *J* = 9.2 Hz). ESI-MS **m*/*z** calcd for C_19_H_16_N_2_O_2_ [(M + H)]^+^: 305.1285; found 305.1291.

##### *tert*-Butyl (4-(5-Nitroindolin-1-yl)butyl)carbamate (**26**)

Obtained from 5-nitroindoline and *tert*-butyl (4-oxobutyl)carbamate following the general procedure G. FC in hexane/ethyl acetate 8/2, *R_f_*: 0.48. Yellow oil (62% yield). ^1^H NMR (400 MHz, CDCl_3_): δ: 1.19 (s, 9H, C*H*_3_); 1.65–1.72 (m, 4H, C*H*_2_); 3.14 (t, 2H, C*H*_2_, *J* = 7.8 Hz); 3.30 (t, 2H, C*H*_2_, *J* = 8.0 Hz); 3.54 (t, 2H, C*H*_2_, *J* = 7.6 Hz); 3.71 (t, 2H, C*H*_2_, *J* = 7.8 Hz); 5.17 (bs, 1H, N*H*); 6.65 (d, 1H, aryl, *J* = 8.2 Hz); 7.81 (s, 1H, aryl); 8.05 (d, 1H, aryl, *J* = 9.6 Hz). ESI-MS **m*/*z** calcd for C_17_H_25_N_3_O_4_ [(M + H)]^+^: 336.1918; found 336.1922.

##### 4-(5-Nitroindolin-1-yl)butan-1-ol (**27**)

Intermediate **27** was synthesized according to the general procedure G, starting from 5-nitroindoline and 4-hydroxybutanal. FC in hexane/ethyl acetate 1/1, *R_f_*: 0.47. Yellow oil (65% yield). ^1^H NMR (400 MHz, CD_3_OD): δ: 1.46–1.53 (m, 2H, C*H*_2_); 1.57–1.65 (m, 2H, C*H*_2_); 2.97 (t, 2H, C*H*_2_, *J* = 8.5 Hz); 3.20–3.25 (m, 2H, C*H*_2_); 3.50 (t, 2H, C*H*_2_, *J* = 6.3 Hz); 3.59 (t, 2H, C*H*_2_, *J* = 8.8 Hz); 6.30 (d, 1H, aryl, *J* = 8.9 Hz); 7.74 (s, 1H, aryl); 7.90 (d, 1H, aryl, *J* = 8.9 Hz). ESI-MS **m*/*z** calcd for C_12_H_16_N_2_O_3_ [(M + H)]^+^: 236.1155; found 236.1160.

##### Synthesis of 4-(Methoxymethoxy)benzaldehyde (**28a**)

4-Hydroxybenzaldehyde (0.2 mmol) was dissolved in DMF, and 0.3 mmol of potassium *tert*-butoxide and 0.3 mmol of methoxymethyl chloride were introduced and the solution was refluxed for 4 h. The mixture was diluted with dichloromethane and washed with 2 M of HCl (3 × 100 mL); the organic phase was dried on anhydrous Na_2_SO_4_, filtered, and concentrated. Flash chromatography in a ratio of 9/1 *n*-hexane/ethyl acetate afforded the MOM-protected compound **28a** in a rather quantitative yield. FC in hexane/ethyl acetate 9/1, *R_f_*: 0.39. White solid (97% yield). ^1^H NMR (400 MHz, CDCl_3_): δ: 3.51 (s, 3H, C*H*_3_); 5.28 (s, 2H, C*H*_2_); 7.17 (d, 2H, aryl, *J* = 8.2 Hz); 7.86 (d, 2H, aryl, *J* = 8.4 Hz); 9.92 (s, 1H, CO*H*).

##### 1-(4-(Methoxymethoxy)benzyl)-5-nitroindoline (**28**)

Intermediate **28** was synthesized according to the general procedure G, starting from 5-nitroindoline and 4-(methoxymethoxy)benzaldehyde. FC in hexane/ethyl acetate 9/1, *R_f_*: 0.48. Yellow oil (92% yield). ^1^H NMR (400 MHz, CDCl_3_): δ: 3.09 (t, 2H, C*H*_2_, *J* = 8.8 Hz); 3.50 (s, 3H, C*H*_3_); 3.63 (t, 2H, C*H*_2_, *J* = 8.6 Hz); 4.39 (s, 2H, C*H*_2_); 5.19 (s, 2H, C*H*_2_); 6.39 (d, 1H, aryl, *J* = 8.8 Hz); 7.04 (d, 2H, aryl, *J* = 8.6 Hz); 7.20 (d, 2H, aryl, *J* = 8.6 Hz); 7.92 (s, 1H, aryl); 8.07 (d, 1H, aryl, *J* = 11.0 Hz). ESI-MS **m*/*z** calcd for C_17_H_18_N_2_O_4_ [(M + H)]^+^: 315.1339; found 315.1336.

##### 1-(4-Fluorobenzyl)indolin-5-amine (**29**)

Intermediate **29** was obtained following general procedure H, starting from **22**. FC in hexane/ethyl acetate 1/1, *R_f_*: 0.41. White solid (95% yield). ^1^H NMR (400 MHz, CDCl_3_): δ: 2.79 (t, 2H, C*H*_2_, *J* = 7.4 Hz); 3.08 (t, 2H, C*H*_2_, *J* = 7.6 Hz); 3.26 (bs, 2H, N*H*_2_); 4.01 (s, 2H, C*H*_2_); 6.26 (d, 1H, aryl, *J* = 8.1 Hz); 6.37 (d, 1H, aryl, *J* = 8.0 Hz); 6.50 (s, 1H, aryl); 6.93 (t, 2H, aryl, *J* = 8.7 Hz); 7.24–7.27 (m, 2H, aryl). ESI-MS **m*/*z** calcd for C_15_H_15_FN_2_ [(M + H)]^+^: 243.1292; found 243.1300.

##### 1-(4-Fluorophenethyl)indolin-5-amine (**30**)

Intermediate **30** was obtained following general procedure H, starting from **23**. FC in hexane/ethyl acetate 7/3, *R_f_*: 0.48. Yellow oil (82% yield). ^1^H NMR (400 MHz, CDCl_3_): δ: 2.88 (t, 2H, C*H*_2_, *J* = 7.3 Hz); 3.13–3.32 (m, 6H, C*H*_2_); 6.38 (d, 1H, aryl, *J* = 8.0 Hz); 6.49 (d, 1H, aryl, *J* = 8.2 Hz); 6.59 (s, 1H, aryl); 7.02 (t, 2H, aryl, *J* = 8.7 Hz); 7.21–7.26 (m, 2H, aryl). ESI-MS **m*/*z** calcd for C_16_H_17_FN_2_ [(M + H)]^+^: 257.1449; found 257.1455.

##### 1-([1,1′-Biphenyl]-4-ylmethyl)indolin-5-amine (**31**)

Intermediate **31** was obtained following general procedure H, starting from **24**. FC in hexane/ethyl acetate 1/1, *R_f_*: 0.50. Yellow oil (65% yield). ^1^H NMR (400 MHz, CDCl_3_): δ: 2.78 (t, 2H, C*H*_2_, *J* = 7.6 Hz); 3.14 (t, 2H, C*H*_2_, *J* = 7.6 Hz); 3.87 (bs, 2H, N*H*_2_); 4.10 (s, 2H, C*H*_2_); 6.31 (d, 1H, aryl, *J* = 8.2 Hz); 6.95 (t, 1H, aryl, *J* = 7.8 Hz); 7.14–7.18 (m, 3H, aryl); 7.26 (s, 1H, aryl); 7.46 (s, 1H, aryl); 7.90–7.98 (m, 5H, aryl). ESI-MS **m*/*z** calcd for C_21_H_20_N_2_ [(M + H)]^+^: 301.1699; found 301.1705.

##### 1-(Naphthalen-2-ylmethyl)indolin-5-amine (**32**)

Intermediate **32** was obtained following general procedure H, starting from **25**. FC in hexane/ethyl acetate 7/3, *R_f_*: 0.47. Yellow oil (59% yield). ^1^H NMR (400 MHz, CDCl_3_): δ: 2.92 (t, 2H, C*H*_2_, *J* = 7.5 Hz); 3.20 (t, 2H, C*H*_2_, *J* = 7.4 Hz); 3.55 (bs, 2H, N*H*_2_); 4.14 (s, 2H, C*H*_2_); 6.29 (d, 1H, aryl, *J* = 7.8 Hz); 7.01–7.08 (m, 2H, aryl); 7.13 (s, 1H, aryl); 7.86–7.95 (m, 6H, aryl). ESI-MS **m*/*z** calcd for C_19_H_18_N_2_ [(M + H)]^+^: 275.1543; found 275.1539.

##### *tert*-Butyl (4-(5-Aminoindolin-1-yl)butyl)carbamate (**33**)

Intermediate **33** was synthesized starting from **26** following procedure H. FC in hexane/ethyl acetate 1/1, *R_f_*: 0.47. Yellow oil (59% yield). ^1^H NMR (400 MHz, CDCl_3_): δ: 1.12 (s, 9H, C*H*_3_); 1.58–1.62 (m, 2H, C*H*_2_); 1.69–1.73 (m, 2H, C*H*_2_); 2.85 (t, 2H, C*H*_2_, *J* = 7.4 Hz); 3.04 (t, 2H, C*H*_2_, *J* = 7.6 Hz); 3.19 (t, 2H, C*H*_2_, *J* = 7.6 Hz); 3.20 (t, 2H, C*H*_2_, *J* = 7.4 Hz); 5.21 (bs, 1H, N*H*); 6.23–6.29 (m, 2H, aryl); 7.68 (s, 1H, aryl). ESI-MS **m*/*z** calcd for C_17_H_27_N_3_O_2_ [(M + H)]^+^: 306.2176; found 306.2182.

##### 4-(5-Aminoindolin-1-yl)butan-1-ol (**34**)

Intermediate **34** was synthesized starting from **27** following procedure H. FC in hexane/ethyl acetate 2/8, *R_f_*: 0.48. Yellow oil (58% yield). ^1^H NMR (400 MHz, CD_3_OD): δ: 2.43–2.49 (m, 2H, C*H*_2_); 2.74–2.78 (m, 2H, C*H*_2_); 2.86 (t, 2H, C*H*_2_, *J* = 8.5 Hz); 3.02 (t, 2H, C*H*_2_, *J* = 8.2 Hz); 4.09–4.24 (m, 4H, C*H*_2_); 6.43 (d, 1H, aryl, *J* = 8.6 Hz); 6.53 (s, 1H, aryl); 7.75 (d, 1H, aryl, *J* = 8.6 Hz). ESI-MS **m*/*z** calcd for C_12_H_18_N_2_O [(M + H)]^+^: 207.1492; found 207.1488.

##### 1-(4-(Methoxymethoxy)benzyl)indolin-5-amine (**35**)

Intermediate **35** was synthesized starting from **28** following procedure H. FC in hexane/ethyl acetate 1/1, *R_f_*: 0.47. White solid (90% yield). ^1^H NMR (400 MHz, CDCl_3_): δ: 3.00 (t, 2H, C*H*_2_, *J* = 8.7 Hz); 3.39 (s, 3H, C*H*_3_); 3.44 (t, 2H, C*H*_2_, *J* = 8.6 Hz); 4.21 (s, 2H, C*H*_2_); 5.03 (s, 2H, C*H*_2_); 6.20 (d, 1H, aryl, *J* = 8.4 Hz); 6.95 (d, 2H, aryl, *J* = 8.2 Hz); 7.07 (d, 2H, aryl, *J* = 8.1 Hz); 7.77 (s, 1H, aryl); 7.93 (d, 1H, aryl, *J* = 10.2 Hz). ESI-MS **m*/*z** calcd for C_17_H_20_N_2_O_2_ [(M + H)]^+^: 285.1598; found 285.1604.

##### 1-(4-Fluorobenzyl)-5-thiocyanatoindoline (**36**)

Obtained from intermediate **29** following the general procedure I. FC in hexane/ethyl acetate 9/1, *R_f_*: 0.48. White solid (58% yield). ^1^H NMR (400 MHz, CDCl_3_): δ: 2.99 (t, 2H, C*H*_2_, *J* = 8.4 Hz); 3.40 (t, 2H, C*H*_2_, *J* = 8.5 Hz); 4.26 (s, 2H, C*H*_2_); 6.37 (d, 1H, aryl, *J* = 8.3 Hz); 6.94 (s, 1H, aryl); 6.97 (d, 1H, aryl, *J* = 5.5 Hz); 7.05 (t, 2H, aryl, *J* = 8.6 Hz); 7.28–7.32 (m, 2H, aryl). ESI-MS **m*/*z** calcd for C_16_H_13_FN_2_S [(M + H)]^+^: 285.0856; found 285.0849.

##### 1-(4-Fluorophenethyl)-5-isothiocyanatoindoline (**37**)

Obtained from intermediate **30** following the general procedure I. FC in hexane/ethyl acetate 9/1, *R_f_*: 0.47. Yellow oil (62% yield). ^1^H NMR (400 MHz, CDCl_3_): δ: 2.75 (t, 2H, C*H*_2_, *J* = 7.1 Hz); 2.86 (t, 2H, C*H*_2_, *J* = 8.4 Hz); 3.22 (t, 2H, C*H*_2_, *J* = 7.7 Hz); 3.46 (t, 2H, C*H*_2_, *J* = 8.5 Hz); 6.16 (d, 1H, aryl, *J* = 8.9 Hz); 6.82–6.84 (m, 2H, aryl); 6.90 (t, 2H, aryl, *J* = 8.7 Hz); 7.07–7.11 (m, 2H, aryl). ESI-MS **m*/*z** calcd for C_17_H_15_FN_2_S [(M + H)]^+^: 299.1013; found 299.1018.

##### 1-([1,1′-Biphenyl]-4-ylmethyl)-5-isothiocyanatoindoline (**38**)

Intermediate **38** was synthesized starting from **31** following procedure I. FC in hexane/ethyl acetate 9.5/0.5, *R_f_*: 0.47. Yellow oil (57% yield). ^1^H NMR (400 MHz, CDCl_3_): δ: 2.76 (t, 2H, C*H*_2_, *J* = 7.6 Hz); 3.12 (t, 2H, C*H*_2_, *J* = 8.0 Hz); 4.13 (s, 2H, C*H*_2_); 6.35 (d, 1H, aryl, *J* = 8.1 Hz); 6.60 (t, 1H, aryl, *J* = 8.4 Hz); 7.07–7.12 (m, 2H, aryl); 7.18 (d, 1H, aryl, *J* = 8.6 Hz); 7.29 (s, 1H, aryl); 7.55 (d, 1H, aryl, *J* = 8.4 Hz); 7.86–7.94 (m, 5H, aryl). ESI-MS **m*/*z** calcd for C_22_H_18_N_2_S [(M + H)]^+^: 343.1263; found 343.1258.

##### 5-Isothiocyanato-1-(naphthalen-2-ylmethyl)indoline (**39**)

Intermediate **39** was synthesized starting from **32** following procedure I. FC in hexane/ethyl acetate 9.5/0.5, *R_f_*: 0.47. Yellow oil (48% yield). ^1^H NMR (400 MHz, CDCl_3_): δ: 2.90 (t, 2H, C*H*_2_, *J* = 7.4 Hz); 3.14 (t, 2H, C*H*_2_, *J* = 8.0 Hz); 4.22 (s, 2H, C*H*_2_); 6.41 (d, 1H, aryl, *J* = 8.4 Hz); 6.90–6.96 (m, 2H, aryl); 7.08 (d, 1H, aryl, *J* = 8.3 Hz); 7.41 (s, 1H, aryl); 7.65–7.78 (m, 5H, aryl). ESI-MS **m*/*z** calcd for C_20_H_16_N_2_S [(M + H)]^+^: 317.1107; found 317.1104.

##### *tert*-Butyl (4-(5-Isothiocyanatoindolin-1-yl)butyl)carbamate (**40**)

Intermediate **40** was synthesized starting from **33** following procedure I. FC in hexane/ethyl acetate 9.5/0.5, *R_f_*: 0.52. Yellow oil (57% yield). ^1^H NMR (400 MHz, CDCl_3_): δ: 1.20 (s, 9H, C*H*_3_); 1.58–1.63 (m, 2H, C*H*_2_); 1.70–1.75 (m, 2H, C*H*_2_); 2.81 (t, 2H, C*H*_2_, *J* = 7.8 Hz); 3.00 (t, 2H, C*H*_2_, *J* = 8.0 Hz); 3.14 (t, 2H, C*H*_2_, *J* = 7.8 Hz); 3.25 (t, 2H, C*H*_2_, *J* = 7.6 Hz); 5.05 (bs, 1H, N*H*); 6.49 (d, 1H, aryl, *J* = 9.4 Hz); 7.22 (s, 1H, aryl); 8.03 (d, 1H, aryl, *J* = 11.2 Hz). ESI-MS **m*/*z** calcd for C_18_H_25_N_3_O_2_S [(M + H)]^+^: 348.1740; found 348.1736.

##### 4-(5-Isothiocyanatoindolin-1-yl)butan-1-ol (**41**)

Intermediate **41** was synthesized starting from **34** following procedure I. FC in hexane/ethyl acetate 1/1, *R_f_*: 0.50. Yellow oil (45% yield). ^1^H NMR (400 MHz, CD_3_OD): δ: 1.68–1.76 (m, 4H, C*H*_2_); 3.00 (t, 2H, C*H*_2_, *J* = 8.0 Hz); 3.15 (t, 2H, C*H*_2_, *J* = 4.0 Hz); 3.48 (t, 2H, C*H*_2_, *J* = 4.0 Hz); 3.73 (t, 2H, C*H*_2_, *J* = 4.0 Hz); 6.41 (d, 1H, aryl, *J* = 8.0 Hz); 6.97–6.99 (m, 2H, aryl). ESI-MS **m*/*z** calcd for C_13_H_16_N_2_OS [(M + H)]^+^: 248.0978; found 248.0984.

##### 5-Isothiocyanato-1-(4-(methoxymethoxy)benzyl)indoline (**42**)

Obtained from intermediate **35** following the general procedure I. FC in hexane/ethyl acetate 9/1, *R_f_*: 0.47. Yellow oil (77% yield). ^1^H NMR (400 MHz, CDCl_3_): δ: 2.97 (t, 2H, C*H*_2_, *J* = 8.4 Hz); 3.40 (t, 2H, C*H*_2_, *J* = 8.5 Hz); 3.51 (s, 3H, C*H*_3_); 4.23 (s, 2H, C*H*_2_); 5.19 (s, 2H, C*H*_2_); 6.39 (d, 1H, aryl, *J* = 8.0 Hz); 6.94–6.96 (m, 2H, aryl); 7.03 (d, 2H, aryl, *J* = 8.6 Hz); 7.25 (d, 2H, aryl, *J* = 8.6 Hz). ESI-MS **m*/*z** calcd for C_18_H_18_N_2_O_2_S [(M + H)]^+^: 327.1162; found 327.1155.

##### 1-(1-(4-Fluorobenzyl)indolin-5-yl)-3-neopentylthiourea (**43**)

Synthesis of **43** was previously described by Ostacolo and co-workers.

Derivative **43** was synthesized according to the general procedure J, starting from **36** and 2,2-dimethylpropan-1-amine. FC in hexane/ethyl acetate 7/3, *R_f_*: 0.45. Yellow oil (52% yield). ^1^H and DEPT NMR spectra are in accordance with the literature.^41^

##### 1-(1-(4-Fluorophenethyl)indolin-5-yl)-3-neopentylthiourea (**44**)

Derivative **44** was synthesized according to the general procedure J, starting from **37** and 2,2-dimethylpropan-1-amine. FC in dichloromethane/ethyl acetate 9.5/0.5, *R_f_*: 0.47. Yellow oil (65% yield). ^1^H NMR (400 MHz, CD_3_OD): δ: 0.91 (s, 9H, C*H*_3_); 2.89 (t, 2H, C*H*_2_, *J* = 7.1 Hz); 2.95 (t, 2H, C*H*_2_, *J* = 8.4 Hz); 3.33–3.37 (m, 2H, C*H*_2_); 3.43 (t, 4H, C*H*_2_, *J* = 8.4 Hz); 6.46 (d, 1H, aryl, *J* = 8.2 Hz); 6.89 (d, 1H, aryl, *J* = 8.2 Hz); 6.94 (s, 1H, aryl); 7.01 (t, 2H, aryl, *J* = 8.8 Hz); 7.27–7.31 (m, 2H, aryl). ^13^C NMR (100 MHz, CD_3_OD): δ: 26.4, 27.8, 32.2, 50.4, 52.8, 55.4, 106.4, 114.5, 114.7, 122.6, 125.4, 130.2, 131.3, 135.8, 151.4, 160.4, 162.8, 181.6. ESI-MS **m*/*z** calcd for C_22_H_28_FN_3_S [(M + H)]^+^: 386.2061; found 386.2055.

##### 1-(1-([1,1′-Biphenyl]-4-ylmethyl)indolin-5-yl)-3-neopentylthiourea (**45**)

Derivative **45** was synthesized according to the general procedure J, starting from **38** and 2,2-dimethylpropan-1-amine. FC in hexane/ethyl acetate 8/2, *R_f_*: 0.47. Yellow oil (63% yield). ^1^H NMR (400 MHz, CD_3_OD): δ: 0.80 (s, 9H, C*H*_3_); 2.87 (t, 2H, C*H*_2_, *J* = 7.1 Hz); 3.29 (t, 2H, C*H*_2_, *J* = 8.3 Hz); 3.33 (bs, 2H, C*H*_2_); 4.23 (s, 2H, C*H*_2_); 6.49 (d, 1H, aryl, *J* = 8.2 Hz); 6.81 (d, 1H, aryl, *J* = 6.4 Hz); 6.88 (s, 1H, aryl); 7.22 (t, 2H, aryl, *J* = 7.4 Hz); 7.30–7.35 (m, 4H, aryl); 7.50 (t, 4H, aryl, *J* = 8.0 Hz). ^13^C NMR (100 MHz, CD_3_OD): δ: 26.4, 27.8, 29.4, 52.6, 53.2, 55.4, 106.9, 122.7, 125.3, 126.5, 126.7, 128.4, 137.2, 140.1, 140.8, 151.7, 181.7. ESI-MS **m*/*z** calcd for C_27_H_31_N_3_S [(M + H)]^+^: 430.2311; found 430.2306.

##### 1-(1-(Naphthalen-2-ylmethyl)indolin-5-yl)-3-neopentylthiourea (**46**)

Derivative **46** was synthesized according to the general procedure J, starting from **39** and 2,2-dimethylpropan-1-amine. FC in hexane/ethyl acetate 7/3, *R_f_*: 0.46. White solid (55% yield). ^1^H NMR (400 MHz, CD_3_OD): δ: 0.92 (s, 9H, C*H*_3_); 3.00 (t, 2H, C*H*_2_, *J* = 8.4 Hz); 3.37–3.45 (m, 4H, C*H*_2_); 4.46 (s, 2H, C*H*_2_); 6.62 (d, 1H, aryl, *J* = 8.2 Hz); 6.91–6.93 (m, 1H, aryl); 7.00 (s, 1H, aryl); 7.46–7.53 (m, 3H, aryl); 7.81–7.86 (m, 5H, aryl); ^13^C NMR (100 MHz, CDCl_3_): δ: 26.4, 27.8, 53.2, 53.4, 55.4, 106.9, 122.7, 125.4, 125.7, 125.8, 126.2, 127.2, 127.3, 127.8, 132.9, 133.5, 135.7, 151.7, 175.0. ESI-MS **m*/*z** calcd for C_25_H_29_N_3_S [(M + H)]^+^: 404.2155; found 404.2160.

##### *tert*-Butyl (4-(5-(3-Neopentylthioureido)indolin-1-yl)butyl)carbamate (**47**)

Intermediate **47** was synthesized starting from **40** and 2,2-dimethylpropan-1-amine following procedure J. FC in hexane/ethyl acetate 7/3, *R_f_*: 0.48. Yellow oil (52% yield). ^1^H NMR (400 MHz, CD_3_OD): δ: 0.89 (s, 9H, C*H*_3_); 1.16 (s, 9H, C*H*_3_); 1.65–1.74 (m, 4H, C*H*_2_); 3.00 (t, 4H, C*H*_2_, *J* = 7.4 Hz); 3.09 (t, 2H, C*H*_2_, *J* = 7.0 Hz); 3.43 (t, 4H, C*H*_2_, *J* = 8.2 Hz); 5.48 (bs, 1H, N*H*); 6.60 (d, 1H, aryl, *J* = 8.5 Hz); 7.01 (d, 1H, aryl, *J* = 9.1 Hz); 7.03 (s, 1H, aryl). ESI-MS **m*/*z** calcd for C_23_H_38_N_4_O_2_S [(M + H)]^+^: 435.2788; found 435.2782.

##### 1-(1-(4-Hydroxybutyl)indolin-5-yl)-3-neopentylthiourea (**48**)

Derivative **48** was synthesized according to the general procedure J, starting from **41** and 2,2-dimethylpropan-1-amine. FC in dichloromethane/ethyl acetate 7/3, *R_f_*: 0.49. Yellow oil (55% yield). ^1^H NMR (400 MHz, CD_3_OD): δ: 0.91 (s, 9H, C*H*_3_); 1.61–1.74 (m, 4H, C*H*_2_); 2.96 (t, 2H, C*H*_2_, *J* = 8.4 Hz); 3.12 (t, 2H, C*H*_2_, *J* = 7.2 Hz); 3.37–3.45 (m, 4H, C*H*_2_); 3.63 (t, 2H, C*H*_2_, *J* = 7.2 Hz); 6.52 (d, 1H, aryl, *J* = 8.2 Hz); 6.92 (d, 1H, aryl, *J* = 8.2 Hz); 6.96 (s, 1H, aryl). ^13^C NMR (100 MHz, CD_3_OD): δ: 23.4, 26.4, 27.8, 29.8, 48.7, 52.8, 55.4, 61.3, 106.6, 122.6, 125.4, 131.4, 151.9, 181.6. ESI-MS **m*/*z** calcd for C_18_H_29_N_3_OS [(M + H)]^+^: 336.2104; found 336.2107.

##### 1-(1-(4-(Methoxymethoxy)benzyl)indolin-5-yl)-3-neopentylthiourea (**49**)

Derivative **49** was synthesized according to the general procedure J, starting from **42** and 2,2-dimethylpropan-1-amine. FC in hexane/ethyl acetate 1/1, *R_f_*: 0.47. Yellow oil (67% yield). ^1^H NMR (400 MHz, dimethyl sulfoxide (DMSO)): δ: 0.89 (s, 9H, C*H*_3_); 2.87 (t, 2H, C*H*_2_, *J* = 8.4 Hz); 3.24 (t, 2H, C*H*_2_, *J* = 8.2 Hz); 3.31–3.33 (m, 5H, C*H*_2_ and C*H*_3_); 4.19 (s, 2H, C*H*_2_); 5.17 (s, 2H, C*H*_2_); 6.55 (d, 1H, aryl, *J* = 8.3 Hz); 6.89 (d, 1H, aryl, *J* = 8.3 Hz); 6.98–7.03 (m, 3H, aryl); 7.09 (bs, 1H, N*H*); 7.27 (d, 2H, aryl, *J* = 8.6 Hz); 9.15 (bs, 1H, N*H*). ^13^C NMR (100 MHz, DMSO): δ: 27.8, 28.3, 52.5, 53.4, 55.3, 56.0, 94.4, 107.0, 116.6, 122.4, 124.4, 129.7, 130.6, 131.7, 150.4, 156.4, 182.1. ESI-MS **m*/*z** calcd for C_23_H_31_N_3_O_2_S [(M + H)]^+^: 414.2210; found 414.2206.

##### Synthesis of 1-(1-(4-Fluorobenzyl)indolin-5-yl)-3-neopentylguanidine (**50**)

**43** (0.05 mmol) was solubilized in dry THF, and 3.8 mmol of Na_2_SO_4_, two beads of CaCl_2_, and 7.0 mmol of triethylamine were introduced and the mixture was stirred for 30 min at room temperature. Then, 2.05 mmol of HgO were added and the solution was refluxed for 1 h. The obtained mixture was filtered into an ammonium hydroxide solution, and the supernatant was stirred at room temperature and checked by TLC until the carbodiimide intermediate disappeared. Subsequently, the hydroxide was quenched by 2 M of HCl and the aqueous layer was extracted with ethyl acetate (3 × 100 mL); the organic solvent was dried on Na_2_SO_4_, filtered, and concentrated. The final compound **50** was isolated after flash chromatography using ethyl acetate as the mobile phase. FC in ethyl acetate/methanol 9/1, *R_f_*: 0.42. White powder (47% yield). ^1^H and DEPT NMR spectra are in accordance with the literature.^41^ ESI-MS **m*/*z** calcd for C_21_H_27_FN_4_ [(M + H)]^+^: 355.2293; found 355.2288.

##### 1-(1-(4-Aminobutyl)indolin-5-yl)-3-neopentylthiourea (**51**)

Derivative **51** was synthesized starting from **47** following procedure D. FC ethyl acetate/methanol 9/1, *R_f_*: 0.42. Yellow oil (52% yield). ^1^H NMR (400 MHz, CD_3_OD): δ: 0.92 (s, 9H, C*H*_3_); 1.71–1.79 (m, 4H, C*H*_2_); 2.98 (t, 4H, C*H*_2_, *J* = 6.6 Hz); 3.14 (t, 2H, C*H*_2_, *J* = 6.2 Hz); 3.41 (t, 2H, C*H*_2_, *J* = 8.3 Hz); 3.45 (bs, 2H, C*H*_2_); 6.54 (d, 1H, aryl, *J* = 8.2 Hz); 6.94 (d, 1H, aryl, *J* = 8.3 Hz); 6.98 (s, 1H, aryl). ^13^C NMR (100 MHz, CD_3_OD): δ: 23.7, 24.3, 25.4, 27.2, 31.2, 47.3, 51.9, 54.4, 105.2, 121.9, 124.7, 126.3, 130.3, 150.7, 180.8. ESI-MS **m*/*z** calcd for C_18_H_30_N_4_S [(M + H)]^+^: 335.2264; found 335.2270.

##### Synthesis of 1-(1-(4-Hydroxybenzyl)indolin-5-yl)-3-neopentylthiourea (**52**)

Methoxymethyl protecting group was removed by dissolving compound **49** in a solution of DCM/TFA (10 mL, 1:1 v-v) at room temperature for 3 h. After quenching by Na_2_CO_3_, the mixture was washed with water (3 × 100 mL) and the organic layer was dried on Na_2_SO_4_, filtered, and evaporated *in vacuo*. The final derivative was obtained after flash chromatography in hexane/ethyl acetate 7/3. FC in hexane/ethyl acetate 7/3, *R_f_*: 0.51. Yellow oil (62% yield). ^1^H NMR (400 MHz, CD_3_OD): δ: 0.91 (s, 9H, C*H*_3_); 2.93 (t, 2H, C*H*_2_, *J* = 8.3 Hz); 3.30 (t, 2H, C*H*_2_, *J* = 8.2 Hz); 3.44 (bs, 2H, C*H*_2_); 4.18 (s, 2H, C*H*_2_); 6.59 (d, 1H, aryl, *J* = 8.2 Hz); 6.76 (d, 2H, aryl, *J* = 8.5 Hz); 6.91 (d, 1H, aryl, *J* = 8.2 Hz); 6.96 (s, 1H, aryl); 7.18 (d, 2H, aryl, *J* = 8.5 Hz). ^13^C NMR (100 MHz, CD_3_OD): δ: 26.4, 27.7, 32.01, 52.4, 52.9, 55.4, 107.0, 114.8, 122.7, 125.3, 128.6, 129.1, 151.7, 156.4, 181.6. ESI-MS **m*/*z** calcd for C_21_H_27_N_3_OS [(M + H)]^+^: 370.1948; found 370.1956.

##### 1-Cyclohexyl-3-(1-(4-fluorobenzyl)indolin-5-yl)urea (**53**)

Derivative **53** was obtained following general procedure F, starting from **29**, which was reacted with cyclohexylamine. FC in hexane/ethyl acetate 7/3, *R_f_*: 0.47. Yellow oil (72% yield). ^1^H NMR (400 MHz, DMSO): δ: 1.09–1.20 (m, 3H, C*H*_2_); 1.24–1.36 (m, 2H, C*H*_2_); 1.53–1.56 (m, 1H, C*H*_2_); 1.63–1.67 (m, 2H, C*H*_2_); 1.77–1.80 (m, 2H, C*H*_2_); 2.82 (t, 2H, C*H*_2_, *J* = 8.2 Hz); 3.14 (t, 2H, C*H*_2_, *J* = 8.1 Hz); 3.40–3.51 (m, 1H, C*H*); 4.05 (s, 1H, N*H*); 4.16 (s, 2H, C*H*_2_); 5.84 (d, 1H, aryl, *J* = 7.9 Hz); 6.47 (d, 1H, aryl, *J* = 8.4 Hz); 6.90 (d, 1H, aryl, *J* = 8.3 Hz); 7.13–7.19 (m, 2H, aryl); 7.37–7.41 (m, 2H, aryl); 7.85 (s, 1H, N*H*). ^13^C NMR (100 MHz, DMSO): δ: 24.9, 25.7, 28.7, 33.6, 40.0, 53.3, 53.8, 107.7, 115.4, 115.6, 116.7, 117.8, 130.40, 130.52, 132.0, 135.1, 147.6, 155.3, 162.9. ESI-MS **m*/*z** calcd for C_22_H_27_FN_3_O [(M + H)]^+^: 368.4671; found 368.4678.

##### 1-(1-(4-Fluorobenzyl)indolin-5-yl)-3-neopentylurea (**54**)

Derivative **54** was obtained following general procedure F, starting from **29**, which was reacted with 2,2-dimethylpropan-1-amine. FC in dichloromethane/ethyl acetate 7/3, *R_f_*: 0.45. Yellow oil (78% yield). ^1^H NMR (400 MHz, CD_3_OD): δ: 0.82 (s, 9H, C*H*_3_); 2.79 (t, 2H, C*H*_2_, *J* = 8.1 Hz); 2.89 (s, 2H, C*H*_2_); 3.11 (t, 2H, C*H*_2_, *J* = 8.1 Hz); 4.07 (s, 2H, C*H*_2_); 6.38 (d, 1H, aryl, *J* = 8.3 Hz); 6.81 (d, 1H, aryl, *J* = 10.3 Hz); 6.92–6.98 (m, 3H, aryl); 7.26–7.29 (m, 2H, aryl). ^13^C NMR (100 MHz, CD_3_OD): δ: 26.1, 28.1, 31.5, 50.7, 53.2, 53.6, 107.2, 114.5, 114.7, 118.4, 120.1, 129.5, 130.2, 130.8, 134.4, 148.8, 158.1, 160.9, 163.3. ESI-MS **m*/*z** calcd for C_21_H_26_FN_3_O [(M + H)]^+^: 356.2133; found 356.2128.

##### Synthesis of *N*-(1-(4-Fluorobenzyl)indolin-5-yl)cyclohexanesulfonamide (**55**)

Intermediate **29** (0.1 mmol) was dissolved in dry DCM under a nitrogen positive pressure. To this solution, 0.12 mmol of 1,8-diazabicyclo[5.4.0]undec-7-ene were added. The solution was stirred at room temperature for 15 min; then, 0.15 mmol of cyclohexylsulfonyl chloride was introduced and further stirred for 2 h. The reaction was then washed with a saturated solution of NaHCO_3_ and brine. The organic phase was extracted, dried over anhydrous Na_2_SO_4_, filtered, and concentrated *in vacuo*. The final product was purified using a 50:50 mixture of *n*-hexane/ethyl acetate as eluent. FC in hexane/ethyl acetate 8/2, *R_f_*: 0.44. Off-white oil (78% yield). ^1^H and DEPT NMR spectra are in accordance with the literature.^41^ ESI-MS **m*/*z** calcd for C_21_H_25_FN_2_O_2_S [(M + H)]^+^: 389.1694; found 389.1687.

##### 1-Benzyl-3-(1-(4-fluorobenzyl)indolin-5-yl)thiourea (**56**)

Derivative **56** was synthesized according to the general procedure J, starting from **36** and benzylamine. FC in hexane/ethyl acetate 7/3, *R_f_*: 0.47. Yellow oil (58% yield). ^1^H NMR (400 MHz, CDCl_3_): δ: 2.97 (t, 2H, C*H*_2_, *J* = 8.4 Hz); 3.38 (t, 2H, C*H*_2_, *J* = 8.4 Hz); 4.22 (s, 2H, *CH*_2_); 4.89 (d, 2H, C*H*_2_, *J* = 5.5 Hz); 6.12 (bs, 1H, N*H*); 6.40 (d, 1H, aryl, *J* = 8.2 Hz); 6.89–6.93 (m, 2H, aryl); 7.04 (t, 2H, aryl, *J* = 8.6 Hz); 7.28–7.37 (m, 7H, aryl); 7.63 (bs, 1H, N*H*). ^13^C NMR (100 MHz, CDCl_3_): δ: 28.2, 49.3, 52.4, 53.4, 107.0, 115.4, 115.6, 123.4, 125.0, 126.3, 127.6, 128.7, 129.3, 132.0, 133.3, 137.7, 152.2, 163.4, 181.9. ESI-MS **m*/*z** calcd for C_23_H_22_FN_3_S [(M + H)]^+^: 392.1591; found 392.1599.

##### 1-(1-(4-Fluorobenzyl)indolin-5-yl)-3-phenylthiourea (**57**)

Derivative **57** was synthesized according to the general procedure J, starting from **36** and aniline. FC in hexane/ethyl acetate 8/2, *R_f_*: 0.48. Yellow oil (67% yield). ^1^H NMR (400 MHz, CD_3_OD): δ: 2.96 (t, 2H, C*H*_2_, *J* = 8.3 Hz); 3.32 (t, 2H, C*H*_2_, *J* = 8.3 Hz); 4.25 (s, 2H, *CH*_2_); 6.54 (d, 2H, aryl, *J* = 8.3 Hz); 6.96 (d, 1H, aryl, *J* = 8.2 Hz); 7.06 (t, 3H, aryl, *J* = 11.1 Hz); 7.19 (t, 1H, aryl, *J* = 7.2 Hz); 7.32–7.41 (m, 5H, aryl). ^13^C NMR (100 MHz, CD_3_OD): δ: 27.8, 52.4, 53.3, 106.7, 114.6, 114.8, 122.5, 124.8, 125.3, 128.4, 129.5, 131.0, 134.2, 139.0, 151.3, 160.9, 163.3, 180.6. ESI-MS **m*/*z** calcd for C_22_H_20_FN_3_S [(M + H)]^+^: 378.1435; found 378.1442.

##### 4-(3-(1-(4-Fluorobenzyl)indolin-5-yl)thioureido)benzoic Acid (**58**)

Derivative **58** was synthesized according to the general procedure J, starting from **36** and 4-aminobenzoic acid. FC in ethyl acetate/methanol 9.8/0.2, *R_f_*: 0.55. Yellow oil (62% yield). ^1^H NMR (400 MHz, CD_3_OD): δ: 2.85 (t, 2H, C*H*_2_, *J* = 8.3 Hz); 3.21 (t, 2H, C*H*_2_, *J* = 8.3 Hz); 4.15 (s, 2H, *CH*_2_); 6.43 (d, 2H, aryl, *J* = 8.3 Hz); 6.87 (d, 1H, aryl, *J* = 10.2 Hz); 6.96 (d, 2H, aryl, *J* = 8.8 Hz); 7.26–7.30 (m, 2H, aryl, *J* = 7.2 Hz); 7.47 (d, 2H, aryl, *J* = 8.6 Hz); 7.85 (d, 2H, aryl, *J* = 8.6 Hz). ^13^C NMR (100 MHz, CD_3_OD): δ: 27.8, 52.4, 53.3, 106.7, 114.6, 114.8, 122.3, 122.8, 124.7, 127.3, 129.4, 129.9, 143.5, 151.2, 160.9, 163.1, 180.4. ESI-MS **m*/*z** calcd for C_23_H_20_FN_3_O_2_S [(M + H)]^+^: 421.1260; found 421.1253.

##### (4-Fluorophenyl)(5-nitroindolin-1-yl)methanone (**59**)

Intermediate **59** was synthesized starting from 5-nitroindoline and 4-fluorobenzoyl chloride following procedure B. FC in hexane/ethyl acetate 8/2, *R_f_*: 0.47. Yellow oil (91% yield). ^1^H NMR (400 MHz, CD_3_OD): δ: 3.16 (t, 2H, C*H*_2_, *J* = 8.3 Hz); 4.12 (t, 2H, C*H*_2_, *J* = 8.4 Hz); 7.11 (t, 2H, aryl, *J* = 8.6 Hz); 7.20 (s, 1H, aryl); 7.52–7.56 (m, 2H, aryl); 8.02–8.04 (m, 2H, aryl). ESI-MS **m*/*z** calcd for C_15_H_11_FN_2_O_3_ [(M + H)]^+^: 287.0826; found 287.0830.

##### 2-(4-Fluorophenyl)-1-(5-nitroindolin-1-yl)ethanone (Intermediate Nitroindoline CO-4-benzyl) (**60**)

Intermediate **60** was synthesized starting from 5-nitroindoline and 4-fluorophenylacetyl chloride following procedure B. FC in hexane/ethyl acetate 8/2, *R_f_*: 0.47. Yellow oil (88% yield). ^1^H NMR (400 MHz, CD_3_OD): δ: 3.31 (t, 2H, C*H*_2_, *J* = 6.8 Hz); 3.84 (s, 2H, C*H*_2_); 4.25 (t, 2H, C*H*_2_, *J* = 7.0 Hz); 7.08 (t, 2H, aryl, *J* = 6.9 Hz); 7.29 (t, 2H, aryl, *J* = 5.0 Hz); 8.05 (s, 1H, aryl); 8.14 (d, 1H, aryl, *J* = 8.9 Hz); 8.34 (d, 1H, aryl, *J* = 7.0 Hz). ESI-MS **m*/*z** calcd for C_16_H_13_FN_2_O_3_ [(M + H)]^+^: 301.0983; found 301.0977.

##### *tert*-Butyl 5-Nitroindoline-1-carboxylate (**61**)

Intermediate **61** was synthesized starting from 5-nitroindoline and di-*tert*-butyl dicarbonate following procedure B. FC in hexane/ethyl acetate 8/2, *R_f_*: 0.45. Yellow solid (88% yield). ^1^H NMR (400 MHz, CDCl_3_): δ: 1.60 (s, 9H, C*H*_3_); 3.19 (t, 2H, C*H*_2_, *J* = 8.7 Hz); 4.11 (t, 2H, C*H*_2_, *J* = 9.0 Hz); 7.28 (d, 1H, aryl, *J* = 7.7 Hz); 8.02 (s, 1H, aryl); 8.12 (d, 1H, aryl, *J* = 11.0 Hz). ESI-MS **m*/*z** calcd for C_13_H_16_N_2_O_4_ [(M + H)]^+^: 265.1183; found 265.1190.

##### (5-Aminoindolin-1-yl)(4-fluorophenyl)methanone (**62**)

Obtained from intermediate **59** following the general procedure H. FC in hexane/ethyl acetate 1/1, *R_f_*: 0.49. Yellow oil (87% yield). ^1^H NMR (400 MHz, CDCl_3_): δ: 3.05 (t, 2H, C*H*_2_, *J* = 8.1 Hz); 3.61 (bs, 2H, N*H*_2_); 4.02 (bs, 2H, C*H*_2_); 6.59 (bs, 3H, aryl); 7.13 (t, 2H, aryl, *J* = 8.7 Hz); 7.58 (t, 2H, aryl, *J* = 5.5 Hz). ESI-MS **m*/*z** calcd for C_15_H_13_FN_2_O [(M + H)]^+^: 257.1085; found 257.1081.

##### 1-(5-Aminoindolin-1-yl)-2-(4-fluorophenyl)ethanone (**63**)

Obtained from intermediate **60** following the general procedure H. FC in hexane/ethyl acetate 1/1, *R_f_*: 0.47. Yellow oil (85% yield). ^1^H NMR (400 MHz, CD_3_OD): δ: 3.11 (t, 2H, C*H*_2_, *J* = 6.6 Hz); 3.84 (s, 2H, C*H*_2_); 4.12 (t, 2H, C*H*_2_, *J* = 6.7 Hz); 6.57 (d, 1H, aryl, *J* = 6.8 Hz); 6.66 (s, 1H, aryl); 7.09 (t, 2H, aryl, *J* = 7.0 Hz); 7.34 (t, 2H, aryl, *J* = 4.4 Hz); 7.90 (d, 1H, aryl, *J* = 6.8 Hz). ESI-MS **m*/*z** calcd for C_16_H_15_FN_2_O [(M + H)]^+^: 271.1241; found 271.1235.

##### *tert*-Butyl 5-Aminoindoline-1-carboxylate (**64**)

Obtained from intermediate **61** following the general procedure H. FC in hexane/ethyl acetate 1/1, *R_f_*: 0.48. White solid (85% yield). ^1^H NMR (400 MHz, CDCl_3_): δ: 1.53 (s, 9H, C*H*_3_); 2.96 (t, 2H, C*H*_2_, *J* = 7.2 Hz); 3.89 (bs, 2H, C*H*_2_); 6.46–6.48 (m, 3H, aryl). ESI-MS **m*/*z** calcd for C_13_H_18_N_2_O_2_ [(M + H)]^+^: 235.1441; found 235.1447.

##### (4-Fluorophenyl)(5-isothiocyanatoindolin-1-yl)methanone (**65**)

Obtained from intermediate **62** following the general procedure I. FC in hexane/ethyl acetate 9/1, *R_f_*: 0.52. Yellow oil (58% yield). ^1^H NMR (400 MHz, CDCl_3_): δ: 3.05 (t, 2H, C*H*_2_, *J* = 8.3 Hz); 4.03 (t, 2H, C*H*_2_, *J* = 8.3 Hz); 6.96–7.02 (m, 3H, aryl); 7.08 (t, 2H, aryl, *J* = 8.6 Hz); 7.48–7.52 (m, 2H, aryl). ESI-MS **m*/*z** calcd for C_16_H_11_FN_2_OS [(M + H)]^+^: 299.0649; found 299.0653.

##### 2-(4-Fluorophenyl)-1-(5-isothiocyanatoindolin-1-yl)ethanone (**66**)

Obtained from intermediate **63** following the general procedure I. FC in hexane/ethyl acetate 9/1, *R_f_*: 0.44. Yellow oil (62% yield). ^1^H NMR (400 MHz, DMSO): δ: 3.17 (t, 2H, C*H*_2_, *J* = 7.0 Hz); 3.89 (s, 2H, C*H*_2_); 4.21 (t, 2H, C*H*_2_, *J* = 6.8 Hz); 7.02 (d, 1H, aryl, *J* = 6.4 Hz); 7.14–7.18 (m, 2H, aryl); 7.32 (bs, 2H, aryl); 7.39 (s, 1H, aryl); 7.93 (d, 1H, aryl, *J* = 7.0 Hz). ESI-MS **m*/*z** calcd for C_17_H_13_FN_2_OS [(M + H)]^+^: 312.0727; found 312.0734.

##### *tert*-Butyl 5-Isothiocyanatoindoline-1-carboxylate (**67**)

Obtained from intermediate **64** following the general procedure I. FC in hexane/ethyl acetate 8/2, *R_f_*: 0.47. Yellow oil (58% yield). ^1^H NMR (400 MHz, CDCl_3_): δ: 1.58 (s, 9H, C*H*_3_); 3.09 (t, 2H, C*H*_2_, *J* = 8.7 Hz); 4.02 (t, 2H, C*H*_2_, *J* = 8.7 Hz); 6.92–6.96 (m, 3H, aryl). ESI-MS **m*/*z** calcd for C_14_H_16_N_2_O_2_S [(M + H)]^+^: 277.1005; found 277.1001.

##### 1-(1-(4-Fluorobenzoyl)indolin-5-yl)-3-neopentylthiourea (**68**)

Derivative **68** was synthesized according to the general procedure J, starting from **65** and 2,2-dimethylpropan-1-amine. FC in dichloromethane/ethyl acetate 8/2, *R_f_*: 0.48. Yellow oil (65% yield). ^1^H NMR (400 MHz, CDCl_3_): δ: 0.91 (s, 9H, C*H*_3_); 3.15 (t, 2H, C*H*_2_, *J* = 8.2 Hz); 3.47 (bs, 2H, *CH*_2_); 4.12 (t, 2H, C*H*_2_, *J* = 8.1 Hz); 7.04 (bs, 1H, N*H*); 7.13–7.17 (m, 4H, aryl); 7.57–7.61 (m, 3H, aryl). ^13^C NMR (100 MHz, CDCl_3_) δ 27.5, 28.2, 51.1, 56.5, 115.7, 115.9, 122.4, 124.6, 129.6, 132.0, 132.5, 134.6, 141.8, 162.7, 165.2, 168.1, 181.0. ESI-MS **m*/*z** calcd for C_21_H_24_FN_3_OS [(M + H)]^+^: 386.1697; found 386.1703.

##### 1-(1-(2-(4-Fluorophenyl)acetyl)indolin-5-yl)-3-neopentylthiourea (**69**)

Derivative **69** was synthesized according to the general procedure J, starting from **66** and 2,2-dimethylpropan-1-amine FC in hexane/ethyl acetate 7/3, *R_f_*: 0.47. Yellow oil (58% yield). ^1^H NMR (400 MHz, CD_3_OD): δ: 0.96 (s, 9H, *CH*_3_); 3.21 (t, 2H, C*H*_2_, *J* = 8.5 Hz); 3.47 (bs, 2H, C*H*_2_); 3.88 (s, 2H, *CH*_2_); 4.21 (t, 2H, C*H*_2_, *J* = 8.5 Hz); 7.06–7.10 (m, 3H, aryl); 7.32–7.35 (m, 3H, aryl); 8.11 (d, 1H, aryl, *J* = 8.6 Hz). ^13^C NMR (100 MHz, CD_3_OD) δ 26.4, 27.4, 41.3, 55.3, 114.7, 115.0, 116.8, 130.5, 130.9, 140.6, 160.8, 163.2, 170.1. ESI-MS **m*/*z** calcd for C_22_H_26_FN_3_OS [(M + H)]^+^: 400.1853; found 400.1859.

##### *tert*-Butyl 5-(3-Neopentylthioureido)indoline-1-carboxylate (**70**)

Intermediate **70** was synthesized according to the general procedure J, starting from **67** and 2,2-dimethylpropan-1-amine FC in hexane/ethyl acetate 7/3, *R_f_*: 0.47. Yellow oil (52% yield). ^1^H NMR (400 MHz, CDCl_3_): δ: 0.82 (s, 9H, C*H*_3_); 0.87 (s, 9H, C*H*_3_); 3.03 (t, 2H, C*H*_2_, *J* = 8.7 Hz); 3.95 (t, 2H, C*H*_2_, *J* = 8.7 Hz); 7.03–7.06 (m, 3H, aryl); 7.46 (bs, 1H, N*H*). ESI-MS **m*/*z** calcd for C_19_H_29_N_3_O_2_S [(M + H)]^+^: 364.2053; found 364.2060.

##### 1-(Indolin-5-yl)-3-neopentylthiourea (**71**)

Obtained from intermediate **70** following the general procedure D. FC in hexane/ethyl acetate 1/1, *R_f_*: 0.47. White oil (95% yield). ^1^H NMR (400 MHz, CDCl_3_): δ: 0.91 (s, 9H, C*H*_3_); 3.04 (t, 2H, C*H*_2_, *J* = 8.7 Hz); 3.55 (t, 2H, C*H*_2_, *J* = 8.3 Hz); 6.72 (d, 1H, aryl, *J* = 8.2 Hz); 6.92 (d, 1H, aryl, *J* = 10.2 Hz); 7.06 (s, 1H, aryl). ESI-MS **m*/*z** calcd for C_14_H_21_N_3_S [(M + H)]^+^: 264.1529; found, 264.1538.

##### 1-Neopentyl-3-(1-(4-nitrobenzyl)indolin-5-yl)thiourea (**72**)

Intermediate **72** was synthesized according to the general procedure G, starting from **71** and 4-nitrobenzaldehyde. FC in hexane/ethyl acetate 7/3, *R_f_*: 0.48. Yellowish oil (68% yield). ^1^H NMR (400 MHz, CDCl_3_): δ: 0.92 (s, 9H, C*H*_3_); 3.08 (t, 2H, C*H*_2_, *J* = 6.7 Hz); 3.50 (t, 2H, C*H*_2_, *J* = 6.7 Hz); 4.41 (s, 2H, C*H*_2_); 6.45 (d, 1H, aryl, *J* = 6.6 Hz); 6.95 (d, 1H, aryl, *J* = 6.5 Hz); 7.06 (s, 1H, aryl); 7.57 (d, 2H, aryl, *J* = 6.8 Hz); 8.25 (d, 2H, aryl, *J* = 7.0 Hz). ESI-MS **m*/*z** calcd for C_21_H_26_N_4_O_2_S [(M + H)]^+^: 399.1849; found 399.1842.

##### Synthesis of 1-Neopentyl-3-(1-(4-nitrosobenzyl)indolin-5-yl)thiourea (**73**)

**72** (0.05 mmol) was solubilized in MeOH, and 0.25 mmol of zinc dust and 0.25 mmol of ammonium chloride were introduced and the solution was refluxed for 1 h. Then, the mixture was cooled to room temperature and filtered over celite; the methanolic phase was evaporated, and the residue was washed with 10% w-w aqueous solution of NaHCO_3_, dried over Na_2_SO_4_, filtered, and concentrated *in vacuo*. The final derivative **73** was isolated after flash chromatography using 9/1 dichloromethane/ethyl acetate as eluent. FC in dichloromethane/ethyl acetate 9/1, *R_f_*: 0.49. Yellowish solid (64% yield). ^1^H NMR (400 MHz, CD_3_OD): δ: 0.91 (s, 9H, C*H*_3_); 2.91 (t, 2H, C*H*_2_, *J* = 8.3 Hz); 3.28 (t, 2H, C*H*_2_, *J* = 8.3 Hz); 3.44 (bs, 2H, C*H*_2_); 4.14 (s, 2H, C*H*_2_); 6.58 (d, 1H, aryl, *J* = 8.3 Hz); 6.73 (d, 2H, aryl, *J* = 8.4 Hz); 6.90 (d, 1H, aryl, *J* = 8.3 Hz); 6.95 (s, 1H, aryl); 7.11 (d, 2H, aryl, *J* = 8.3 Hz). ^13^C NMR (100 MHz, CD_3_OD): δ: 26.4, 27.7, 32.0, 52.5, 52.9, 55.4, 107.0, 115.5, 115.7, 122.7, 125.3, 127.6, 128.9, 129.9, 131.6, 146.0, 151.7, 181.6. ESI-MS **m*/*z** calcd for C_21_H_26_N_4_OS [(M + H)]^+^: 383.1900; found 383.1905.

##### 1-(4-Fluorobenzyl)-5-nitro-1*H*-indole (**74**)

Intermediate **74** was synthesized starting from 5-nitroindole and 4-fluorobenzyl chloride following procedure C. FC in hexane/ethyl acetate 8/2, *R_f_*: 0.47. Yellow oil (85% yield). ^1^H NMR (400 MHz, CDCl_3_): δ: 5.35 (s, 2H, C*H*_2_); 6.74 (d, 1H, aryl, *J* = 3.1 Hz); 7.02 (t, 2H, aryl, *J* = 8.1 Hz); 7.08–7.12 (m, 2H, aryl); 7.28–7.31 (m, 2H, aryl); 8.03 (s, 1H, aryl); 8.08 (d, 1H, aryl, *J* = 9.1 Hz); 8.60 (s, 1H, N*H*). ESI-MS **m*/*z** calcd for C_15_H_11_FN_2_O_2_ [(M + H)]^+^: 271.0877; found 271.0884.

##### 1-(4-Fluorobenzyl)-1*H*-indol-5-amine (**75**)

Intermediate **75** was synthesized starting from **74** following procedure H. FC in hexane/ethyl acetate 7/3, *R_f_*: 0.52. Yellow oil (82% yield). ^1^H NMR (400 MHz, CDCl_3_): δ: 3.46 (bs, 2H, N*H*_2_); 5.24 (s, 2H, C*H*_2_); 6.40 (d, 1H, aryl, *J* = 3.0 Hz); 6.67 (dd, 1H, aryl, *J*′ = 2.2, Hz *J*″ = 6.4 Hz); 6.98–7.02 (m, 3H, aryl); 7.06–7.11 (m, 4H, aryl). ESI-MS **m*/*z** calcd for C_15_H_13_N_2_ [(M + H)]^+^: 241.1136; found 241.1129.

##### 1-(4-Fluorobenzyl)-5-isothiocyanato-1*H*-indole (**76**)

Intermediate **76** was obtained following the general procedure I, starting from **75**. FC in hexane/ethyl acetate 9/1, *R_f_*: 0.47. Yellow oil (64% yield). ^1^H NMR (400 MHz, CDCl_3_): δ: 5.13 (s, 2H, C*H*_2_); 6.29 (d, 1H, aryl, *J* = 2.6 Hz); 6.51 (dd, 1H, aryl, *J*′ = 2.1, Hz *J*″ = 8.7 Hz); 6.81 (d, 1H, aryl, *J* = 1.9 Hz); 6.87–7.01 (m, 5H, aryl); 7.19 (s, 1H, aryl). ESI-MS **m*/*z** calcd for C_16_H_11_FN_2_S [(M + H)]^+^: 283.0700; found 283.0702.

##### 1-(1-(4-Fluorobenzyl)-1*H*-indol-5-yl)-3-neopentylthiourea (**77**)

Derivative **77** was obtained following general procedure J, starting from **76**, which was reacted with 2,2-dimethylpropan-1-amine. FC in hexane/ethyl acetate 7/3, *R_f_*: 0.48. Yellow oil (76% yield). ^1^H NMR (400 MHz, CDCl_3_): δ: 0.77 (s, 9H, C*H*_3_); 3.40 (d, 2H, C*H*_2_, *J* = 5.5 Hz); 5.23 (s, 2H, C*H*_2_); 5.93 (bs, 1H, N*H*); 6.50 (d, 1H, aryl, *J* = 3.1 Hz); 6.91–6.96 (m, 2H, aryl); 7.01 (t, 2H, aryl, *J* = 5.3 Hz); 7.14 (d, 1H, aryl, *J* = 3.1 Hz); 7.21 (t, 2H, aryl, *J* = 9.3 Hz); 7.43 (s, 1H, aryl); 7.62 (bs, 1H, N*H*). ^13^C NMR (100 MHz, CDCl_3_): δ: 27.4, 49.8, 56.6, 102.3, 111.1, 115.7, 116.0, 119.1, 120.4, 127.7, 128.4, 129.6, 130.0, 132.6, 135.3, 161.2, 163.6, 181.8. ESI-MS **m*/*z** calcd for C_21_H_24_FN_3_S [(M + H)]^+^: 370.1748; found 370.1752.

##### 9-(4-Fluorobenzyl)-9*H*-carbazole (**78**)

Intermediate **78** was obtained following general procedure C, starting from carbazole, which was reacted with 4-fluorobenzyl chloride. FC in hexane/ethyl acetate 7/3, *R_f_*: 0.47. Yellow oil (97% yield). ^1^H NMR (400 MHz, CDCl_3_): δ: 5.30 (s, 2H, C*H*_2_); 6.80 (t, 2H, aryl, *J* = 9.0 Hz); 6.94–6.98 (m, 2H, aryl); 7.15 (t, 2H, aryl, *J* = 8.0 Hz); 7.20 (d, 2H, aryl, *J* = 8.4 Hz); 7.32 (t, 2H, aryl, *J* = 8.8 Hz); 8.02 (d, 2H, aryl, *J* = 8.2 Hz). ESI-MS **m*/*z** calcd for C_19_H_14_FN_2_ [(M + H)]^+^: 276.1183; found 276.1177.

##### Synthesis of 9-(4-Fluorobenzyl)-3-nitro-9*H*-carbazole (**79**)

Intermediate **78** (0.1 mmol) was solubilized in acetic anhydride and cooled to 0 °C. To this solution, 0.05 mmol of nitric acid was added and the solution was stirred for a further 2 h. Then, the mixture was quenched with a 2 M NaOH solution and extracted with dichloromethane. The organic layer was dried over anhydrous Na_2_SO_4_, filtered, and concentrated *in vacuo*. Purification by flash chromatography using dichloromethane/hexane 8/2 as a mobile phase afforded intermediate **79**.

FC in dichloromethane/hexane 8/2, *R_f_*: 0.47. Yellow solid (77% yield). ^1^H NMR (400 MHz, CDCl_3_): δ: 5.47 (s, 2H, C*H*_2_); 6.91 (t, 2H, aryl, *J* = 8.6 Hz); 7.01–7.05 (m, 2H, aryl); 7.16–7.20 (m, 2H, aryl); 7.29–7.36 (m, 2H, aryl); 7.47 (t, 1H, aryl, *J* = 8.2 Hz); 8.12 (d, 1H, aryl, *J* = 7.8 Hz); 8.29 (d, 1H, aryl, *J* = 8.8 Hz); 8.99 (s, 1H, aryl). ESI-MS **m*/*z** calcd for C_19_H_14_FN_2_O_2_ [(M + H)]^+^: 321.1034; found 321.1040.

##### 9-(4-Fluorobenzyl)-9*H*-carbazol-3-amine (**80**)

Intermediate **80** was obtained following general procedure H, starting from **79**. FC in hexane/ethyl acetate 2/8, *R_f_*: 0.40. Yellowish solid (90% yield). ^1^H NMR (400 MHz, CDCl_3_): δ: 3.48 (bs, 2H, N*H*_2_); 5.31 (s, 2H, C*H*_2_); 6.78–6.86 (m, 3H, aryl); 6.97–7.00 (m, 2H, aryl); 7.04 (d, 1H, aryl, *J* = 8.5 Hz); 7.10 (t, 1H, aryl, *J* = 7.8 Hz); 7.19 (d, 1H, aryl, *J* = 8.2 Hz); 7.30 (t, 1H, aryl, *J* = 7.1 Hz); 7.37 (s, 1H, aryl); 7.93 (d, 1H, aryl, *J* = 7.8 Hz). ESI-MS **m*/*z** calcd for C_19_H_15_FN_2_ [(M + H)]^+^: 291.1292; found 291.1300.

##### 9-(4-Fluorobenzyl)-3-isothiocyanato-9*H*-carbazole (**81**)

Intermediate **81** was obtained following the general procedure I, starting from **80**. FC in hexane/ethyl acetate 9/1, *R_f_*: 0.47. Yellowish oil (55% yield). ^1^H NMR (400 MHz, CDCl_3_): δ: 5.49 (s, 2H, C*H*_2_); 6.96 (t, 2H, aryl, *J* = 8.6 Hz); 7.08–7.12 (m, 2H, aryl); 7.27–7.34 (m, 2H, aryl); 7.04 (d, 1H, aryl, *J* = 8.2 Hz); 7.38 (d, 1H, aryl, *J* = 8.2 Hz); 7.51 (t, 1H, aryl, *J* = 8.2 Hz); 8.00 (s, 1H, aryl); 8.09 (d, 1H, aryl, *J* = 7.8 Hz). ESI-MS **m*/*z** calcd for C_20_H_13_FN_2_S [(M + H)]^+^: 333.0856; found 333.0864.

##### 1-(9-(4-Fluorobenzyl)-9*H*-carbazol-3-yl)-3-neopentylthiourea (**82**)

Derivative **82** was synthesized starting from **81** and 2,2-dimethylpropan-1-amine following procedure J. FC in dichloromethane/ethyl acetate 9.5/0.5, *R_f_*: 0.52. Yellowish oil (58% yield). ^1^H NMR (400 MHz, CD_3_OD): δ: 0.94 (s, 9H, C*H*_3_); 3.48 (bs, 2H, C*H*_2_); 5.59 (s, 2H, C*H*_2_); 6.97 (t, 2H, aryl, *J* = 8.7 Hz); 7.15–7.18 (m, 2H, aryl); 7.24 (t, 1H, aryl, *J* = 7.8 Hz); 7.34 (d, 1H, aryl, *J* = 8.1 Hz); 7.43–7.52 (m, 3H, aryl); 8.07–8.11 (m, 2H, aryl). ^13^C NMR (100 MHz, CD_3_OD): δ: 26.4, 45.2, 55.4, 109.0, 109.5, 114.8, 115.1, 117.7, 119.2, 120.0, 122.5, 123.4, 124.0, 126.1, 128.2, 133.4, 139.0, 141.2, 160.9, 163.3, 182.0. ESI-MS **m*/*z** calcd for C_25_H_26_FN_3_S [(M + H)]^+^: 420.1904; found 420.1911.

### Computational Details

The ligand geometries were built through the Build Panel of Maestro (version 11) as follows: OPLS3 force field,^51^ the Polak–Ribier conjugate gradient algorithm (PRCG, 9 × 10^7^ steps, maximum derivative less than 0.001 kcal/mol), using a GB/SA (generalized Born/surface area) solvent treatment^52^ to mimic the presence of H_2_O. The so-obtained structures were processed with LigPrep,^53^ generating all possible tautomers, stereoisomers, and protonation states at a pH of 7.0 ± 1.0. The structures of 5-LOX (PDB ID: 3O8Y)^54^ and sEH (PDB ID: 3I28)^44^ for the docking calculations were processed by Protein Preparation Wizard^55,56^ in detail: ligand, buffer ions, and water molecules were deleted; all hydrogens were added and bond order was assigned, missing side chains and loops were checked; residue alternate positions were checked considering the A conformation; the side-chain charges were assigned considering their p*K*_a_ at a physiological pH of 7.4.

For binding investigation toward 5-LOX, the Induced Fit Docking^57−59^ was applied, using the extended protocol, generating 80 ligand–protein poses at XP precision. The grid was centered on Fe^2+^ with an inner box of 10 Å, and the outer one was automatically generated. The conformational search was performed allowing the sample ring conformations of the small molecules, with an energy window of 2.5 kcal/mol. For Prime refinement, the default values were used. For docking calculations on sEH, Glide software^60,61^ was employed to dock the ligand against sEH. To validate the docking methodology, the cocrystallized ligand 34N with sEH was docked and the obtained conformation was compared with the experimental one (RMSD = 0.486 Å).^62−64^ The inner and outer receptor grid boxes of 10 Å and 17 Å, respectively, centered on the *x*, *y*, and *z* coordinates: 75.42, −9.30, and 68.12. In the first step, Standard Precision (SP) was applied along with default parameters, producing one pose per ligand. These poses from the SP calculations were utilized as input conformations for three rounds of predictions in the Extra Precision (XP) Glide mode: flexible ligand; only amide bond trans conformation allowed; nitrogen inversion and ring conformations (with an energy cutoff of 2.5 kcal/mol) sampling. The enhanced sampling mode was utilized, saving 10,000 poses/ligand for the initial docking step and 1000 poses/ligand for energy minimization. One thousand maximum output structures/ligands were kept applying 0.8 as the scaling factor for van der Waals radii and 0.15 as the partial charge cutoff. Postdocking optimization was executed on docked poses, filtering through 100 maximum number of poses and 0.5 kcal/mol cutoff to reject the obtained minimized conformations. The energy contributions of the Epik state penalty, aromatic bonds, and intramolecular H-bond reward were considered in the predictions. The docking outcome analysis and figure preparation were carried out by Maestro (version 11).

### Cell-Free 5-LOX Activity Assay

Human recombinant 5-LOX was expressed in *Escherichia coli* Bl21 (DE3) cells that were transformed with pT3–5LO (wt 5-LOX) plasmid and purified on an ATP-agarose column (Econo-Pac, Bio-Rad, Hercules, CA) by affinity chromatography.^65,66^ In a first step, *E. coli* was lysed in 50 mM triethanolamine/HCl pH 8.0 with EDTA (5 mM), soybean trypsin inhibitor (60 μg/mL), phenylmethylsulfonyl fluoride (1 mM), dithiothreitol (1 mM), and lysozyme (1 mg/mL) and sonified 3 times for 15 s. The homogenate was centrifuged at 40,000*g* for 20 min at 4 °C. The supernatant was transferred to an ATP-agarose column (Sigma-Aldrich, Deisenhofen, Germany), which was sequentially washed with phosphate-buffered saline (PBS) pH 7.4, 1 mM EDTA, 50 mM phosphate buffer pH 7.4, 0.5 M NaCl, 1 mM EDTA, and finally 50 mM phosphate buffer pH 7.4 plus 1 mM EDTA. The enzyme was eluted with 50 mM phosphate buffer pH 7.4, 1 mM EDTA, and 20 mM ATP.

The purified 5-LOX (0.5 μg in PBS pH 7.4 containing 1 mM EDTA) was preincubated with vehicle (DMSO) or test compounds for 15 min on ice. 5-LOX product formation was started by the addition of arachidonic acid (Sigma-Aldrich; 20 μM) and CaCl_2_ (2 mM) and stopped by an equal volume of ice-cold methanol containing PGB_1_ (200 ng) as the internal standard after 10 min at 37 °C. Major 5-LOX metabolites (*all-trans* isomers of LTB_4_ and 5-HETE) were extracted on Sep-Pak C18 35 cc Vac Cartridges (Waters, Milford, MA), separated by reversed-phase HPLC (RP-HPLC) on a Nova-Pak C18 Radial-Pak Column (4 μm, 5 mm × 100 mm, Waters) under isocratic conditions (73% methanol/27% water/0.007% trifluoroacetic acid) at a flow rate of 1.2 mL/min and detected at 235 and 280 nm.^65^

### 5-LOX Product Formation by Human PMNL

Human PMNLs were freshly isolated from leukocyte concentrates that were obtained from the Institute for Transfusion Medicine of the University Hospital Jena (Germany). Thus, venous blood was collected in heparinized tubes (16 U heparin/mL blood), with informed consent of registered male and female healthy adult volunteers (18–65 years) who fasted for at least 12 h. These volunteers regularly donated blood (every 8–12 weeks) and were physically inspected by a clinician. They had not taken antibiotics or anti-inflammatory drugs for more than 10 days before blood donation and were free of apparent infections, inflammatory disorders, or acute allergic reactions. Leukocytes were concentrated by centrifugation (4000*g*/20 min/20 °C) of freshly withdrawn blood and subjected to density gradient centrifugation on a lymphocyte separation medium (LSM 1077, GE Healthcare, Freiburg, Germany). Erythrocytes were removed by dextran sedimentation and hypotonic lysis. PMNLs were obtained from the cell pellet. Freshly isolated PMNLs (5 × 10^6^) suspended in PBS pH 7.4 with 1 mg/mL glucose were preincubated with the test compounds for 15 min on ice. 5-LOX product formation in PMNL was triggered by the addition of Ca^2+^-ionophore A23187 (2.5 μM; Sigma-Aldrich) followed by incubation for 10 min at 37 °C. The reaction was stopped with an equal volume of methanol containing PGB_1_ (200 ng) as the internal standard. Major 5-LOX metabolites (*all-trans* isomers of LTB and 5-HETE) and, for PMNL, additionally LTB_4_ were extracted and analyzed by RP-HPLC, as described for the determination of cell-free 5-LOX activity.

### sEH Activity

Human recombinant sEH was expressed in Sf9 insect cells and purified by affinity chromatography. Briefly, Sf9 cells were infected with a recombinant baculovirus and lysed after 72 h in 50 mM NaHPO_4_ pH 8, 300 mM NaCl, 10% glycerol, 1 mM EDTA, 1 mM phenylmethanesulfonyl fluoride, 10 μg/mL leupeptin, and 60 μg/mL STI by sonication (3 × 10 s, 4 °C). Sequential centrifugation at 20,000*g* (10 min, 4 °C) and 100,000*g* (60 min, 4 °C) yielded a supernatant, which was subjected to a benzylthio-sepharose affinity chromatography. Elution with 4-fluorochalcone oxide in PBS pH 7.4 with 1 mM dithiothreitol and 1 mM EDTA yielded sEH, which was dialyzed and concentrated. The purified sEH (60 ng) in 25 mM tris–HCl pH 7 with 0.1 mg/mL bovine serum albumin (BSA) was preincubated with the vehicle (DMSO) or test compounds for 1 min at room temperature. The sEH substrate PHOME (20 μM, Cayman Chemicals) was added to start the enzymatic reaction, which was stopped after 60 min in the darkness by the addition of ZnSO_4_ (200 mM). The formation of the fluorescent product 6-methoxynaphthaldehyde was measured using a NOVOstar fluorescence microplate reader (BMG Labtech, Ortenberg, Germany), with excitation at 330 and emission at 465 nm.

### Cell Culture

A murine monocyte/macrophage J774 cell line was obtained from the American Type Culture Collection (ATTC TIB 67). The cell line was grown in adhesion in Dulbecco’s modified Eagle’s medium (DMEM) supplemented with glutamine (2 mM, Aurogene Rome, Italy), Hepes (25 mM, Aurogene Rome, Italy), penicillin (100 U/mL, Aurogene Rome, Italy), streptomycin (100 μg/mL, Aurogene Rome, Italy), fetal bovine serum (FBS, 10%, Aurogene Rome, Italy), and sodium pyruvate (1.2%, Aurogene Rome, Italy) (DMEM completed). The cells were plated at a density of 1 × 10^6^ cells in 75 cm^2^ culture flasks and maintained at 37 °C under 5% CO_2_ in a humidified incubator until 90% confluence. The culture medium was changed every 2 days. Before a confluent monolayer appeared, the subculturing cell process was carried out.

### Assessment of COX-1 Activity

Cells (0.5 × 10^6^ cells/mL) were pretreated with **73** (0,1–10 μM), indomethacin (10 μM), or celecoxib (10 μM) for 2 h and further incubated for 30 min with AA (15 μM).^46,67^ At the end of the incubation, the supernatants were collected for the measurement of PGE_2_ levels with commercially available ELISA kits according to the manufacturer’s instructions (R&D Systems, Aurogene, Rome, Italy).

### Assessment of COX-2 Activity

Cells (0.5 × 10^6^ cells/mL) were stimulated, for 24 h, with lipopolysaccharide (LPS) from *E. coli*, serotype 0111:B4 (10 μg/mL; 100 μL in DMEM completed with FBS, Sigma-Aldrich, Milan, Italy), to induce COX-2, then pretreated for 2 h with test compound (0–10 μM), indomethacin (10 μM), or celecoxib (10 μM) and further incubated for 30 min with AA (15 μM). In another set of experiments, cells were pretreated for 2 h in the absence or presence of test compound and then stimulated for 24 h with LPS (10 μg/mL). The supernatants were collected for the measurement of PGE_2_ levels by ELISA assay (R&D Systems, Aurogene, Rome, Italy).

### Western Blot Analysis

The analysis of COX-2 in J774 macrophages was performed on whole cell lysates. After stimulation with LPS for 24 h, cells were washed with cold PBS, collected by scraping, and centrifuged at 8000 rpm for 5 min at 4 °C. Pellets were lysed by syringing with RIPA buffer (Trizma Base, NaCl, 100 mM EDTA, 10% Na-deoxycholate, and 10% Nonidet P-40), completed with 200 mM of activated orthovanadate and complete protease inhibitor cocktail (Sigma-Aldrich), and centrifuged at 12,000 rpm for 10 min at 4 °C. The supernatants were collected, and protein concentration in cell lysates was determined by Bio-Rad Protein Assay (Bio-Rad). Equal amounts of protein (50 μg) were mixed with gel loading buffer (50 mM tris, 10% sodium dodecyl sulfate (SDS), 10% glycerol, 10% 2-mercaptoethanol, and 2 mg/mL of bromophenol) in a ratio of 4:1, boiled for 5 min. Each sample was loaded and electrophoresed on a 10% SDS–polyacrylamide gel. The proteins were transferred onto nitrocellulose membranes (0.2 μm nitrocellulose membrane, Trans-Blot TurboTM, Transfer Pack, Bio-Rad Laboratories). The membranes were blocked with 0.1% PBS-Tween containing 5% nonfat dry milk. After blocking, the membranes were incubated with the relative primary antibody overnight at 4 °C. Mouse monoclonal antibody anti-COX-2 (BD Transduction Laboratories) was diluted to 1:1000 in 0.1% PBS-Tween, 5% nonfat dry milk; mouse monoclonal antibody anti-β-actin (Santa Cruz Biotechnology) was diluted to 1:1000 in 0.1% PBS-Tween, 5% nonfat dry milk. After the incubation, the membranes were washed three times with 0.1% PBS-Tween and were incubated for 2 h at room temperature with horseradish peroxidase-conjugated antimouse secondary antibody (Santa Cruz Biotechnology) diluted to 1:2000 in 0.1% PBS-Tween containing 5% nonfat dry milk. The membranes were washed, and protein bands were detected by an enhanced chemiluminescence system (ChemiDoc, Bio-Rad). Densitometric analysis was performed with Image Lab software (Bio-Rad Laboratories).

### Cell Viability

Cell respiration, an indicator of cell viability, was assessed by the mitochondrial-dependent reduction of 3-(4,5-dimethylthiazol-2-yl)-2,5-diphenyltetrazolium bromide (MTT; Sigma-Aldrich, Milan, Italy) to formazan. Cells were plated to a seeding density of 1.0 × 10^5^ in 96-multiwell. After stimulation with test compound for 24 h, cells were incubated in 96-well plates with MTT (0.2 mg/mL) for 1 h. Culture medium was removed by aspiration, and the cells were lysed in DMSO (0.1 mL). The extent of reduction of MTT to formazan within cells was quantified by the measurement of OD_550_.

### Animals

Male CD-1 mice (33–39 g, 8 weeks, Charles River Laboratories; Calco, Italy) and female BALB/c mice (20 g, 8 weeks, Charles River Laboratories) were fed with standard rodent chow and water and acclimated for 4 days at a 12 h light and 12 h dark schedule in a constant air-conditioned environment (21 ± 2 °C). Mice were randomly assigned to groups, and experiments were carried out during the light phase. Experimental procedures were conducted in conformity with Italian (D.L. 26/2014) and European (directive 2010/63/EU) regulations on the protection of animals used for scientific purposes and approved by the Italian Ministry.

### *In Vivo* Plasma Levels of **73**

For the analysis of plasma levels of **73**, mice (5 per group) received 10 mg/kg of i.p. injection in a volume of 500 μl of 2% DMSO in saline. After selected time points, mice were sacrificed (CO_2_ atmosphere) and blood (approximately 0.7–0.9 mL) was collected by intracardiac puncture using citrate as an anticoagulant. Then, plasma was obtained by centrifugation at 800*g* at 4 °C for 10 min and immediately frozen at −80 °C. For the extraction of **73** and the internal standard (IS) tolbutamide from mouse plasma, the method of protein precipitation was employed. The frozen mouse plasma samples were thawed at room temperature. Ten microliters of IS solution (2 μg mL^–1^) were added at 100 μL of plasma and vortexed for 30 s. Then, 890 μL of ice-cold methanol was added to all tubes and the extraction was performed by vortex mixing for 5 min, followed by centrifugation for 10 min at 14,600 rpm at 25 °C. The supernatants were collected, filtered by 0.45 μM of RC-membranes, and then injected in UHPLC-MS/MS. Stock solutions of **73** and tolbutamide were prepared in DMSO (1 mg mL^–1^). The standards of **73** were prepared by dilution of the stock solution with methanol to obtain working stock solutions in plasma of the following concentrations: 0.25, 2.5, 5, 10, 25, and 50 ng mL^–1^ for calibration curve. Calibration standards were prepared by spiking 100 μL of mouse plasma with suitable working solutions of **73** and IS to obtain a final concentration of 20 ng mL^–1^ of the IS in each sample. The analyses were performed using a Shimadzu Nexera UHPLC (Kyoto, Japan) consisting of two LC-30AD pumps, an SIL-30AC autosampler, a CTO-20AC column oven, and a CBM-20A controller. The samples were separated on a reversed-phase analytical column (Luna Omega Polar C18, 1.6 μm × 100 mm × 2.1 mm, Phenomenex, Bologna, Italy). The oven temperature was set at 40 °C. The mobile phases consisted of H_2_O (component A) and ACN (component B), both acidified with 0.1% v/v HCOOH, delivered at a constant flow rate of 0.3 mL min^–1^. Analysis was performed in gradient elution as follows: 0.0–7.0 min, 30–95% B; 7.0–8.0 min, isocratic to 95% B; in 2 min returning to 30% B and remaining unchanged at the end of the run. The total run time was 14 min.

The chromatographic system was coupled online to a triple quadrupole LC-MS-8050 (Shimadzu) equipped with an electrospray ionization (ESI) source operating in positive mode. Quantification was performed in MRM (multiple reaction monitoring) mode. The transitions were **m*/*z** 383 > 133.1 (quantifier ion) and 383 > 278.2 (qualifier ion) for compound **73** and **m*/*z** 271 > 74 (quantifier ion) and 271 > 155 (qualifier ion) for IS, and a dwell time of 100 ms. Interface temperature, DL temperature, and heat block temperature were set to 250, 250, and 300 °C, respectively. Nebulizing gas, heating gas, and drying gas flows were set to 3, 10, and 10 L min^–1^, respectively. All of the data was collected in the centroid mode and acquired and processed using Lab Solution workstation software. The drug half-life was calculated using GraphPad Prism 9.1.0 (GraphPad Software, La Jolla, CA).

### Zymosan-Induced Peritonitis

CD-1 mice were pretreated i.p. with **73** (10 mg/kg), zileuton (10 mg/kg), AUDA (10 mg/kg), or vehicle (0.5 mL, DMSO 2% in saline) 30 min before zymosan (2 mg/mL in saline, i.p., 0.5 mL, Sigma-Aldrich). Mice were sacrificed by inhalation of CO_2_ after 30 min or 4 h to analyze peritoneal LTC_4_ (30 min), LTB_4_, cell infiltration, PGE_2_, nitrite/nitrate (NOx), and TNF-α peritoneal exudate levels. Peritoneal exudates were collected and centrifugated; cells were counted in exudates after trypan blue staining. Levels of LTB_4_, LTC_4_, PGE_2_ (Cayman Chemical, BertinPharma, Montigny Le Bretonneux, France), and TNF-α (R&D Systems, Aurogene, Rome, Italy) were quantified in the exudate by ELISA according to the manufacturer’s instructions. Measurements of nitrite and nitrate (NOx) were based on the reduction of nitrate to nitrite by cadmium^68^ and subsequent determination of nitrite by Griess reaction. The reduction of nitrate to nitrite was performed in a microplate: 40 μL of STB (75% of 0.49 M NH_4_Cl and 25% of 0.06 M Na_2_B_4_O_7_) and 115 μL of nitrate standard curves or samples were pipetted in each well. Cadmium granules (2–2.5 g) were rinsed three times with deionized distilled water, and then they were added to samples. The microplate was then shaken automatically for 90 min. Subsequently, 155 μL of the mixture from each well was centrifugated; then, 100 μL of supernatants were transferred into another microplate. 100 μL of Griess reagent (0.1% naphthylethylenediamide dihydrochloride in H_2_O and 1% sulfanilamide in 5% concentrated H_2_PO_4_; vol. 1:1; Sigma-Aldrich, Milan, Italy) were added, and absorbance was measured within 10 min in a spectrophotometer at a wavelength of 540 nm.

### Quantitation of Eicosanoids in Peritoneal Exudate Fluid

The analyses were performed using a Shimadzu Nexera UHPLC (Kyoto, Japan) coupled online to a triple quadrupole LC-MS-8050 (Shimadzu) equipped with an electrospray ionization (ESI) source operating in negative mode. The samples were separated on a reversed-phase analytical column (Acquity UPLC BEH C18 column, 130 Å, 1.7 μm × 100 mm × 2.1 mm, Waters, Milford, MA). The oven temperature was set at 45 °C. The mobile phases consisted of ACN:H_2_O 60:40 acidified with 0.02% v/v CH_3_COOH (component A) and ACN: IPA 50:50 (component B), delivered at a constant flow rate of 0.3 mL min^–1^. Analyses were performed in gradient elution as follows: 0.0–6.0 min, 1–55% B; 6.00–6.50 min, 55–95% B; 6.50–7.50 min isocratic to 95% B; in 3.5 min returning to 1% B and remained unchanged at the end of the run. The total run time was 11.5 min.

Eicosanoid quantification was carried out in multiple reaction monitoring (MRM) mode monitoring transition from deprotonated precursor to product. For optimization of the mass spectrometer standards, 5,6-EET, 8,9-EET, 11,12-EET, 14,15-EET, 5,6-DHET, 8,9-DHET, 11,12-DHET, 14,15-DHET, 11,12-DHET d_11_, and 14,15-EET d_11_ at a concentration of 500 ng mL^–1^ in EtOH were individually introduced into the mass spectrometer with flow injection mode. As the *m*/*z* 319 and *m*/*z* 337 precursor ions were obtained for all EETs and DHETs, respectively, the most specific ion product transition was used for quantification, as reported in Table S4. The dwell time was set to 100 ms for all of the monitored transitions. Interface temperature, DL temperature, and heat block temperature were set to 300, 250, and 350 °C, respectively. Nebulizing gas, heating gas, and drying gas flows were set to 3, 10, and 10 L min^–1^, respectively. All of the data was collected in the centroid mode and acquired and processed using Lab Solution workstation software. For the preparation of calibration curves, stock solutions (100 μg mL^–1^) of 5,6-EET, 8,9-EET, 11,12-EET, 14,15-EET, 5,6-DHET, 8,9-DHET, 11,12-DHET, and 14,15-DHET were diluted with ethanol. Working standard solutions for all eicosanoids were prepared by the serial dilution of stock solutions to obtain the necessary concentrations (2.5–200 ng mL^–1^). A solution containing internal deuterated eicosanoid standards (ISs: 11,12-DHET-*d*_11_ and 14,15-EET-*d*_11_) was prepared at 100 ng mL^–1^ in ethanol. Quantitation of eicosanoids in peritoneal exudate fluid (Tables S5 and S6) was performed using linear regression of the response ratios (peak area analyte/peak area internal standard) obtained from the calibration curve to calculate the corresponding eicosanoid amount. The extraction of eicosanoids from peritoneal exudates was performed, as previously reported.^69^ Briefly, 500 μL of peritoneal exudates were diluted to 1 mL with phosphate salt buffer and spiked with ISs. The eicosanoids were extracted using Strata-X reversed-phase SPE columns (Phenomenex, Bologna, Italy). After loading the sample, the columns were washed with 10% MeOH, and the eicosanoids were then eluted with MeOH. Prior to the LC-MS/MS analysis, the eluent was dried under vacuum using a SpeedVac (Savant, Thermo Scientific, Milan, Italy) and dissolved in 50 μL of UHPLC solvent A.

### Experimental Model of Murine Asthma

BALB/c mice were treated with 0.4 mL s.c. of a suspension containing 100 μg of ovalbumin (OVA) absorbed to 3.3 mg of aluminum hydroxide gel on days 0 and 7.^70,71^ Compound **73** (10 mg/kg), zileuton (35 mg/kg), or vehicle (dimethyl sulfoxide 4%, 0.5 mL) was administered i.p. 30 min (Figure 5A) before each OVA administration. Animals were sacrificed on day 21 by an overdose of enflurane, and lungs, bronchi, and plasma were collected. In particular, blood was collected by intracardiac puncture using citrate as an anticoagulant. Then, plasma was obtained by centrifugation at 800*g* at 4 °C for 10 min and immediately frozen at −80 °C.^72^ Total IgE levels were measured by ELISA kit (BD Biosciences, Pharmingen, San Jose, CA). Bronchi were cut in rings of 1–2 mm in length, placed in organ baths, and fixed to an isometric force transducer 7006 connected to a Powerlab 800 (AD Instruments, Ugo Basile, Comerio, Italy). After stretching the rings to a resting tension of 0.5 g and equilibration for at least 30 min, the rings were challenged with carbachol (1 μM) until a reproducible response was observed. To assess bronchial reactivity, the cumulative response to carbachol (0.001 to 3.16 μM) and salbutamol (0.01–30 μM) was measured.^50^ Results were expressed as dyne per mg tissue.

The right lung lobes harvested from mice were rapidly fixed in 4% formalin. The tissues were embedded in paraffin, and cryosections of 7 μm were cut. The slices were processed to remove the paraffin, and following rehydration, hematoxylin and eosin (H&E) staining was performed. Sections were analyzed using a Leica Microsystem with a scale bar of 50 μm (H&E). Pulmonary cell infiltration and epithelial thickness were evaluated using ImageJ Fiji software.^50^

Left lungs were isolated and homogenized in PBS (Sigma-Aldrich, Milan, Italy). The homogenate was centrifuged (4 °C, 6000*g*, 10 min).^50^ The levels of LTC_4_ (Cayman Chemical, BertinPharma, Montigny Le Bretonneux, France), IL-13, and IL-4 (Invitrogen, Vienna, Austria) were measured by ELISA according to the manufacturer’s instructions. The levels of LTC_4_, IL-13, and IL-4 were expressed as pg/mL.

### Statistical Analysis

The results are expressed as the mean ± SEM of *n* observations, where *n* represents the number of animals or number of experiments performed on different days. Statistical evaluation was performed by one-way or two-way ANOVA using GraphPad InStat (Graphpad Software Inc., San Diego, CA) followed by a Bonferroni post hoc test for multiple comparisons, respectively. Post hoc tests were run only when *F* achieved *P* < 0.05, and there was no significant variance in the homogeneity. A *P* value <0.05 was used to define statistically significant differences between mean values.

## Supporting Information Available

IFD score values calculated by induced fit docking of Schrödinger suite (Tables S1 and S2); NMR spectra and HPLC traces of final compounds (Figures S1–S75); effect of **73** on COX-1 and COX-2 in intact cells (Figure S76); compound **73** concentration in mouse plasma after intraperitoneal administration (Table S3); optimal LC-MS/MS parameters for eicosanoids quantification (Table S4); typical MRM chromatograms of eicosanoid standards and their deuterated standards (Figure S77); method validation parameters for the quantitation of dihydroxyeicosatrienoic acids (Table S5); and method validation parameters for the quantitation of epoxyeicosatrienoic acids (Table S6) (PDF)
Molecular formula strings (CSV)

## Abbreviations Used

AA: arachidonic acid
AUDA: 12-(3-adamantan-1-yl-ureido)-dodecanoic acid
BOC: *tert*-butyloxycarbonyl
CBZ: benzyloxycarbonyl
COX: cyclooxygenase
CYP450: cytochrome P450 monooxygenases
DCM: dichloromethane
DMF: dimethylformamide
DiHETrEs: dihydroxyeicosatrienoic acids
EETs: epoxyeicosatrienoic acids
FLAP: 5-LOX activating protein
20-HETE: 20-hydroxyeicosatetraenoic acid
HOBt: hydroxybenzotriazole
HBTU: 2-(1*H*-benzotriazole-1-yl)-1,1,3,3-tetramethyluronium hexafluorophosphate
5-LOX: 5-lipoxygenase
LPS: lipopolysaccharides
LTB_4_: leukotriene B4
LTC_4_: leukotriene C4
LTs: leukotrienes
MOM: methoxymethyl
NSAIDs: nonsteroidal anti-inflammatory drugs
NOx: nitric oxides
OVA: ovalbumin
PGs: prostaglandins
PGE_2_: prostaglandin E2
PMNLs: polymorphonuclear leukocytes
sEH: epoxide hydrolase
TEA: trimethylamine
Th2: T-helper type 2 cytokines
TIS: triisopropylsilane
TNF-α: tumor necrosis factor-α
TFA: trifluoroacetic acid
THF: tetrahydrofuran
