# Supplementary material for: Discovery and Optimization of Indoline-Based Compounds as Dual 5-LOX/sEH Inhibitors: In Vitro and In Vivo Anti-Inflammatory Characterization
Source: J Med Chem. 2022 Nov 1;65(21):14456–80. doi: 10.1021/acs.jmedchem.2c00817 (PMC9661480; doi:10.1021/acs.jmedchem.2c00817)
Supplement: Supplementary file 1 — jm2c00817_si_001.pdf [file jm2c00817_si_001.pdf]

SUPPORTING INFORMATION

Discovery and optimization of indoline-based compounds as dual 5-LOX/sEH inhibitors: *in vitro* and *in vivo* anti-inflammatory characterization

Ida Cerqua,[¥,#] Simona Musella,[‡,#] Lukas Klaus Peltner,[¶,#] Danilo D'Avino,[¥] Veronica Di Sarno,[‡] Elisabetta Granato,[¥] Vincenzo Vestuto,[‡] Rita Di Matteo,[¥] Simona Pace,[¶] Tania Ciaglia,[‡] Rossella Bilancia, [¥,¶] Gerardina Smaldone,[‡] Francesca Di Matteo,[‡] Simone Di Micco, [₸] Giuseppe Bifulco,[‡] Giacomo Pepe,[‡] Manuela Giovanna Basilicata,[‡] Manuela Rodriquez,[‡] Isabel M. Gomez-Monterrey,[¥] Pietro Campiglia,[‡,₸] Carmine Ostacolo,[¥] Fiorentina Roviezzo,[¥] Oliver Werz,[¶] Antonietta Rossi,[¥,*] Alessia Bertamino.[‡,*]

[¥] Department of Pharmacy, University Federico II of Naples, Via D. Montesano 49, 80131, Naples, Italy.

[‡] Department of Pharmacy, University of Salerno, Via G. Paolo II 132, 84084, Fisciano, Salerno, Italy.

[¶] Department of Pharmaceutical/Medicinal Chemistry, Institute of Pharmacy, Friedrich-Schiller-University, Philosophenweg 14, D-07743 Jena, Germany.

[₸] European Biomedical Research Institute (EBRIS), Via S. De Renzi 50, 84125, Salerno, Italy.

Corresponding Authors: Antonietta Rossi (antrossi@unina.it) and Alessia Bertamino (abertamino@unisa.it)

Table of content:

1. **Supplementary table:**
   Table S1. IFD score values calculated by Induced-Fit Docking of Schrödinger suite **S2**
   Table S2. IFD score values calculated by Induced-Fit Docking of Schrödinger suite **S3-S8**
2. **NMR spectra and HPLC traces of synthesized compounds. S9-S83**
3. **Figure S76:** Effect of **73** on COX-1 and COX-2 in intact cells **S84**
4. **Table S3:** Compound **73** concentration in mouse plasma **S85**
5. **Table S4** and **Figure S77:** LC-MS/MS and HPLC traces for lipidomic analyses **S86**
6. **Tables S5** and **S6:** Method validation paramenters for lipidomic analyses **S87**

Table S1. IFD score values calculated by Induced-Fit Docking of Schrödinger suite. For compounds **4**, **5**, **8** and **10** both enantiomers were considered in calculations and their configuration is indicated in brackets.

compound	IFDscore(kcal/mol)
**4** (R)	-1427,277
**4** (S)	-1426,616
**5** (R)	-1426,781
**5** (S)	-1424,094
**8** (R)	NDa
**8** (S)	NDa
**10** (R)	NDa
**10** (S)	-1425,922
**13**	-1426,755
**17**	-1432,766
**18**	-1436,503
**43**	-1436,350
**56**	-1432,691

[a]not determined

Table S2. IFD score values calculated for the in-house library. IFD score values calculated by Induced-Fit Docking of Schrödinger suite. For compounds **betac7** and **betac8** both enantiomers were considered in calculations and their configuration is indicated in brackets.

	structure	compound	IFDscore(kcal/mol)
1	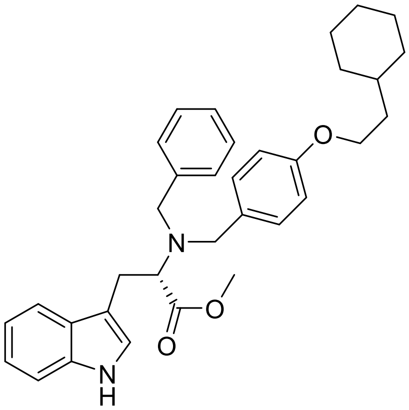	np132	-1433,585[a]
2	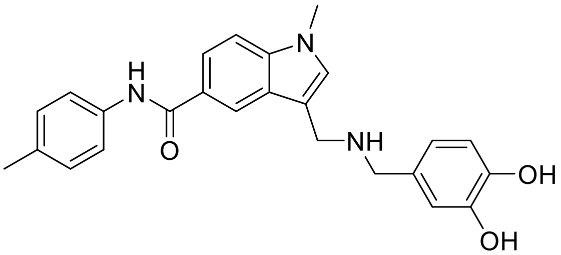	hs34	-1433,234[a]
3	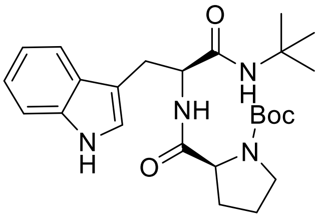	cov5	-1433,926[a]
4	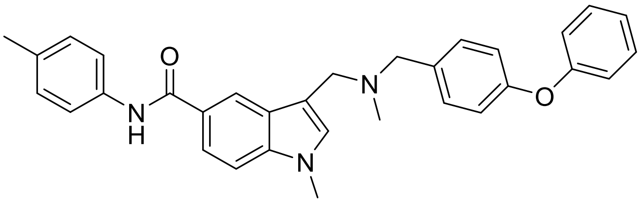	in17	-1432,795[a]
5	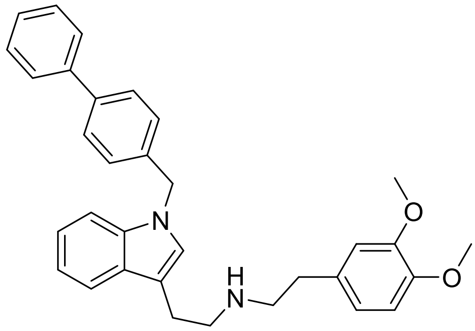	soylul4	-1430,605[a]

6	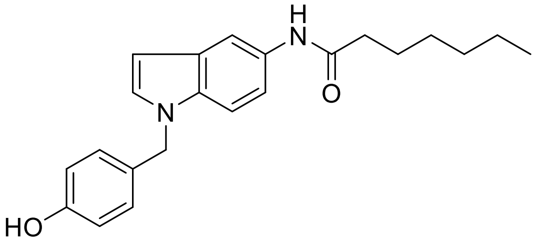	rt37	-1434,593[a]
7	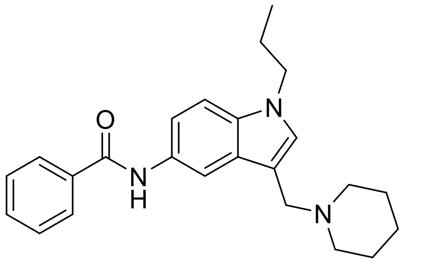	zos7	-1430,564[a]
8	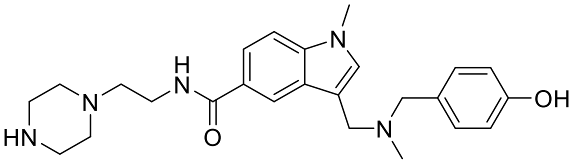	Haspin1	-1429,773[a]
9	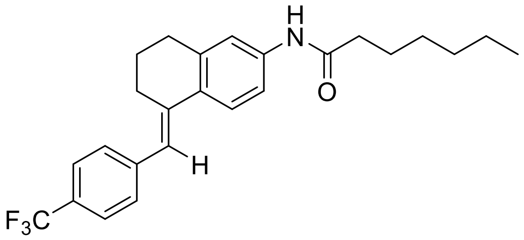	rt41	-1431,951[a]
10	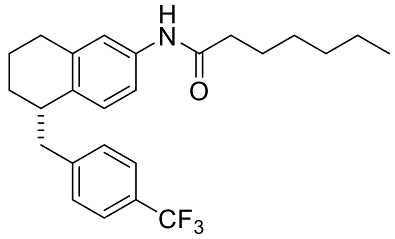	rt42	-1434,431[a]
11	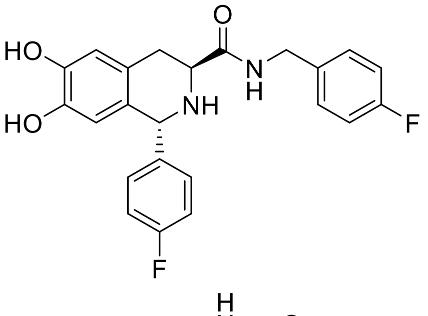	igm3_9	-1432,011[a]
12	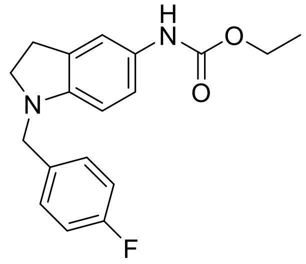	rt8	-1432,448[a]

13	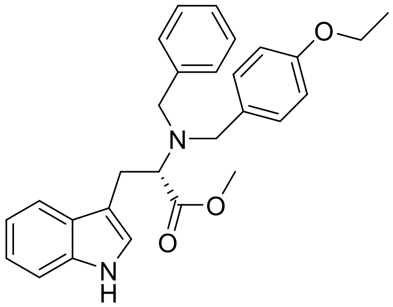	np131	-1431,91[a]
14	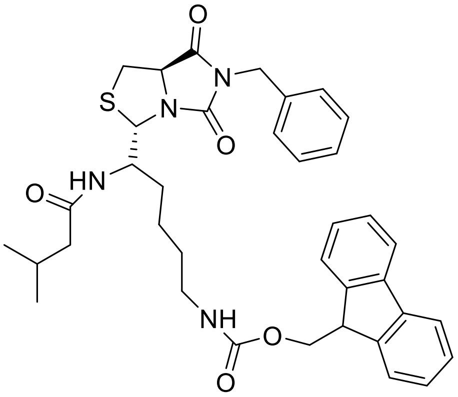	np79	-1432,227[a]
15	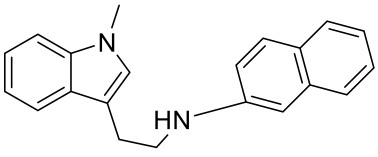	zos13	-1430,586[a]
16	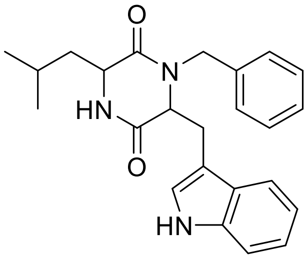	np154	-1429,45[a]
17	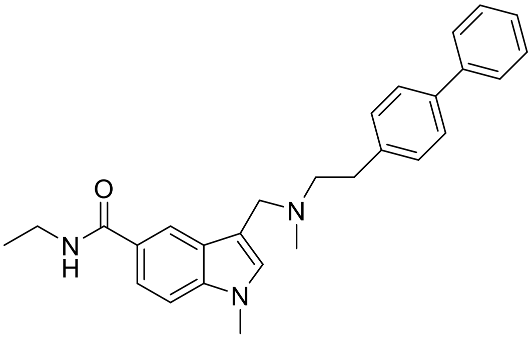	in16	-1429,985[a]
18	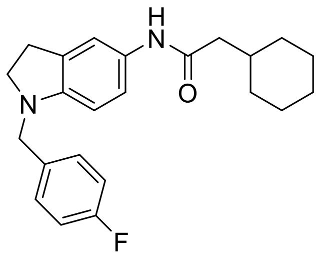	rt17	-1430,146[a]

19	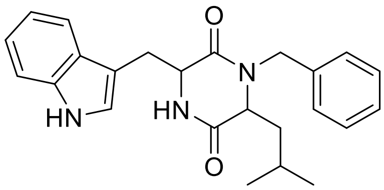	np153	-1431,336[a]
20	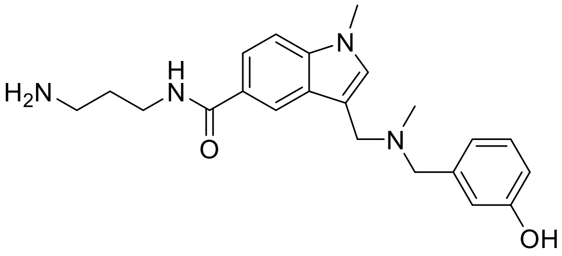	hs19	-1428,637[a]
21	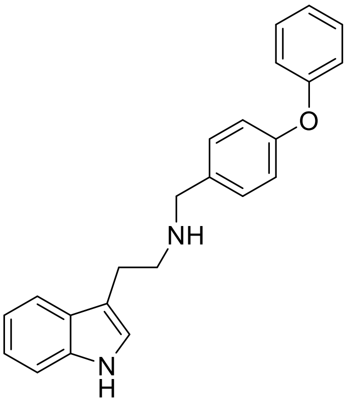	igm1_5	-1429,609[a]
22	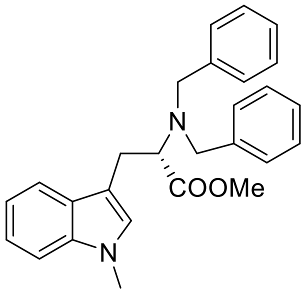	tr61	-1429,146[a]
23	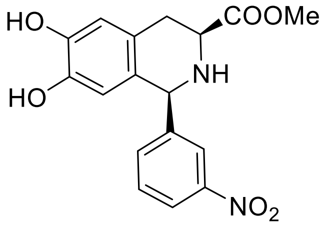	betac8 (S,S)	-1428,142[a]
24	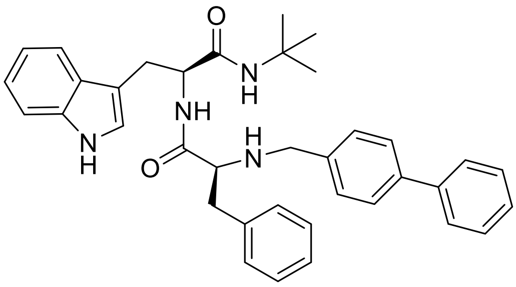	covid1	-1429,713[a]

25	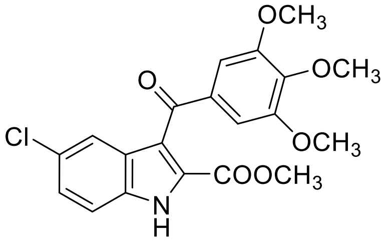	at14	-1428,463[a]
26	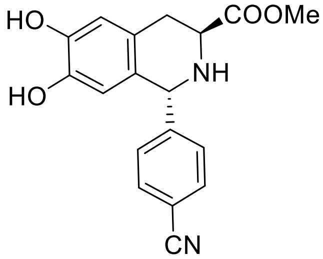	betac7 (S,R)	-1426,862[a]
27	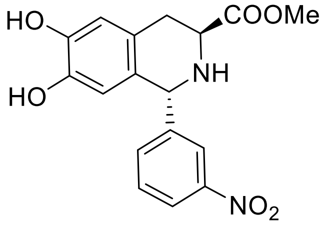	betac8 (S,R)	-1427,284[a]
28	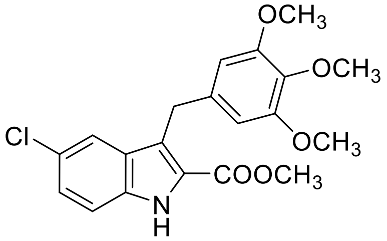	at15	-1425,559[a]
29	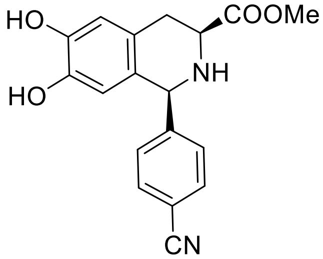	betac7 (S,S)	-1424,87[a]
30	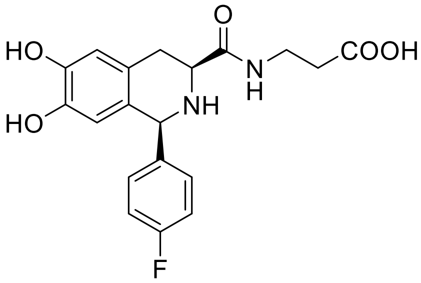	igm3_12	-1423,165[a]
31	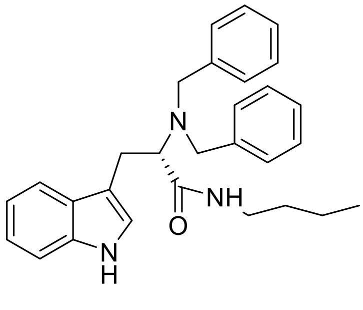	tr62	ND[b]

32	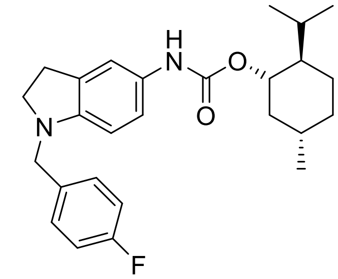	rt3	ND[b]
33	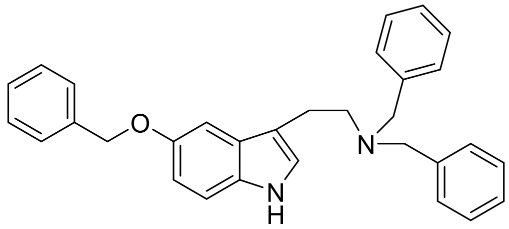	tr1	ND[b]
34	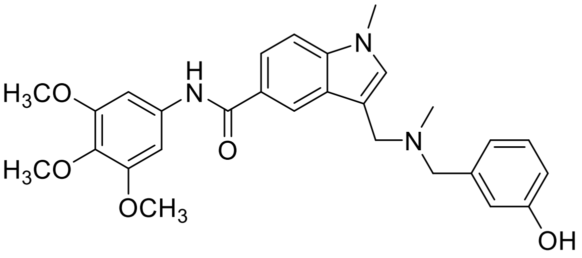	hs21	ND[b]
35	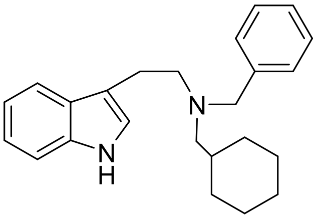	tr4	ND[b]
36	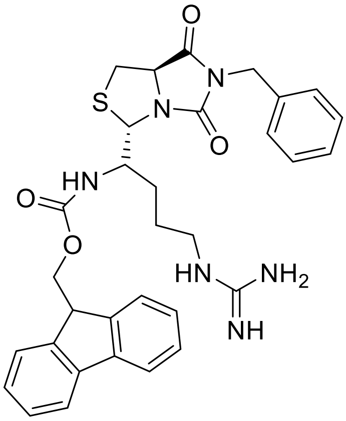	np78	ND[b]
37	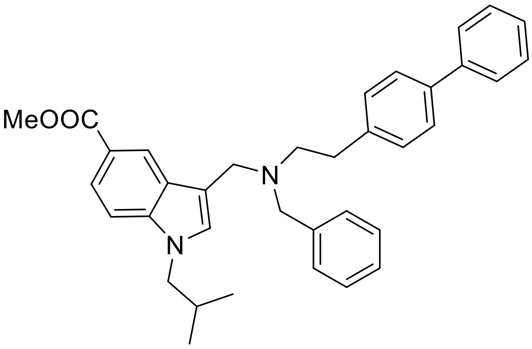	cov2	ND[b]

[a]distorted conformation

[b]not determined

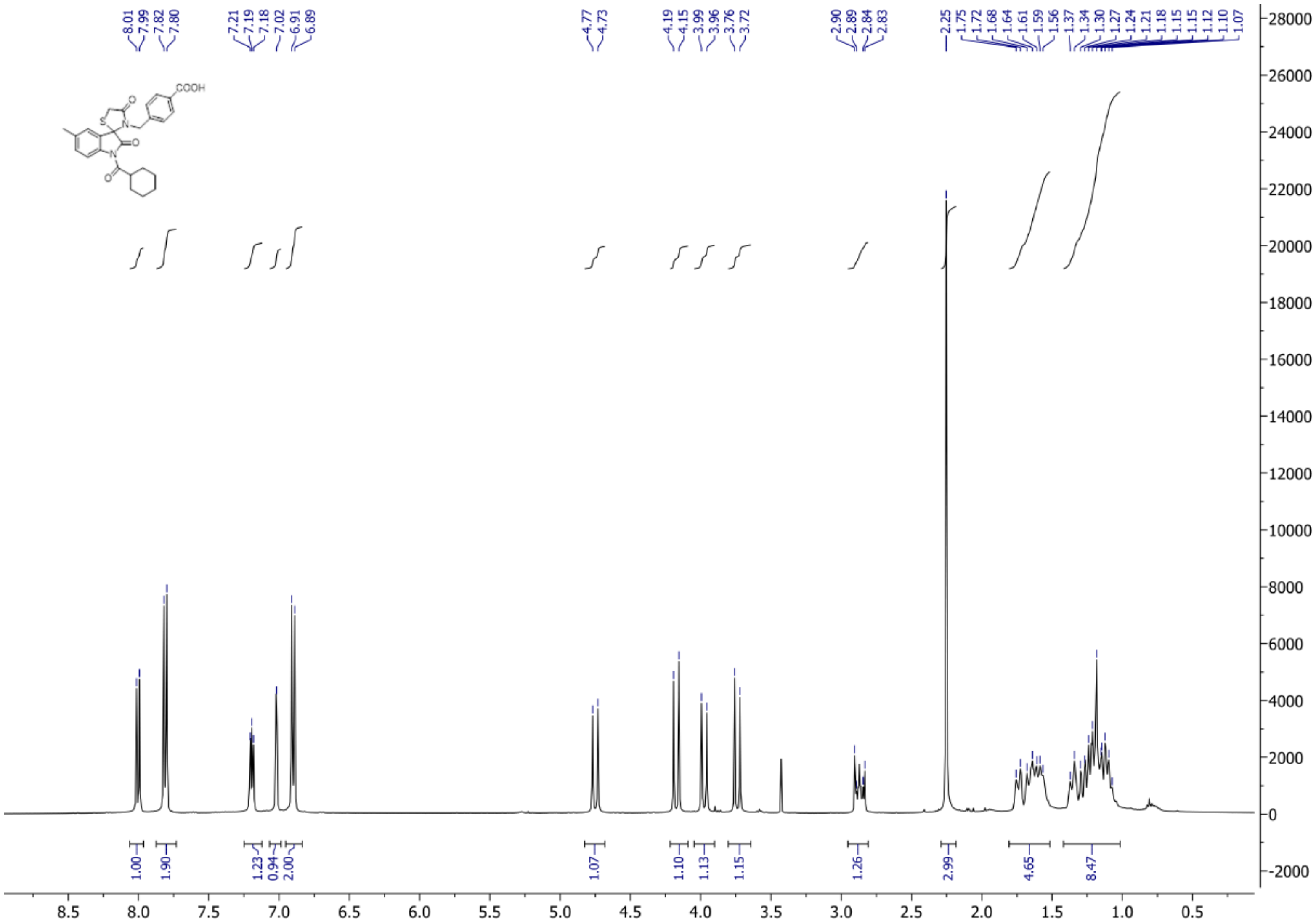

Figure S1: 1H NMR spectra of compound **4**

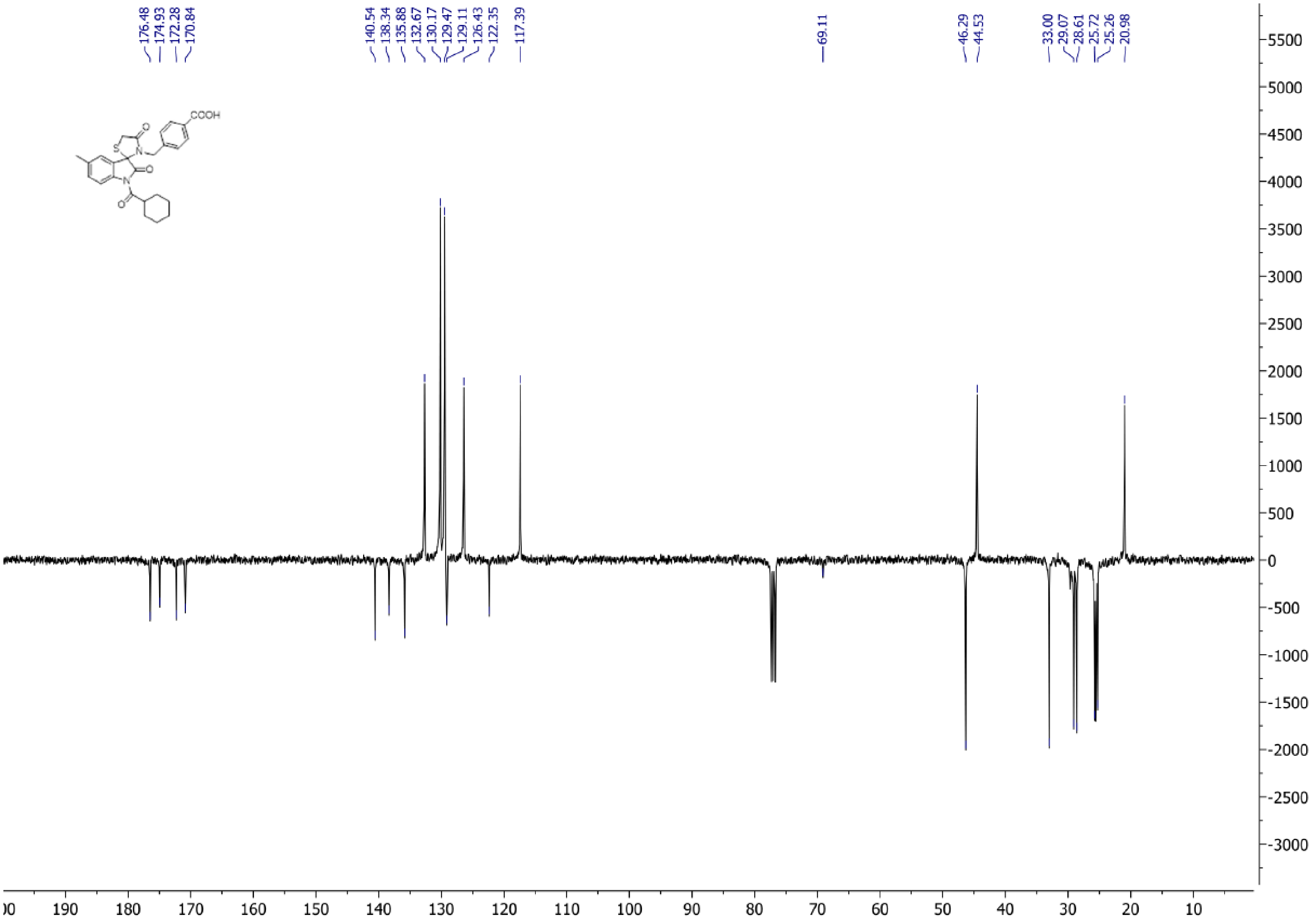

Figure S2: DEPT spectra of compound **4**

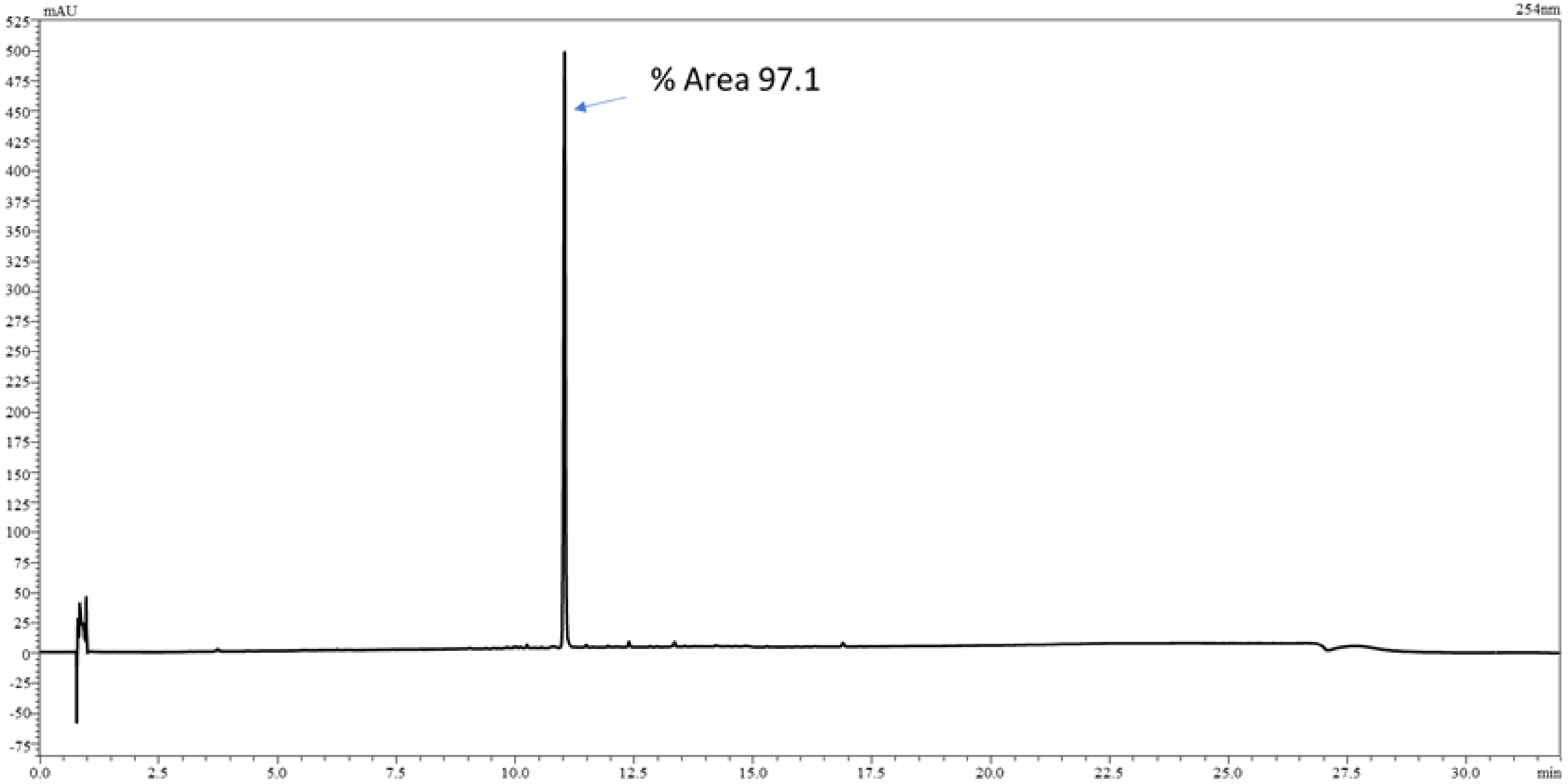

Figure S3: HPLC trace of compound **4**

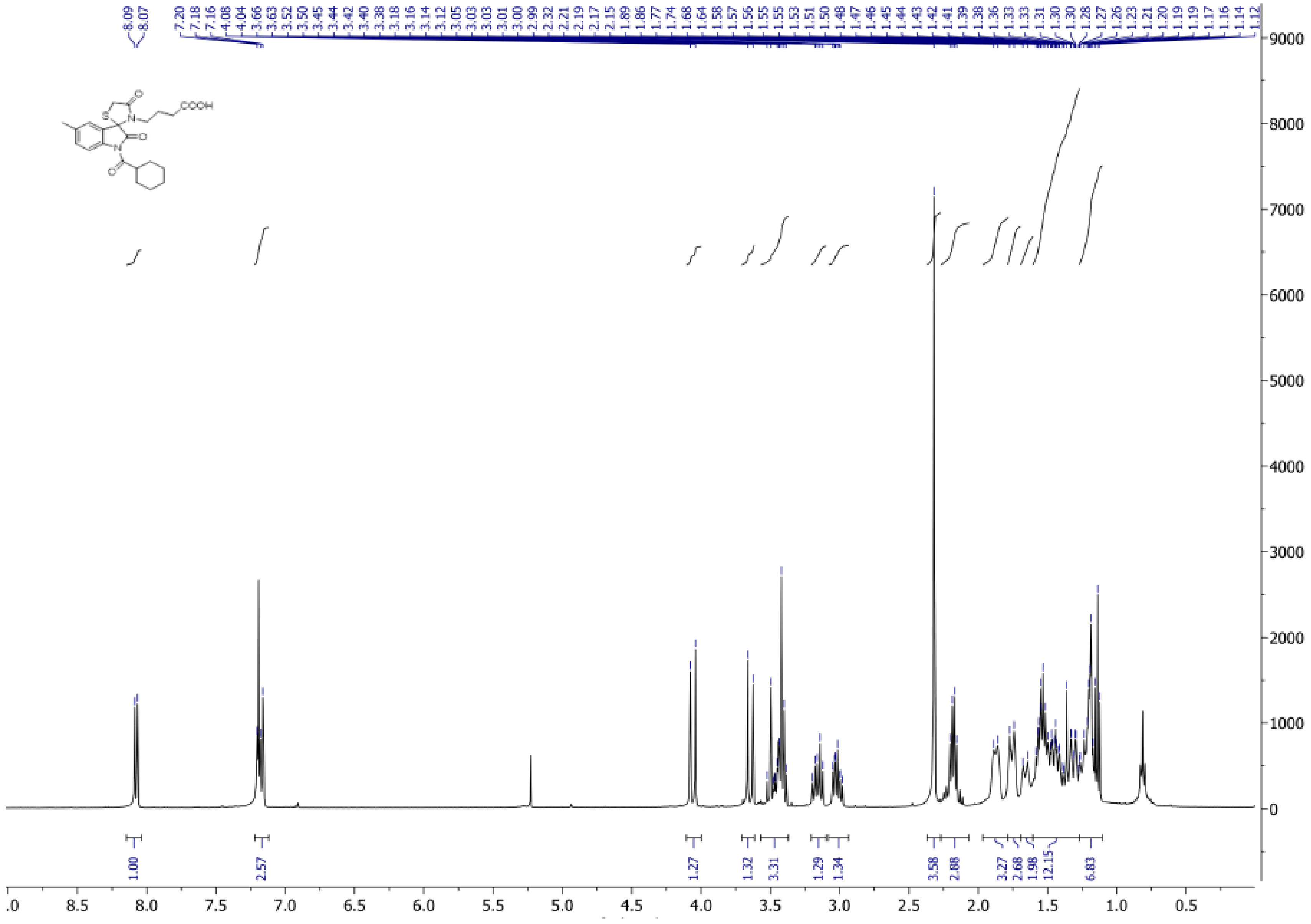

Figure S4: 1H NMR spectra of compound **5**

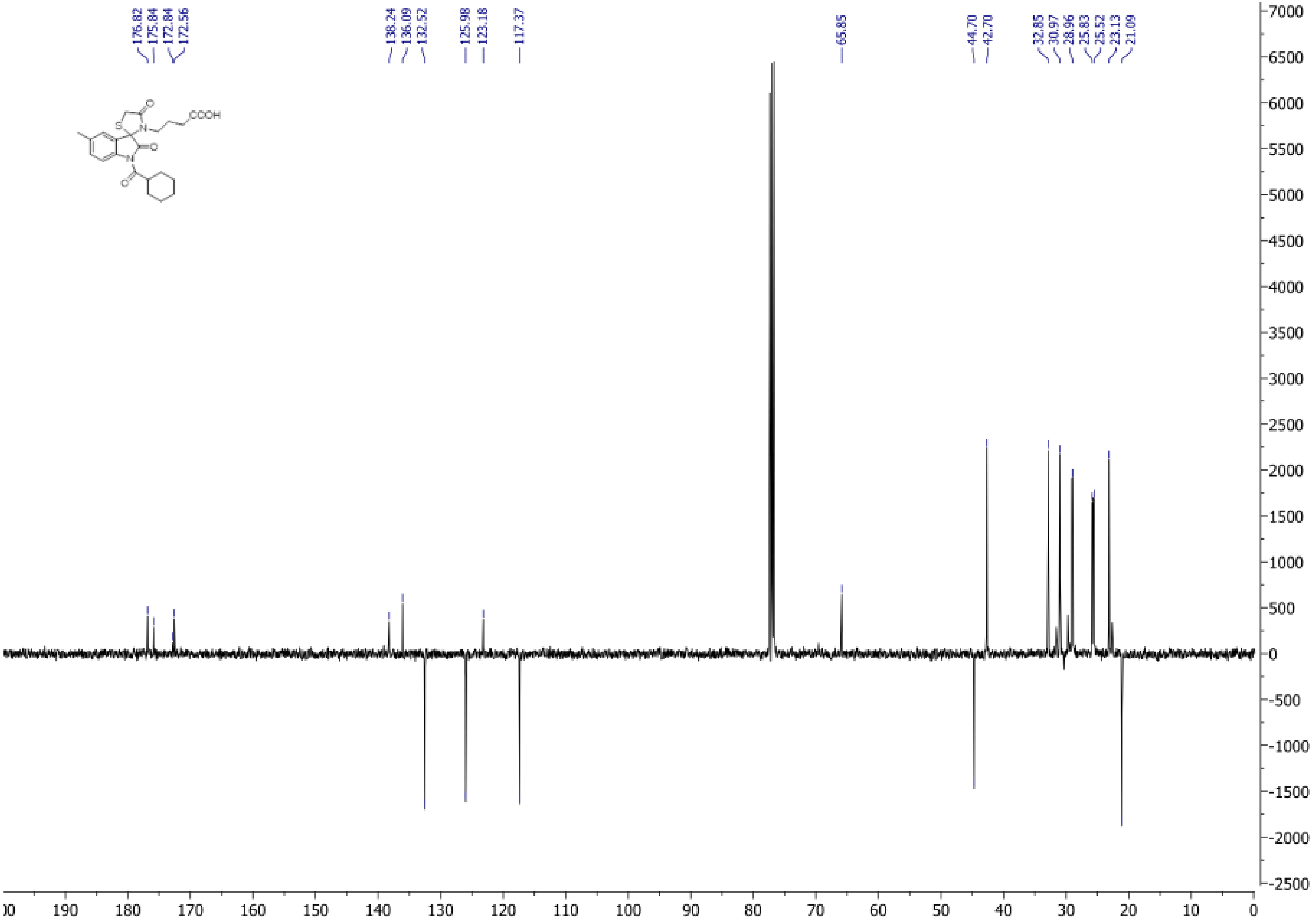

Figure S5: DEPT spectra of compound **5**

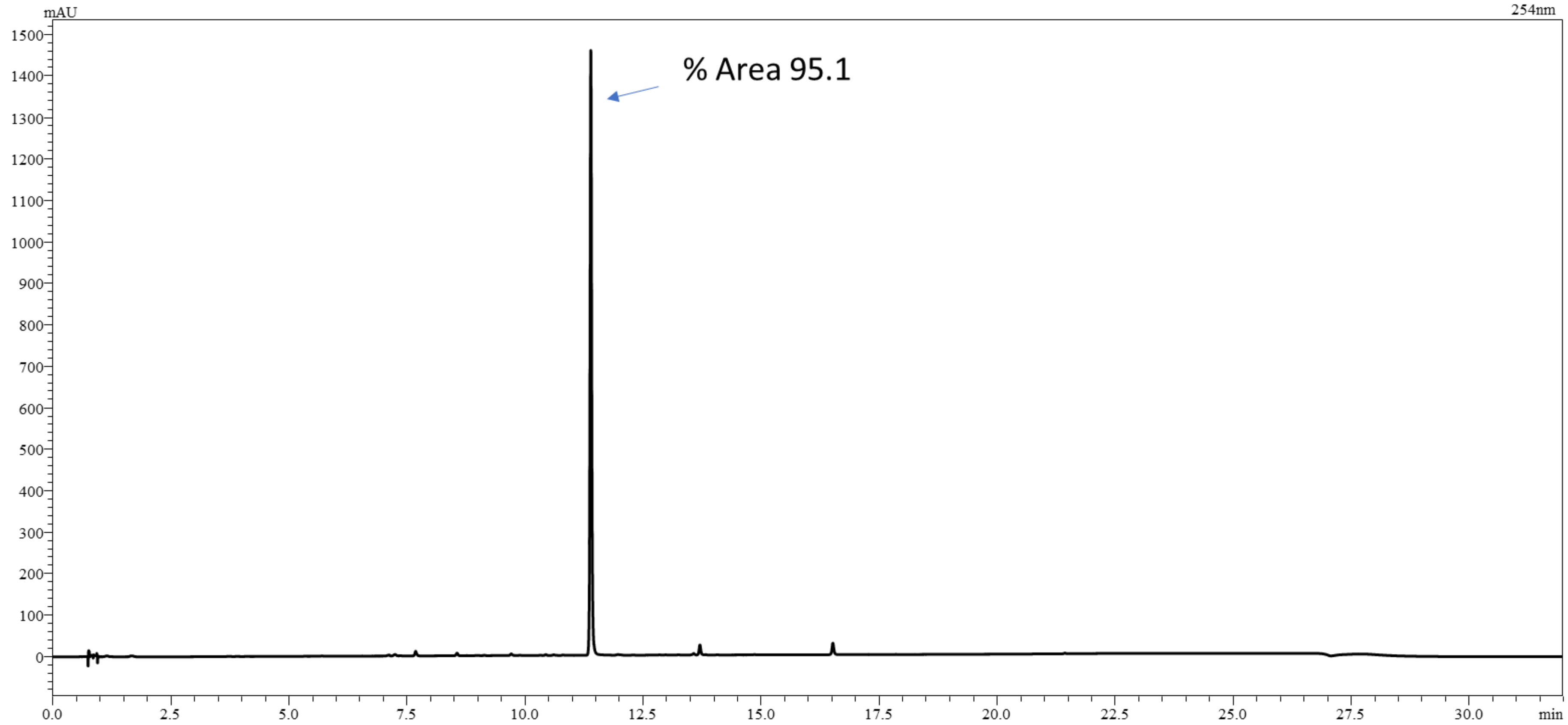

Figure S6: HPLC trace of compound **5**

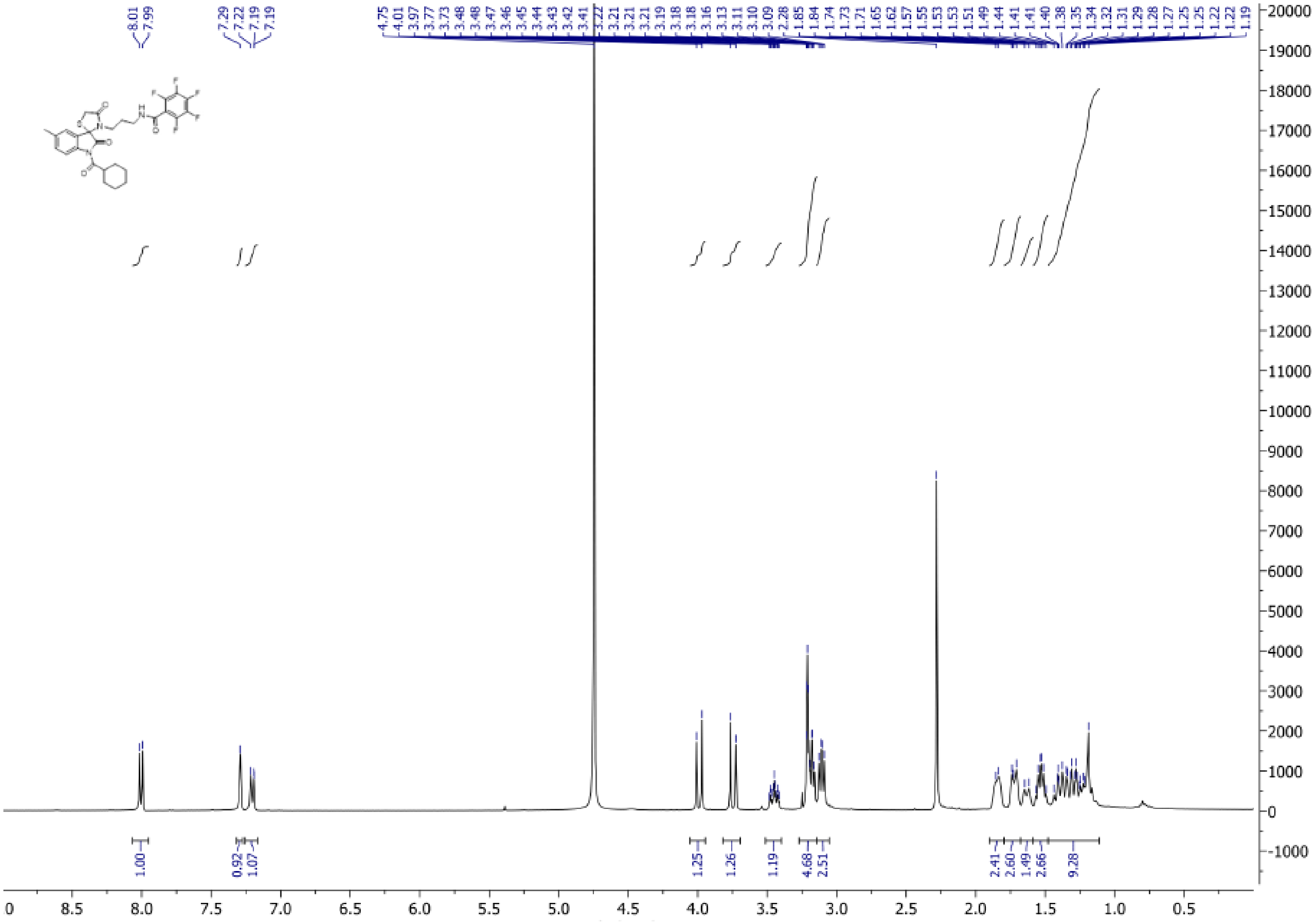

Figure S7: 1H NMR spectra of compound **8**

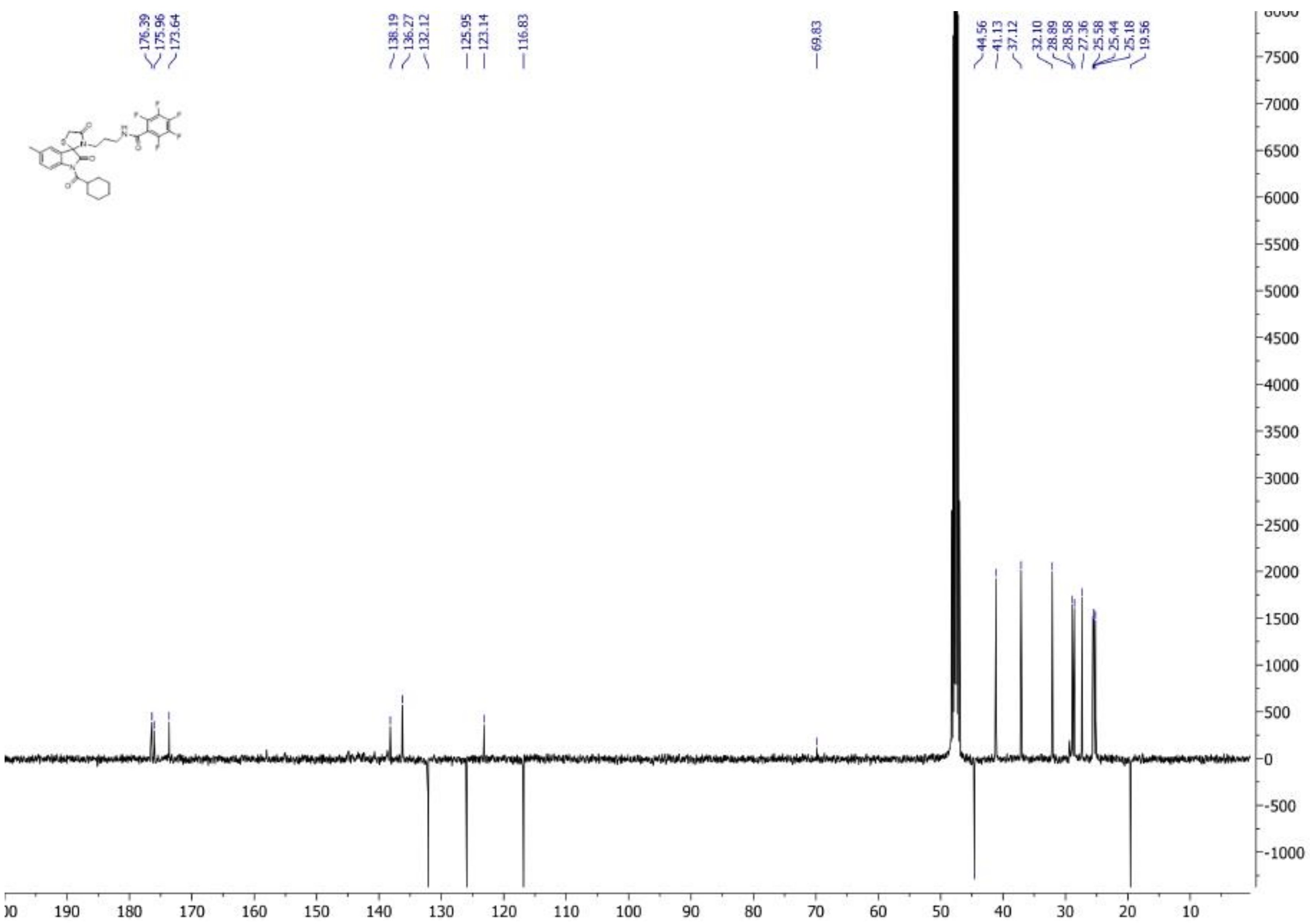

Figure S8: DEPT spectra of compound **8**

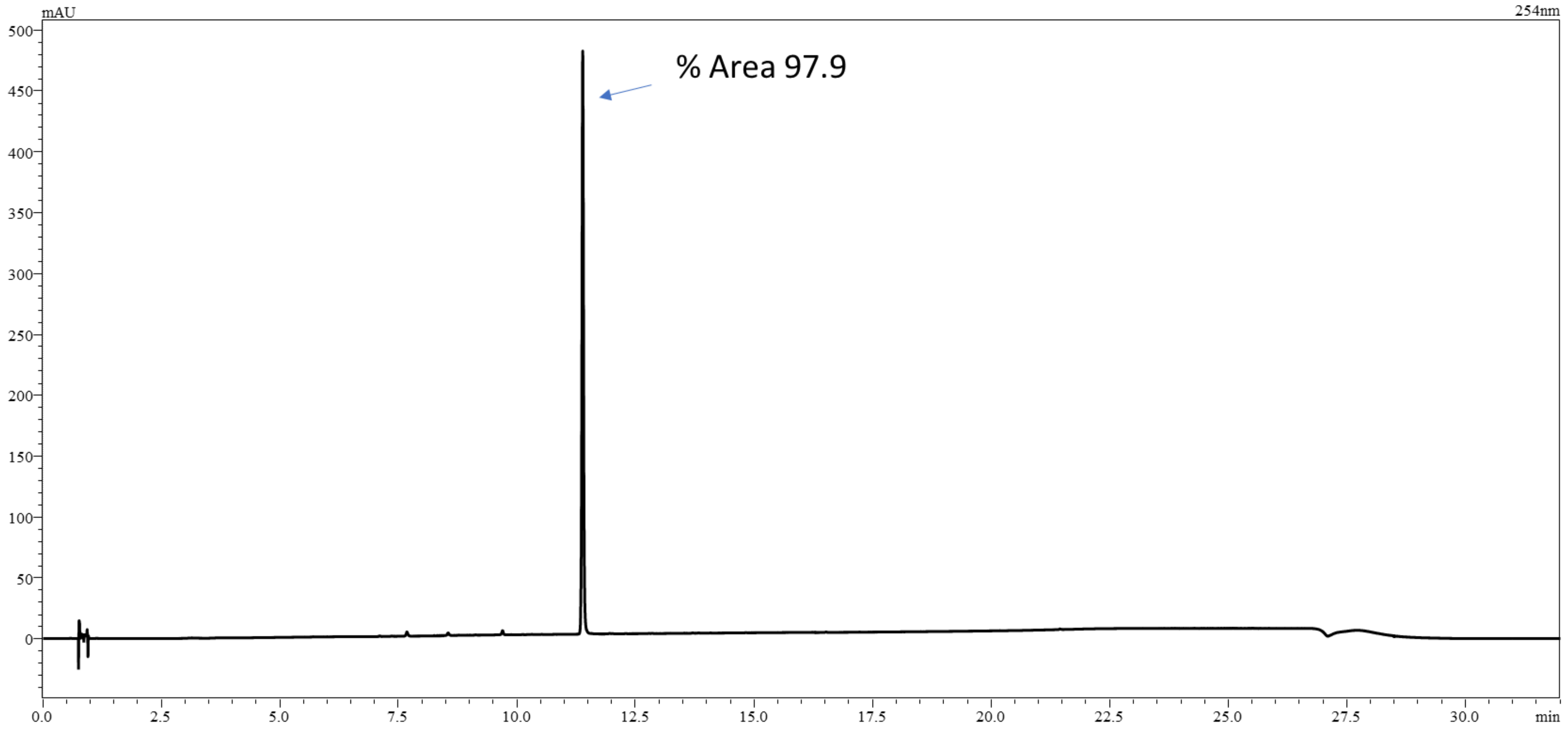

Figure S9: HPLC trace of compound **8**

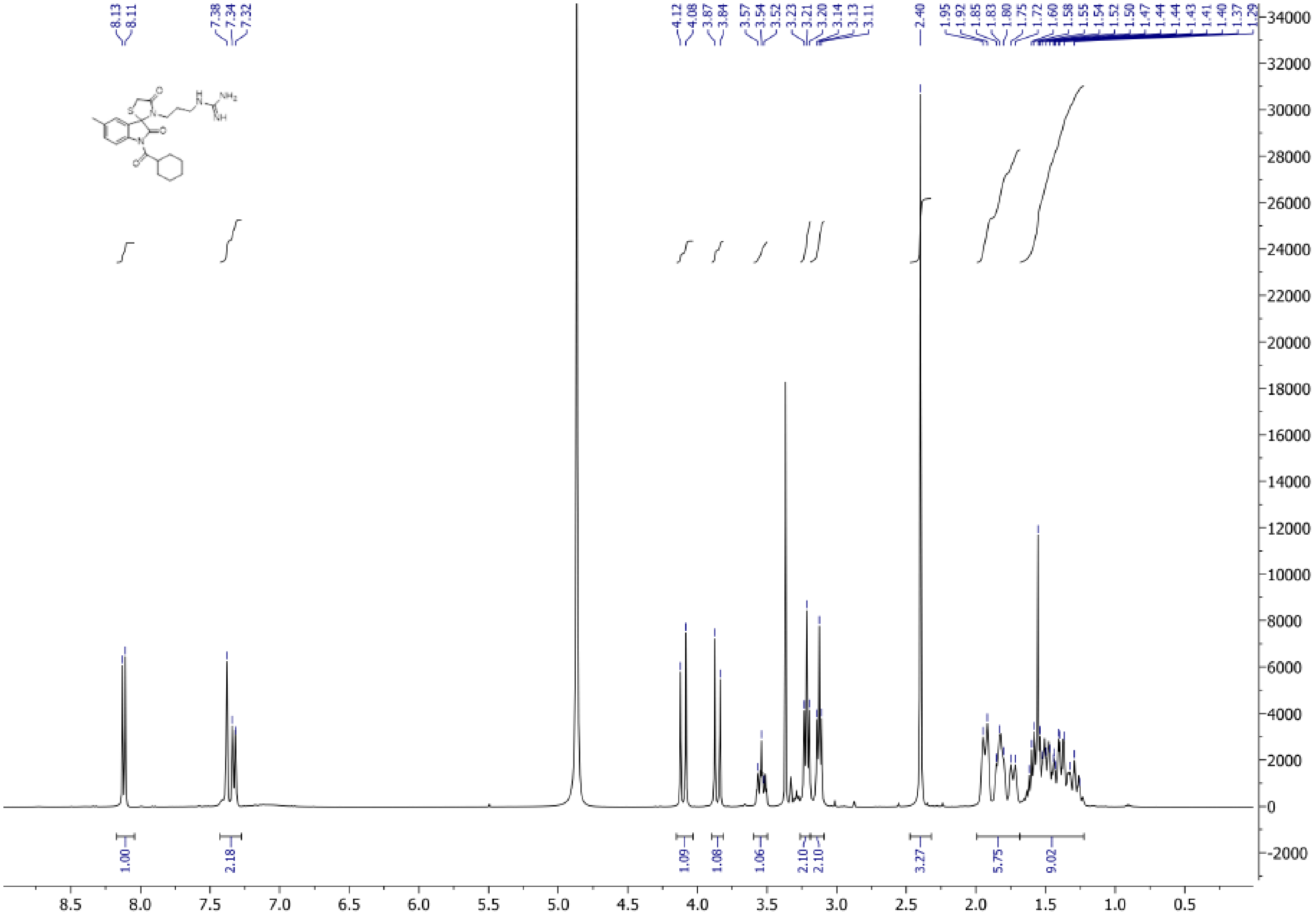

Figure S10: 1H NMR spectra of compound **10**

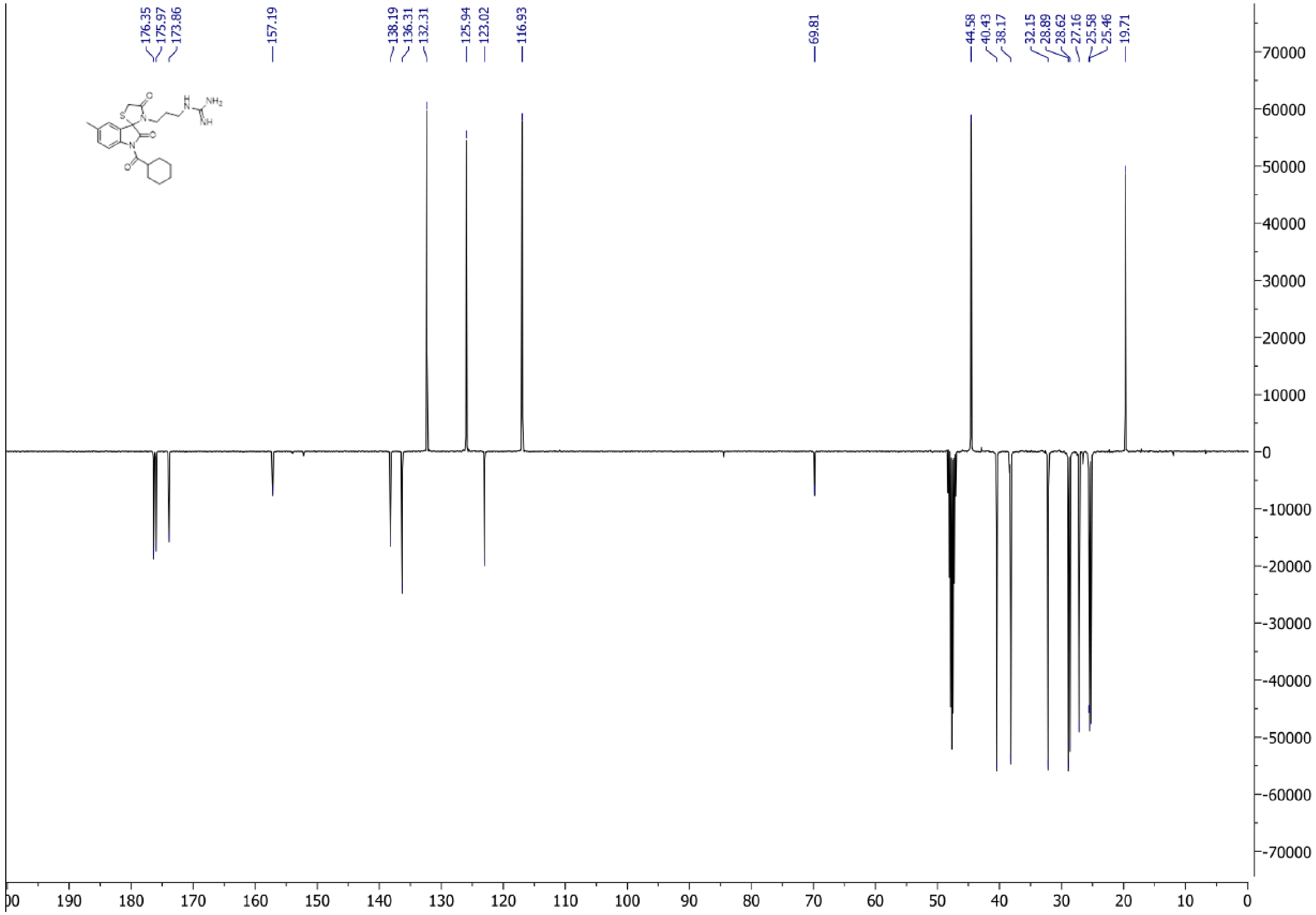

Figure S11: DEPT spectra of compound **10**

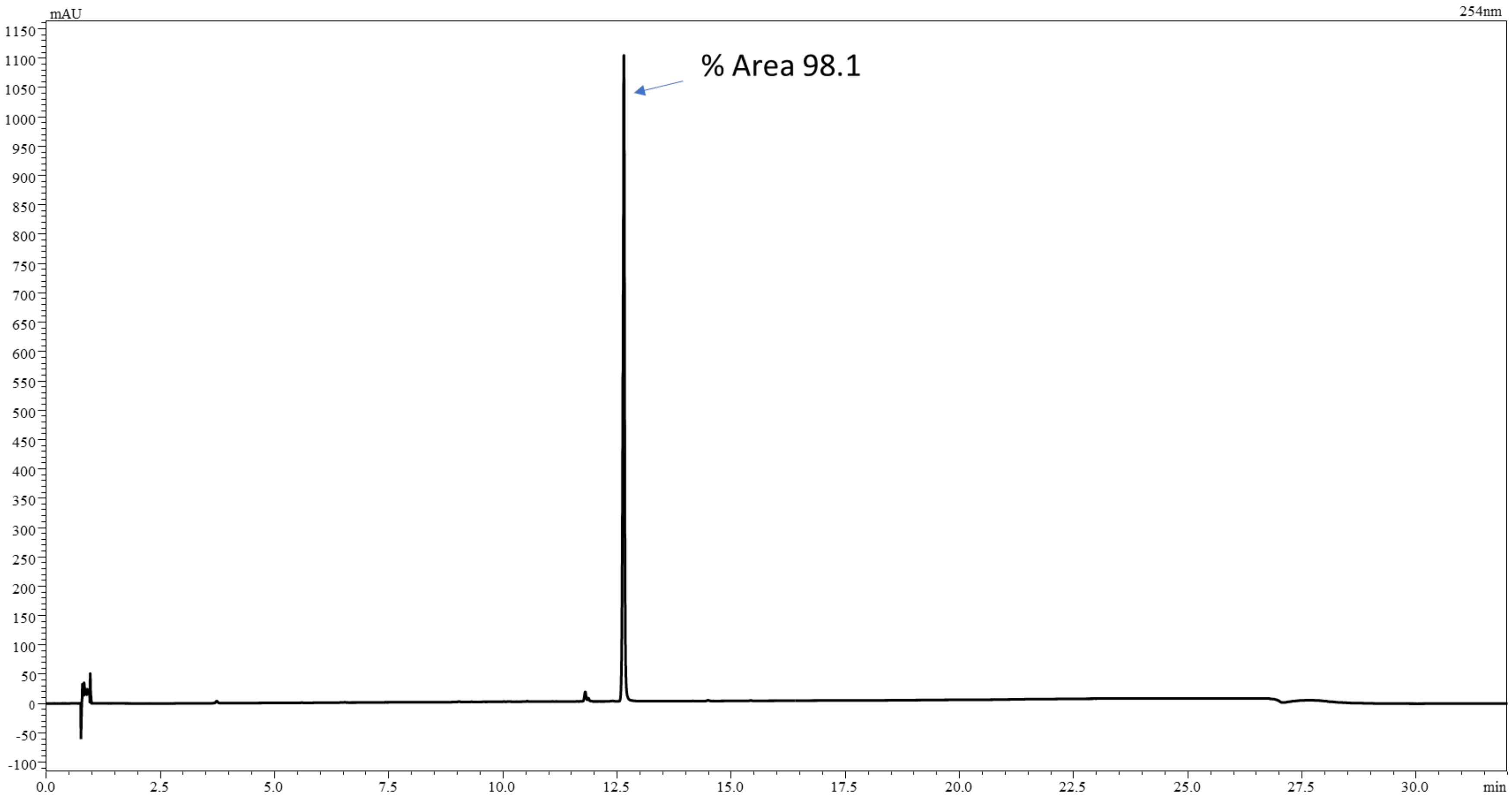

Figure S12: HPLC trace of compound **10**

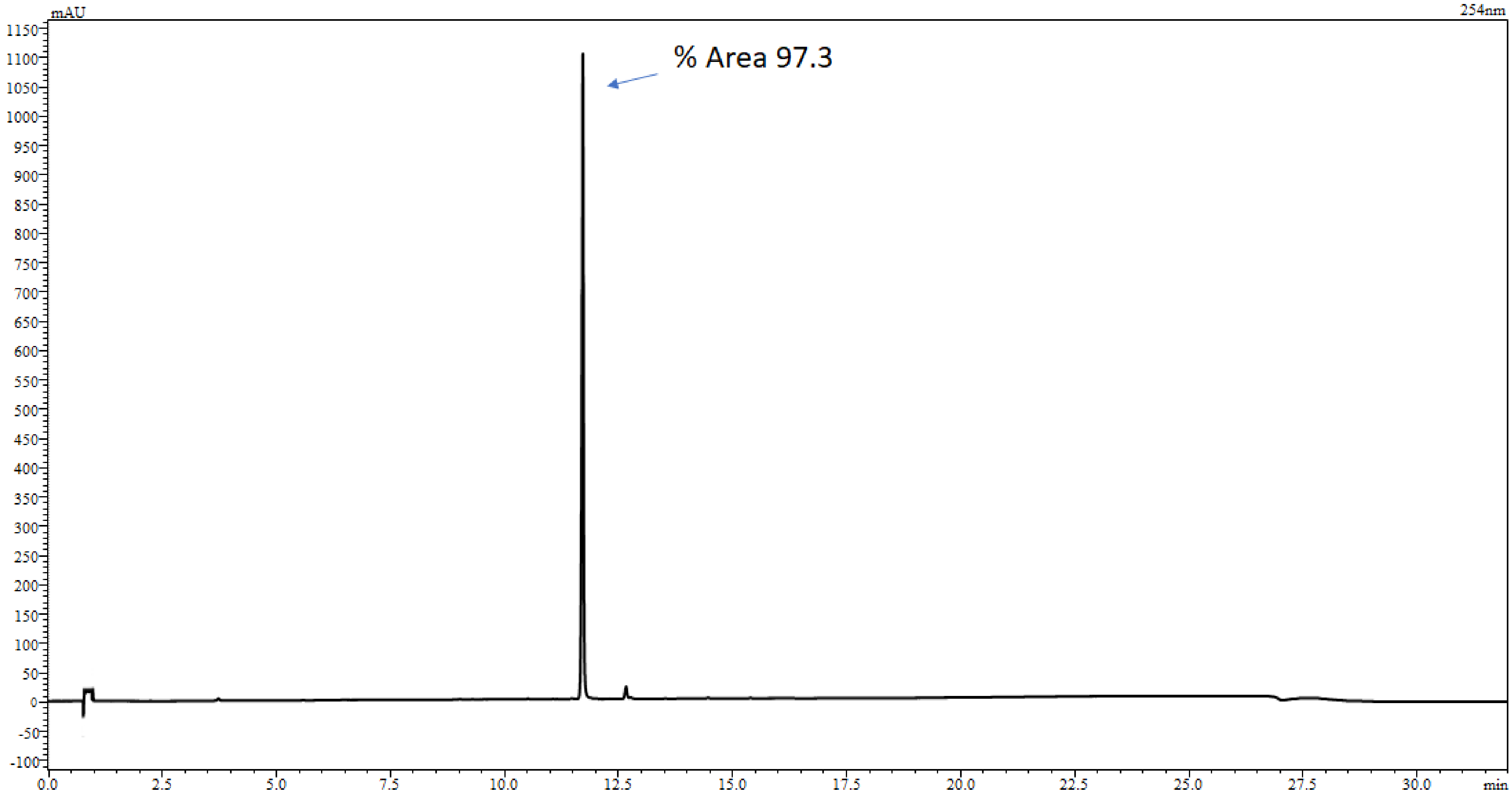

Figure S13: HPLC trace of compound **13**

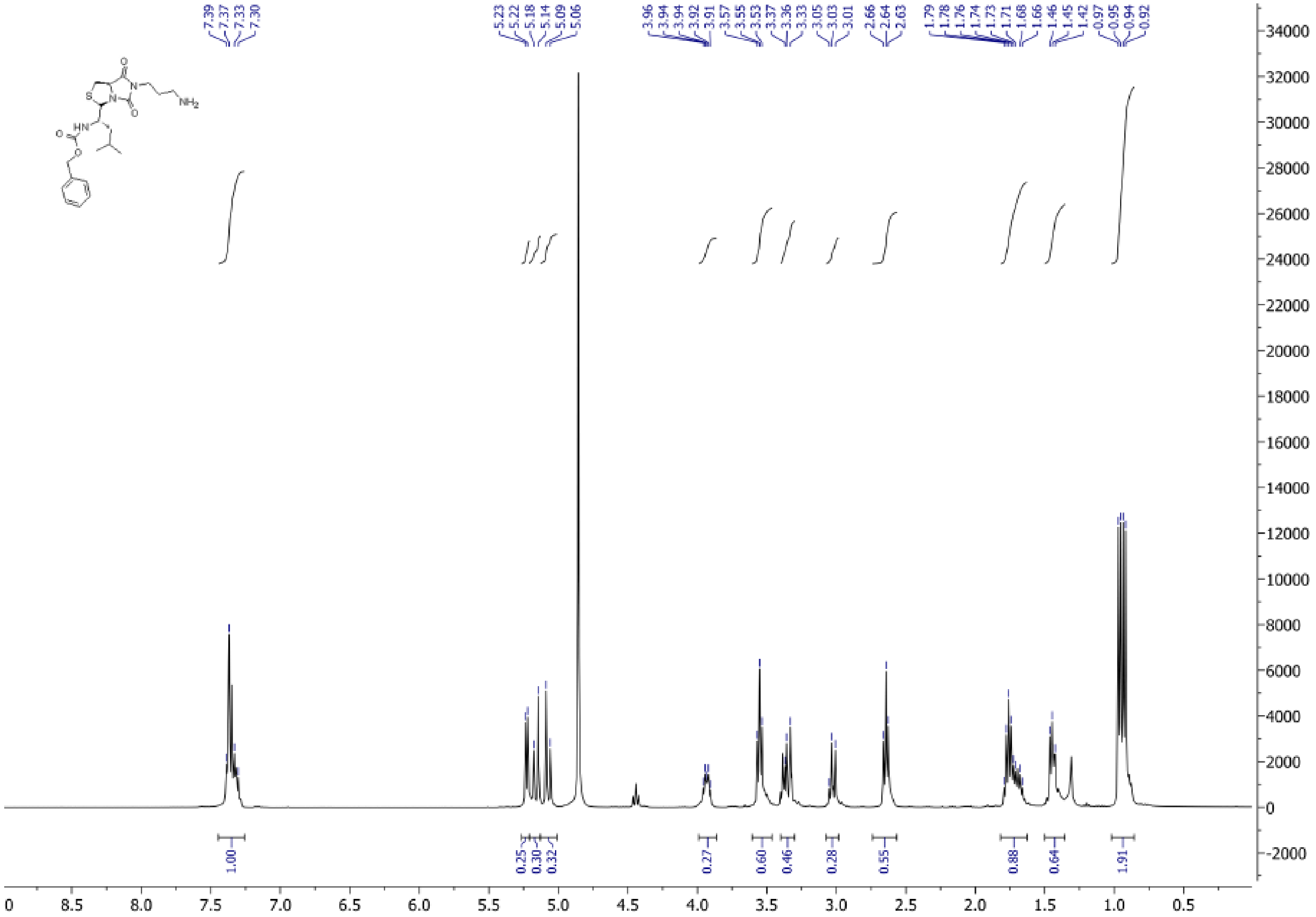

Figure S14: 1H NMR spectra of compound **17**

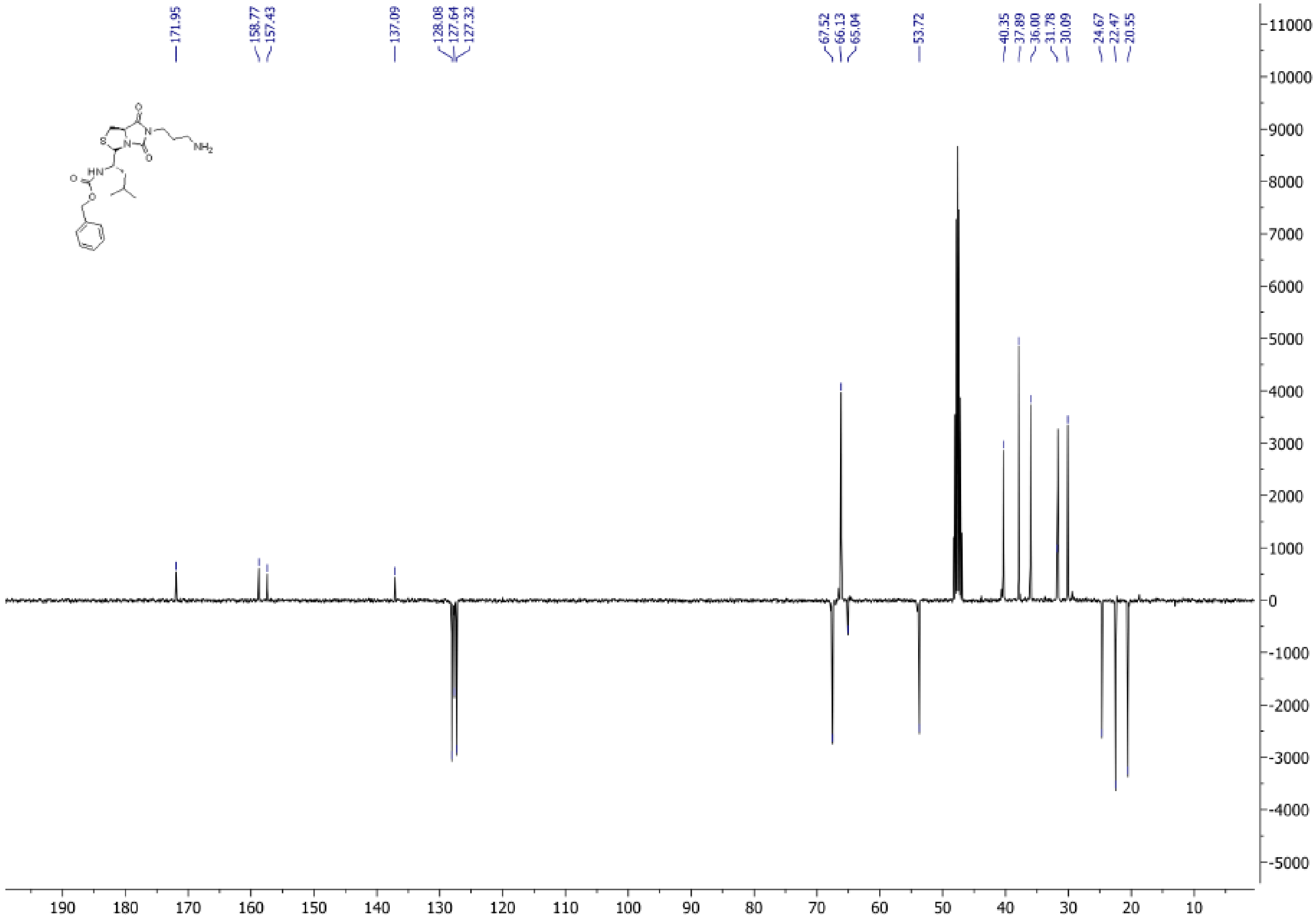

Figure S15: DEPT spectra of compound **17**

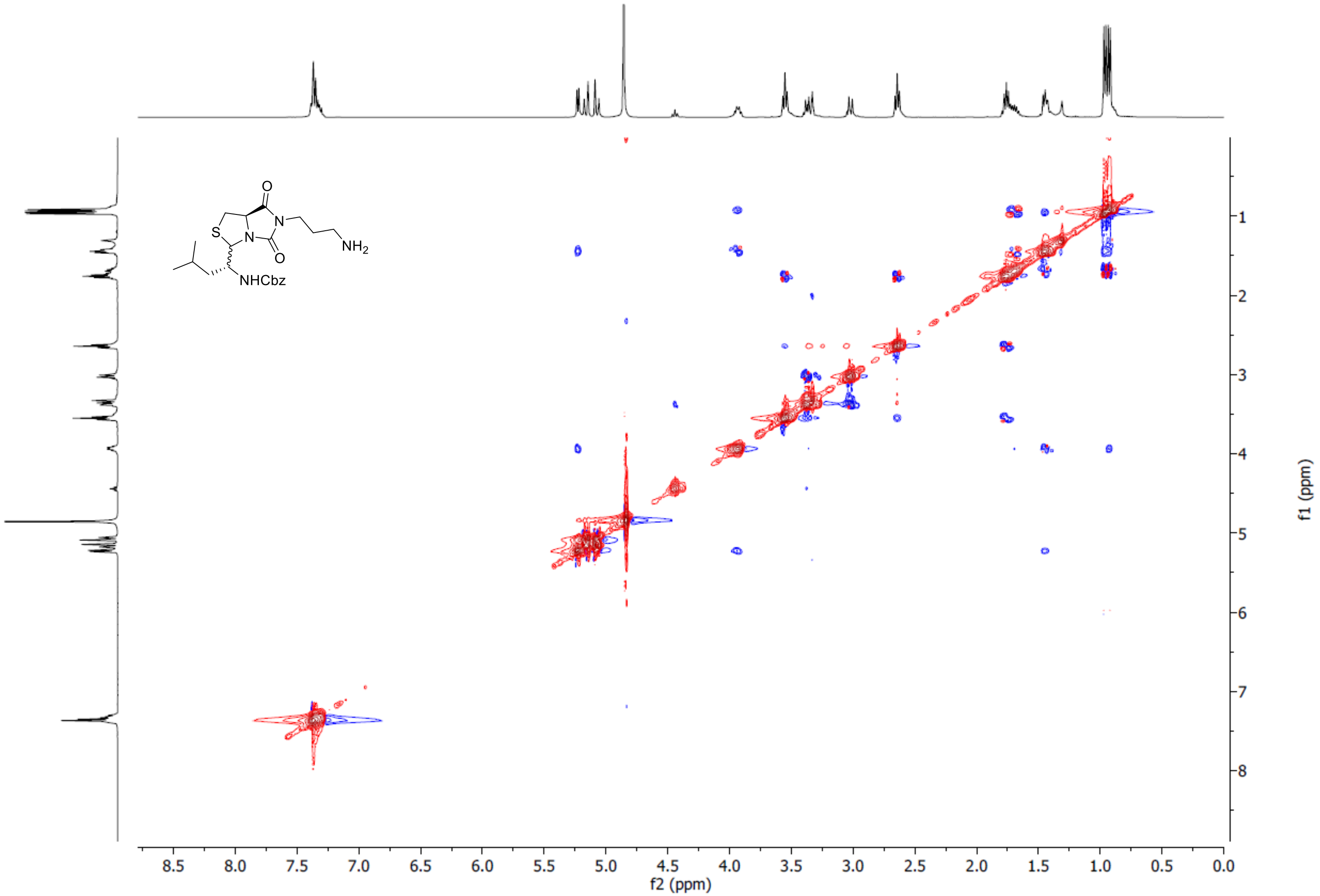

Figure S16: Roesy spectra of compound **17**

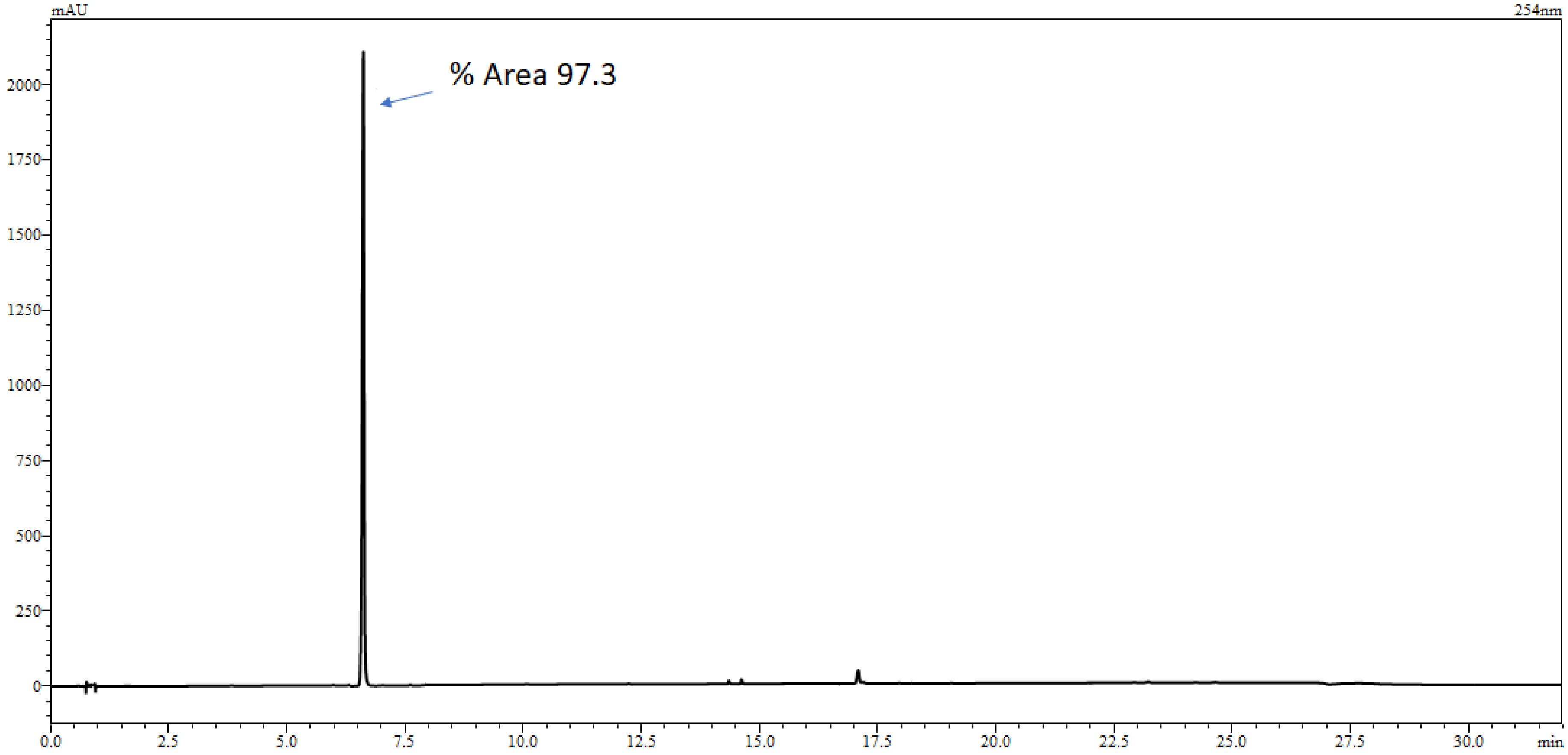

Figure S17: HPLC trace of compound **17**

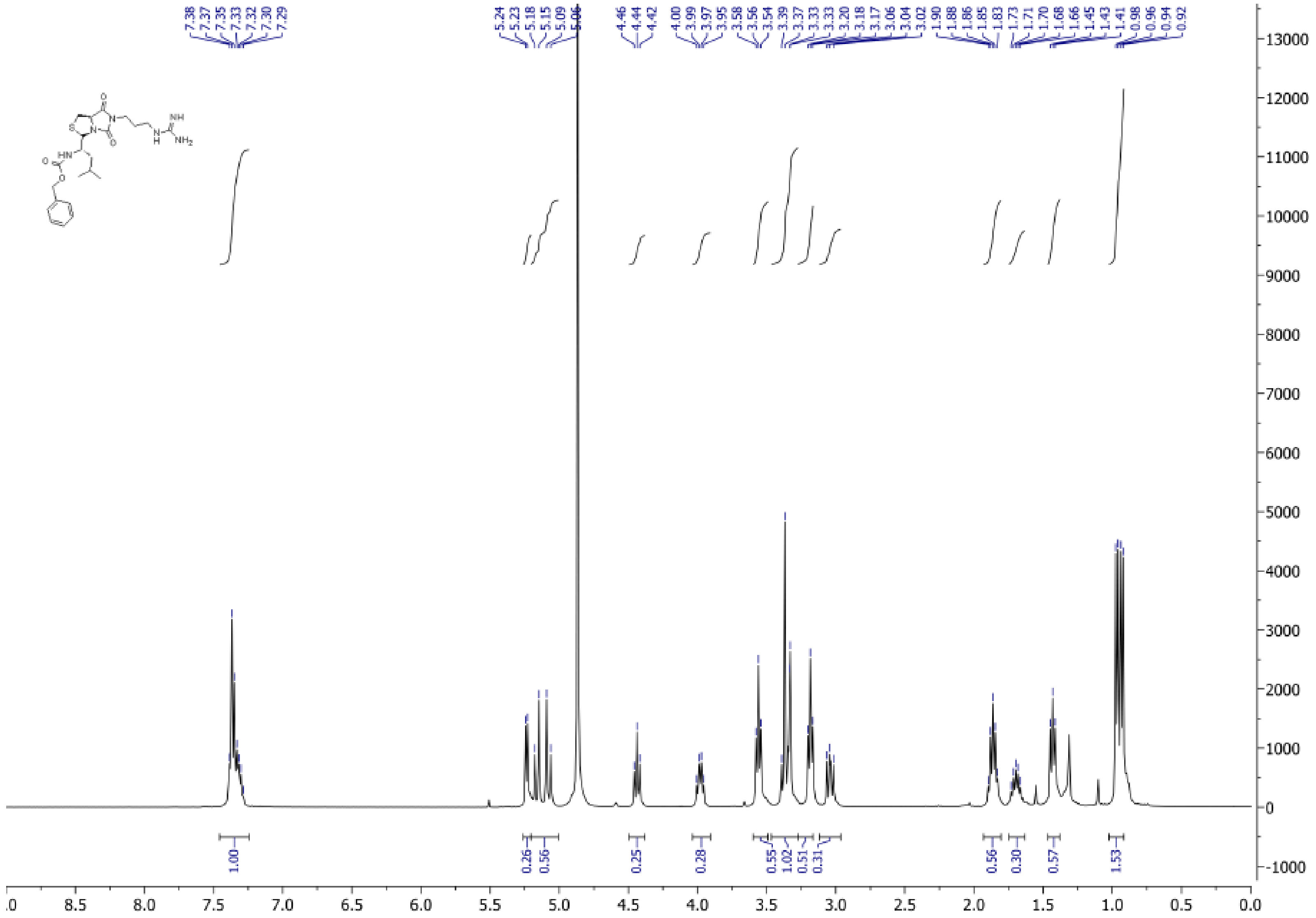

Figure S18: 1H NMR spectra of compound **18**

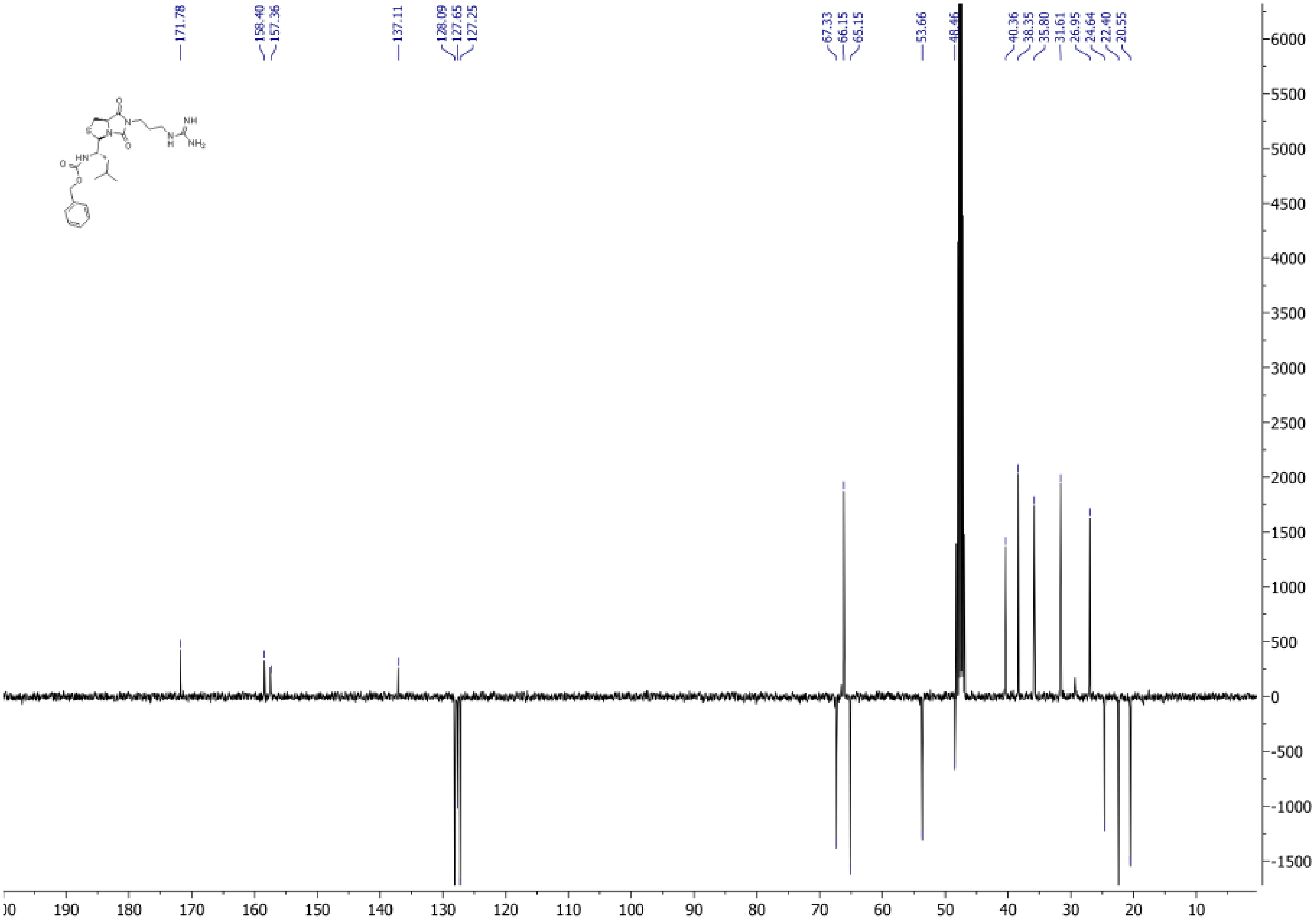

Figure S19: DEPT spectra of compound **18**

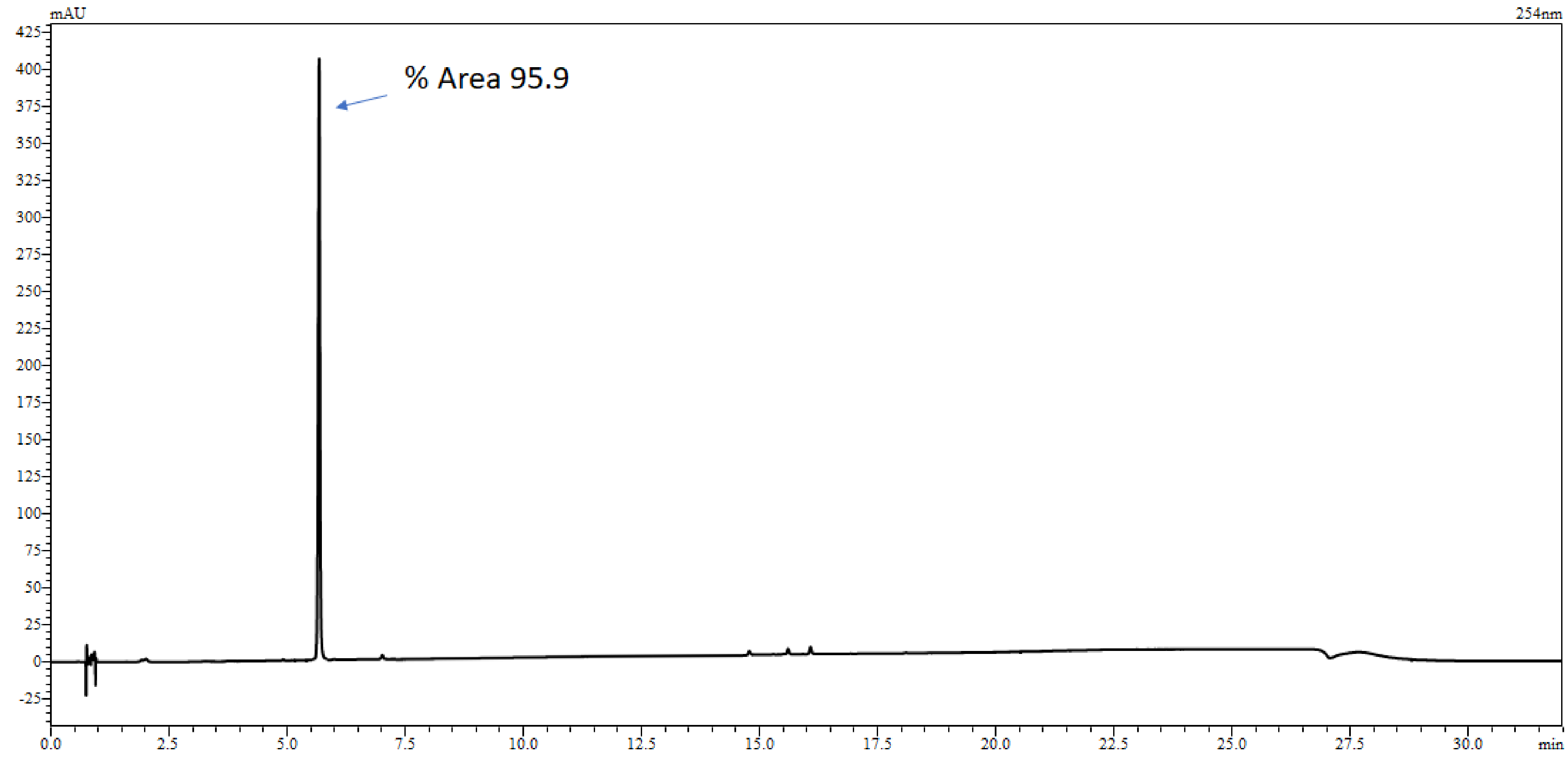

Figure S20: HPLC trace of compound **18**

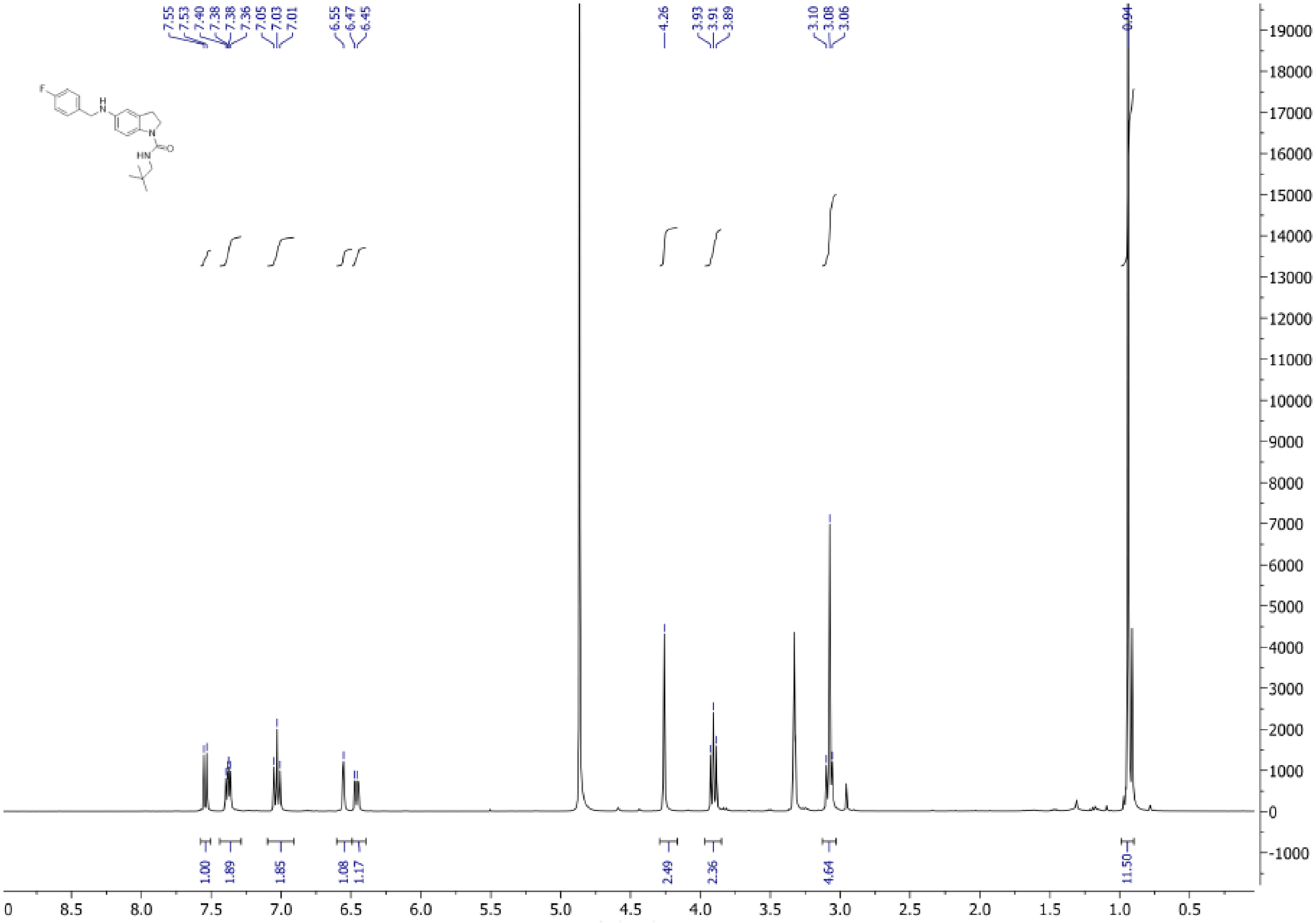

Figure S21: 1H NMR spectra of compound **21**

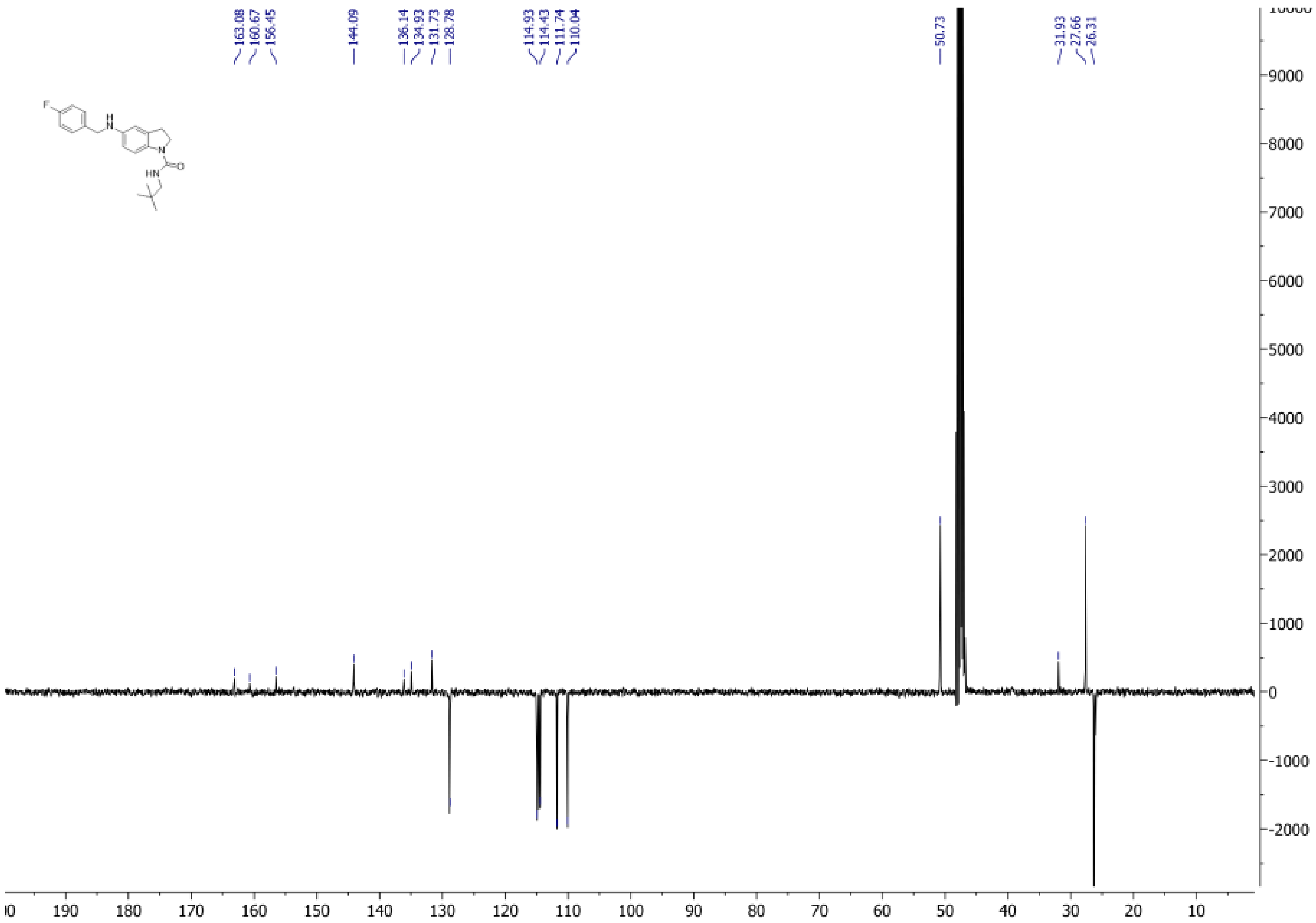

Figure S22: DEPT spectra of compound **21**

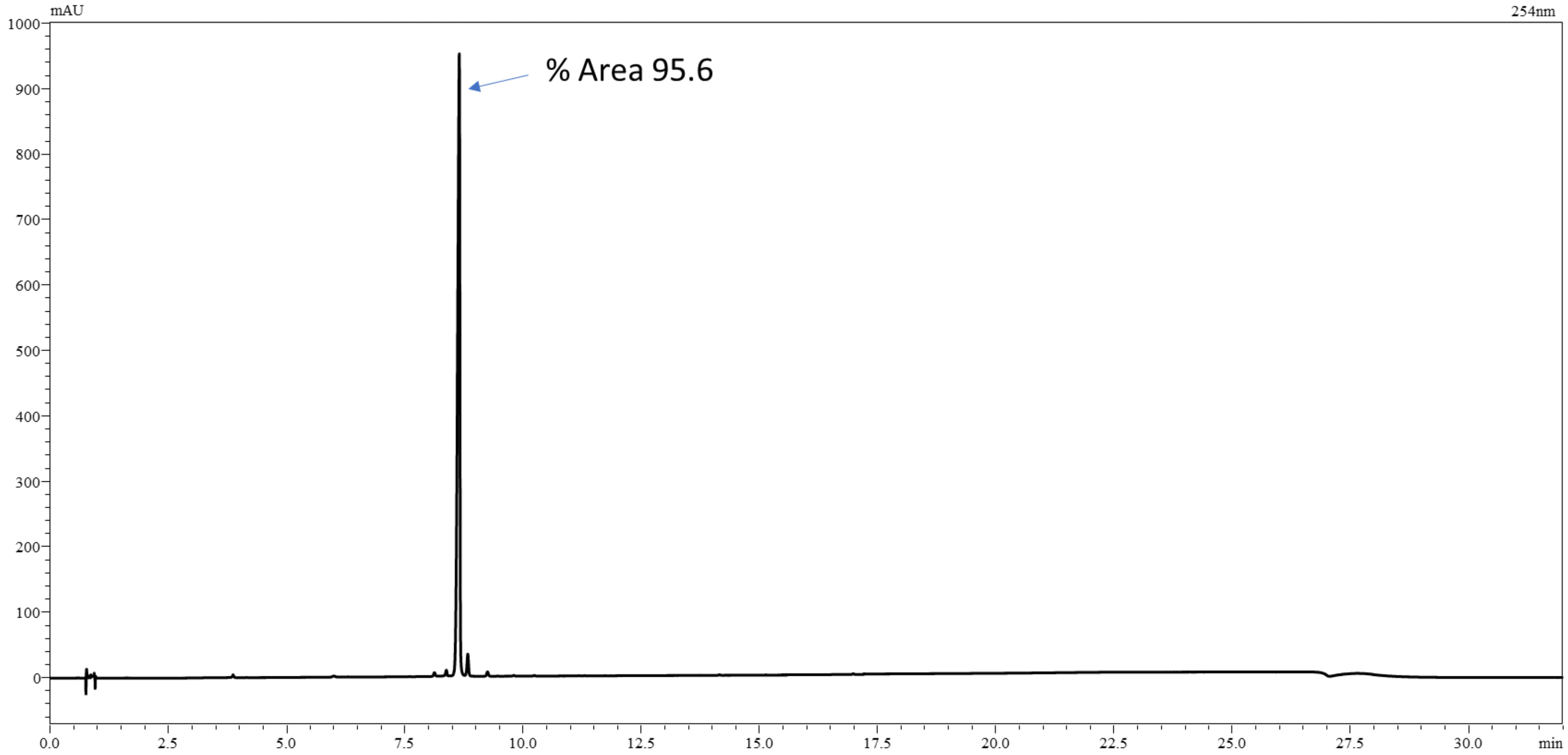

Figure S23: HPLC trace of compound **21**

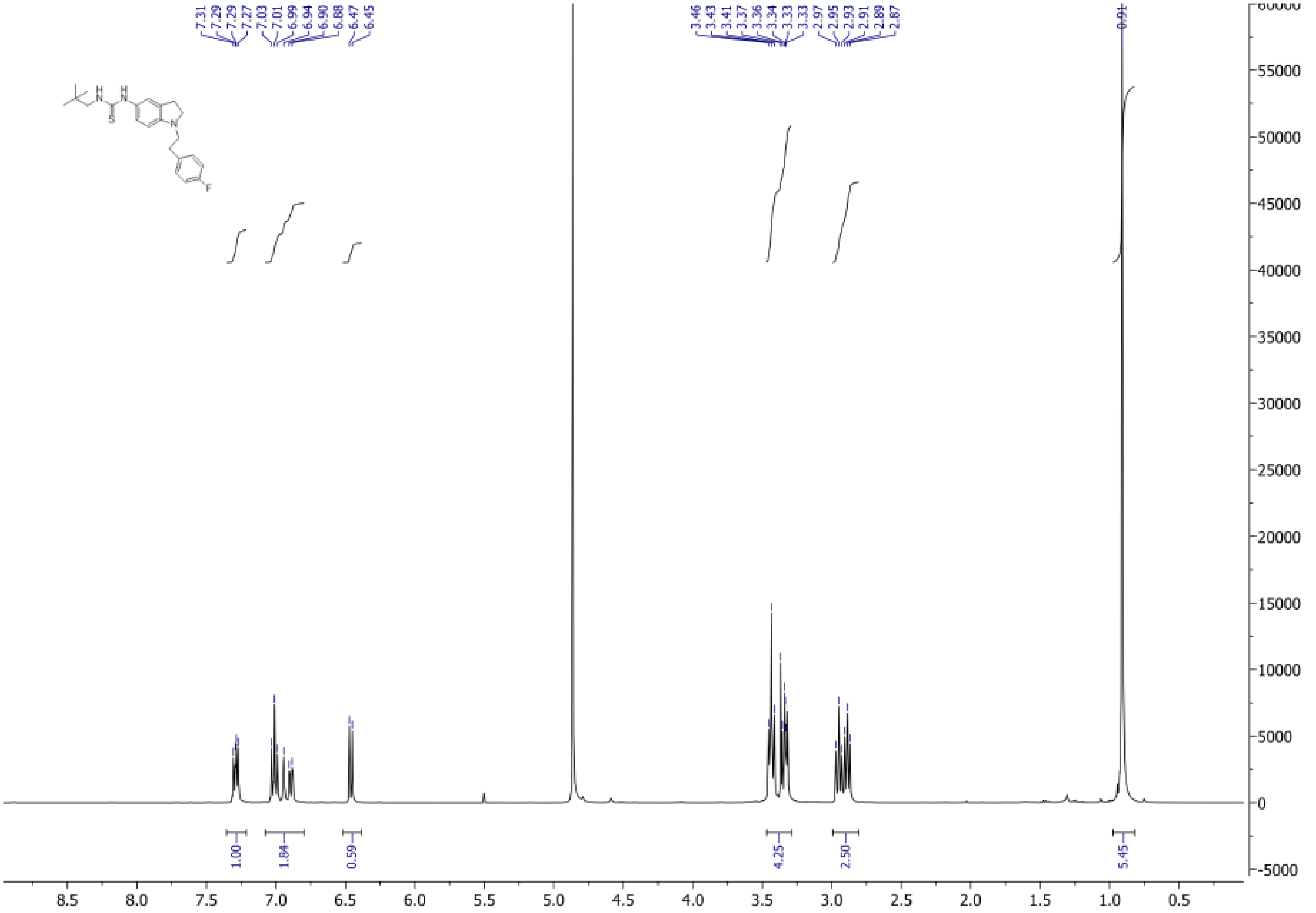

Figure S24: 1H NMR spectra of compound **44**

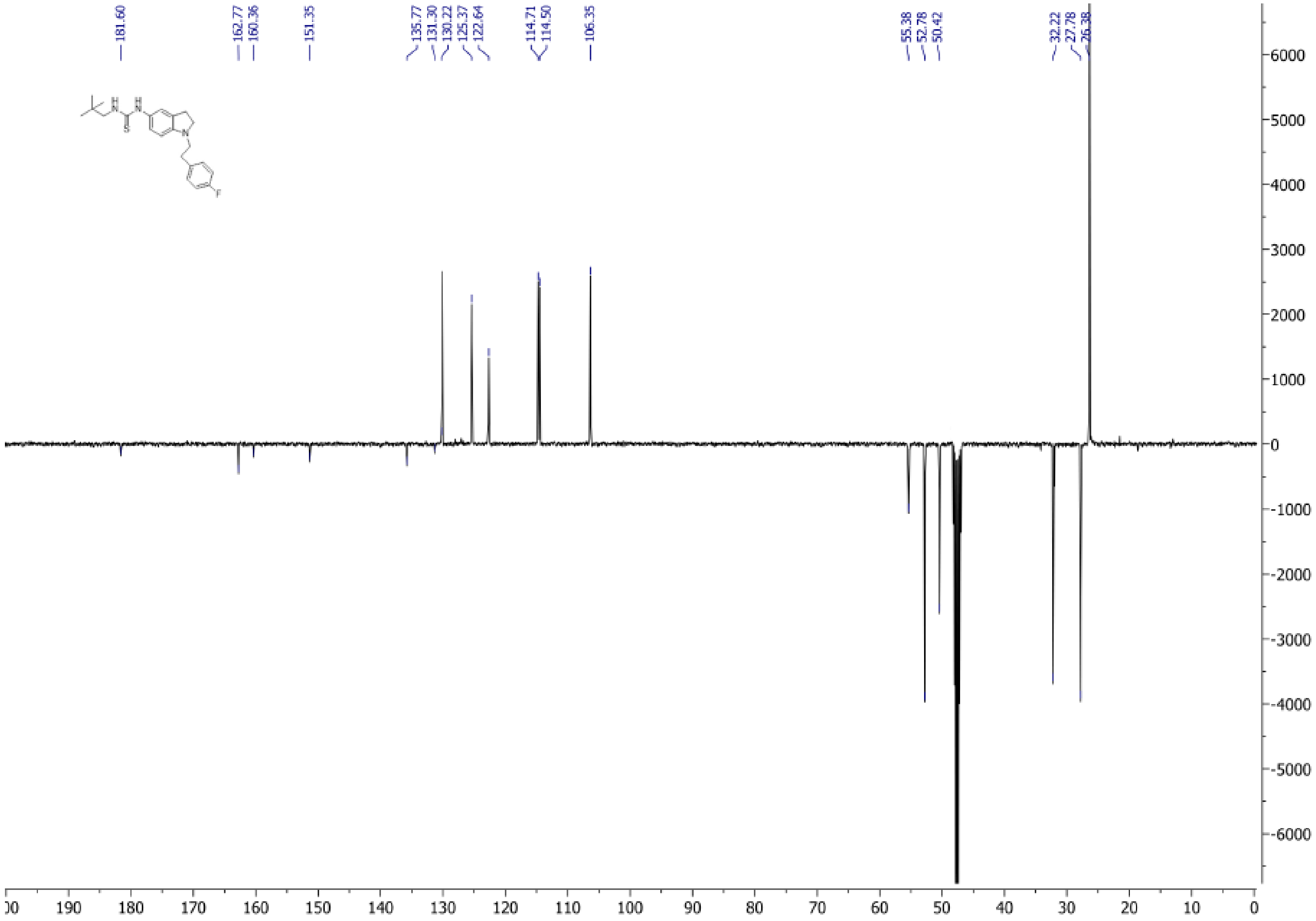

Figure S25: DEPT spectra of compound **44**

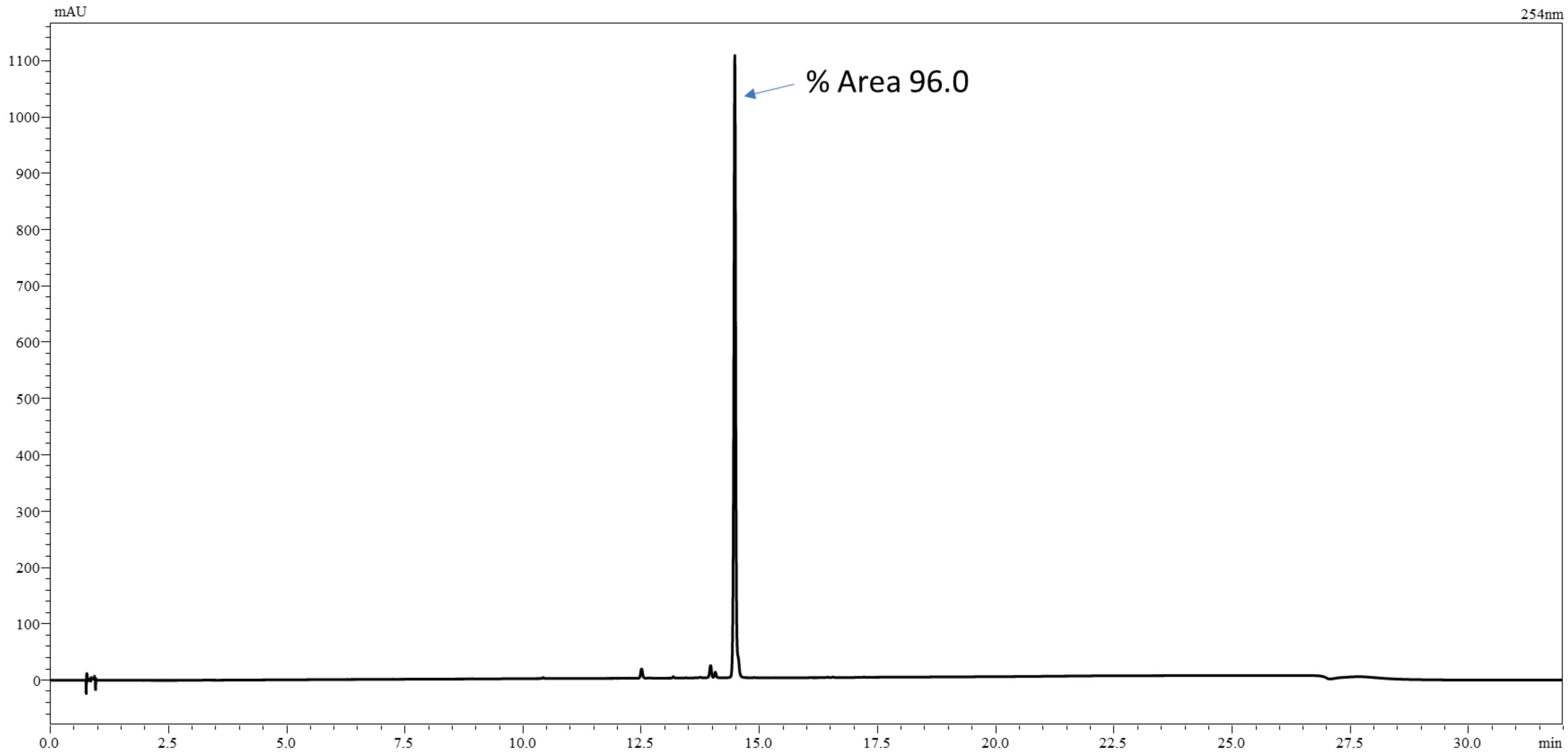

Figure S26: HPLC trace of compound **44**

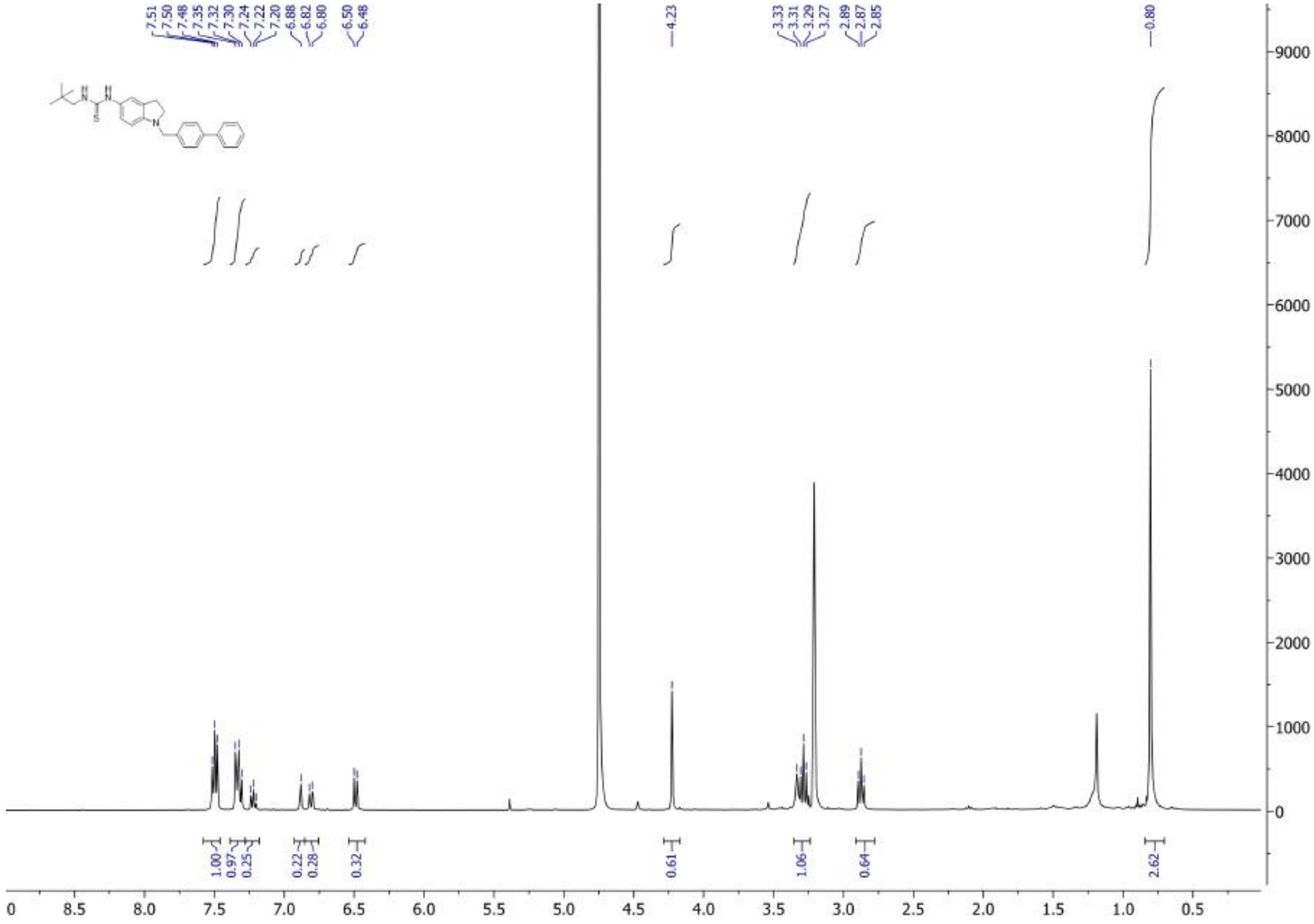

Figure S27: 1H NMR spectra of compound **45**

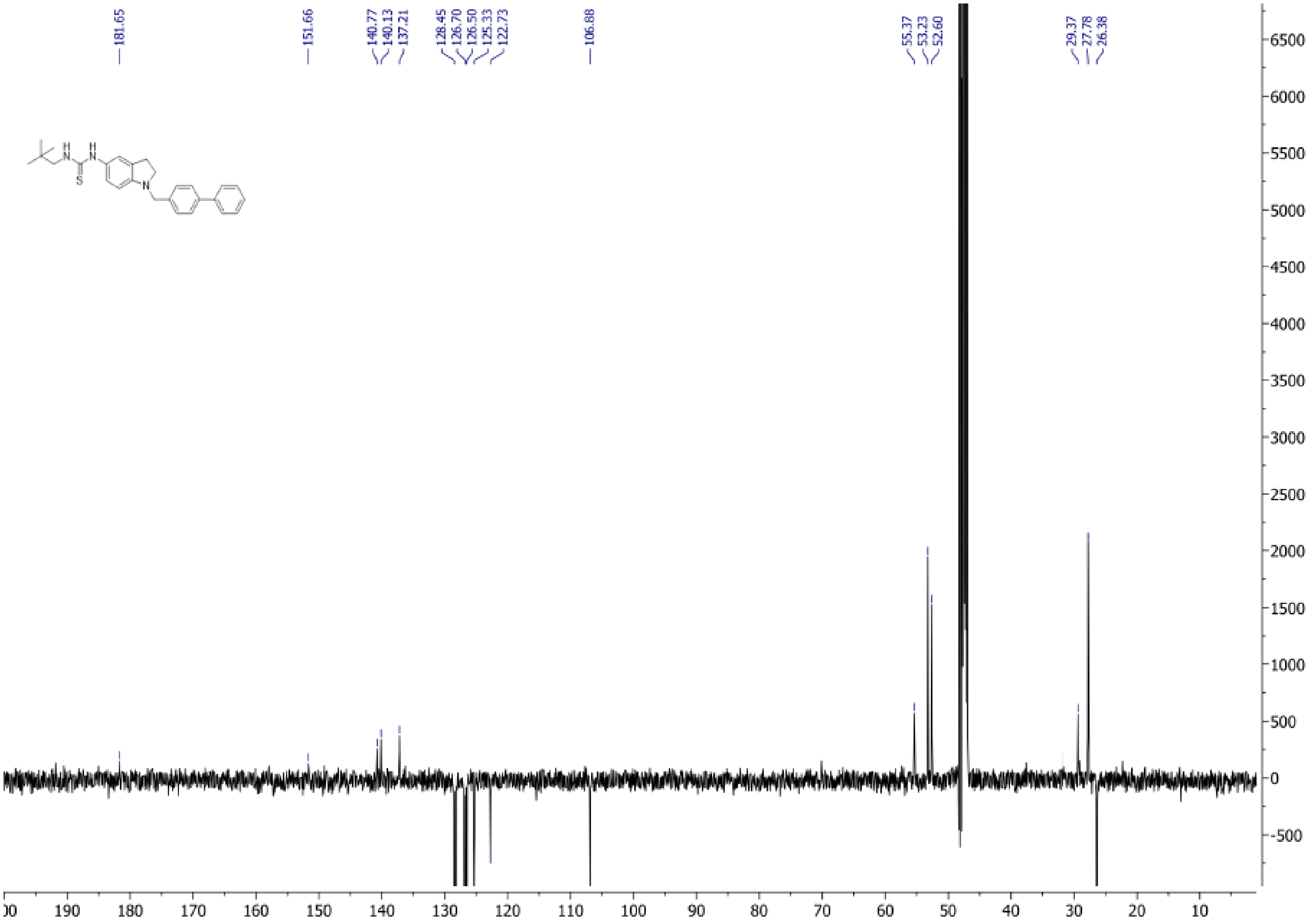

Figure S28: DEPT spectra of compound **45**

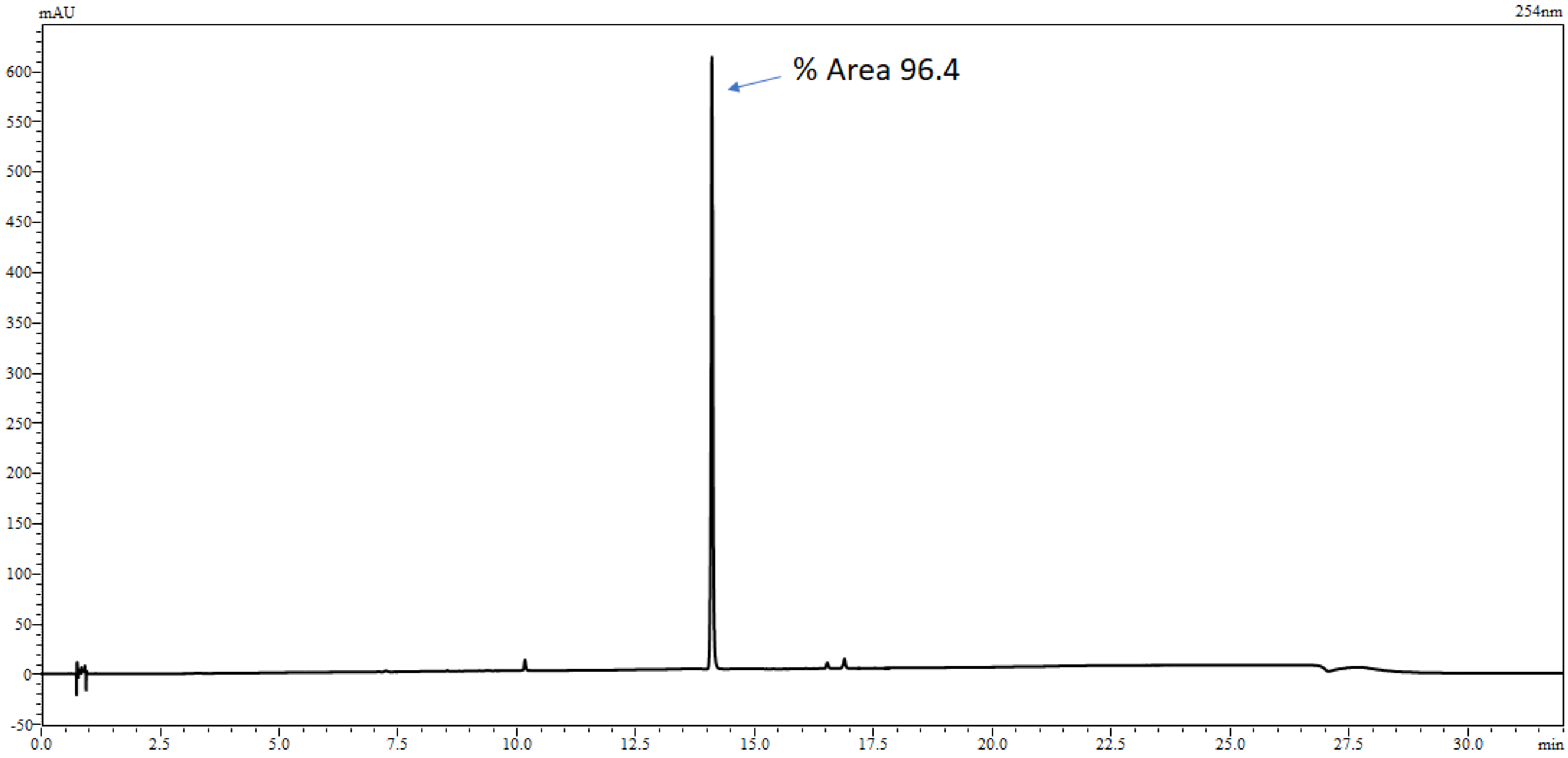

Figure S29: HPLC trace of compound **45**

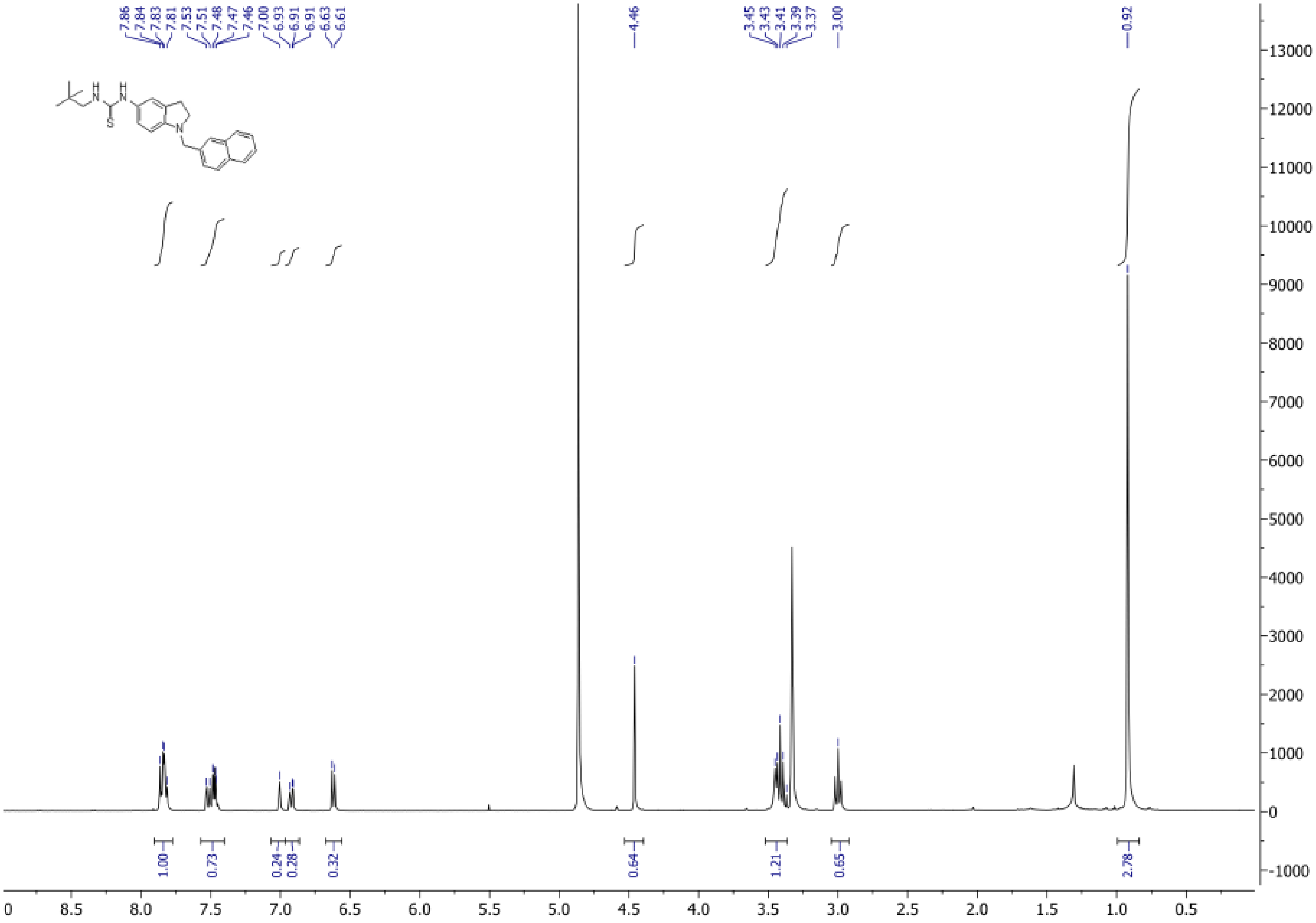

Figure S30: 1H NMR spectra of compound **46**

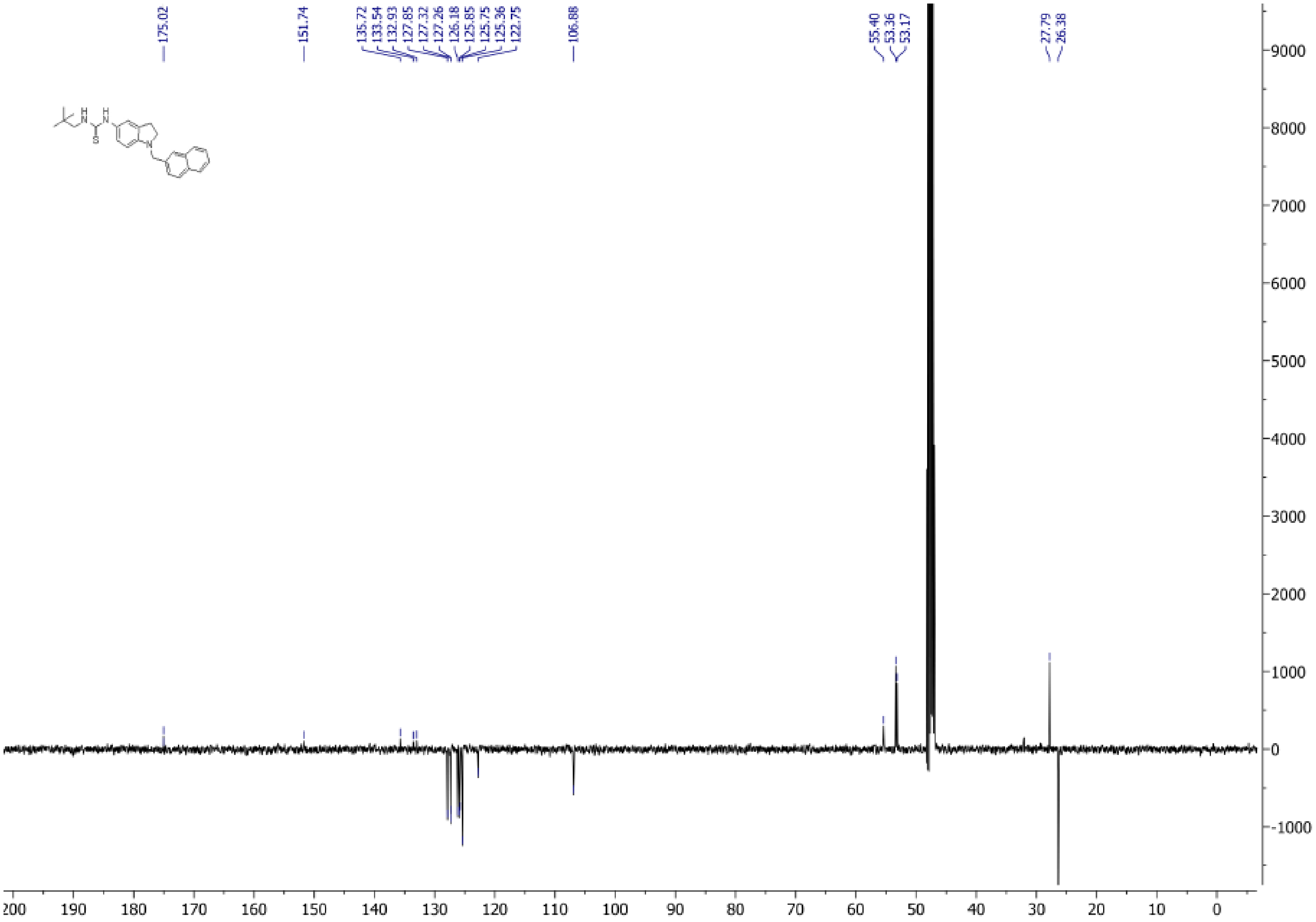

Figure S31: DEPT spectra of compound **46**

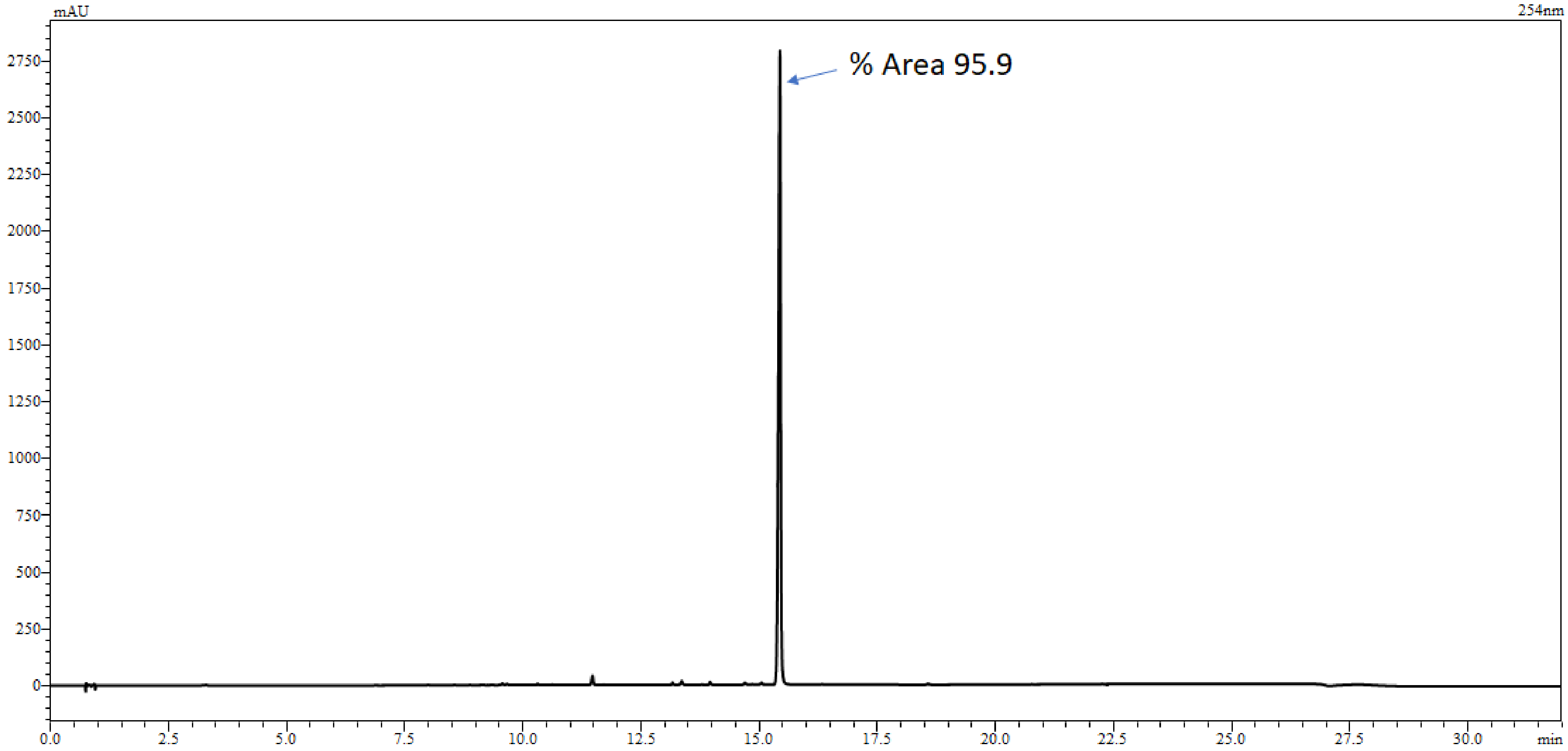

Figure S32: HPLC trace of compound **46**

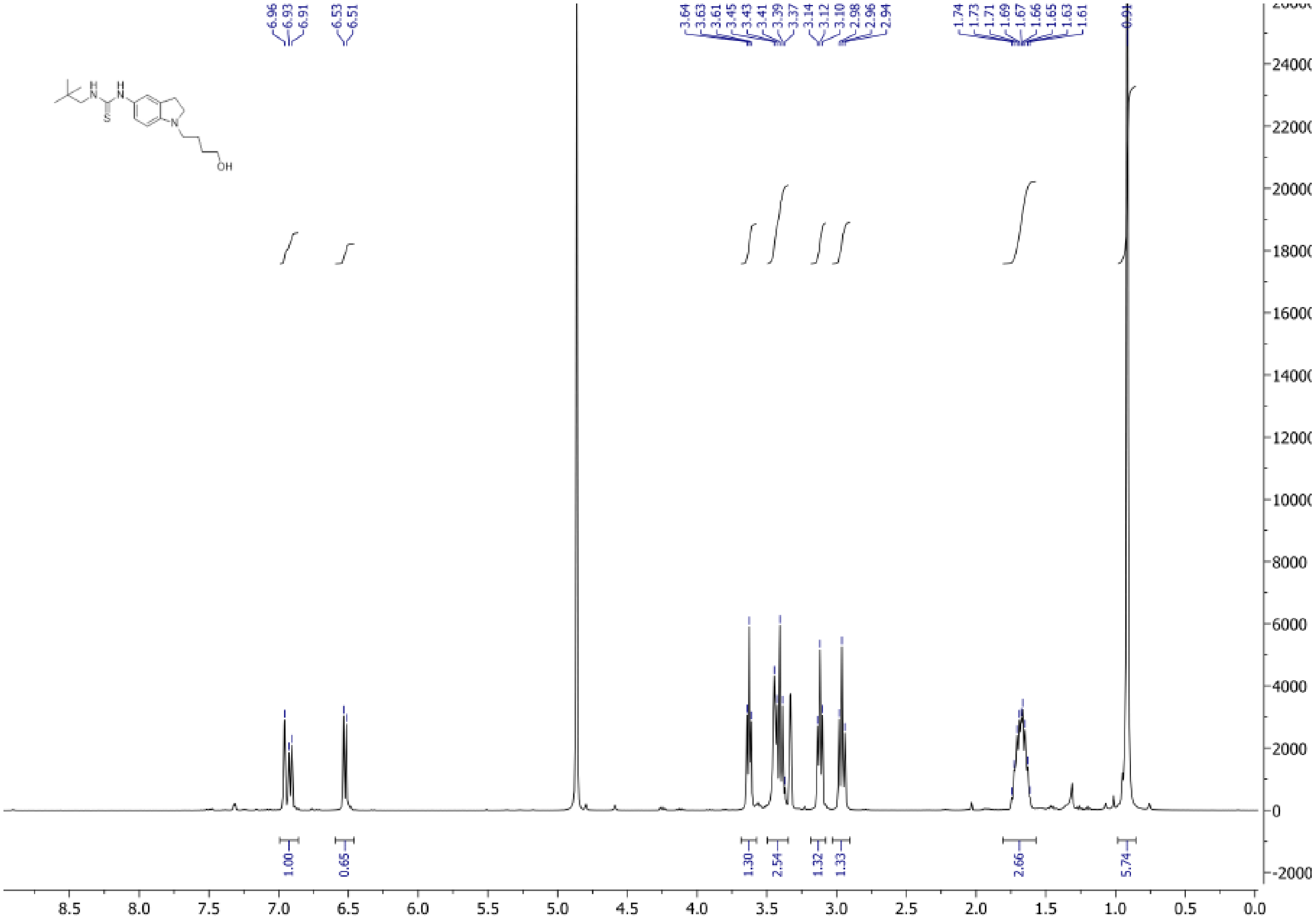

Figure S33: 1H NMR spectra of compound **48**

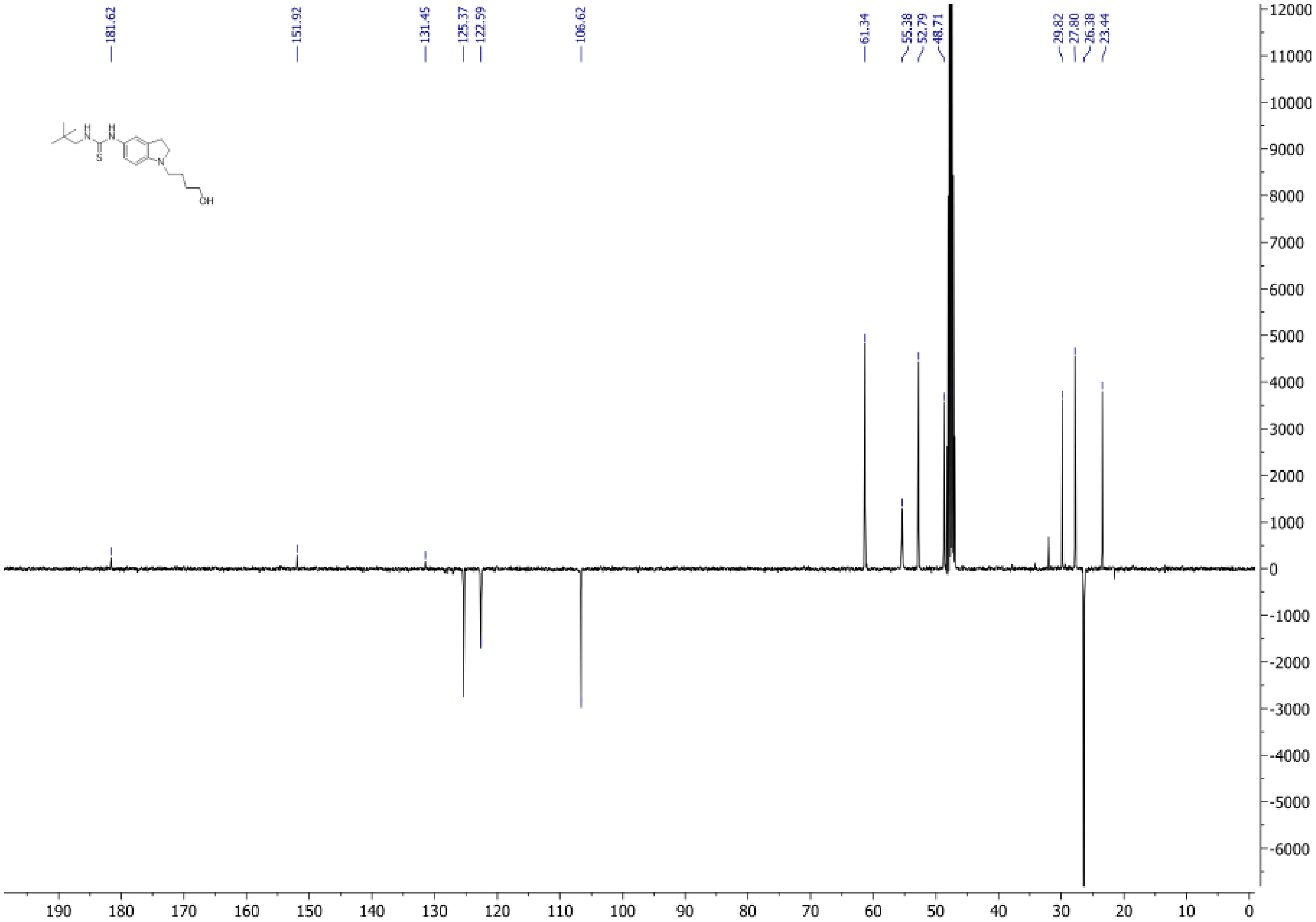

Figure S34: DEPT spectra of compound **48**

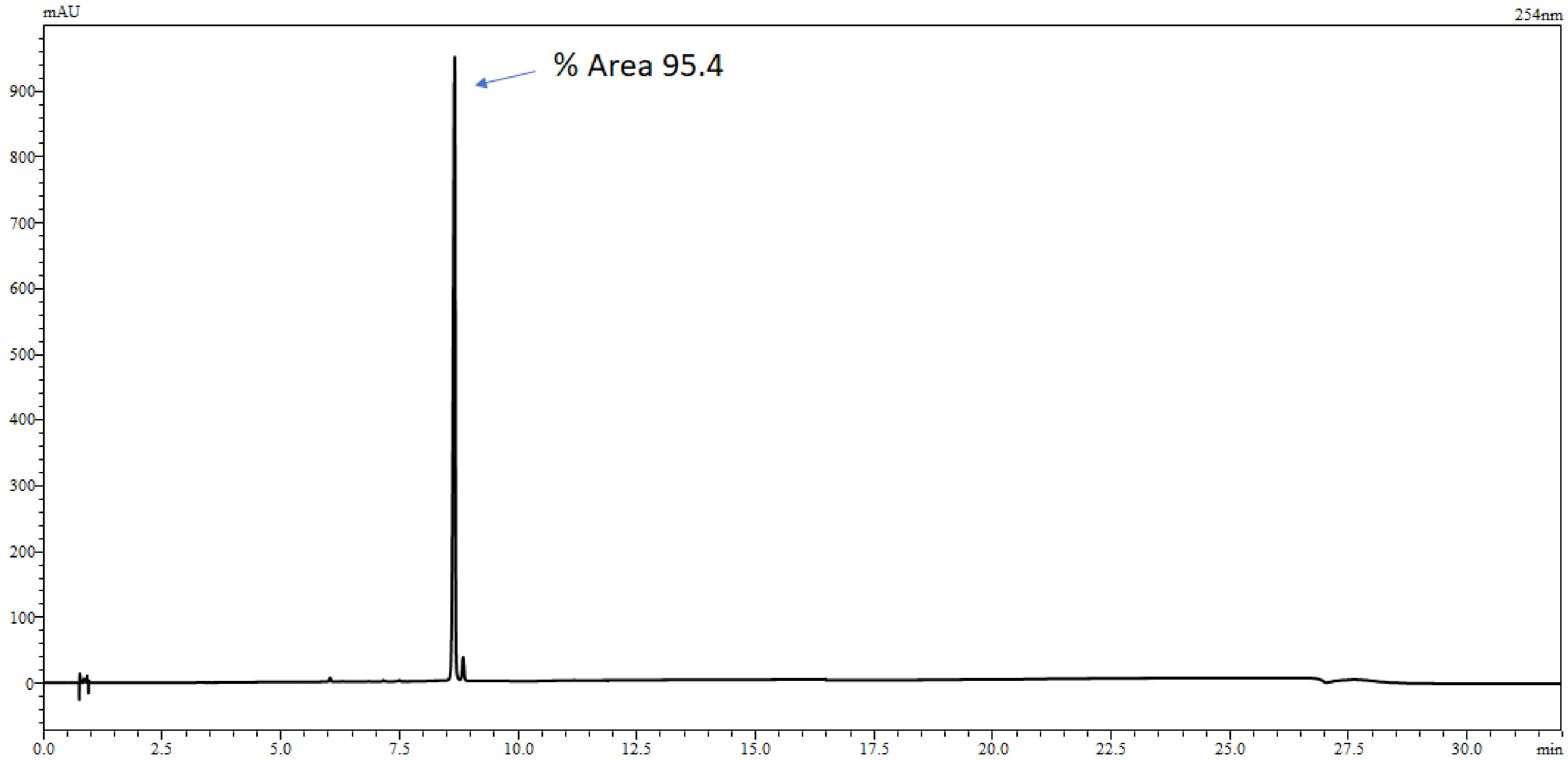

Figure S35: HPLC trace of compound **48**

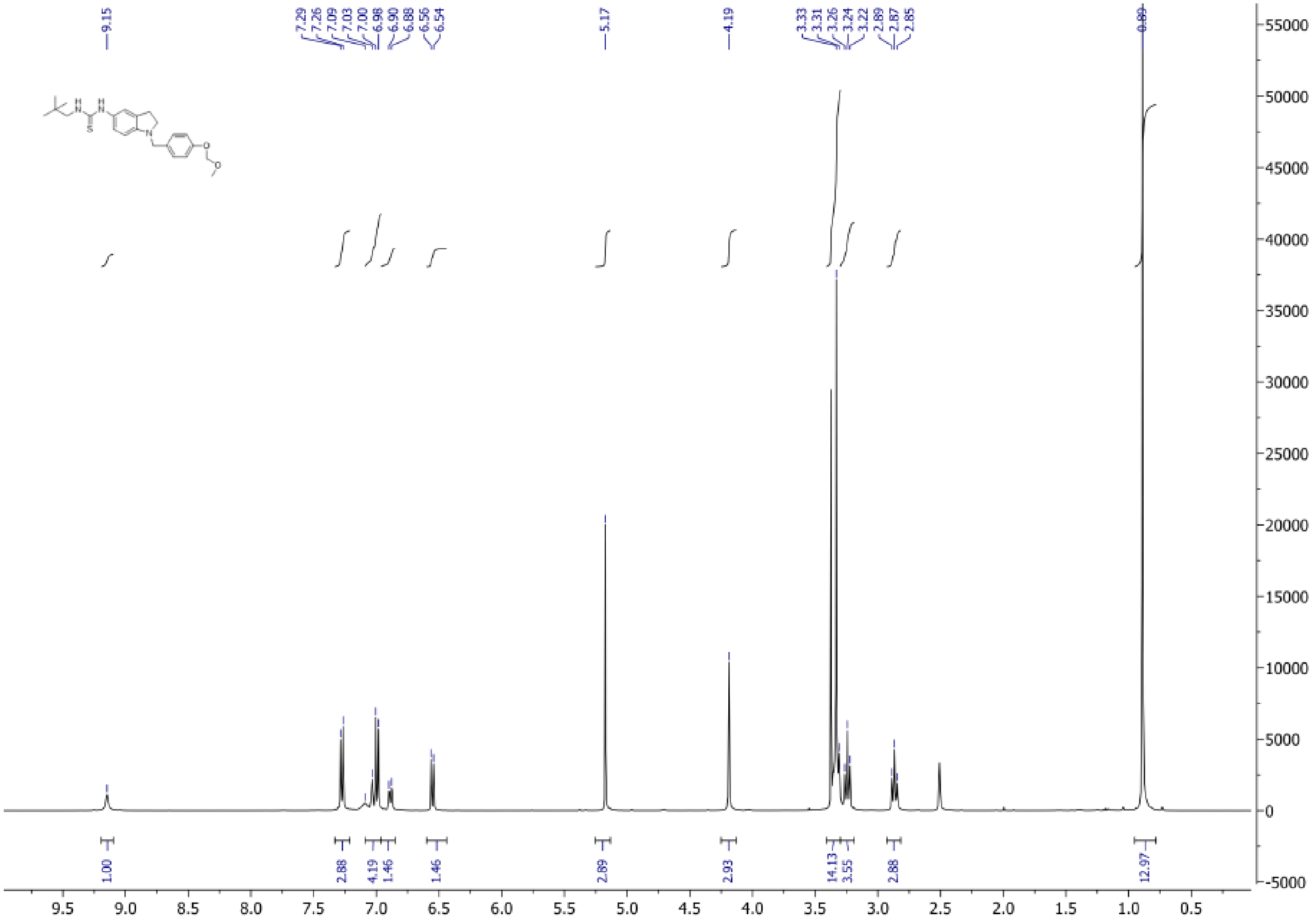

Figure S36: 1H NMR spectra of compound **49**

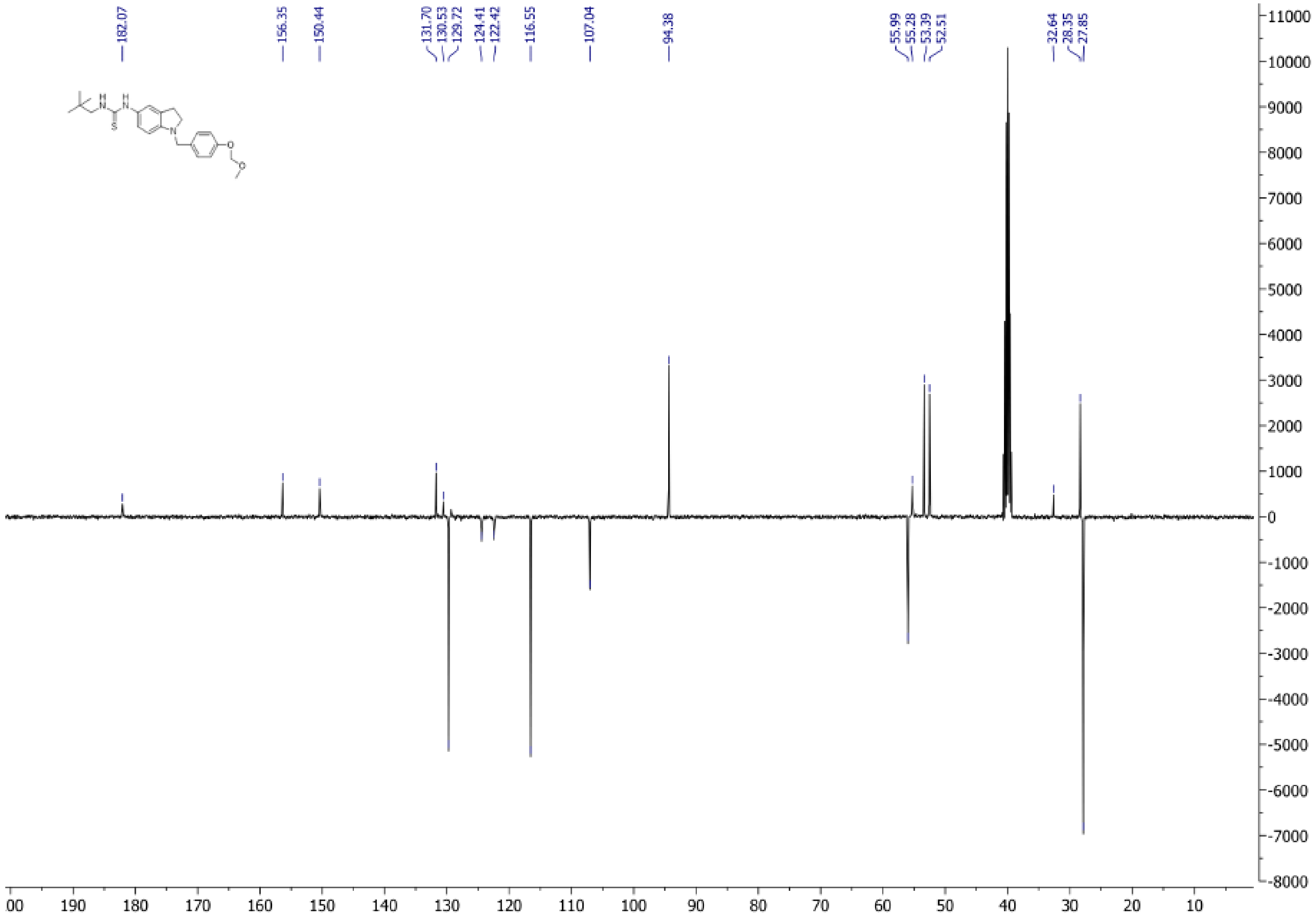

Figure S37: DEPT spectra of compound **49**

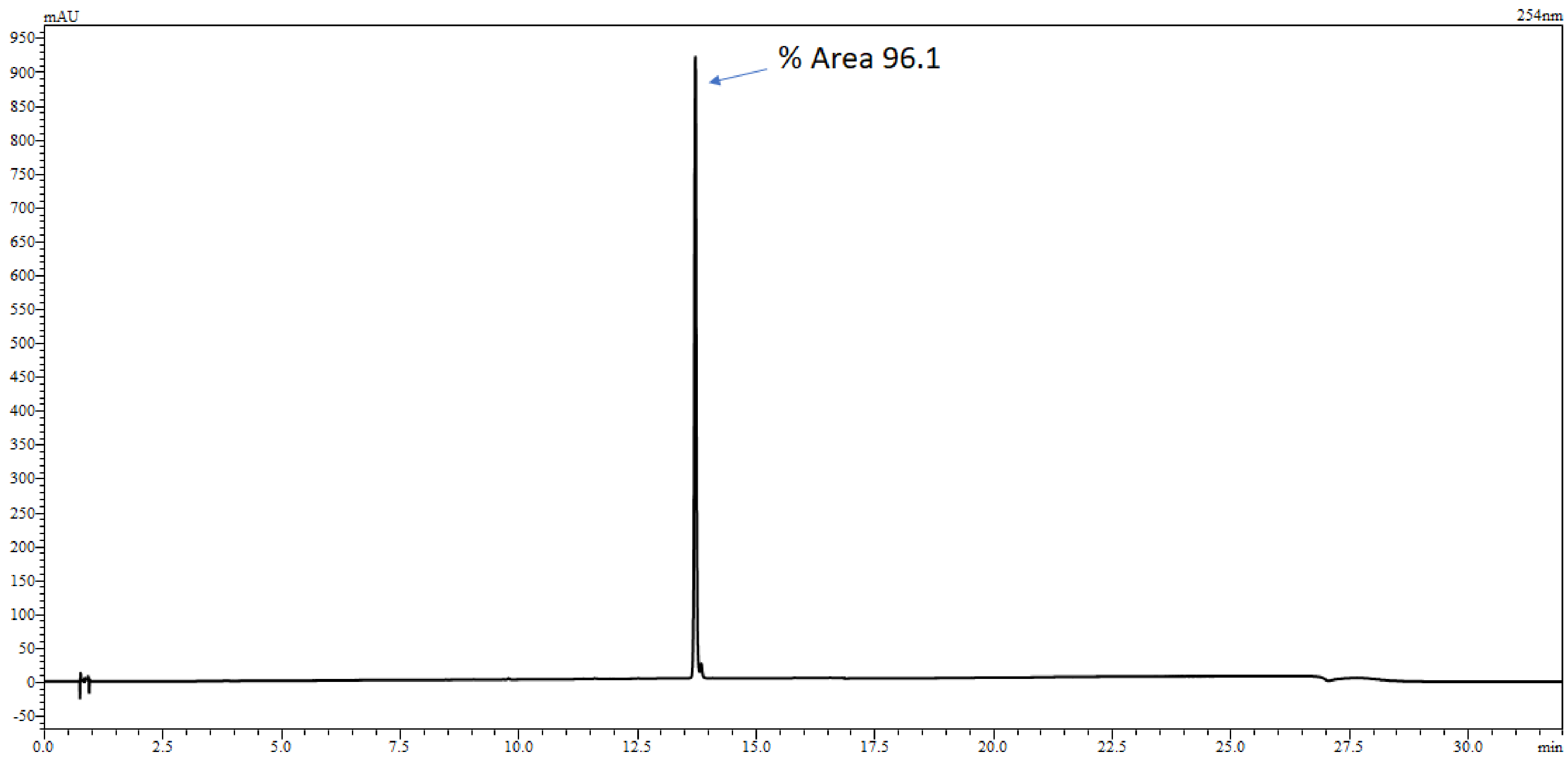

Figure S38: HPLC trace of compound **49**

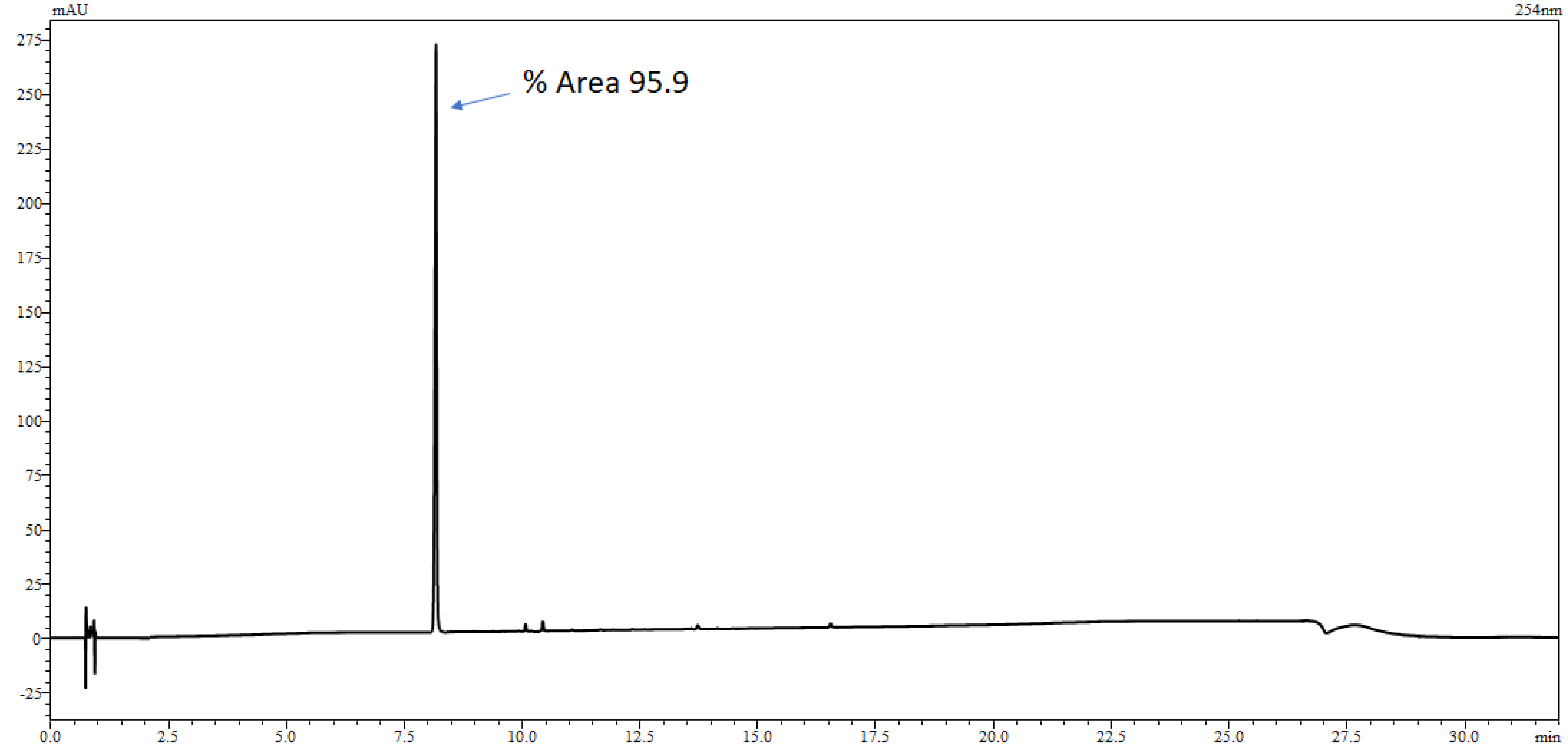

Figure S39: HPLC trace of compound **50**

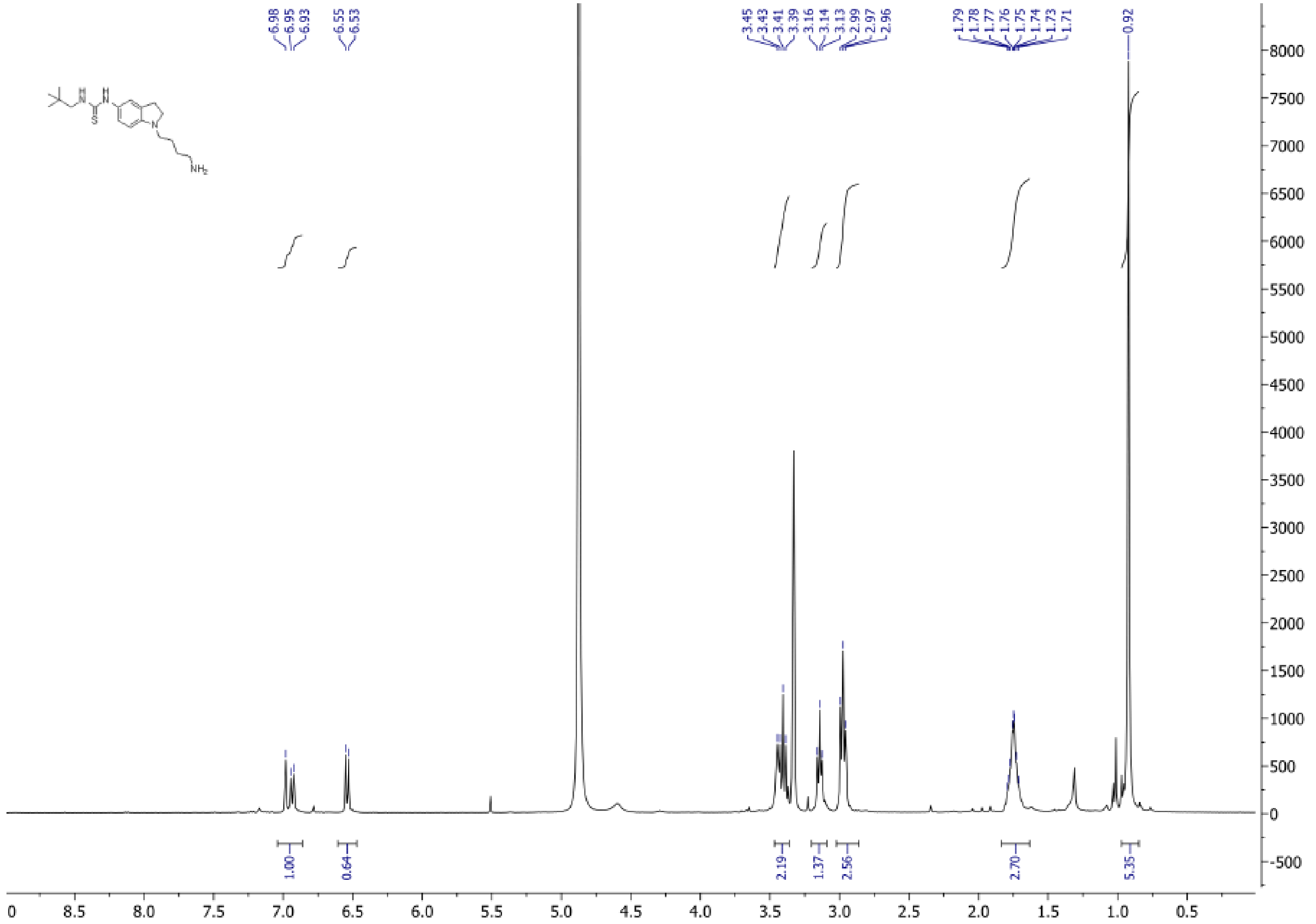

Figure S40: 1H NMR spectra of compound **51**

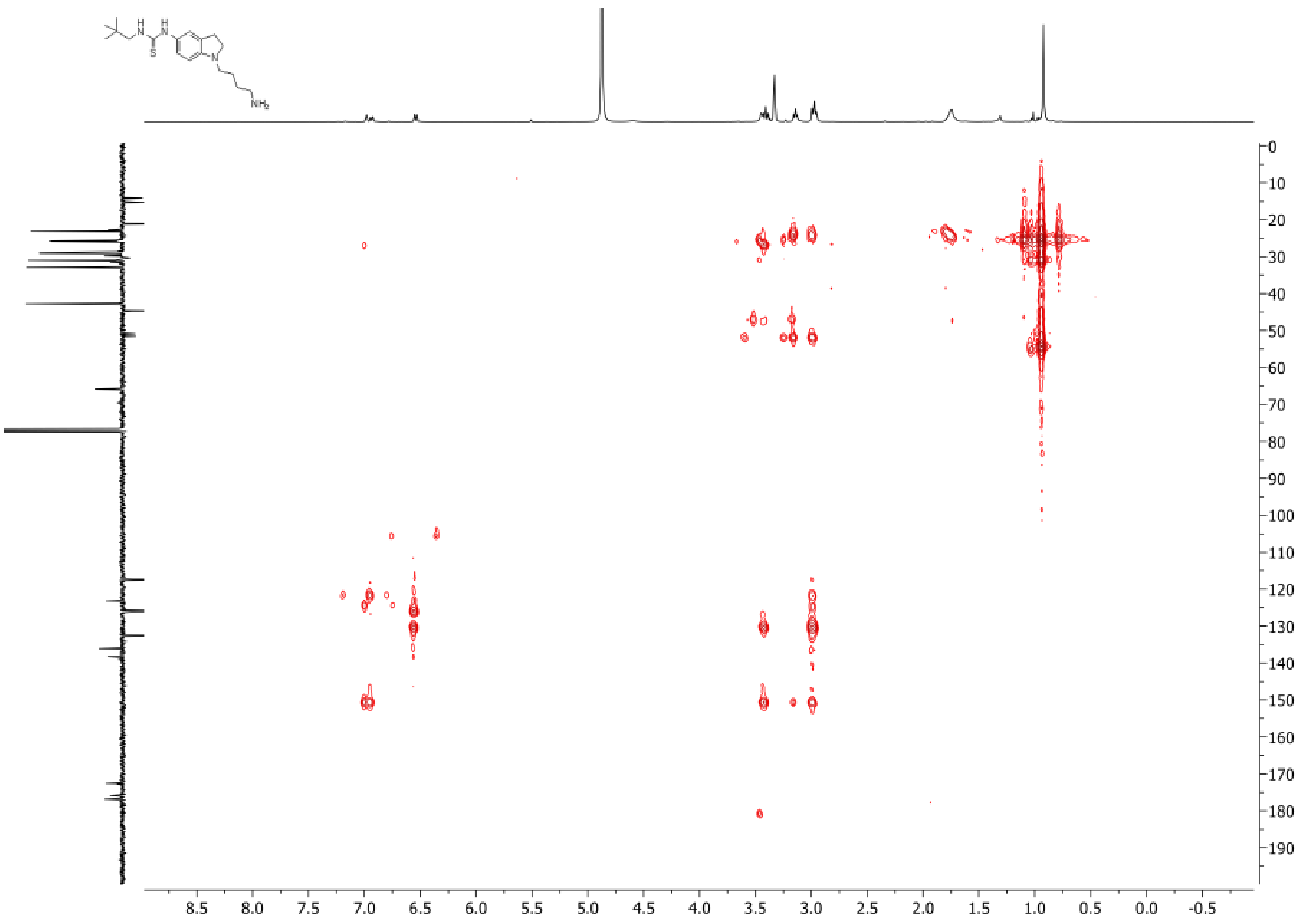

Figure S41: HMBC spectra of compound **51**

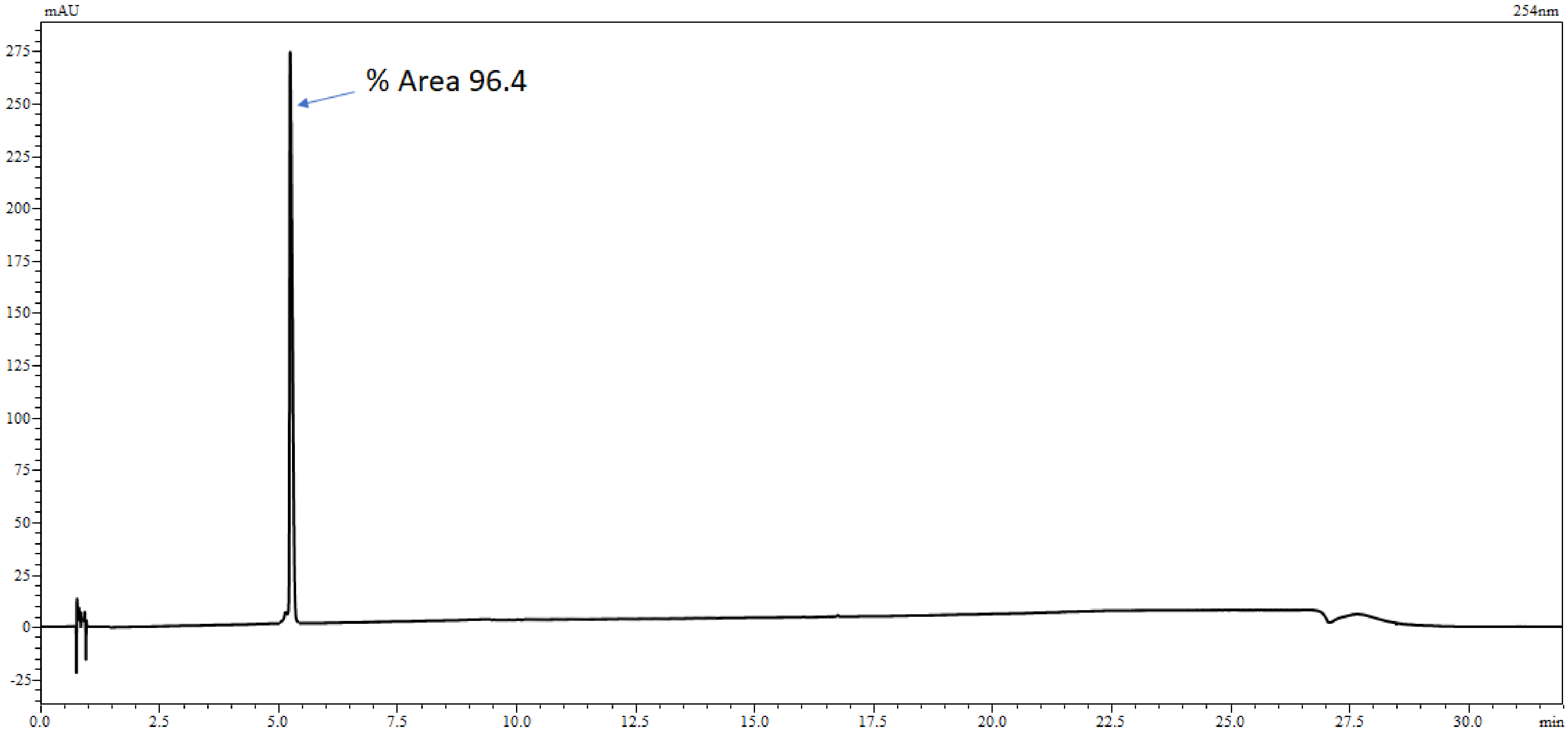

Figure S42: HPLC trace of compound **51**

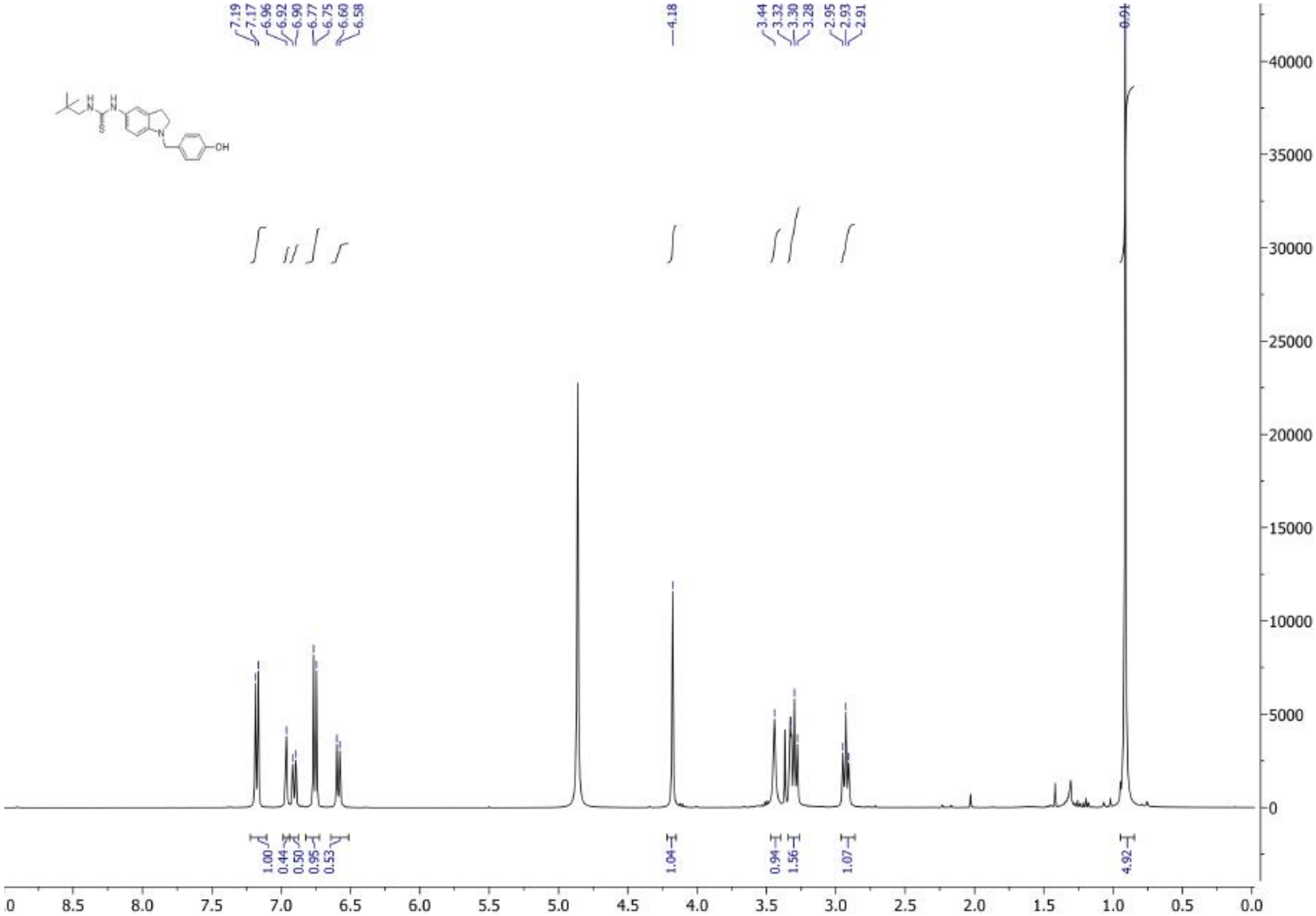

Figure S43: 1H NMR spectra of compound **52**

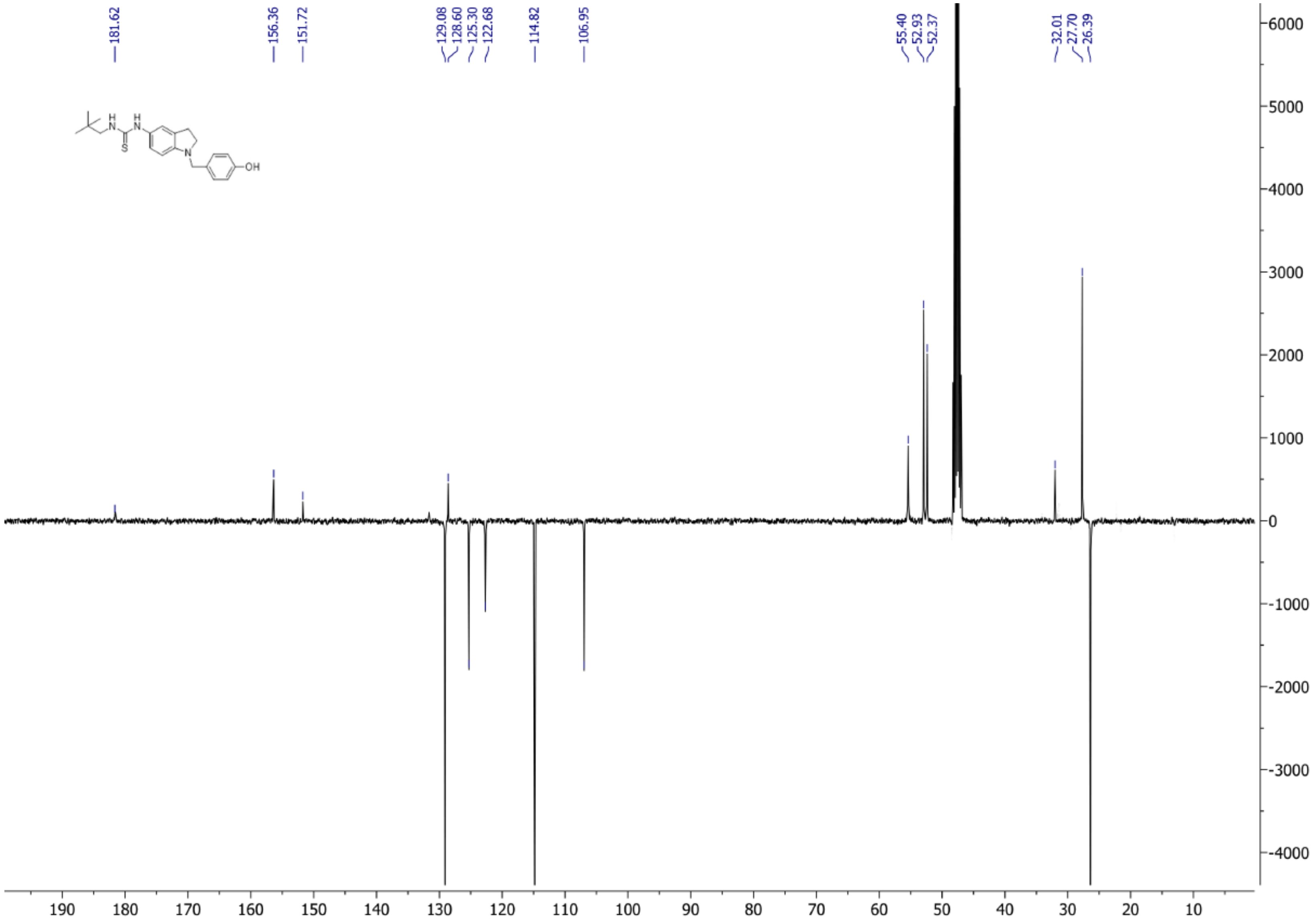

Figure S44: DEPT spectra of compound **52**

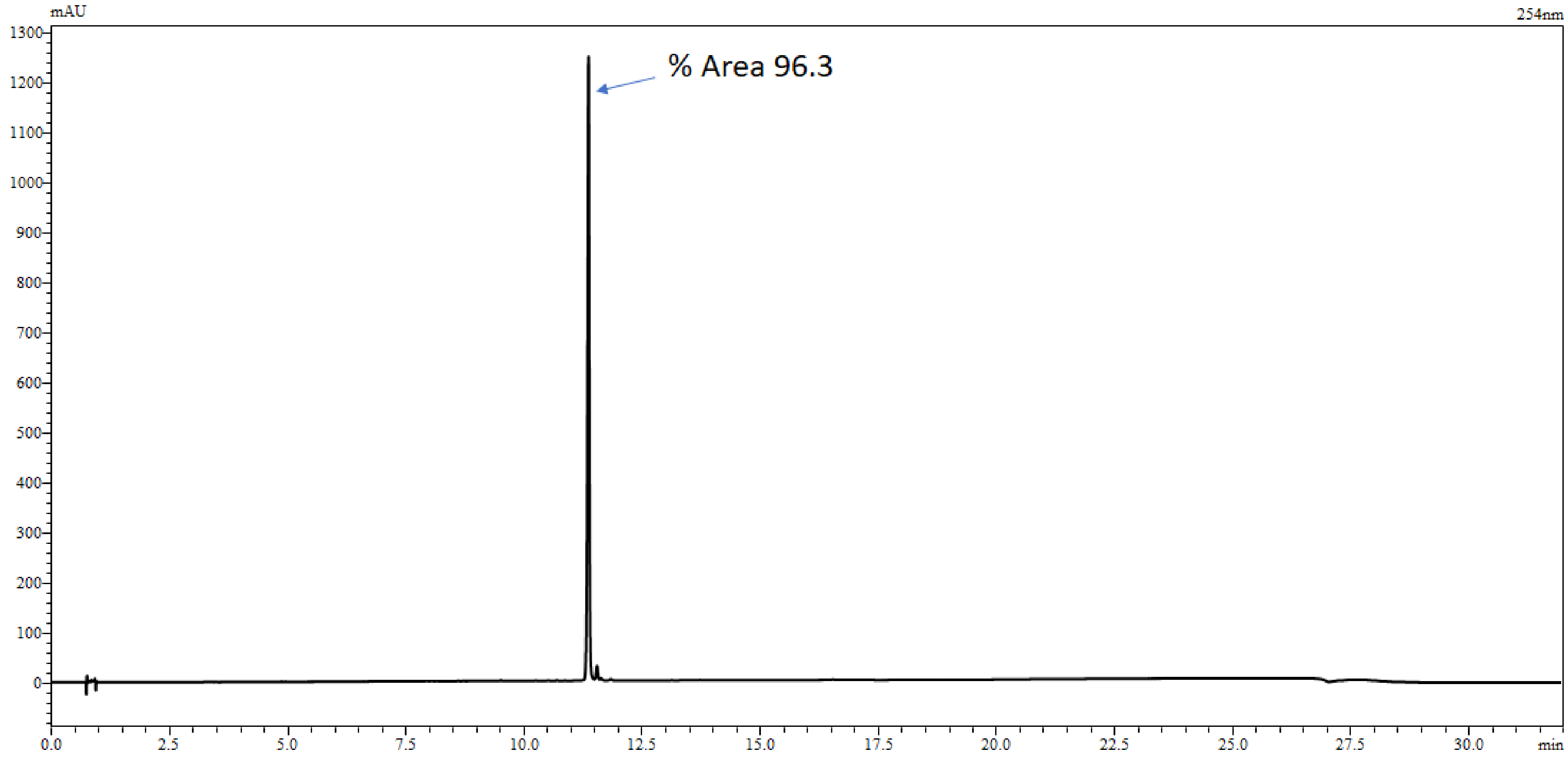

Figure S45: HPLC trace of compound **52**

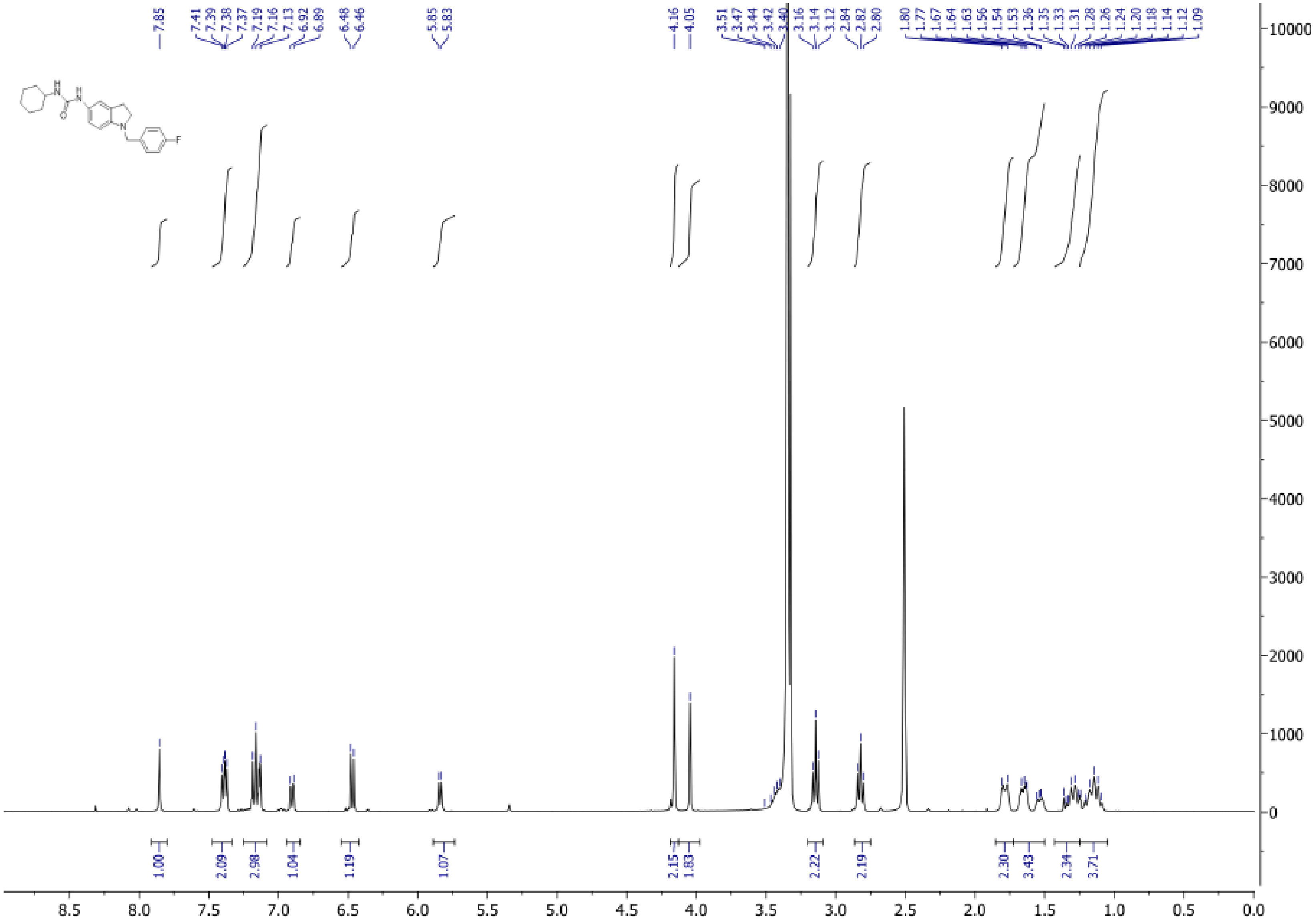

Figure S46: 1H NMR spectra of compound **53**

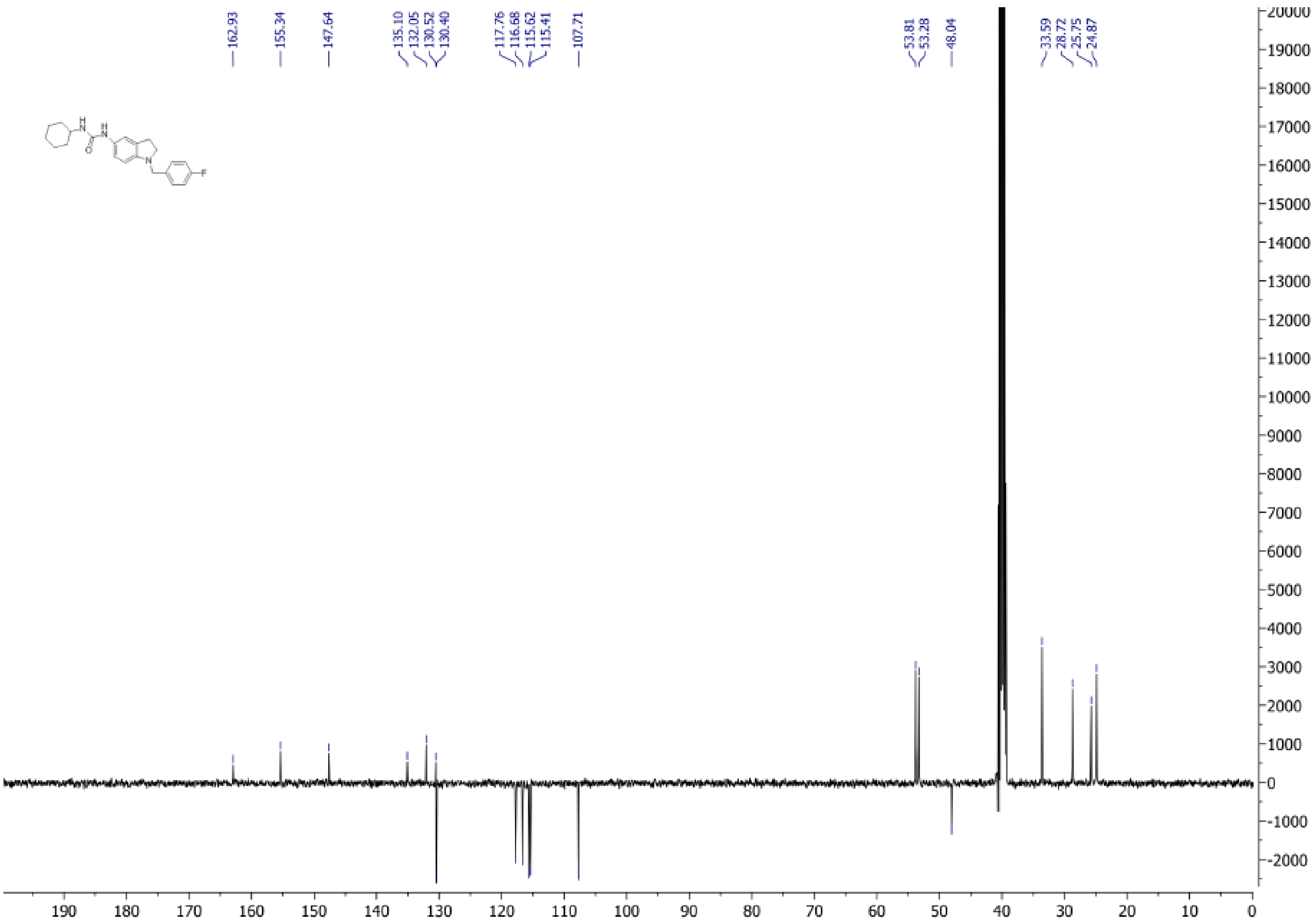

Figure S47: DEPT spectra of compound **53**

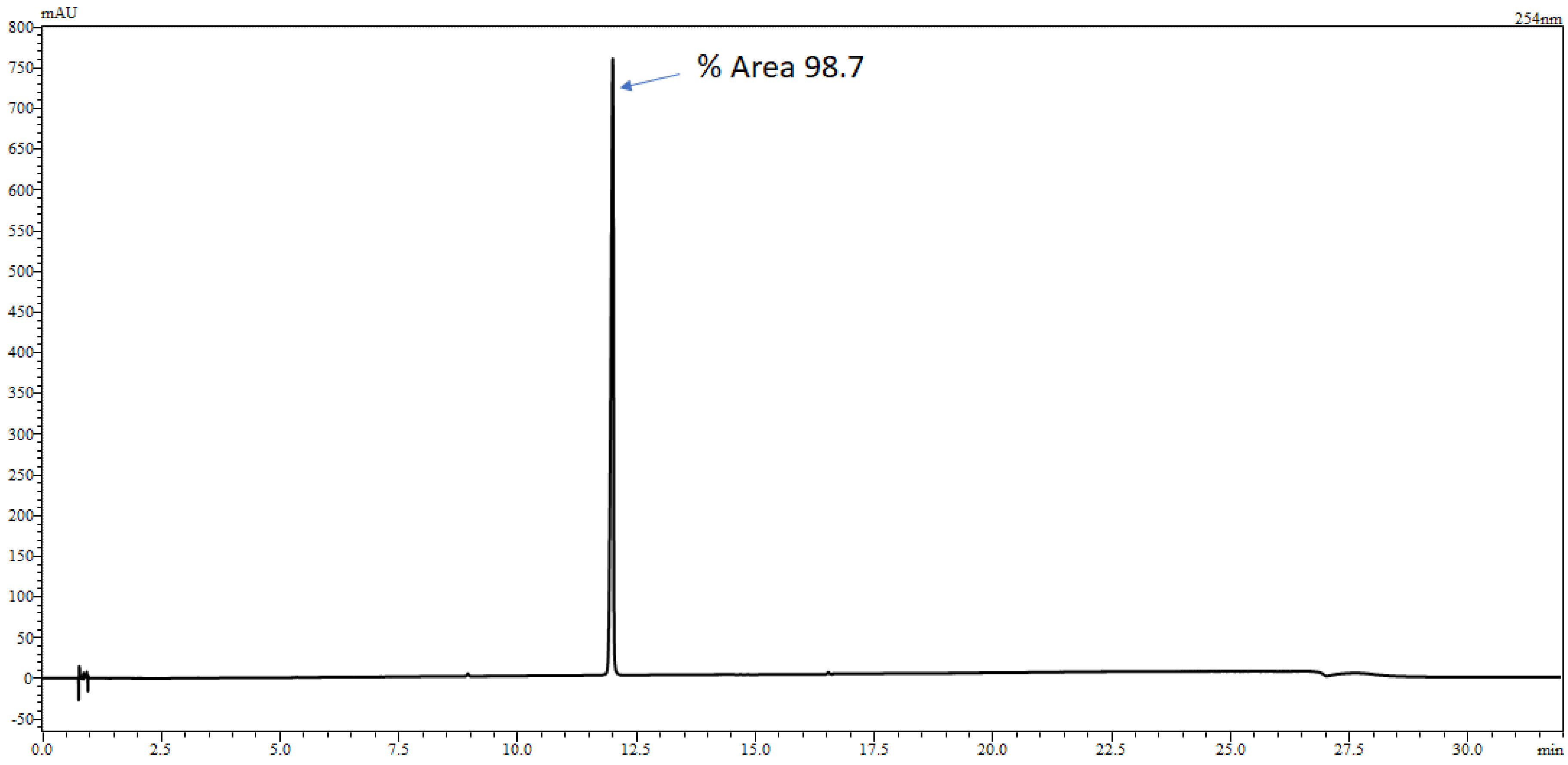

Figure S48: HPLC trace of compound **53**

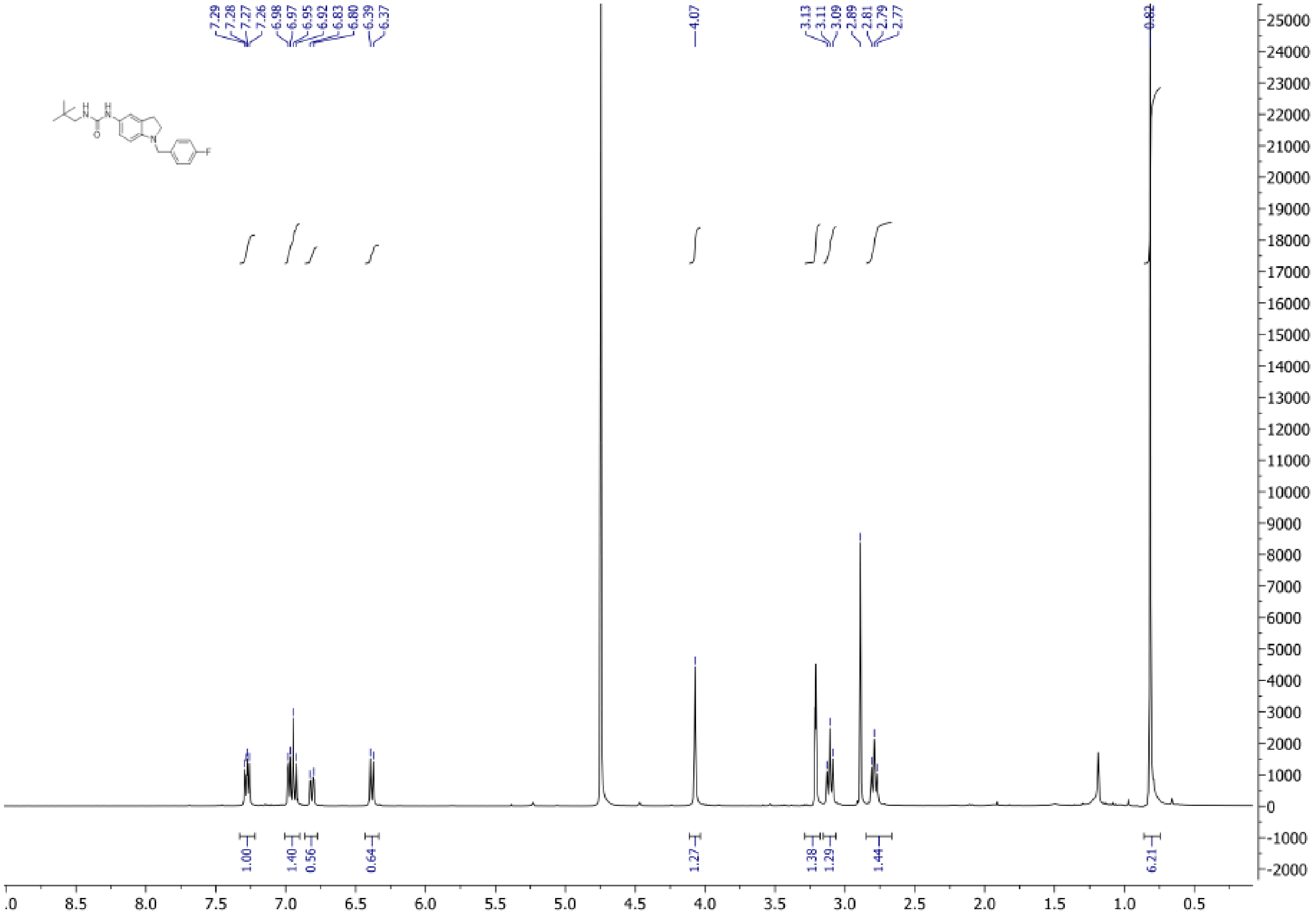

Figure S49: 1H NMR spectra of compound **54**

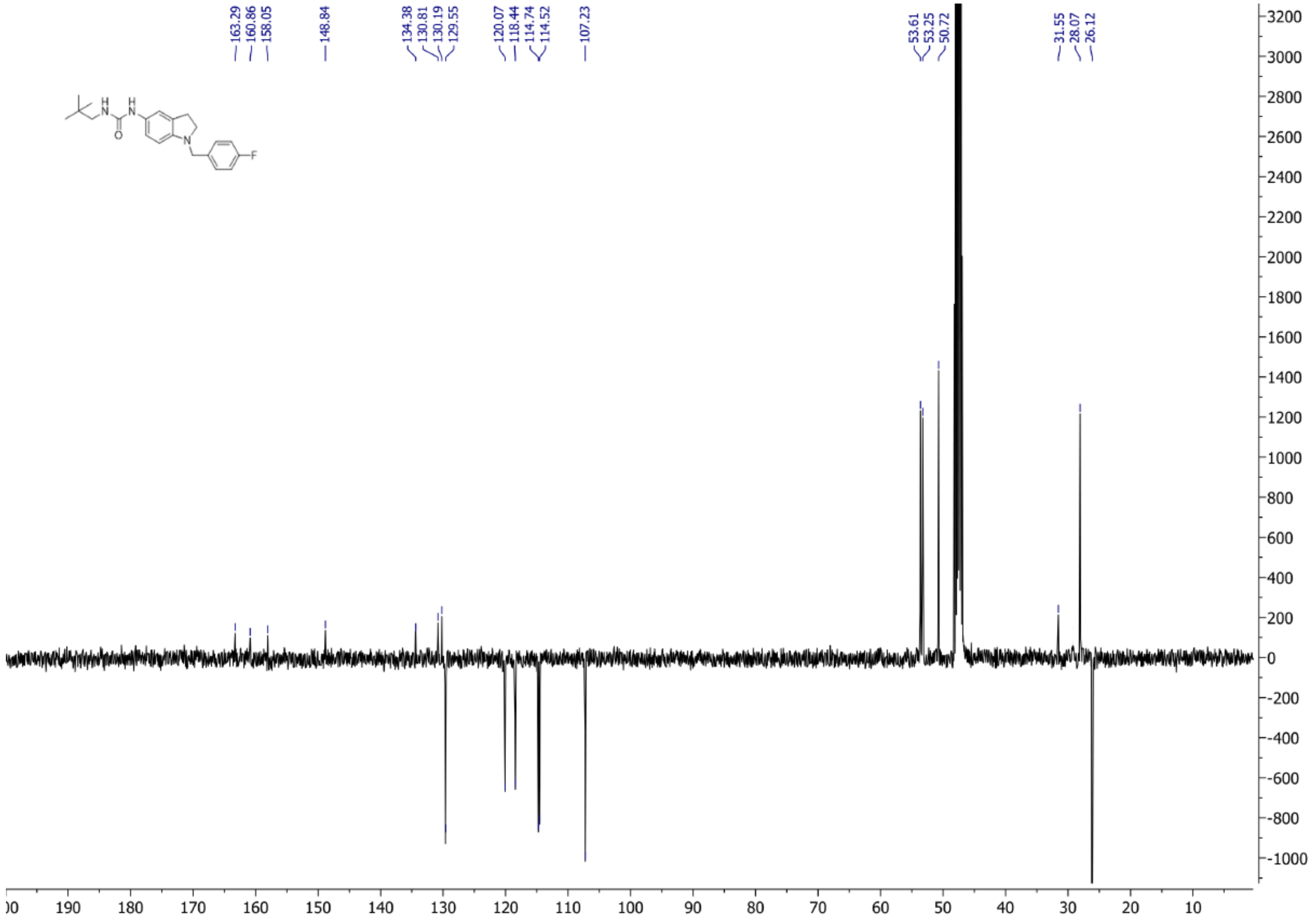

Figure S50: DEPT spectra of compound **54**

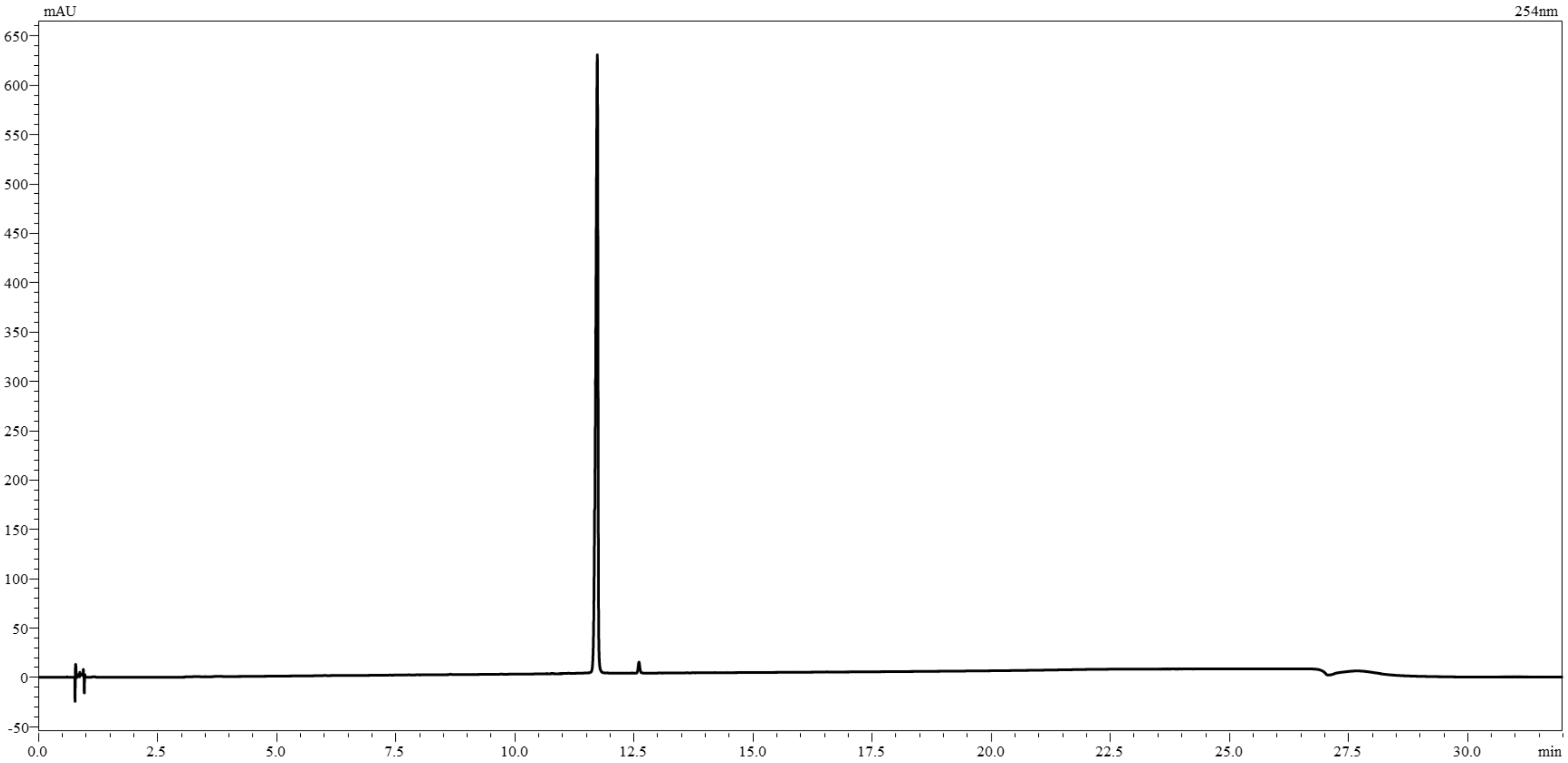

Figure S51: HPLC trace of compound **54**

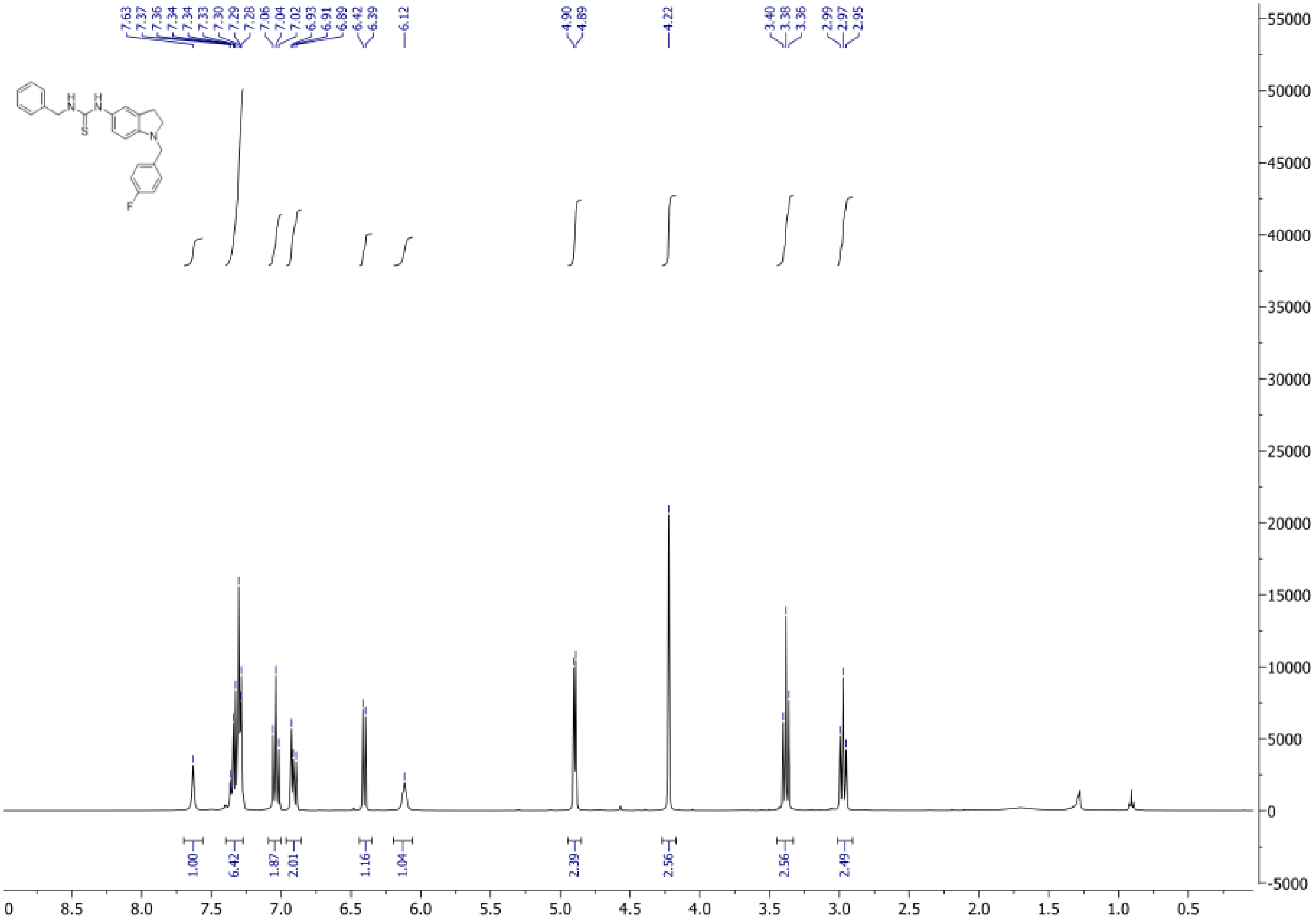

Figure S52: 1H NMR spectra of compound **56**

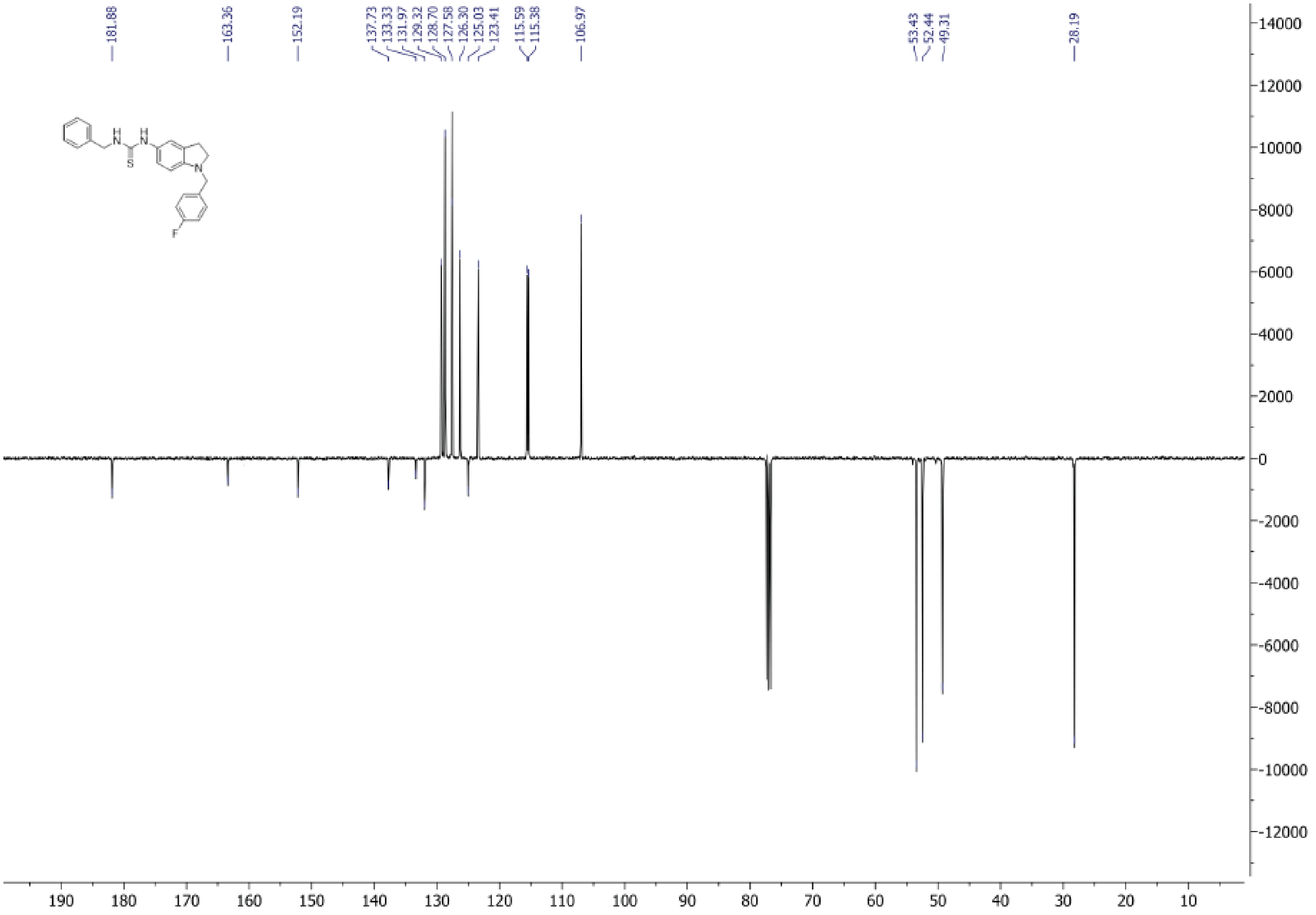

Figure S53: DEPT spectra of compound **56**

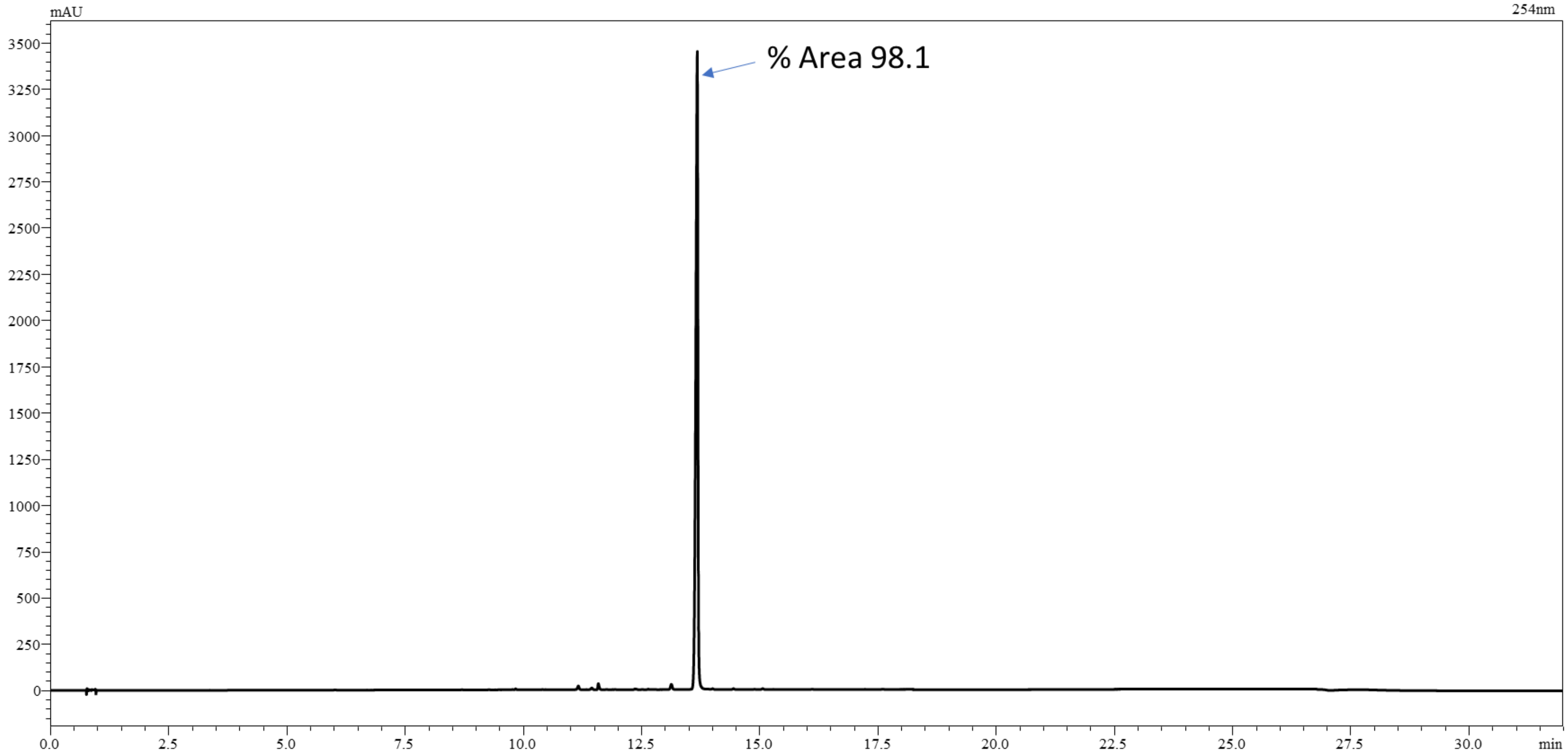

Figure S54: HPLC trace of compound **56**

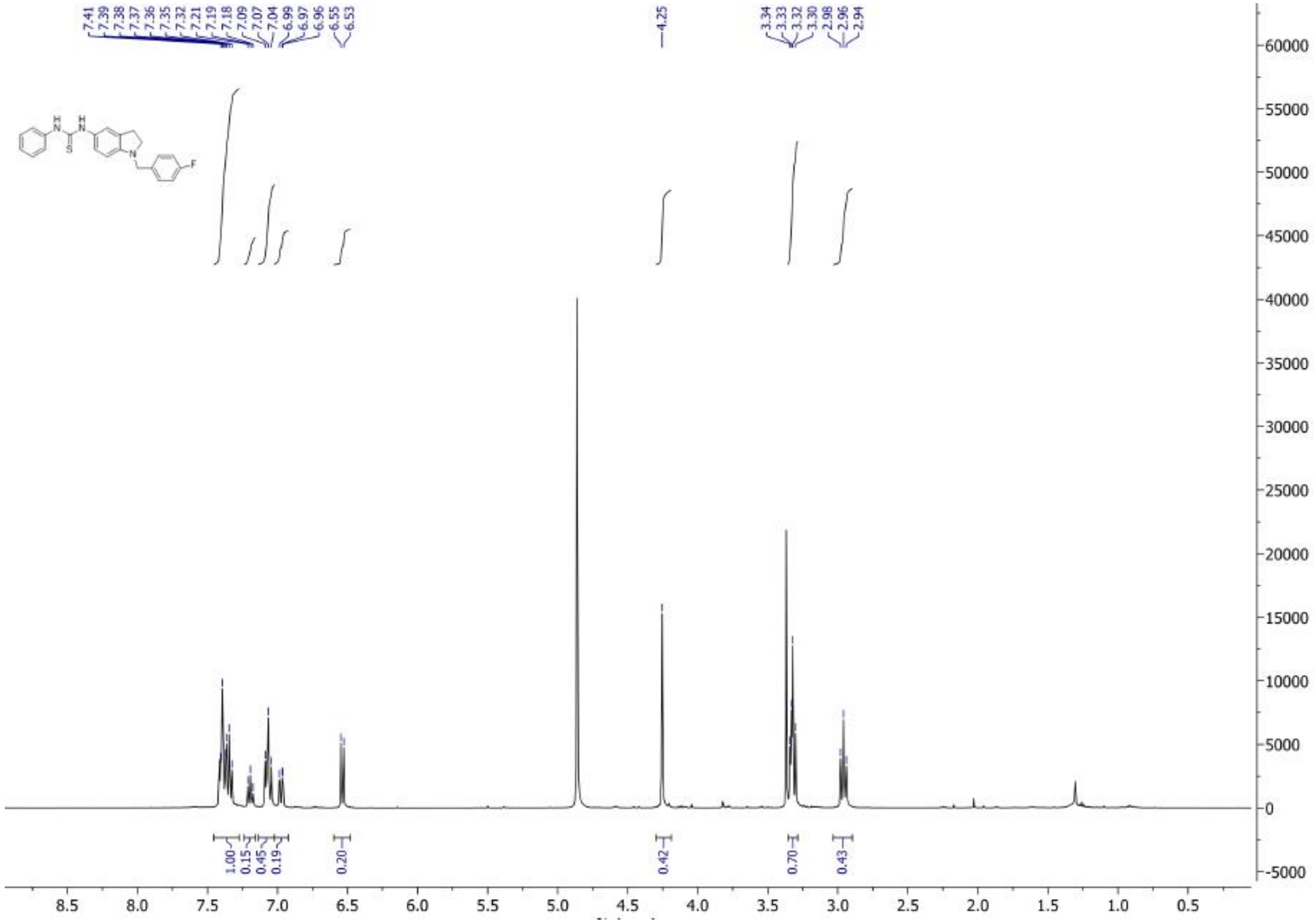

Figure S55: 1H NMR spectra of compound **57**

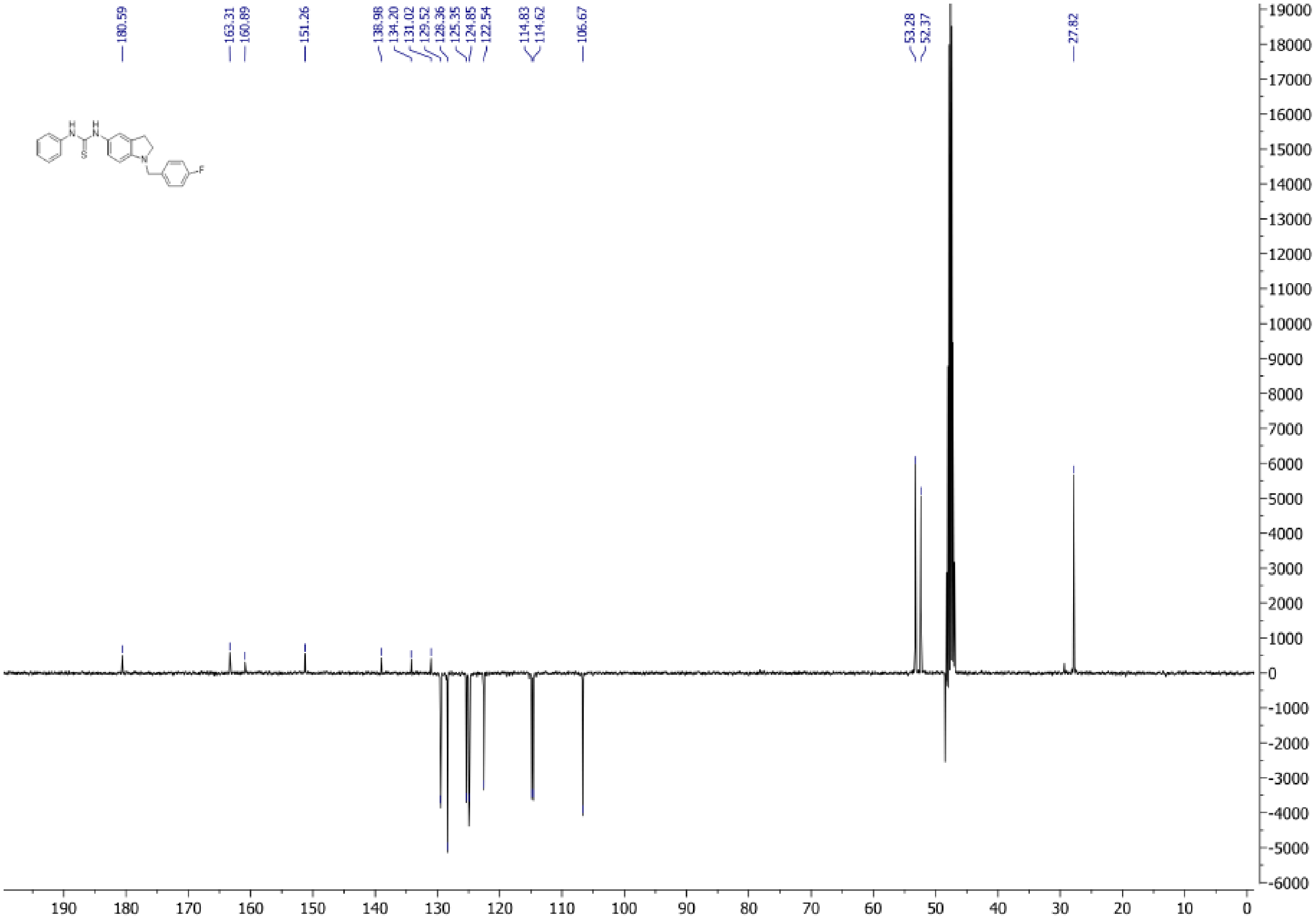

Figure S56: DEPT spectra of compound **57**

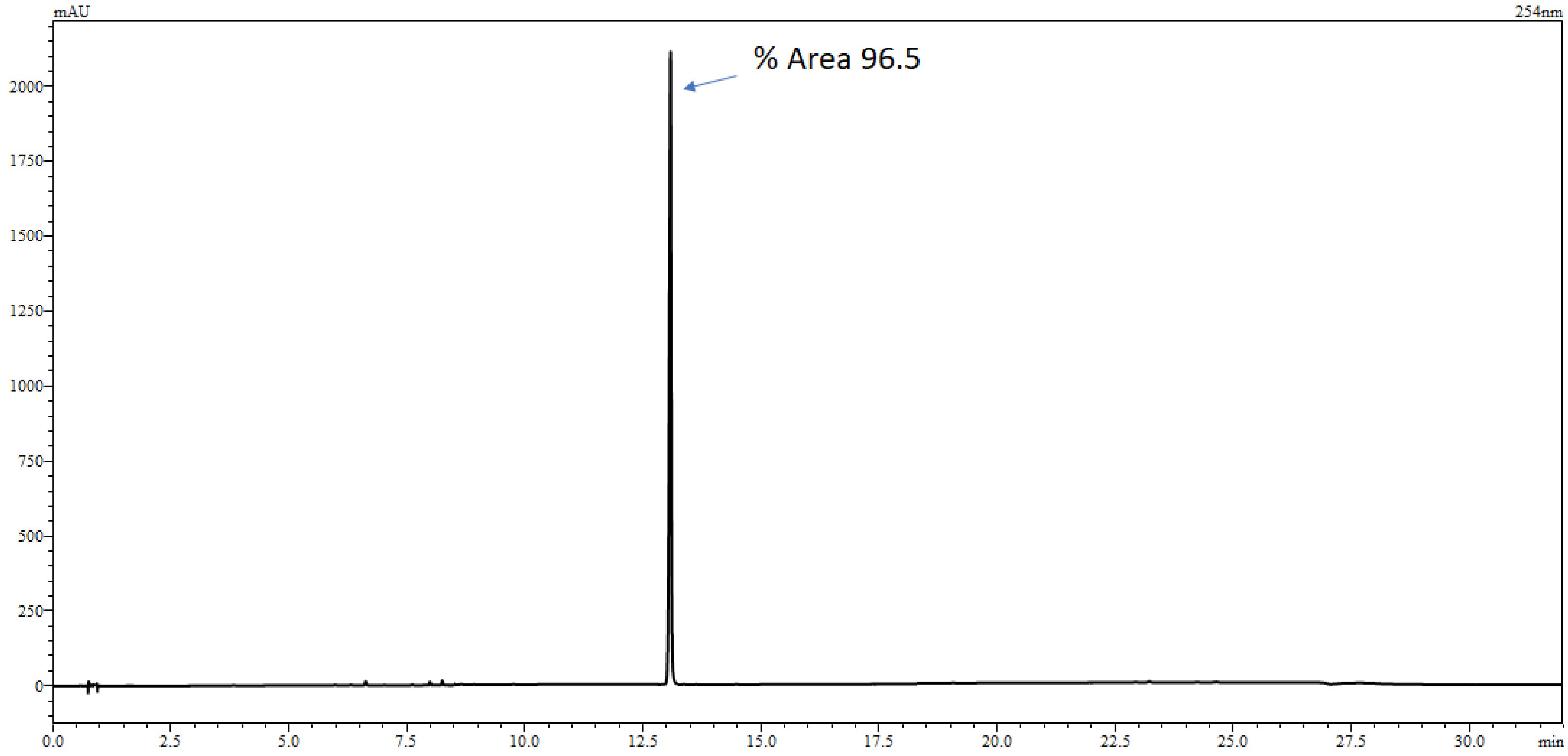

Figure S57: HPLC trace of compound **57**

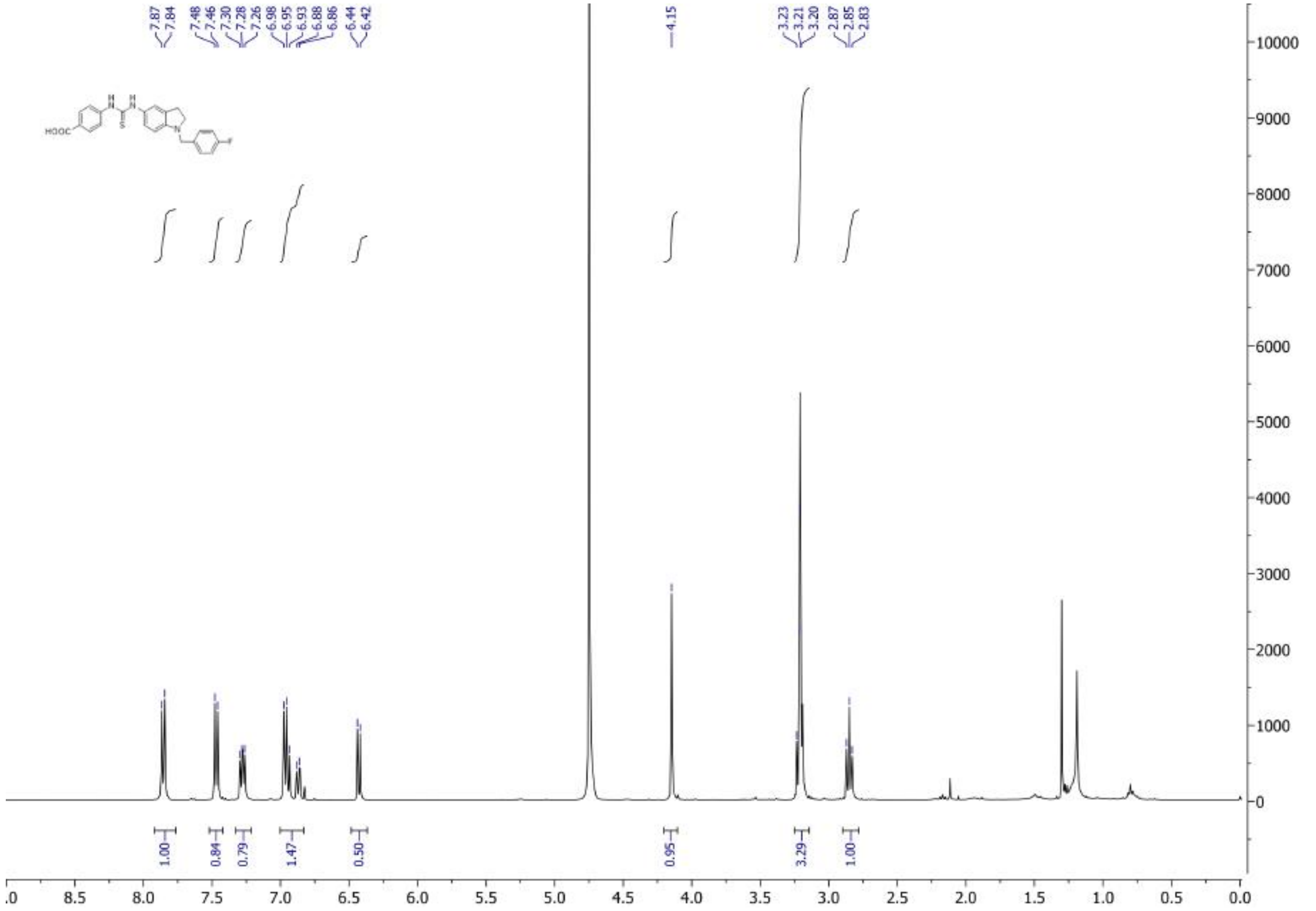

Figure S58: 1H NMR spectra of compound **58**

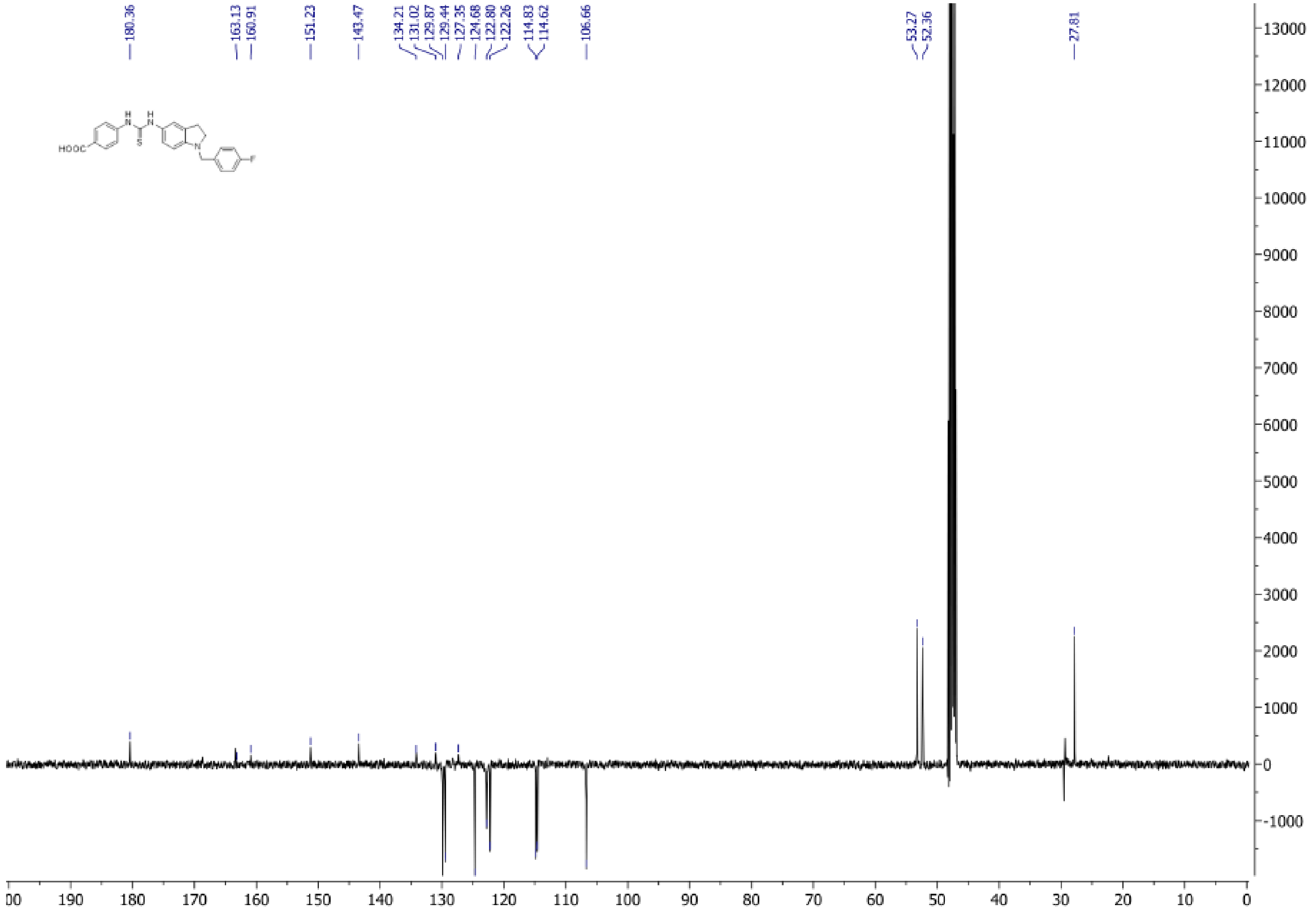

Figure S59: DEPT spectra of compound **58**

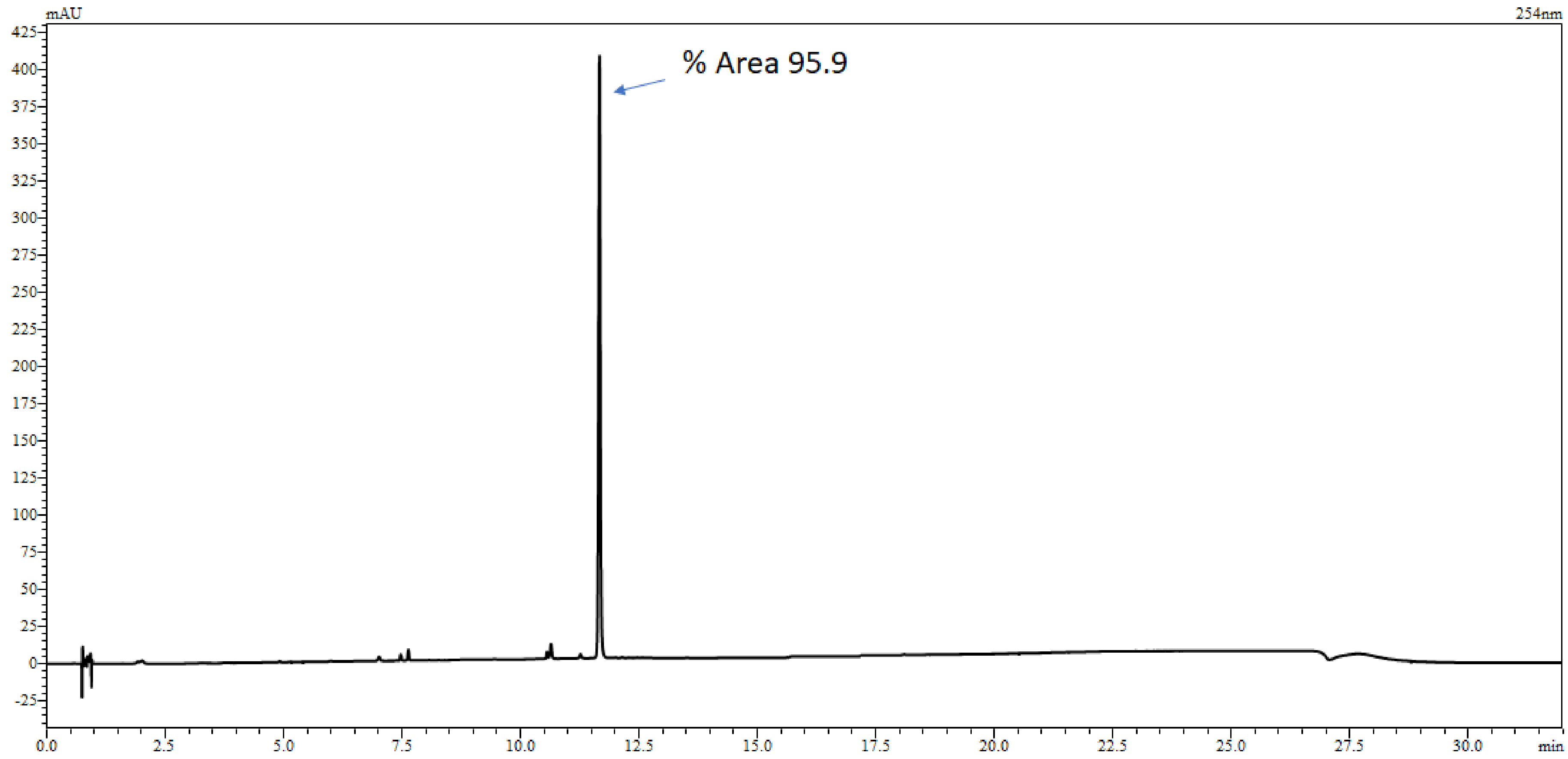

Figure S60: HPLC trace of compound **58**

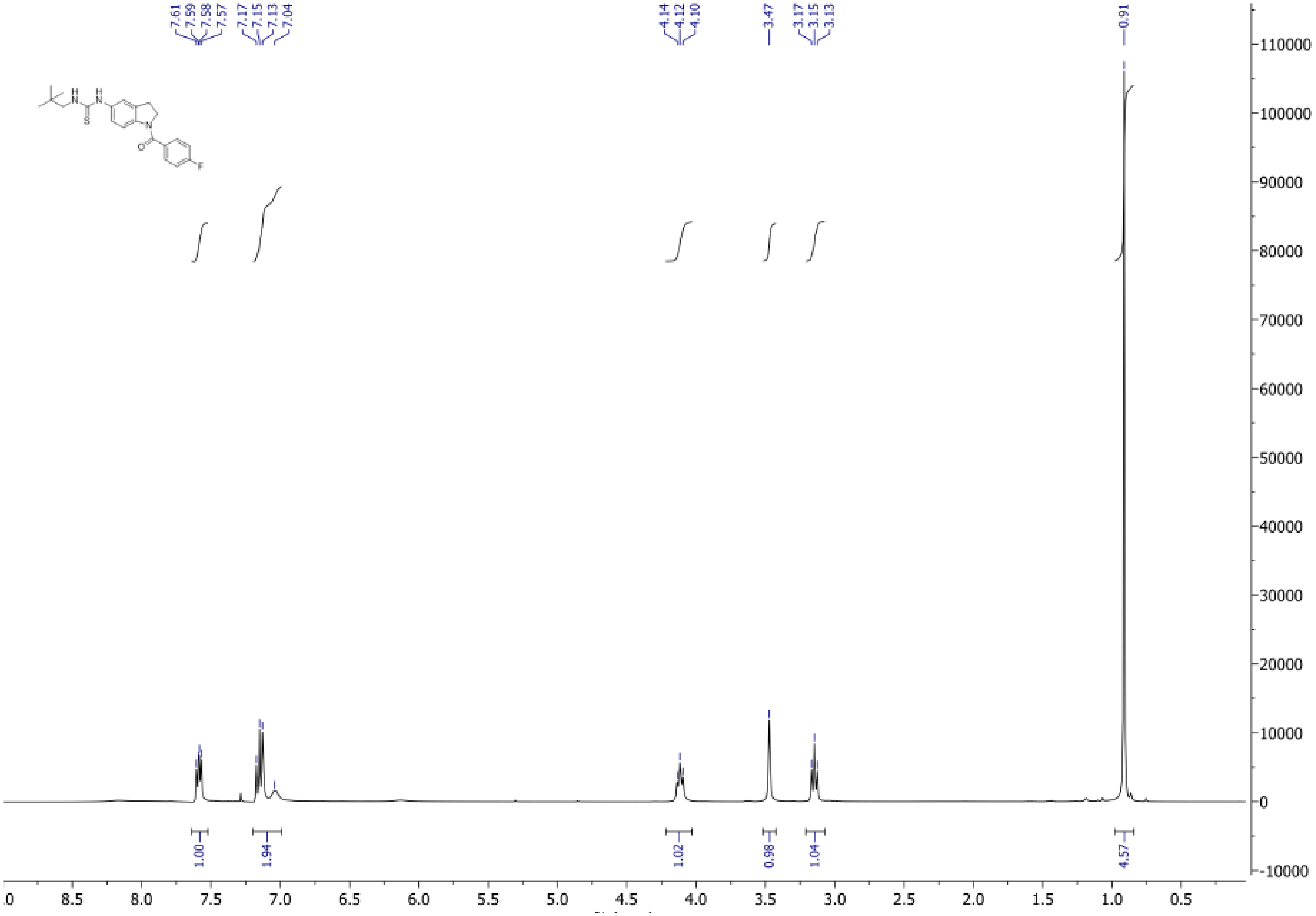

Figure S61: 1H NMR spectra of compound **68**

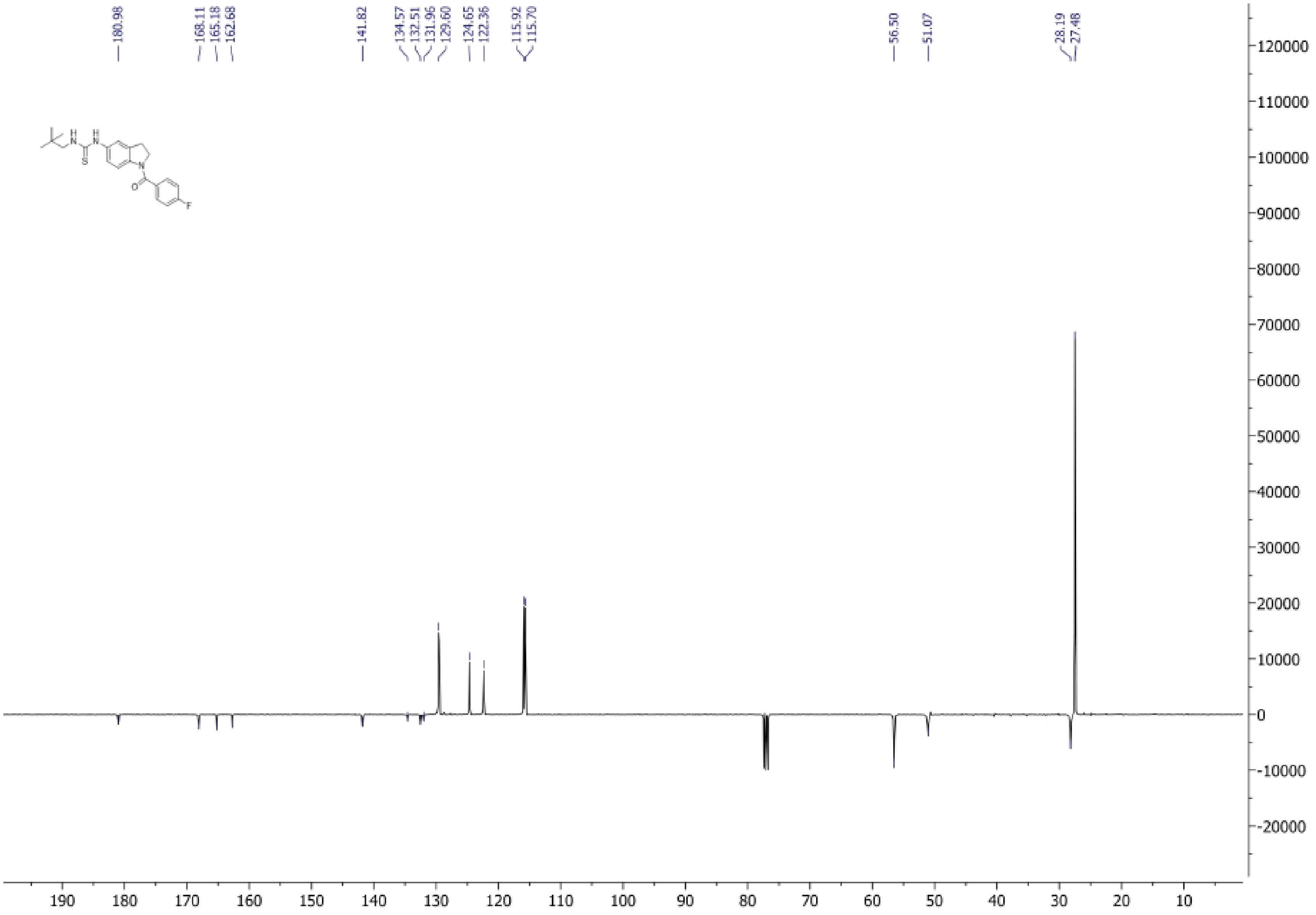

Figure S62: DEPT spectra of compound **68**

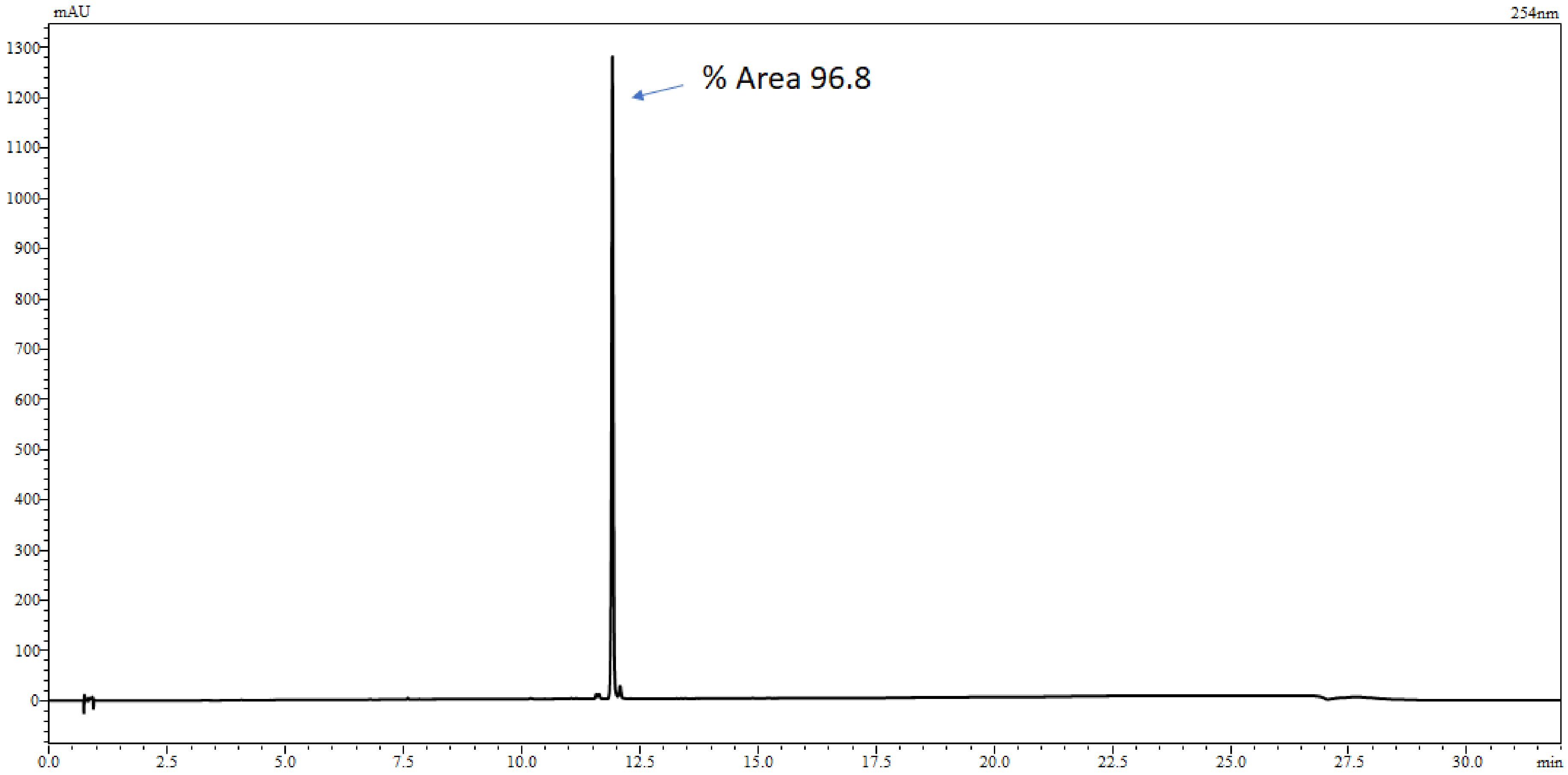

Figure S63: HPLC trace of compound **68**

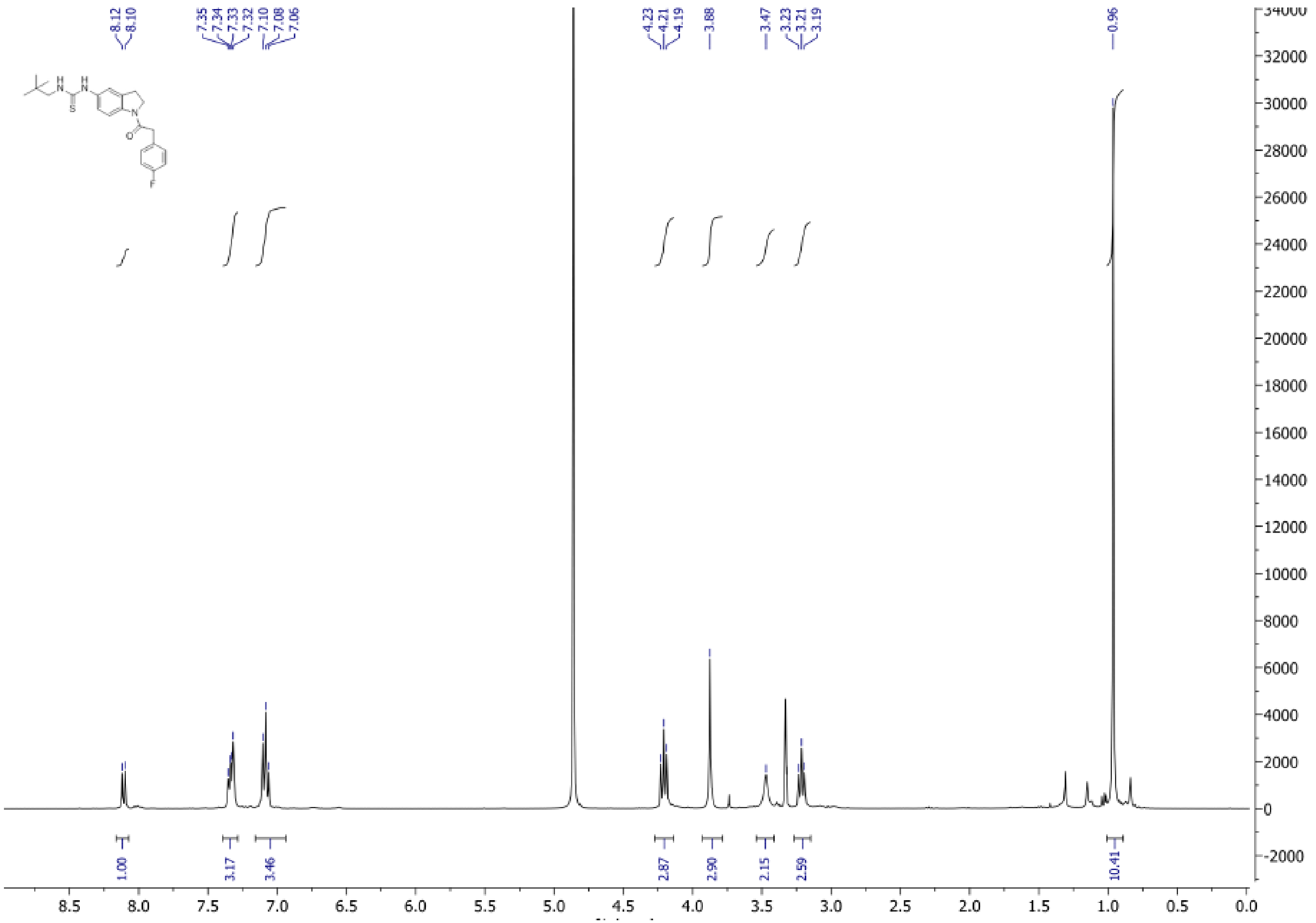

Figure S64: 1H NMR spectra of compound **69**

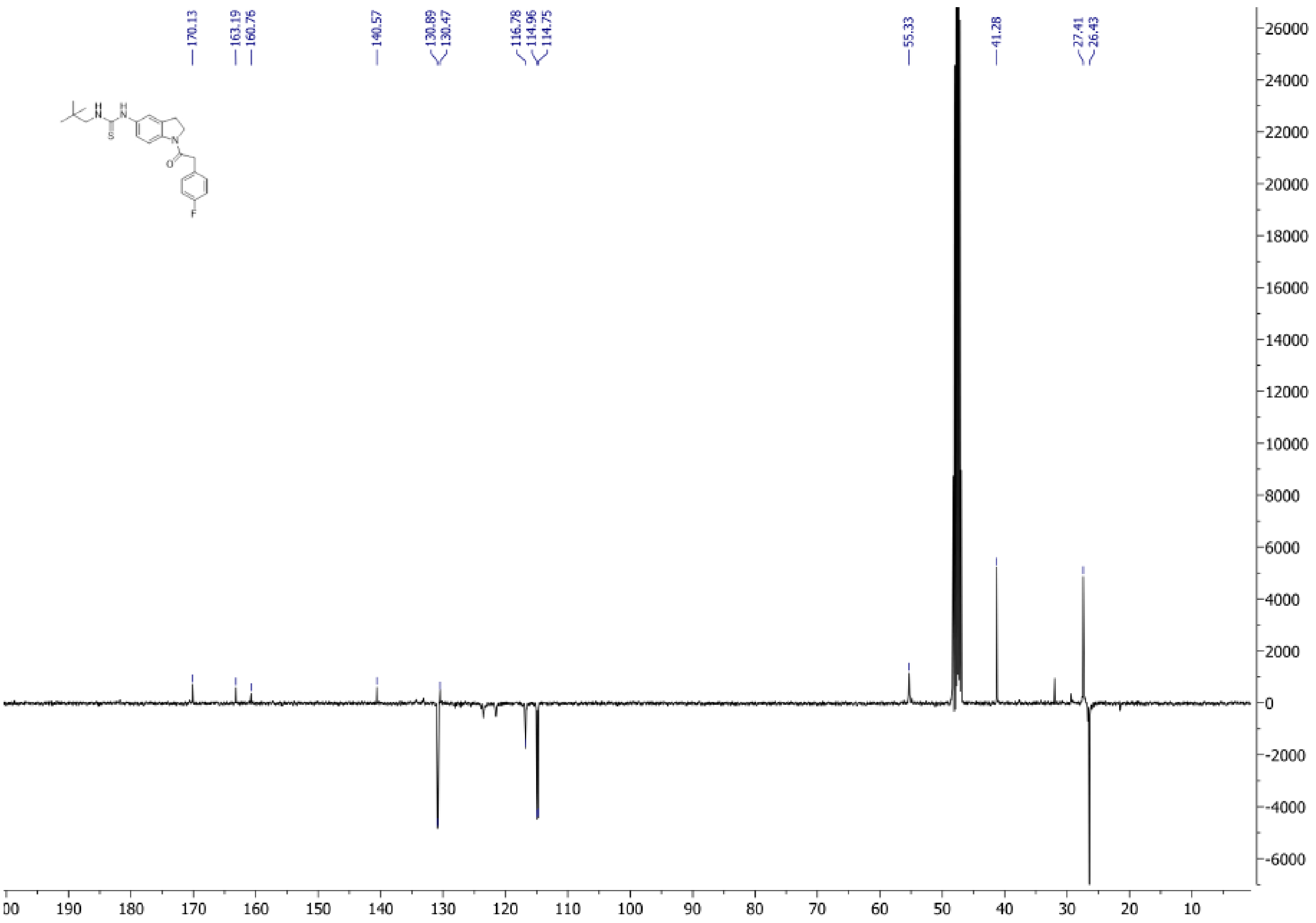

Figure S65: DEPT spectra of compound **69**

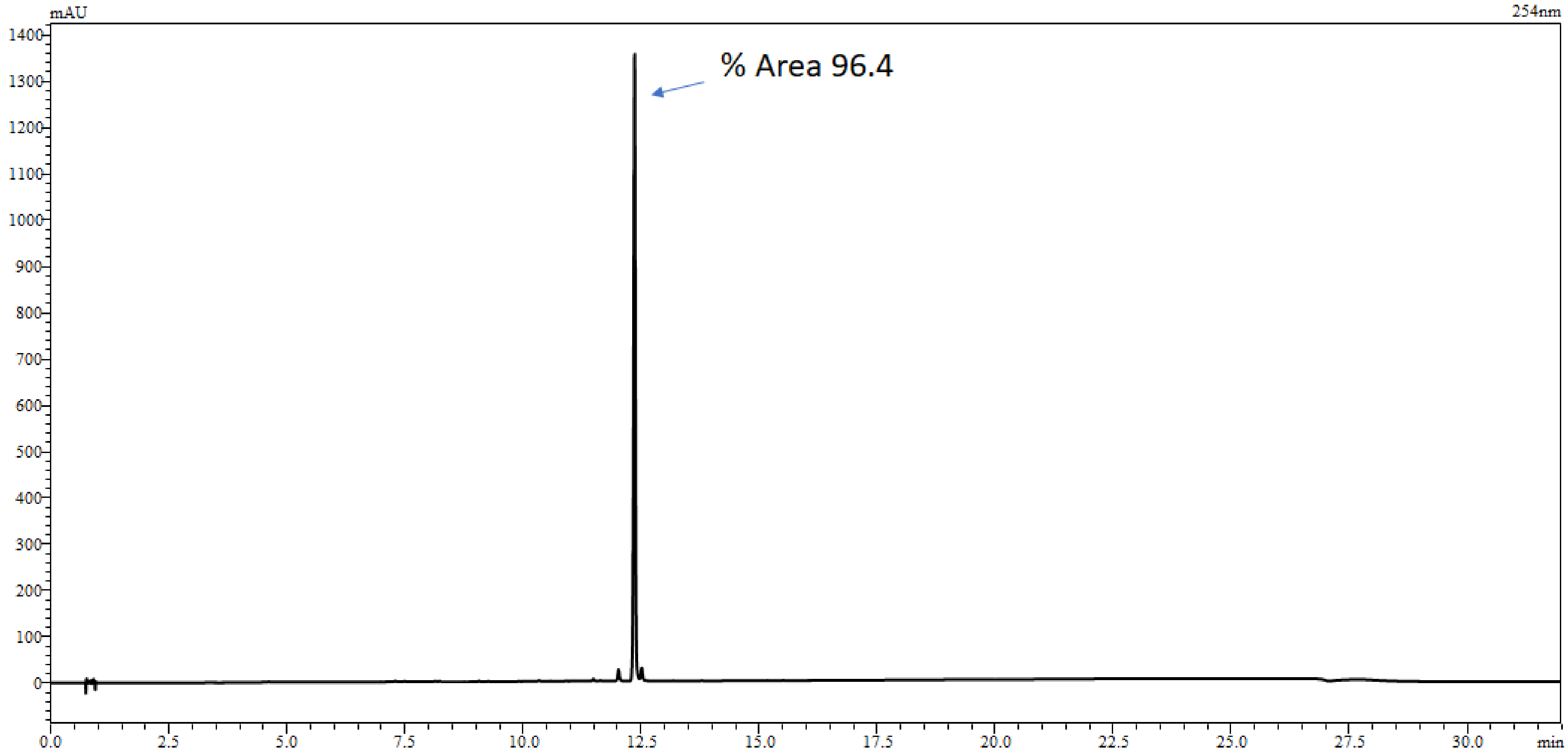

Figure S66: HPLC trace of compound **69**

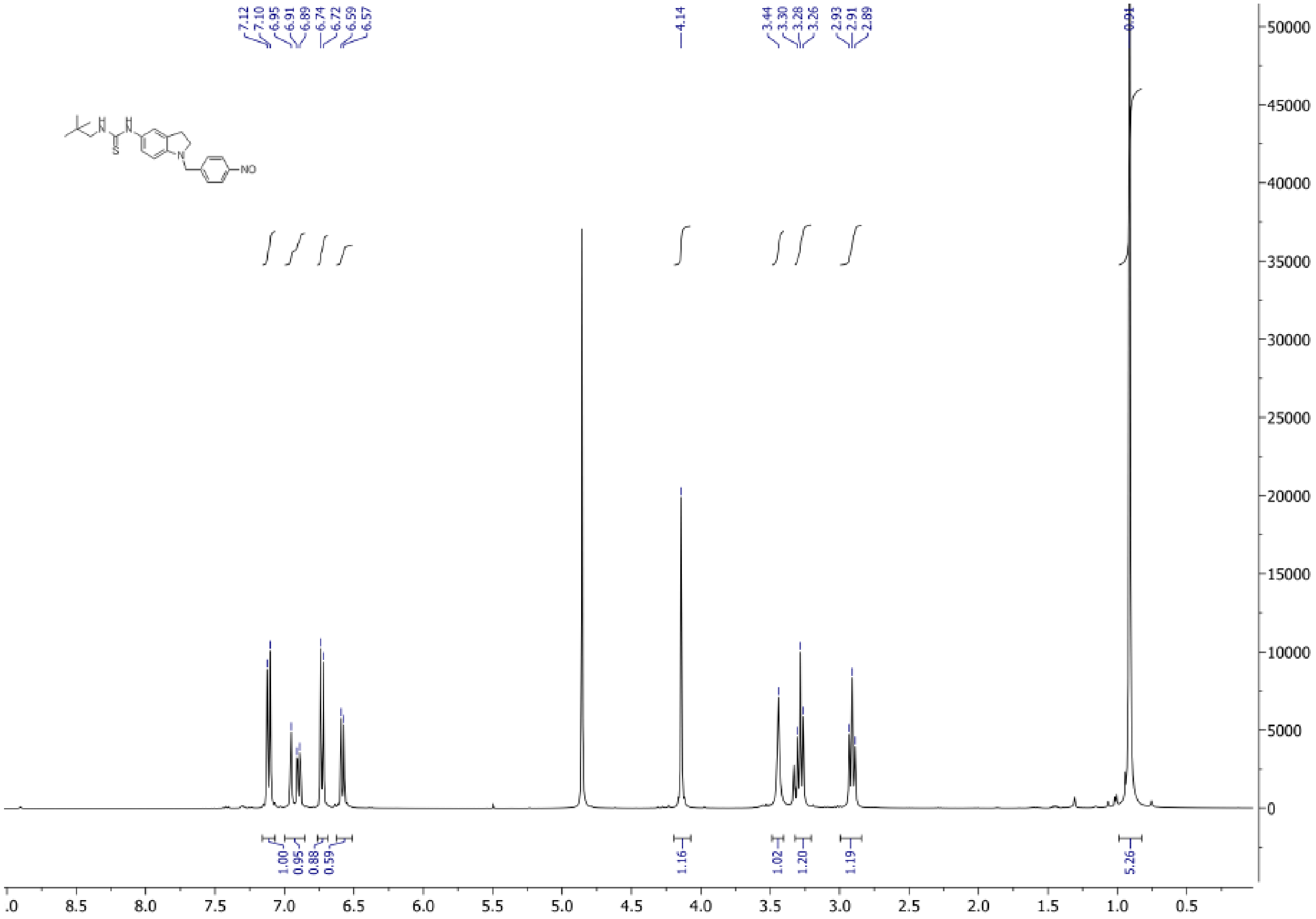

Figure S67: 1H NMR spectra of compound **73**

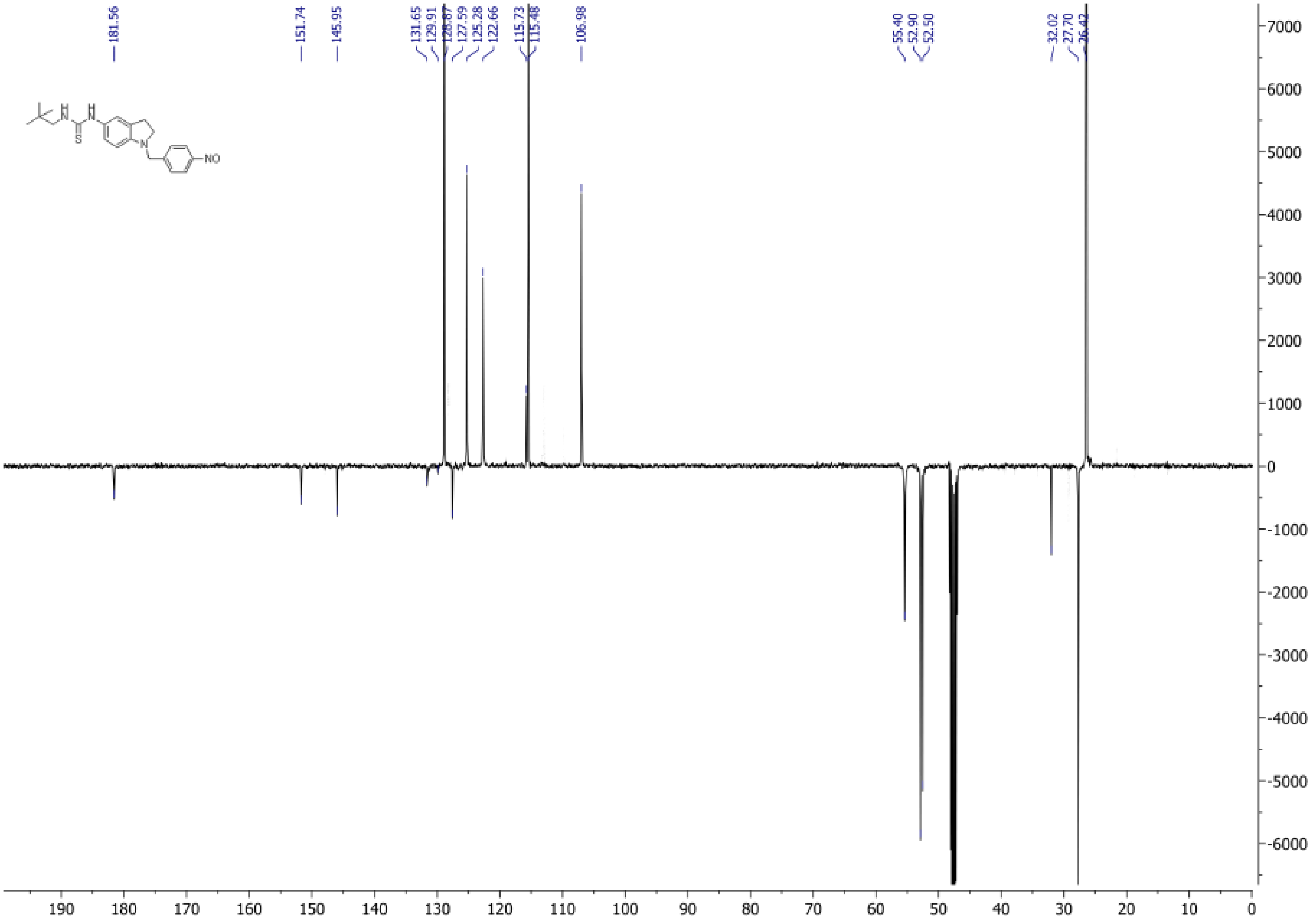

Figure S68: DEPT spectra of compound **73**

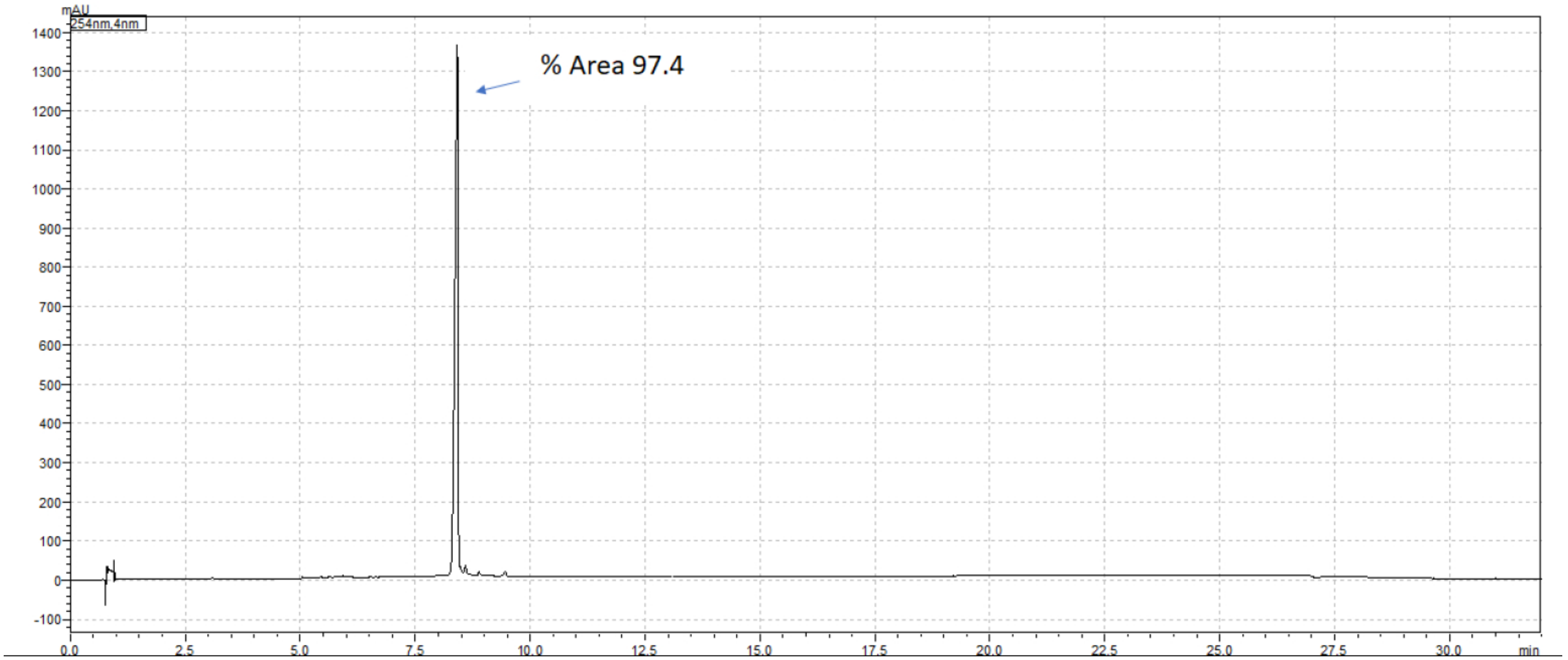

Figure S69: HPLC trace of compound **73**

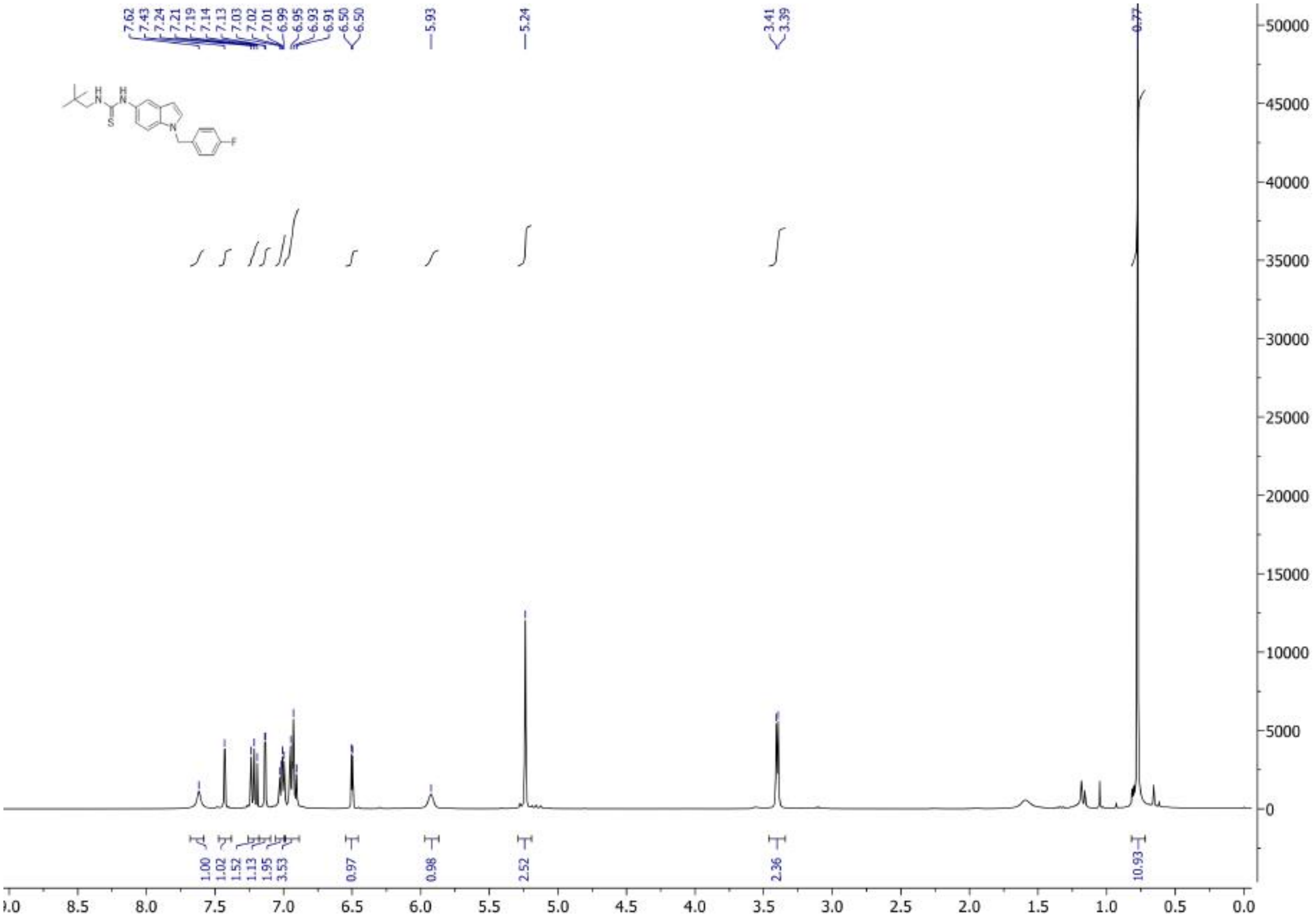

Figure S70: 1H NMR spectra of compound **77**

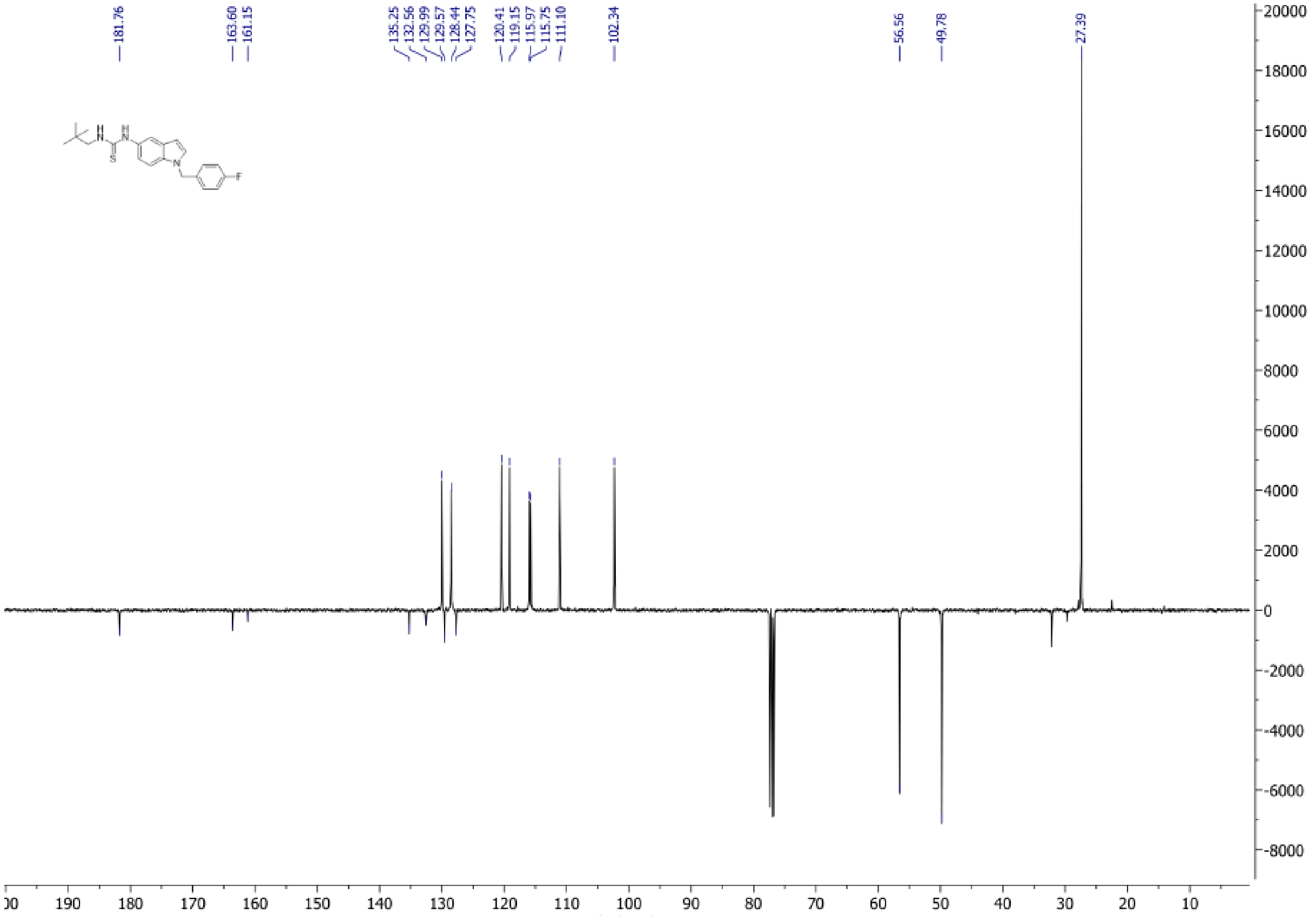

Figure S71: DEPT spectra of compound **77**

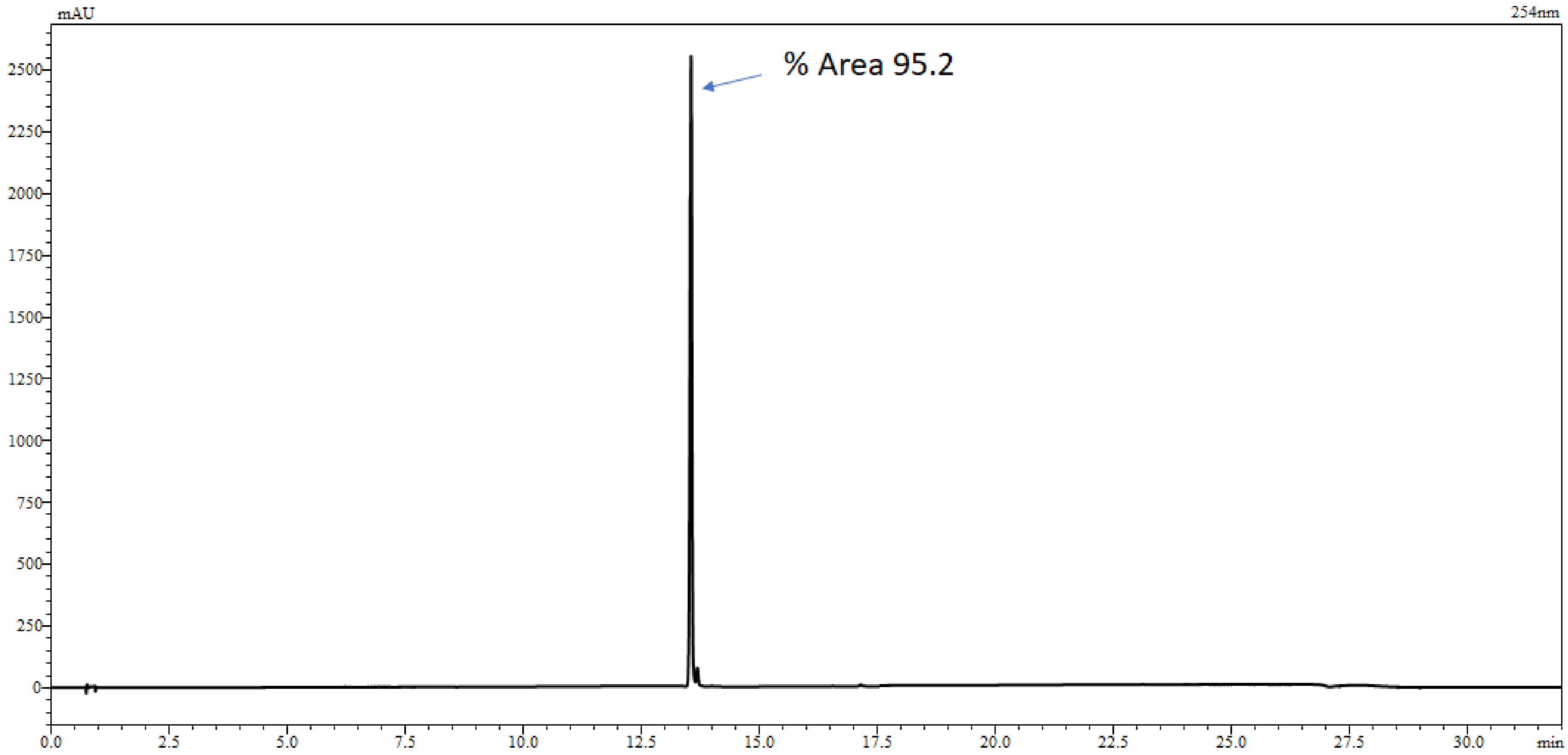

Figure S72: HPLC trace of compound **77**

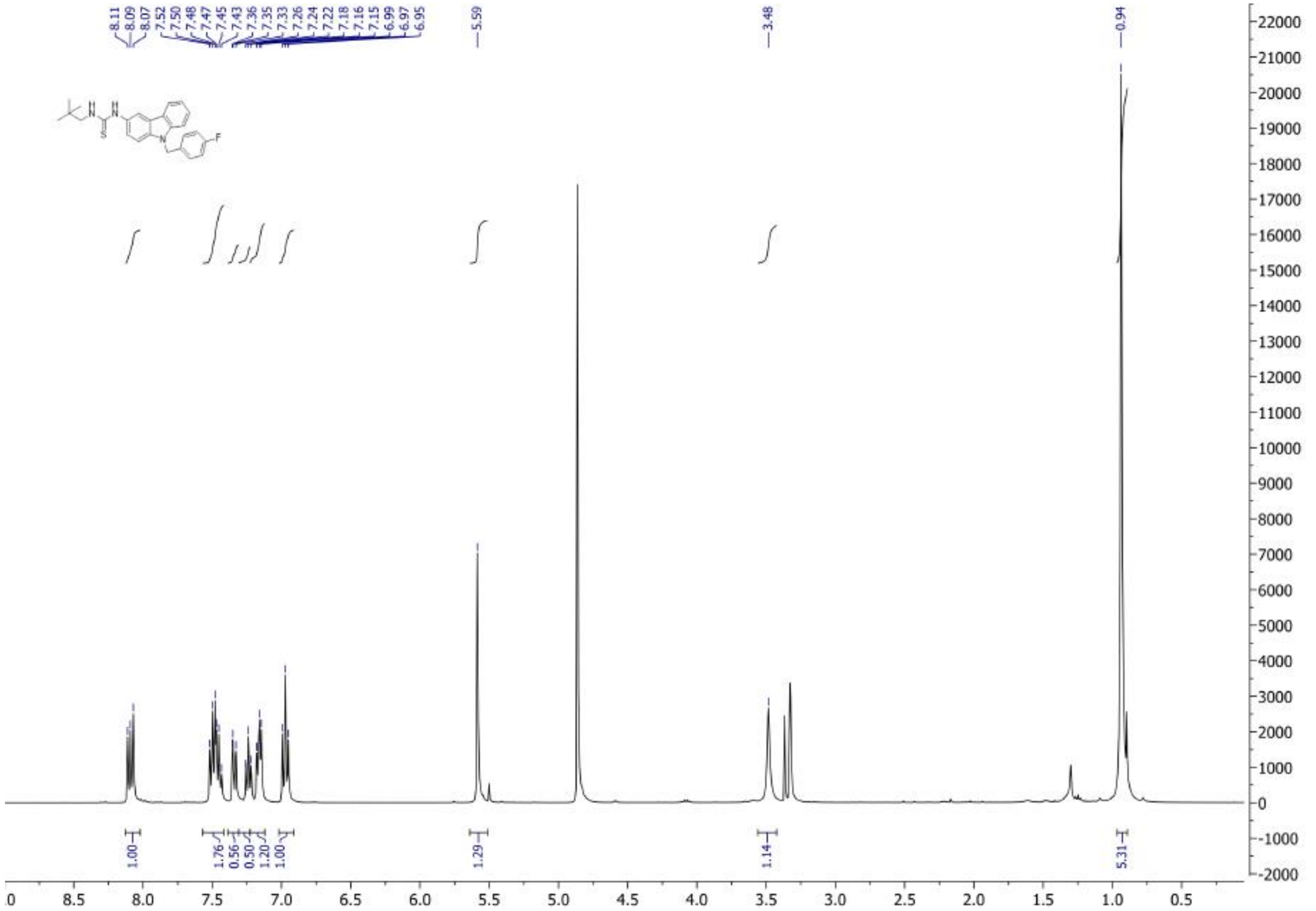

Figure S73: 1H NMR spectra of compound **82**

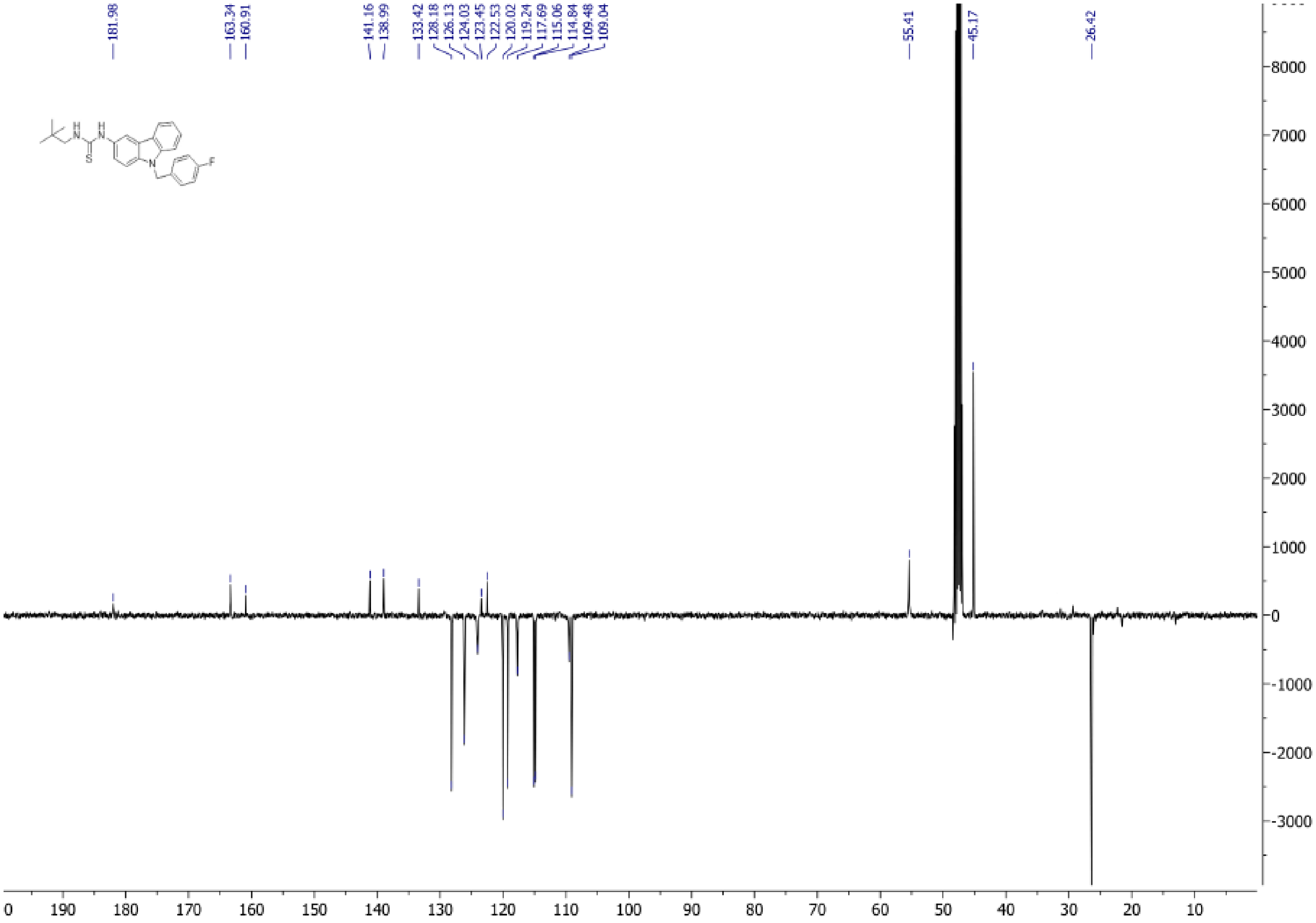

Figure S74: DEPT spectra of compound **82**

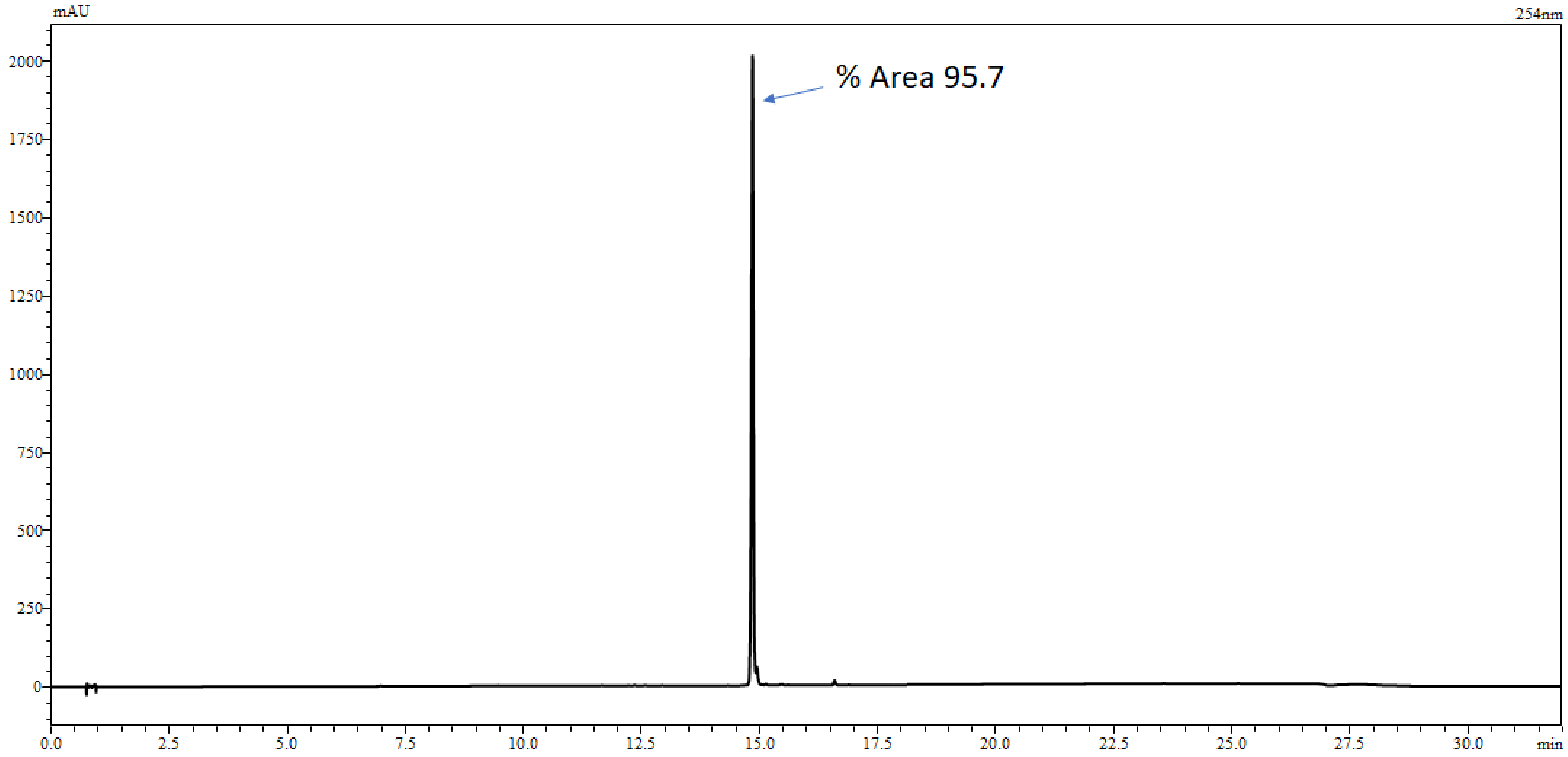

Figure S75: HPLC trace of compound **82**

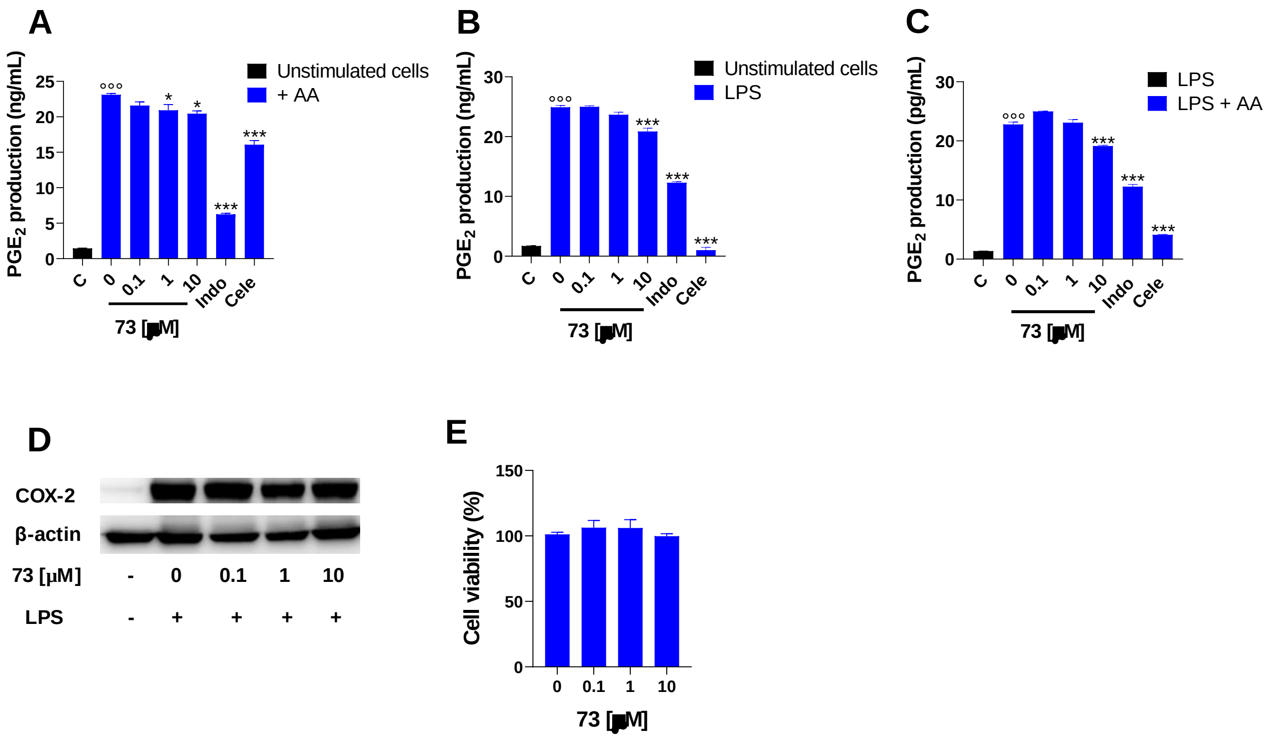

Figure S76: Effect of **73** on COX-1 and COX-2 in intact cells. (A) J774 cells were pre-treated for 2 h with 73 compound (0-10 µM), Indomethacin (Indo, 10 µM) or Celecoxib (Cele, 10 µM) and then incubated with arachidonic acid (15 µM) for 30 min to stimulate COX-1 activity. (B) J774 cells were pre-treated for 2h with test compound (0-10 µM), Indo (10 µM) or Cele (10 µM) and then stimulated for 24 h with LPS (10 µg/mL) to induce COX-2. (C) Cells were stimulated, for 24 h, with LPS (10 µg/mL), to induce COX-2, then pre-treated for 2 h with **73**, Indo or Cele and further incubated for 30 min with AA (15 µM). The supernatants were collected for the measurement of PGE_2 levels by ELISA assay. (D) J774 cells were pre-treated for 2h with test compound (0-10 µM) and then stimulated for 24 h with LPS (10 µg/mL) to induce COX-2 evaluated by western blot. (E) Cell viability was evaluated by the mitochondrial-dependent reduction of MTT to formazan. Values represent means ± S.E.M.; n = 3 experiments. Data were analyzed by one-way ANOVA plus Bonferroni. Statistical significance is reported as follows °°° P < 0.001 vs. unstimulated cells, and * p < 0.05 vs. AA alone (A), *** P < 0.001 vs. LPS alone (B) and LPS + AA (C).

Table S3: Compound **73** concentration in mouse plasma after intraperitoneal administration (10 mg Kg^{-1}). Values are represented as mean with ±SD (n=4). All analyses were performed in triplicate

**Time (h)**	**ng_{drug} / mL_{plasma}**	**$t_{1/2}$**
0.5	15.4 ± 2.5	1.6
1	12.3 ± 0.9	
2	10.6 ± 0.6	
4	1.1 ± 0.1	

Table S4: Optimal LC-MS/MS parameters for Eicosanoids quantification

Peak	Analyte	*m/z* (MS1)	*m/z* (MS2)	Q1 Pre bias	CE (V)	Q3 Pre Bias
**1**	**14,15-DHET**	337.0	207.3	14.0	19.0	13.0
**2**	**11,12-DHET**	337.0	167.2	14.0	21.0	10.0
**3**	**8,9-DHET**	337.0	185.2	18.0	19.0	11.0
			127.2	14.0	23.0	12.0
**4**	**5,6-DHET**	337.1	145.2	14.0	18.0	14.0
**5**	**14,15-EET**	319.0	219.3	13.0	12.0	22.0
			175.4	17.0	15.0	17.0
**6**	**11,12-EET**	319.0	167.3	13.0	15.0	16.0
			208.2	13.0	13.0	13.0
**7**	**8,9-EET**	319.0	127.3	13.0	16.0	12.0
			155.3	13.0	13.0	15.0
**8**	**5,6-EET**	319.1	191.3	13.0	13.0	19.0
**9**	**11,12-DHET-*d*$_{11}$**	348.2	167.3	11.0	20.0	10.0
**10**	**14,15-EET-*d*$_{11}$**	330.2	175.4	18.0	15.0	18.0

DHET: Dihydroxyeicosatrienoic acid; EET: Epoxyeicosatrienoic acid; CE: Collision energy.

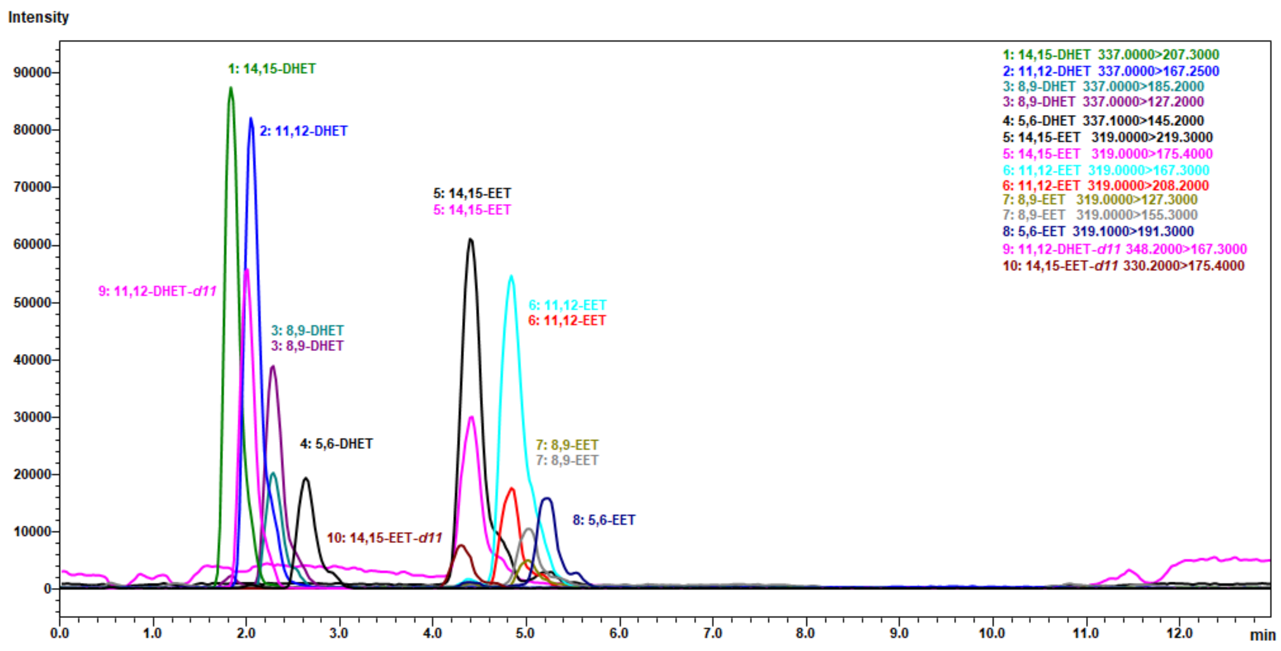

Figure S77: Typical MRM Chromatograms of Eicosanoid standards and their deuterated standards

Table S5: Method validation parameters for quantitation of Dihydroxyeicosatrienoic acids

Parameters	5,6-DHET	8,9-DHET	11,12-DHET	14,15-DHET
**Retention time (min) ± dev. st. (n = 4)**	2.724 ± 0.003	2.358 ± 0.002	2.110 ± 0.002	1.882 ± 0.001
**Regression equation Linear range (5-200 ng mL^{-1})**	y = 25.2718x – 0.59681	y = 18.24249x – 0.83450	y = 6.31708x + 0.40919	y = 6.62982x + 0.03462
**Correlation coefficient (R^2)**	≥ 0.99996	≥ 0.99994	≥ 0.99992	≥ 0.99996
**Intraday (n = 3) Analyte concentration (ng mL^{-1}): Accuracy (%)**	10 : 95<br>25 : 99<br>50 : 100	10 : 104<br>25 : 98<br>50 : 98	10 : 91<br>25 : 103<br>50 : 101	10 : 96<br>25 : 99<br>50 : 100
**Interday (n = 3) Analyte concentration (ng mL^{-1}): Accuracy (%)**	10 : 96<br>25 : 98<br>50 : 103	10 : 95<br>25 : 97<br>50 : 102	10 : 96<br>25 : 96<br>50 : 104	10 : 93<br>25 : 98<br>50 : 102
**LOQ (ng mL^{-1})**	1.59	3.14	0.25	0.46
**LOD (ng mL^{-1})**	0.52	1.04	0.08	0.15

LOQ: 10 × (standard deviation of the response/slope of calibration curve); LOD: 3.3 × (standard deviation of the response/slope of calibration curve).

Table S6: Method validation parameters for quantitation of Epoxyeicosatrienoic acids

Parameters	5,6-EET	8,9-EET	11,12-EET	14,15-EET
**Retention time (min) ± dev. st. (n = 4)**	5.360 ± 0.002	5.162 ± 0.009	4.976 ± 0.005	4.542 ± 0.008
**Regression equation Linear range (2.5-200 ng mL^{-1})**	y = 5.68908x – 2.27453	y = 8.63471x – 0.78802	y = 1.80165x – 0.50401	y = 4.06231x – 0.75328
**Correlation coefficient (R^2)**	≥ 0.99989	≥ 0.99992	≥ 0.99986	≥ 0.99987
**Intraday (n = 3) Analyte concentration (ng mL^{-1}): Accuracy (%)**	10 : 100<br>25 : 96<br>50 : 98	10 : 101<br>25 : 97<br>50 : 100	10 : 96<br>25 : 95<br>50 : 102	10 : 99<br>25 : 94<br>50 : 101
**Interday (n = 3) Analyte concentration (ng mL^{-1}): Accuracy (%)**	10 : 94<br>25 : 98<br>50 : 103	10 : 95<br>25 : 95<br>50 : 103	10 : 96<br>25 : 96<br>50 : 102	10 : 94<br>25 : 99<br>50 : 103
**LOQ (ng mL^{-1})**	1.69	1.15	0.99	1.10
**LOD (ng mL^{-1})**	0.56	0.38	0.33	0.37

LOQ: 10 × (standard deviation of the response/slope of calibration curve); LOD: 3.3 × (standard deviation of the response/slope of calibration curve).
